# FunlncModel: integrating multi-omic features from upstream and downstream regulatory networks into a machine learning framework to identify functional lncRNAs

**DOI:** 10.1093/bib/bbae623

**Published:** 2024-11-27

**Authors:** Yan-Yu Li, Feng-Cui Qian, Guo-Rui Zhang, Xue-Cang Li, Li-Wei Zhou, Zheng-Min Yu, Wei Liu, Qiu-Yu Wang, Chun-Quan Li

**Affiliations:** The First Affiliated Hospital & National Health Commission Key Laboratory of Birth Defect Research and Prevention, Hengyang Medical School, University of South China, Hengyang, Hunan, 421001, China; Hunan Provincial Key Laboratory of Multi-omics and Artificial Intelligence of Cardiovascular Diseases, University of South China, Hengyang, Hunan, 421001, China; School of Computer, University of South China, Hengyang, Hunan, 421001, China; Institute of Biochemistry and Molecular Biology, Hengyang Medical College, University of South China, Hengyang, Hunan, 421001, China; The First Affiliated Hospital & National Health Commission Key Laboratory of Birth Defect Research and Prevention, Hengyang Medical School, University of South China, Hengyang, Hunan, 421001, China; Hunan Provincial Key Laboratory of Multi-omics and Artificial Intelligence of Cardiovascular Diseases, University of South China, Hengyang, Hunan, 421001, China; School of Computer, University of South China, Hengyang, Hunan, 421001, China; Institute of Biochemistry and Molecular Biology, Hengyang Medical College, University of South China, Hengyang, Hunan, 421001, China; Institute of Biochemistry and Molecular Biology, Hengyang Medical College, University of South China, Hengyang, Hunan, 421001, China; School of Medical Informatics, Daqing Campus, Harbin Medical University, Daqing, 163000, China; State Key Laboratory of Stem Cell and Reproductive Biology, Institute of Zoology, Chinese Academy of Sciences, Beijing, 100101, China; School of Computer, University of South China, Hengyang, Hunan, 421001, China; College of Science, Heilongjiang Institute of Technology, Harbin, Heilongjiang, 150000, China; The First Affiliated Hospital & National Health Commission Key Laboratory of Birth Defect Research and Prevention, Hengyang Medical School, University of South China, Hengyang, Hunan, 421001, China; Hunan Provincial Key Laboratory of Multi-omics and Artificial Intelligence of Cardiovascular Diseases, University of South China, Hengyang, Hunan, 421001, China; School of Computer, University of South China, Hengyang, Hunan, 421001, China; Institute of Biochemistry and Molecular Biology, Hengyang Medical College, University of South China, Hengyang, Hunan, 421001, China; The First Affiliated Hospital & National Health Commission Key Laboratory of Birth Defect Research and Prevention, Hengyang Medical School, University of South China, Hengyang, Hunan, 421001, China; Hunan Provincial Key Laboratory of Multi-omics and Artificial Intelligence of Cardiovascular Diseases, University of South China, Hengyang, Hunan, 421001, China; Key Laboratory of Rare Pediatric Diseases, Ministry of Education, University of South China, Hengyang, Hunan, 421001, China; School of Computer, University of South China, Hengyang, Hunan, 421001, China; Institute of Biochemistry and Molecular Biology, Hengyang Medical College, University of South China, Hengyang, Hunan, 421001, China

**Keywords:** multi-omics analysis, functional lncRNA, upstream/downstream regulatory network analysis, machine learning algorithm, systems biology

## Abstract

Accumulating evidence indicates that long noncoding RNAs (lncRNAs) play important roles in molecular and cellular biology. Although many algorithms have been developed to reveal their associations with complex diseases by using downstream targets, the upstream (epi)genetic regulatory information has not been sufficiently leveraged to predict the function of lncRNAs in various biological processes. Therefore, we present FunlncModel, a machine learning–based interpretable computational framework, which aims to screen out functional lncRNAs by integrating a large number of (epi)genetic features and functional genomic features from their upstream/downstream multi-omic regulatory networks. We adopted the random forest method to mine nearly 60 features in three categories from >2000 datasets across 11 data types, including transcription factors (TFs), histone modifications, typical enhancers, super-enhancers, methylation sites, and mRNAs. FunlncModel outperformed alternative methods for classification performance in human embryonic stem cell (hESC) (0.95 Area Under Curve (AUROC) and 0.97 Area Under the Precision-Recall Curve (AUPRC)). It could not only infer the most known lncRNAs that influence the states of stem cells, but also discover novel high-confidence functional lncRNAs. We extensively validated FunlncModel’s efficacy by up to 27 cancer-related functional prediction tasks, which involved multiple cancer cell growth processes and cancer hallmarks. Meanwhile, we have also found that (epi)genetic regulatory features, such as TFs and histone modifications, serve as strong predictors for revealing the function of lncRNAs. Overall, FunlncModel is a strong and stable prediction model for identifying functional lncRNAs in specific cellular contexts. FunlncModel is available as a web server at https://bio.liclab.net/FunlncModel/.

## Introduction

Long noncoding RNAs (lncRNAs) are a class of non-protein-coding RNA molecules with >200 nucleotide transcripts [1–4]. As emerging key regulators, lncRNAs have been proposed to perform specific functions in diverse processes, including cell self-renewal, proliferation, differentiation, and disease [5–8]. Previous studies have generally described the regulatory functions of lncRNAs through downstream targets, such as the ceRNA mechanisms and interactions with proteins [9, 10]. Recently, their specific (epi)genetic regulatory mechanisms as well as transcriptional and post-translational regulatory patterns also were widely emphasized and illuminated [11–15]. For instance, the lncRNA linc-RoR, occupied by core transcription factors (TFs), has been found to regulate the efficiency of reprogramming of embryonic stem cell (ESC). It has been confirmed that three human embryonic stem cell (hESC)-crucial lncRNAs (lncRNA-ES1, lncRNA-ES2, and lncRNA-ES3) regulate the expression of pluripotency-related genes [16]. The lncRNAs AK028326 (activated by OCT4) and AK141205 (repressed by NANOG) have been described as regulatory factors for controlling ESC fate, and dysregulation in their function causes a complex interplay between the protein and lncRNA that determines the state of pluripotency [17]. The TF p53 specifically mediates lncPRESS1 to regulate pluripotent gene expression, and lncPRESS1 can indirectly safeguard the hESC state by interacting with the protein SIRT6 [18–20]. Using knockdown, Lin *et al*. defined that TP63 and SOX2 regulate the lncRNA CCAT1 by co-occupying in its distal regulatory elements (super-enhancers, SEs), thereby promoting squamous cancer progression [21]. Moreover, some researches have demonstrated that the single-nucleotide polymorphisms (SNPs) and DNA methylations occurring in the regulatory regions of lncRNAs lead to varying degrees of influence on disease development [22, 23]. Briefly, these intensive efforts used biology experiments to demonstrate the indispensability of multi-omic regulatory elements in functional studies on lncRNAs and to exhibit the tremendous complexity of transcriptional regulation, making it deeper to comprehend lncRNA functional mechanism in various biological processes. Nevertheless, because such biological experiments are extremely resource intensive, only a small fraction of the functional and biological roles of lncRNAs could be clearly defined. Given these observations, it has become an urgent need to utilize the advantage of algorithms with minimum resource consumption for functional lncRNA identification and investigate their transcriptional regulatory mechanism.

Many computational approaches have been developed to investigate lncRNAs, and most of them have focused on predicting their associations with complex diseases, such as LRLSLDA [24], SIMCLDA [25], LDAP [26], MFLDA [27], and LDAPred [28]. Although much has been done on how lncRNAs modulate downstream targets, very little is presented in terms of information about upstream (epi)genetic regulations. The biological complexity and cell specificity of lncRNA transcriptional regulation have not been fully considered by most of the existing methods. Sun *et al*. first integrated TF–lncRNA, miRNA–lncRNA, and lncRNA–PCG interactions into a cell-specific biological network, and implemented the FIS scoring system to accurately recover functional lncRNAs of the mouse skeletal muscle cells. Their results highlighted the necessity of these specific regulatory elements for functional lncRNA identification and further introduced a novel idea for the relevant algorithms [29]. Notably, lncRNAs are regulated by a variety of regulatory elements beyond the mentioned TF–lncRNA, miRNA–lncRNA, and lncRNA–PCG relationships. These elements, such as histone modification, typical enhancers (TEs), SEs, and SNPs, also play crucial roles in the transcriptional regulation of lncRNAs, and their integration is essential for gaining comprehensive insights into lncRNA regulation and function [17, 19–21]. Therefore, there is an urgent need to integrate the (epi)genetic and post-transcriptional regulatory data and construct lncRNA-specific multi-omic biological networks for more comprehensive identification of functional lncRNAs. Despite the diversity of cell types and experimental conditions that make data integration challenging, we are convinced that such arduous efforts can drive research on the functions of lncRNAs, and provide more reliable and comprehensive conditions for analyzing and predicting functional lncRNAs.

Here, we develop a computational framework based on machine learning, FunlncModel, to improve predictions of functional lncRNAs by integrating a large number of (epi)genetic features and functional genomic features from their upstream/downstream multi-omic regulatory networks. Based on the multi-omic networks, we further mined three categories of features as input to the FunlncModel that are likely to influence the critical regulatory roles of lncRNAs, surpassing existing methods in terms of quantity and biological significance. Finally, the random forest learning algorithm was utilized to implement the classification models (the “HESC” model and “Combiner” model) for predicting functional lncRNAs in diverse cellular contexts. Moreover, we determine the optimal set of functional lncRNAs based on random permutations as high-confidence functional lncRNAs and further perform a thorough investigation of their regulatory mechanisms.

## Method

### Constructing (epi)genetic regulatory network of lncRNAs

FunlncModel predicted functional lncRNAs based on random forest machine learning model training on large-scale multi-omic features generated from comprehensive lncRNA upstream/downstream regulatory network. To construct the FunlncModel model, we collected multiple types of (epi)genetic information, including TFs, histone modifications, TEs, SEs, chromatin accessibility regions, SNPs, and methylation sites. We used this information to construct the upstream (epi)genetic regulatory network of lncRNAs by integrating multiple biosamples and regulatory relationships of the specific cellular contexts (Fig. S1 green boxes, Table S2, Supplementary Note 1).

### Establishing post-transcriptional regulatory network

**Figure 1:**
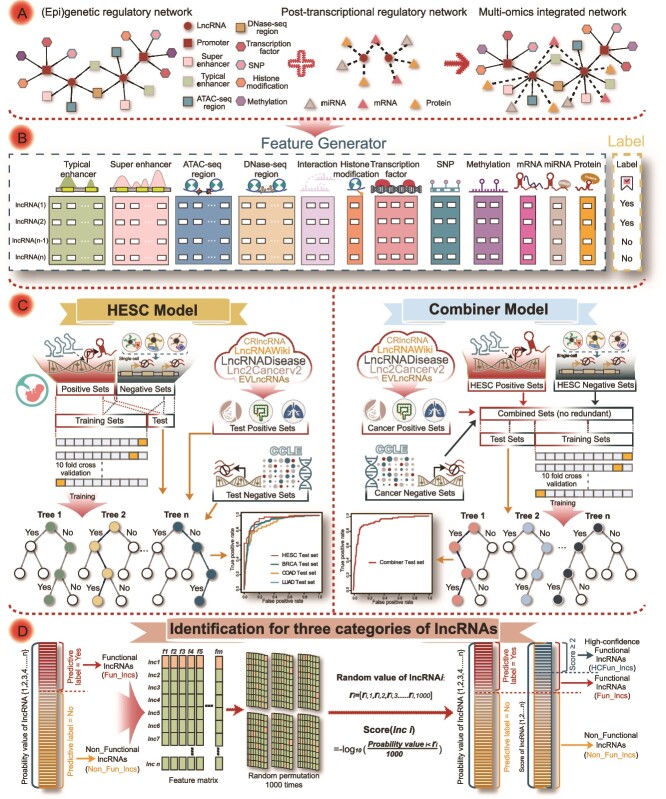
The overall workflow of the construction of model and the identification of functional lncRNAs. The pipeline for HCFun_lncs identification involved four steps. (A) The multi-omic network was established by integrating (epi)genetic and post-transcriptional regulatory networks. (B) Genomic features generated by the multi-omic network for model construction. (C) Flowchart for the HESC and combiner model construction based on different training datasets. (D) Fun_lncs and Non_Fun_lncs were further divided by prediction labels of the abovementioned classifier, where the labels were generated based on probabilities and a threshold (usually 0.5). Next, high-confidence functional lncRNAs (HCFun_lncs) were distinguished from typical functional lncRNAs (Fun_lncs) using the random permutation score.

The lncRNA-target genes were collected from LncRNA2Target v2.0 [30], and genes undergoing significant changes in expression after being knocked down or overexpressing a lncRNA were considered to be the targets of the given lncRNA. The associated miRNAs were obtained from starBase v2.0 [31] and LncACTdb 2.0 [32]. In addition, we collected the lncRNA–protein interactions from starBase v2.0 [31] and EuRBPDB [33] (Fig. 1A middle panel, Supplementary Note 1).

### Network integration and construction of the FunlncModel model

Network integration. To capture more comprehensive regulatory relationships and ensure the connectivity of the network, we collected 3D chromatin interactions (e.g. ChIA-PET 3C, 4C, 5C, and Hi-C) from 4DGenome [34], Oncobase [35], 3D Genome Browser [36], and NCBI [37] (Fig. 1A and Table S1). After incorporating the relationships of 3D chromatin interactions into the (epi)genetic regulatory network mentioned above, we further combined the post-transcriptional regulatory networks and the (epi)genetic regulatory network to create a comprehensive multi-omic network of lncRNAs in specific cellular contexts. The final extensive multi-omic regulatory networks were composed by 12 182 lncRNAs and their associated >10 types of regulatory factors, which provided a comprehensive view for the regulatory landscape of lncRNAs. Specifically, the hESC regulatory network contained 17 012 441 edges; the breast cancer regulatory network encompassed 6 795 331 edges; the colon cancer regulatory network contained 4 693 192 edges; the lung cancer regulatory network included 9 576 710 edges. These regulatory edges involved chromatin interactions, distance, targeted regulation, and expression correlation, capturing the complexity across diverse biological contexts.

Generating multi-omic feature sets. According to the regulatory specificities of the lncRNAs and the topological properties of the network, we mined 57 features for unraveling the complex mechanisms underlying lncRNA-mediated regulation, including outdegree and indegree in the network, and the signal strength and rank of the neighbor nodes (Fig. 1B). These features could also be categorized into three major types based on the lncRNA-specific transcriptional regulation mechanisms, including the upstream proximal, the upstream distal, and the downstream regulation (Supplementary Note 2, Table 1, Table S1; see Results). For instance, the number of lncRNA-associated TFs was calculated as an upstream proximal feature, as follows:


(1) \begin{equation*} T{F_{ChIP}}_{num,j}=\sum_{n=1}^N{TF}_{n,j} \end{equation*}

**Table 1:** The feature descriptions

Feature category	Subcategory	Feature	Number	Description
Feature C1	Transcription factor	${f}_3$ , ${f}_4$, ${f}_5$	3	The amount of TFs (ChIP-seq and motif)
Feature C1	Histone modification	${f}_8$	1	The type of transcriptional activation histone modifications
Feature C1	Methylation	${f}_6$ , ${f}_7$	2	Methylation sit count and normalized signal strength
Feature C1	SNP	${f}_1$ , ${f}_2$	2	RiskSNP and commonSNP sit
Feature C2	Super-enhancer	${f}_{12-15}$ , ${f}_{26}$, ${f}_{31-33}$, ${f}_{41}$, ${f}_{46-48}$	12	SE-related features, including the ChIP-seq signal value and normalized rank (manner: ROSE and chromatin interaction)
Feature C2	Enhancer	${f}_{16-22}$ , ${f}_{25}$, ${f}_{27-30}$, ${f}_{34-36}$, ${f}_{40}$, ${f}_{43-45}$, ${f}_{49-51}$	23	TE-related features, including the normalized signal value and rank (manner: ROSE and chromatin interaction)
Feature C2	Chromatin accessibility	${f}_{9-11}$ , ${f}_{23}$, ${f}_{37-39}$, ${f}_{52-54}$	10	The quantity of chromatin accessibility regions (manner: ROSE and chromatin interaction)
Feature C2	3D chromatin interaction	${f}_{24}$	1	The 3D chromatin interaction frequency
Feature C3	mRNA, miRNA	${f}_{55}$ , ${f}_{56}$	2	The associated mRNA and miRNA amount
Feature C3	Protein	${f}_{57}$	1	The number of associated proteins

According to the specific transcriptional regulatory mechanism of the lncRNAs, we mined 57 features from their upstream/downstream transcriptional regulatory networks and grouped them into three major types: (i) Feature C1, those associated with the upstream proximal regulation of lncRNAs; (ii) Feature C2, those associated with the upstream distant regulation of lncRNAs; and (iii) Feature C3, those associated with the downstream regulation of lncRNAs. For example,${f}_3$ represents the amount of TFs, whose ChIP-seq peak overlapped with the lncRNA promoter region ($TF\_{ChIP}_{num}$); ${f}_{18}$ represents the normalized signal value of enhancers associated with lncRNAs, their regulatory relationships were identified by ROSE python script ($Dis\_{TE}_{signal}$); ${f}_7$ represents the normalized methylation signal strength of lncRNA promoter region ($MS$) (see Table S1; Supplementary Note 1).

where $TF\_{ChIP}_{num,j}$ represents the number of TFs associated with ${lncRNA}_j$, and the regulatory relationship between the $n$th TF and the $j$th lncRNA is denoted by ${TF}_{n,j}\in \left\{1,0\right\}$.

The HESC model establishment and application. To screen functional lncRNAs in hESCs, we first collected multi-omic data of hESC-associated samples to generate the lncRNA upstream/downstream transcriptional regulatory networks. Then, we captured three categories of features from the multi-omic networks by their hESC-specific transcriptional regulatory mechanisms for the HESC model construction (Table 1, Table S1, Supplementary Note 1–2). The collection of positive and negative training datasets was critical for the accuracy of the predictive model. We first collected 326 iPSC-related lncRNAs with functions that impact cell growth, as screened by Liu *et al*. using the genome-scale CRISPRi technique [38]. After alignment and filtering, a total of 238 lncRNAs were included in the positive dataset (Table S13). Since the absence of experimental evidence for nonfunctional lncRNAs, as well as the fact that specific expression and activities were hallmarks of functional lncRNAs, the unexpressed lncRNAs were presumed to be incapable of functioning in this specific state [29, 39, 40]. Therefore, we further analyzed single-cell RNA-Seq data from Li *et al*. [41], which provided valuable insights into the expression patterns of lncRNAs in hESCs and late blastocyst cells. A total of 152 unexpressed lncRNAs in hESCs (Fragments Per Kilobase per Million = 0) but expressed in late blastocyst cells were defined as the negative dataset (Table S13). This strategy to choose the negative dataset helped avoid the scenario of non-expression caused by errors in the sequencing technology. By default, 80% of the input positive and negative datasets were randomly extracted for model training, while the remaining 20% were reserved for testing in order to assess overall performance. Given that the overlap of lncRNAs with similarly regulated between the training set and the test set, the proposed prediction tasks may not adequately measure the model’s generalization power. We thus added the experiments involving data segmentation based on lncRNA sequence similarity. Specifically, we obtained lncRNA sequence from LNCipedia [42] and employed MMseqs2 [43] to cluster lncRNAs based on lncRNA sequence similarity. We then assigned these clusters to either the training set or the test set, thus maintaining an 80/20% split. This approach ensures that lncRNAs within the same cluster (likely to be similarly regulated) are not split across the training and test datasets. We further standardized and transformed the training dataset, screened a subset of predictors that could be used to produce an accurate model, and finally trained the random forest model by using the open-source R package caret (Fig. 1C left panel, Supplementary Note 3).

Furthermore, we processed the relevant datasets of the other three cancer types (breast, colon, and lung cancer) to generate the lncRNA multi-omic regulatory networks and construct feature matrix in the corresponding cellular contexts as input of HESC model, respectively. To evaluate the HESC model’s capability for generalization, we further collected cancer-related lncRNAs (Table S13), functional lncRNAs involved in the growth of cancer cells from Lnc2Cancer v2.0 [44], LncRNADisease v2.0 [45], LncRNAWiki [46], CRlncRNA [47], EVLncRNAs [48], and LncRNADisease [49], as well as functional lncRNAs involved in the seven cancer hallmarks (apoptosis, invasion, metastasis, migration, prognosis, epithelial mesenchymal transition, and proliferation) from CRlncRNA [47]. These lncRNAs with given functional labels were utilized to report the classification performance of the HESC model.

Random Permutation Score for identifying high-confidence functional lncRNAs (HCFun_lncs). We preliminarily identified candidate functional lncRNAs (Fun_lncs) by using the probabilities predicted by the model. To further sort these Fun_lncs according to priority, we proposed the random permutation strategy (Fig. 1D). Let the feature matrix be $$F=\left[\begin{array}{ccc}{f}_{1,1}& \begin{array}{cc}{f}_{1,2}& \dots \end{array}& {f}_{1,m}\\{}\begin{array}{c}{f}_{2,1}\\{}\vdots \end{array}& \begin{array}{cc}\begin{array}{c}{f}_{2,2}\\{}\vdots \end{array}& \dots \end{array}& \begin{array}{c}{f}_{2,m}\\{}\vdots \end{array}\\{}{f}_{n,1}& \begin{array}{cc}{f}_{n,2}& \dots \end{array}& {f}_{n,m}\end{array}\right]$$, where ${f}_{i,j}$ represents the value of feature $j$ of the lncRNA $i$, $n$ is the number of lncRNAs, and $m$ is the number of features. We first obtained the probabilities of all unknown functional lncRNAs from the optimal hESC model and then randomly permuted the feature matrix of these lncRNAs for 1000 times. The random matrix $k$ could be written as $$\left[\begin{array}{c}{L}_1\\{}\begin{array}{c}{L}_2\\{}\vdots \end{array}\\{}{L}_n\end{array}\right]=\left[\begin{array}{ccc}{f}_{2,1}& \begin{array}{cc}{f}_{3,2}& \dots \end{array}& {f}_{1,m}\\{}\begin{array}{c}{f}_{n,1}\\{}\vdots \end{array}& \begin{array}{cc}\begin{array}{c}{f}_{2,2}\\{}\vdots \end{array}& \dots \end{array}& \begin{array}{c}{f}_{4,m}\\{}\vdots \end{array}\\{}{f}_{1,1}& \begin{array}{cc}{f}_{4,2}& \dots \end{array}& {f}_{n,m}\end{array}\right]$$. For lncRNA $i$, we obtained the vector of random probability values ${r}_{\mathrm{i}}=\left[{\mathrm{r}}_{\mathrm{i},1},{r}_{\mathrm{i},2},{r}_{\mathrm{i},3}\cdots{r}_{i,k}\cdots{r}_{i,1000}\right]$, where ${r}_{i,k}$ represents the probability of the lncRNA $i$ as calculated from the random matrix $k$. Using these random values, we calculated the score of each lncRNA. Finally, lncRNAs with a score >2 were considered to be HCFun_lncs, as follows:


(2) \begin{equation*} {\displaystyle \begin{array}{c}\mathrm{Score}\left(\mathrm{lnc}\ i\right)=-{\log}_{10}\displaystyle\frac{\sum_{k=1}^{1000}I\left({\mathrm{Probability}\ \mathrm{value}}_i<{\mathrm{r}}_{i,k}\right)\ }{1000}\\{}\end{array}} \end{equation*}

The Random Permutation Score was designed to further capture high-confidence functional lncRNAs with high prediction probability values, effectively capturing the most reliable candidates from a broader set of predicted functional lncRNAs. According to the Random Permutation Score, HCFun_lncs with high prediction probability values and confidence levels were distinguished from typical functional lncRNAs.

## Results

### Performance evaluation of the HESC model of FunlncModel

**Figure 2:**
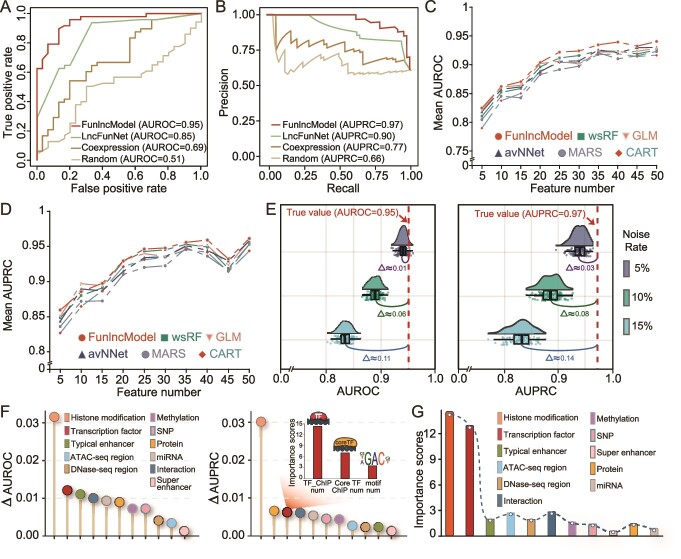
Evaluation of the effectiveness of the HESC model of FunlncModel based on functional lncRNAs. (A–B) The ROC and PRC curves of FunlncModel, the baseline control test (random permutation of known labels), and other two existing approaches to identify functional lncRNAs. (C–D) The line chart showed AUROC and AUPRC performance indicators of multiple machine learning methods, where the models were constructed based on 10 different random selections of *N* features (where *N* = 5, 10, …, max_num_features). (E) The changes in classification performance after the addition of noise. (F) A scatter diagram representing changes in the accuracy of the model when a certain type of feature was removed. (G) The importance scores of features generated based on MDG values of random forest.

Using these multi-omic regulatory features from the lncRNA upstream/downstream regulatory network and the RF machine learning algorithm, we trained and constructed the HESC model of FunlncModel to prioritize functional lncRNAs in HESC (see Methods, Supplementary Note 1–3). One-fifth of all positive and negative sets were considered by default as the independent test set for the HESC model to evaluate model performance and 10-fold cross-validation was used on the remaining sets to determine the optimal parameters of the model. FunlncModel achieved AUROC of 0.95 and AUPRC of 0.97 on the independent test sets, thus delivering excellent classification performance of our model for uncovering known Fun_lncs (Fig. 2A–B, Table 2, Fig. S2A, Supplementary Note 5). To further test the robustness of FunlncModel against sequence similarity biases, we also added data segmentation experiments based on lncRNA sequence similarity (as detailed in the Methods section). As shown in Table S10 and Fig. S8A–B, FunlncModel retains strong predictive power, demonstrating high accuracy and robustness even with reduced sequence similarity between training and test sets. Next, we compared FunlncModel with the existing functional lncRNA identification approaches including LncFunNet and co-expression (see Supplementary Note 11) [29, 50]. It was apparent that FunlncModel outperformed these approaches in terms of sensitivity and specificity, achieving a 10% higher AUROC and 7% higher AUPRC than LncFunNet, as well as a 26% higher AUROC and 20% higher AUPRC than co-expression (Fig. 2A–B). The baseline control test (random permutation of known labels) further verified the error-free calculation of the proposed model (Fig. 2A–B). We also developed models based on all selected features, 10 different random selections of *N* features (where *N* = 5, 10, …, max_num_features), and employing various popular supervised learning approaches, including Generalized Linear Model (GLM), Model Averaged Neural Network (avNNet), Multivariate Adaptive Regression Spline [37], weighted Subspace Random Forest (wsRF), and CART (Fig. S9B, Fig. 2C–D). As shown in Fig. 2C–D, the accuracy of all methods improved as the number of features increased, which provided a more comprehensive insight into the impact of feature variability on model accuracy. Notably, the RF approach significantly outperformed the other approaches when number of features was >5, indicating its superior suitability for predicting functional lncRNAs in terms of both classification accuracy and model interpretability.

**Table 2:** The performance values of HESC and combiner model in 10-fold cross-validation experiments

Performance values		HESC model					Combined model
		Validation set	Remaining test set	LUAD test set	BRCA test set	COAD test set	Remaining test set
AUROC	Fold1	**0.996**	**0.95**	**0.83**	**0.92**	**0.89**	0.95
	Fold2	0.87	0.93	0.80	0.90	0.85	0.97
	Fold3	0.86	0.94	0.81	0.92	0.88	0.96
	Fold4	0.77	0.93	0.80	0.91	0.87	0.95
	Fold5	0.87	0.94	0.82	0.92	0.87	0.96
	Fold6	0.79	0.93	0.81	0.91	0.87	0.96
	Fold7	0.87	0.93	0.82	0.91	0.88	0.96
	Fold8	0.88	0.93	0.81	0.91	0.87	0.96
	Fold9	0.92	0.94	0.83	0.91	0.89	0.96
	Fold10	0.93	0.93	0.73	0.85	0.77	0.96
AUPRC	Fold1	**0.997**	**0.97**	**0.53**	**0.70**	**0.64**	0.93
	Fold2	0.93	0.96	0.51	0.62	0.56	**0.94**
	Fold3	0.92	0.96	0.47	0.69	0.61	0.93
	Fold4	0.75	0.96	0.49	0.65	0.70	0.92
	Fold5	0.87	0.96	0.51	0.69	0.64	0.93
	Fold6	0.81	0.96	0.55	0.63	0.68	0.93
	Fold7	0.93	0.96	0.49	0.68	0.60	0.93
	Fold8	0.92	0.96	0.48	0.68	0.58	0.93
	Fold9	0.95	0.96	0.50	0.69	0.62	0.93
	Fold10	0.96	0.95	0.37	0.50	0.38	0.92

To evaluate the robustness of FunlncModel to noise, we randomly added noise at rates of 5%, 10%, and 15% to the train and test sets, and conducted 100 iterations of random noise analysis. As shown in Fig. 2E, there was only a slight decline in accuracy (mean AUROC/PRC of train sets: 0.92/0.95, 0.89/0.92, and 0.81/0.87; mean AUROC/PRC of test sets: 0.94/0.94, 0.89/0.89, and 0.84/0.83), thus revealing the insensitivity of our model to noise. We also observed a drop in accuracy as the negative data were replaced (Fig. S2B).

Among the results, some known functional lncRNAs were successfully predicted. For instance, GAS5 (as a Fun_lnc with a probability of 0.814) has been reported to promote and control hESC self-renewal [51]. Meanwhile, we identified a known functional lncRNA ESRG (a known ESC-related lncRNA) as Fun_lnc [52]. Another functional lncRNA, NEAT1, was also identified as Fun_lnc (with a probability of 0.858), which was confirmed as a protein-binding scaffold to regulate the fates of Bone marrow mesenchymal stromal cells (BMSCs) by maintaining pluripotency [53]. Additionally, we found that FunlncModel successfully identified most of the known lncRNAs from Ref [54] (Table S15), which influence the states of stem cells. We also displayed which lncRNAs were annotated by LNCipedia [42]. Taken together, these results demonstrated that FunlncModel, as a reliability prediction model, has the powerful ability to recover well-studied hESC-specific functional lncRNAs.

To verify whether the integration of multi-omic features was necessary, we first quantified their contributions (odds ratio and relative risk) for FunlncModel classification outcome (Fig. S2D–E, Table 1). The TEs, histone modifications, and TFs made more prominent contributions to the model among these features, which was consistent with their transcriptional activation-related properties. Correspondingly, the Fun_lncs did show significantly higher than Non_Fun_lncs on most of the features, especially those related to transcriptional activation, implying their stronger transcriptional activities (Fig. S2F, Table 1; two-sided Wilcoxon rank-sum test). We further collected files of the H3K4me3 and H3K27ac signals from ENCODE [55], and utilized deepTools to obtain a high-resolution view of their transcriptional landscape [56] (Fig. S2G). Higher transcriptional activities were observed in the promoter regions of the Fun_lnc group, hinting at its potential to perform important functions. In addition, its correctness was once again demonstrated by the significant differences in feature values between the positive and negative groups in the training sets (Fig. S2H; two-sided Wilcoxon rank-sum test).

We further measured the changes in precision and recall by removing each category of features and found that the absence of any category resulted in a decrease in the classification accuracy (Fig. 2F, Table 1, Supplementary Note 2). Meanwhile, these features with more obvious changes tended to generate higher importance scores of the RF-based approach (Fig. 2G and Fig. S2C, Table 1; see Supplementary Note 3). For instance, the histone modification feature, as one of the top-ranked features in terms of importance score, demonstrated the most significant impact on classification accuracy. This impact may be attributed to their irreplaceable regulatory roles in maintaining the pluripotency of ESCs and determining cell fate [57, 58]. The TF-related features reflected a similar trend; especially scores of TF-related features identified by ChIP-seq data (TF_ChIPnum and Core_TF_ChIPnum) were higher than those of the TF-related motif features, where this was consistent with the advantage of the ChIP-seq in identifying TF targets over motif-based strategy. These results suggested that the upstream/downstream multi-omic features played indispensable roles and as strong predictors for identifying functional lncRNAs.

### High-confidence functional lncRNAs performed greater capabilities of transcriptional regulation

**Figure 3:**
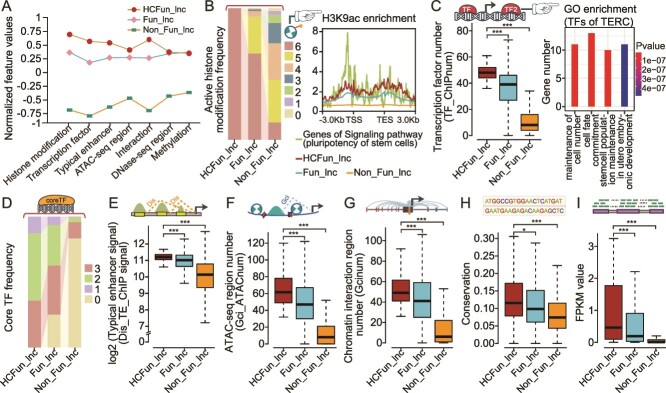
Analysis of the high-confidence functional lncRNAs in HESC model. (A) The line chart exhibited the differences in the mean values of the features of HCFun_lncs, Fun_lncs, and other lncRNAs for these features with importance scores >2, where the mean values have been standardized. (B) The histogram of the distributions of frequencies of three categories of lncRNAs with different amounts of histone modification–based enrichment. The right side visualized the enrichment of H3K9ac in the promoter region of the three categories of lncRNAs and the genes of the hsa04550 pathway (the signaling pathway that regulates the pluripotency of stem cells). (C) The number of TFs occupied in promoter regions of three categories of lncRNAs, as well as GO terms enrichment analysis result of TERC-associated TFs. (D) Histogram displays the distribution of the three categories of lncRNAs occupied by hESC-related core TFs. (E) The normalized ChIP-seq signal values of TEs of regulating three categories of lncRNAs, where their regulatory relationships were identified by ROSE. (F) The number of ATAC-seq regions regulating three categories of lncRNAs, where their regulatory relationships were identified by chromatin 3D interaction strategy. (G) The conservation scores of three categories of lncRNAs. (H) The expression levels of three categories of lncRNAs in hESC samples. ^*^P < .05; ^**^P < .001; ^***^P < .0001; two-sided Wilcoxon rank-sum test.

Machine learning–based predictions are often dense. We thus performed the random permutation strategy to rank and prioritize the Fun_lncs. The details of the permutations are described in the Methods. According to the random permutation test for HESC model, we obtained high-confidence functional lncRNAs with high prediction probability values from the numerous functional lncRNAs (HCFun_lncs; with score >2) (Table S14). As shown in Fig. S9C–D, FunlncModel also exhibited outstanding classification performance utilizing the Random Permutation Score. We investigated whether HCFun_lncs conducted even more specific activities and regulatory capabilities. Indeed, we observed clear differences in the mean values of features among HCFun_lncs, Fun_lncs, and Non_Fun_lncs groups (Fig. 3A and Fig. S3A). Most of the HCFun_lncs were marked by more histone modifications than the other two categories of lncRNAs (Fig. 3B). Among these transcriptional activation histone modifications, H3K9ac had been reported as a key marker for the initiation of ESC pluripotency and associated with gene transcription activation. As shown in Fig. 3B, HCFun_lncs did exhibit a higher H3K9ac signal in their promoter regions, which was consistent with the activity trend of important genes (from signaling pathways regulating pluripotency of stem cells; hsa04550). HCFun_lncs were also regulated by more TFs than the other two categories of lncRNAs, where this conformed to their higher transcriptional activities (Fig. 3C). These TFs of HCFun_lncs were usually significantly enriched in hESC-related GO terms. For instance, TERC, a lncRNA with a high ranking in our HESC model, was regulated by TFs related to maintaining the population of stem cells (Fig. 3C). Upregulation of TERC was a key feature influencing the state of pluripotency of iPS cells, and its regulatory region was occupied by the core TFs, including SOX2, NANOG, and OCT4 [59]. Consistently with this, we found that most of HCFun_lncs had highly enriched core TFs (SOX2, MYC, NANOG, and OCT4), which have been extensively studied and demonstrated to play crucial roles in maintaining pluripotency, regulating gene expression, and modulating signaling pathways [17] (Fig. 3D). The comparison results described that HCFun_lncs were also regulated by more DNA regulatory elements, such as the TEs and accessible chromatin regions (Fig. 3E–G; two-sided Wilcoxon rank-sum test). Furthermore, there are several common criteria for evaluating the importance of lncRNAs, such as sequence conservation and specific expression [60, 61]. As expected, HCFun_lncs did demonstrate higher sequence conservation during biological evolution (see Supplementary Note 8) and higher expression levels than the other categories of lncRNAs (Fig. 3H–I). These results indicated the HCFun_lncs were significantly superior to other lncRNAs in terms of epigenetic modification, sequence conservation, and specific expression, as well as the potential capabilities of TFs, histone modifications, and other (epi)genetic features for function explanations of HCFun_lncs.

**Figure 4:**
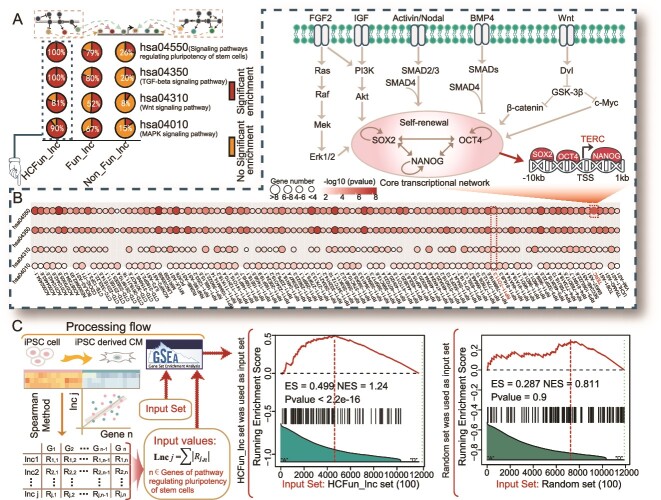
Function analysis of the high-confidence functional lncRNAs. (A) The distribution for the enrichment of important pathways. (B) Detailed information on the enrichment of each high-confidence functional lncRNAs for the four hESC-related pathways, where the colors represent the significance of the enrichment and the sizes represent their number of overlapping genes. (C) Flowchart of processing and the results of analysis of HCFun_lncs based on correlations of expressions with important genes of the hsa04550 pathway (signaling pathway regulating the pluripotency of stem cells), as well as the result of a random control group. Their enrichment scores and normalized enrichment scores have been marked. The right side showed the results of the random control group.

We performed pathway enrichment analysis by using TFs occupying in lncRNA promoter region to further explore their biological functions [62]. HCFun_lncs and Fun_lncs were significantly enriched in several crucial pathways that were extensively studied, including the pluripotency of stem cells (hsa04550), TGF-beta (hsa04350), Wnt (hsa04310), and the MAPK signaling pathway (hsa04010) [63–68]. Wnt signaling pathway (hsa04310), as one of the well-known pathways, was involved in the regulation of stem cell self-renewal and differentiation. Activation of the Wnt pathway promoted self-renewal, while inhibition of this pathway induced differentiation of hESCs [68]. HCFun_lncs produced a more comprehensive distribution of enrichment in the four pathways than Fun_lncs and Non_Fun_lncs, with over 80% of them significantly enriched in these crucial pathways (Fig. 4A–B). For instance, TERC-associated TFs were significantly enriched in the pluripotency of stem cell signaling pathway (hsa04550). We thus drew the detailed regulatory pattern that demonstrated how the signaling pathway directed its terminal TFs (SOX2, OCT4, and NANOG) to regulate and control the TERC by binding to its promoter region, thereby maintaining the pluripotency and self-renewal of hESC (Fig. 4B). Collectively, the HCFun_lncs were typically regulated by more specific functional elements and involved in critical biological processes, which could provide valuable insights into the complex regulatory networks and specific mechanisms underlying the maintenance of the pluripotent state of hESCs.

### Investigations of high-confidence functional lncRNAs in hESC differentiation processes

**Figure 5:**
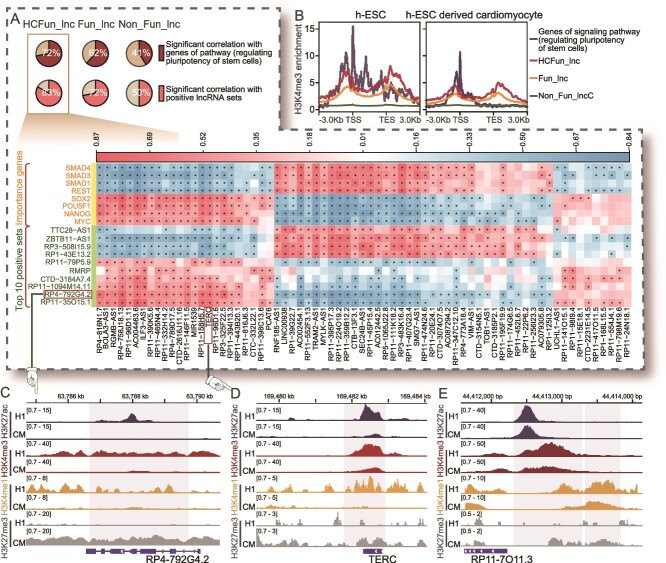
Analysis of high-confidence functional lncRNAs in hESCs and cardiomyocytes. (A) The pie charts show the percentage distribution of lncRNAs that were significantly correlated with at least 10 positive lncRNAs or at least one gene of the hsa04550 pathway in the three categories. The heatmap reflects the correlation of expressions between HCFun_lncs and important genes of the hsa04550 pathway (the signaling pathway that regulates the pluripotency of stem cells) and the top-10 positive lncRNA sets; ^*^P < .05. (B) The enrichment signal of H3K4me3 histone modifications in promoter regions of important genes, and three categories of lncRNAs in the hESC samples and cardiomyocyte samples derived from them. (C–E) Integrative Genomics Viewer (IGV) plots of diverse histone marks in lncRNA promoter regions (RP4-792G4.2, TERC, and RP11-7O11.3) in the hESC samples and cardiomyocyte samples derived from them.

To further dissect the dynamic changes in the transcriptional regulation of HCFun_lncs during processes of differentiation of the hESCs, we calculated coefficients of correlation of expressions between each evaluated lncRNA, and important genes related to the pluripotency and self-renewal of hESCs (from signaling pathways regulating pluripotency of stem cells; hsa04550) (see Supplementary Note 6). The Gene Set Enrichment Analysis (GSEA) analysis results showed that HCFun_lncs exhibited a stronger expression correlation with the important genes than the random control group (Fig. 4C) [69]. Moreover, HCFun_lncs were more strongly correlated with positive lncRNAs (from the positive training sets) (Fig. 5A and Fig. S3B). We found that HCFun_lncs not only exhibited high H3K4me3 activities in their promoter regions, which reflected the transcription of active genes (Fig. 5B), but also showed significant changes in signals among hESC and hESC-derived cardiomyocyte cellular contexts, suggesting the strong dynamic changes in and the ESC-specificity of HCFun_lncs (Fig. 5B). HCFun_lncs possessed much stronger expression correlation with positive lncRNAs/genes (from hsa04550 pathway) among all the evaluated lncRNAs, and more similar regulation patterns and trend of variations in activity to those of important genes during differentiation processes.

Notably, RP4-792G4.2 (FOXD3 antisense RNA 1), a known functional lncRNA, was confirmed to influence iPSCs cell growth rates [38]. The promoter region of RP4-792G4.2 was enriched with higher active signals (H3K27ac, H3K4me3, and H3K4me1) and lower inhibiting signals (H3K27me3) in hESCs compared with cardiomyocyte (see Supplementary Note 10, Fig. 5C). We also observed similar signal enrichment in TERC promoter region, whose functions in hESCs were already described above (Fig. 5D). Furthermore, we found potential novel functional lncRNAs in HCFun_lncs. For example, a novel lncRNA RP11-7O11.3 [42, 70] (approved symbol: LINC02918) was ranked high by FunlncModel. RP11-7O11.3-associated TFs were significantly enriched in critical hESC-associated pathways (Fig. 4B) and showed similar signal enrichment for histone modification to that of the known functional lncRNA RP4-792G4.2 (Fig. 5E, Fig. S3C–D), suggesting the specific transcription activity and significant potential of RP11-7O11.3 as an hESC-related functional lncRNA. Taken together, this shows that FunlncModel not only accurately recovered most known functional lncRNAs, but also contributed to the discovery of potential novel functional lncRNAs in hESCs.

### Evaluating the model’s generalization ability using three cancer sets and seven cancer hallmarks

**Figure 6:**
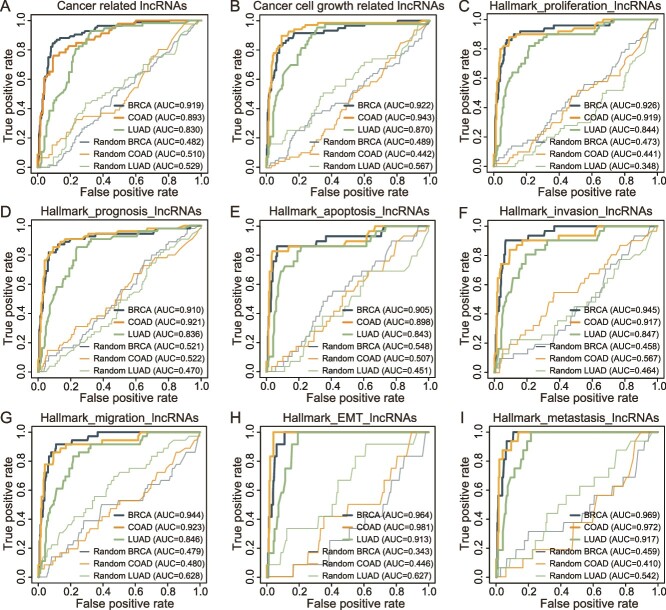
Evaluating the HESC model’s predictive ability on independent cancer test sets. (A) The ROC curves of known cancer-related lncRNA sets and the control group. (B) The ROC curves of lncRNAs related to cancer cell growth and the control group. (C–I) The ROC curves of 7 cancer hallmark lncRNA sets, including apoptosis, invasion, metastasis, migration, prognosis, EMT, and proliferation, and their control group.

FunlncModel exhibited excellent classification performance in terms of predicting and classifying functional lncRNAs in hESCs. Given that many studies have revealed the crucial roles of (epi)genetic elements for lncRNAs in the context of cancer [21–23] and the comprehensive evidence for the existence of cancer-related lncRNAs, we therefore analyzed several cancer samples with well-rounded data, including breast cancer, colon cancer, and lung cancer (see Methods). Cancer-specific feature matrixes were used as independent test sets to objectively evaluate whether the HESC model could be adopted in a diversity of cellular contexts and tasks of function prediction (see Methods). We first calculated the AUROC and AUPRC values on the independent test sets containing data on the three types of cancer, respectively (Fig. 6A, Fig. S10A, Fig. S8C–D, Tables S10–11). Then, we tested the power of FunlncModel to predict known functional lncRNAs involved in the growth of cancer cells and seven hallmarks of cancer (apoptosis, invasion, metastasis, migration, prognosis, EMT, and proliferation) (Fig. 6B–I, Fig. S10B–I). We tested up to 27 cancer-related functional prediction tasks and observed promising predictive performance, as all AUROC values were >0.8 (mean: 0.908; SD: 0.044), which was much higher than that of the control group (random permutation of the labels) (Fig. 6B–I; see Methods, Supplementary Note 5). Compared to established tools for predicting disease-related lncRNAs (CapsNet-LDA [71] and LncDisease [72]), FunlncModel also achieved higher classification accuracy, specifically in terms of cancer and cancer cell growth (Table S11). Moreover, the identified HCFun_lncs exhibited more TFs and histone modification enrichment and were regulated by more DNA functional elements than the other two categories of lncRNAs (Fig. S4A–H; two-sided Wilcoxon rank-sum test). These HCFun_lncs also possessed higher expression levels, suggesting their stronger transcription activities (Fig. S4I; two-sided Wilcoxon rank-sum test) [73]. An analysis of the enrichment of pathways of TFs occupying the promoter region of the lncRNAs [62] showed that the distribution of enrichment of important pathways for a diversity of cancers was more comprehensive in the HCFun_lncs group (Fig. S4J). In summary, the above analyses illustrated that our proposed model could be applied to multiple different types of biological systems and tasks of function prediction, and reliably identify the relevant functional lncRNAs.

### The combiner model of FunlncModel can improve accuracy of identification of functional lncRNAs in diverse cellular contexts

**Figure 7:**
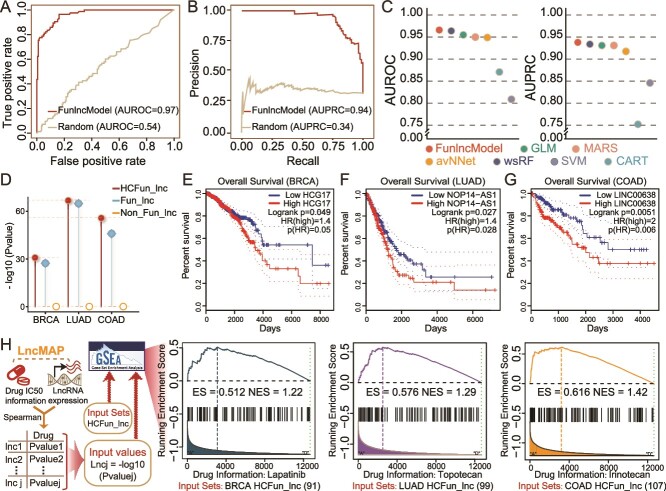
Analysis of the combiner model. (A–B) The ROC and PRC curves of FunlncModel and baseline control group. (C) The values of AUROC and AUPRC of several machine learning methods. (D) The results of hypergeometric enrichment of three-group lncRNA sets with cancer-related lncRNAs of EVLncRNAs2. The *y*-axis represents the −log10 (*P*-values of enrichment analyses). (E–G) The survival outcomes of HCG17, NOP14-AS1, and LINC00638 in BRCA, LUAD, and COAD, respectively. (H) Processing flow chart and the analysis results of HCFun_lncs based on correlations with a treatment drug, where their enrichment scores and normalized enrichment scores have been marked.

As described above, the HESC model demonstrated outstanding performance in cancer cellular contexts but did not incorporate specific cancer-related information. Assuming that the incorporation of cancer information could more effectively capture cancer-specific features and enhance the generalization performance, we thus developed the Combiner model to test this assumption, which aimed to improve the accuracy of identifying cancer-related functional lncRNAs. More cancer-related datasets were incorporated to construct the multi-omic networks for diverse cellular contexts and generate more cancer-specific features. Besides, we add more known cancer-related functional lncRNAs to the training set (see Methods; Supplementary Note 1–4, Table 1, Table S1, and Table S16). As expected, the Combiner model had AUROC/AUPRC of 0.97/0.94, higher than the HESC model in terms of predicting cancer-related functional lncRNAs (Fig. 7A–B, Fig. S5A–B, Table 2). Compared with the other machine learning methods, the RF approach once achieved >1% higher classification accuracy (Fig. 7C). Subsequently, we quantized the importance scores of each feature (Fig. S5C). There were prominent distinctions in the three categories of lncRNAs among different cellular contexts for features with high importance scores, such as TFs, histone modifications, and miRNAs (Fig. S5D–H; two-sided Wilcoxon rank-sum test).

Following the above, we identified functional lncRNAs with high confidences (HCFun_lncs) in breast, colon, and lung cancers. As shown in Figs. S5I and J, HCFun_lncs were involved in more cancer phenotype and disease processes than the other two categories of lncRNAs (see Supplementary Note 9). The HCFun_lncs of each cancer were more significantly enriched in cancer-related lncRNAs from EVLncRNAs2 [74], which was a manually curated database of experimentally validated functional lncRNAs (Fig. 7D, Fig. S6A–C, Table S12). Importantly, these HCFun_lncs were significantly enriched with lncRNAs that influenced the prognoses of cancer patients, implying that many lncRNAs of HCFun_lncs related to the genesis of tumors. For instance, the overexpressions of HCG17, OP14-AS1, and LINC00638 led to poor prognosis of cancer patient survival outcomes, respectively (Fig. 7E–G, Fig. S6D, Table S12; see Methods, Supplementary Note 7). The gain- and loss-of-function assay for LINC00638 confirmed its function in regulating the proliferation, apoptosis, and invasion of non-small cell lung cancer cells [75]. Tang *et al*. demonstrated that inhibiting COX10-AS1 significantly increased both early and late apoptosis rates in cancer cells using cell apoptosis detection [76]. Furthermore, WAC-AS1’s functional role in breast invasive carcinoma was validated through multiple experiments, including qRT-PCR, lncRNA knockdown, CCK-8 assays, and terminal deoxynucleotidyl transferase-mediated dUTP nick-end labeling (TUNEL) staining [77]. These results highlighted precision and reliability of FunlncModel in discovering promising novel lncRNAs.

Moreover, we tested the relationships between HCFun_lncs and cancer-related drugs (lapatinib, topotecan, and irinotecan). The GSEA analysis showed that HCFun_lncs were significantly enriched in drug-related lncRNAs (Fig. 7H, *P*-value <2.2e−16; see Methods, Supplementary Note 7). We found that a total of 99 lncRNAs appeared in at least two cancer types, and ~74% of them were confirmed as known cancer-related lncRNAs, such as MAGI1-IT1, PTOV1-AS1, and DLEU2 (Fig. S7A) [74]. For the remaining 26 HCFun_lncs, survival analysis result also revealed that their expressions were significantly associated with survival (Fig. S7B; log-rank test *P*-value = .0015). Most of these lncRNAs were significantly correlated with survival in the case of at least one cancer type (Fig. S7C) [78]. For instance, the known cancer-related lncRNA THAP7-AS1 [79], WAC-AS1 [80], PRKAG2-AS1 [81], and SRRM2-AS1 [82] were significantly correlated with the patient’s survival outcomes in LIHC, PRAD, and BRCA, respectively (Fig. S7D–G).

## Discussion

LncRNAs are critical to biological processes and diseases. Nevertheless, only a few lncRNAs have been characterized, and the functions of the vast majority remain unknown. Discriminating functional lncRNAs from thousands of candidates of multiple biological processes is still urgently required. Existing algorithms for lncRNAs tend to reveal their functions in complex diseases, such as LRLSLDA [24], SIMCLDA [25], LDAP [26], MFLDA [27], and LDAPred [28]. Although the relevant research has taken advantage of the strengths of computational methods to integrate similarities, expressions, interaction relationships, and information on topological structures into models, the tremendous biological complexity of lncRNAs, such as their distinctive (epi)genetic mechanisms and transcriptional regulatory patterns in specific cells, has not been considered. In light of this, we present FunlncModel, an algorithm to single out specific and common functional lncRNAs in diverse cellular contexts by integrating multiple (epi)genetic features and functional genomic features from their upstream/downstream multi-omic regulatory networks. Although integrating comprehensive (epi)genetic and regulatory data is a challenging task, these (epi)genetic and regulatory features carried out an excellent performance in model prediction, highlighting their great potential and suggesting the challenge task deserves study in depth. In summary, we have provided a framework with high predictive power that can be used in a variety of cellular contexts to shed in-depth light on the biological mechanisms for Fun_lncs, thereby guiding functional experiments.

**Key Points**

- We present a computational framework based on machine learning, FunlncModel, to improve predictions of functional lncRNAs by integrating a large number of (epi)genetic features and functional genomic features from their upstream/downstream multi-omic regulatory networks.
- We mine nearly 60 features in three categories from >2000 datasets across 11 data types, including transcription factors, histone modifications, typical enhancers, super-enhancers, methylation sites, and mRNAs.
- We apply FunlncModel on the hESC dataset and up to 27 cancer-related prediction tasks, achieving excellent classification performance.

## Supplementary Material

Supplemental_Figure_bbae623

Supplementary_Table1_bbae623

Supplementary_Table2_bbae623

Supplementary_Table3_bbae623

Supplementary_Table4_bbae623

Supplementary_Table5_bbae623

Supplementary_Table6_bbae623

Supplementary_Table7_bbae623

Supplementary_Table8_bbae623

Supplementary_Table9_bbae623

Supplementary_Table10_bbae623

Supplementary_Table11_bbae623

Supplementary_Table12_bbae623

Supplementary_Table13_bbae623

Supplementary_Table14_bbae623

Supplementary_Table15_bbae623

Supplementary_Table16_bbae623

Supplementary_MATERIALS_AND_METHODS_bbae623

## Code availability

The FunlncModel online server is freely available at https://bio.liclab.net/FunlncModel/. Downloadable datasets and source code can be found at the FunlncModel website or https://github.com/chunquanlipathway/FunlncModel.
