# Supplementary material for: FunlncModel: integrating multi-omic features from upstream and downstream regulatory networks into a machine learning framework to identify functional lncRNAs
Source: Brief Bioinform. 2024 Nov 27;26(1):bbae623. doi: 10.1093/bib/bbae623 (PMC11601888; doi:10.1093/bib/bbae623)
Supplement: Supplemental_Figure_bbae623 [file supplemental_figure_bbae623.pdf]

Supplementary Figure Legends

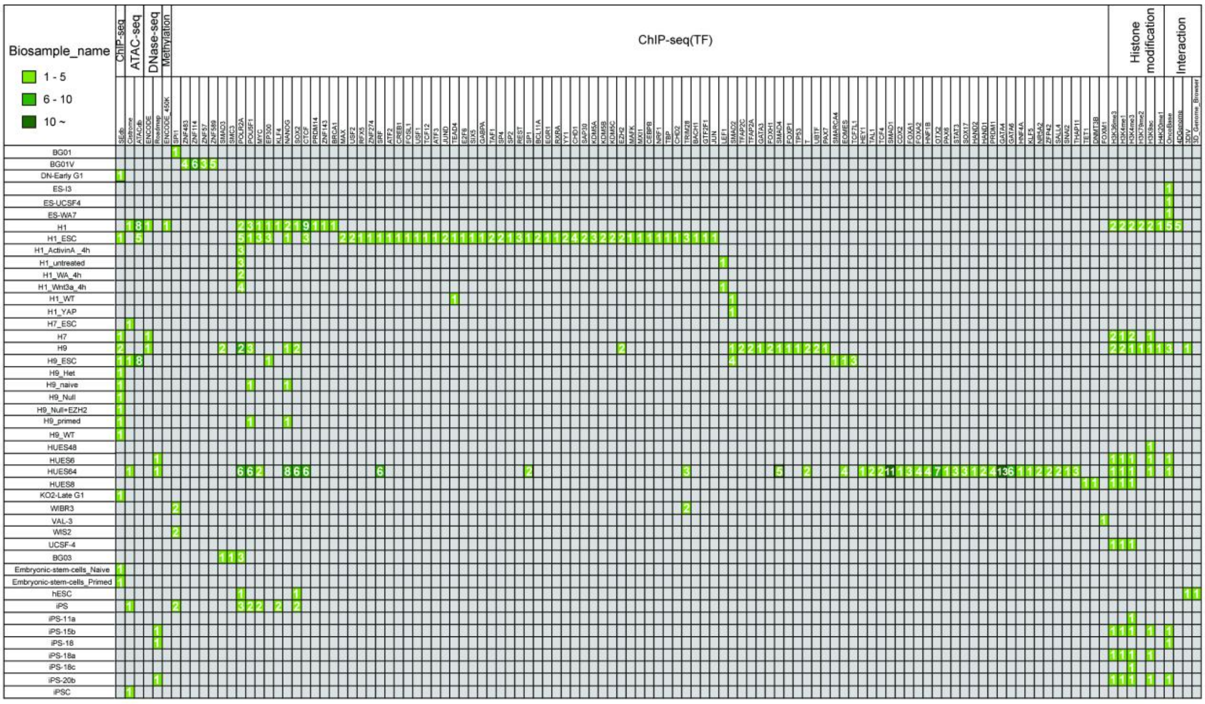

Supplementary Figure 1. The biosamples information of HESC cellular context. The row represents human embryonic stem cell associated sample name. The column represents data type. The gradient color represents the corresponding number of samples.

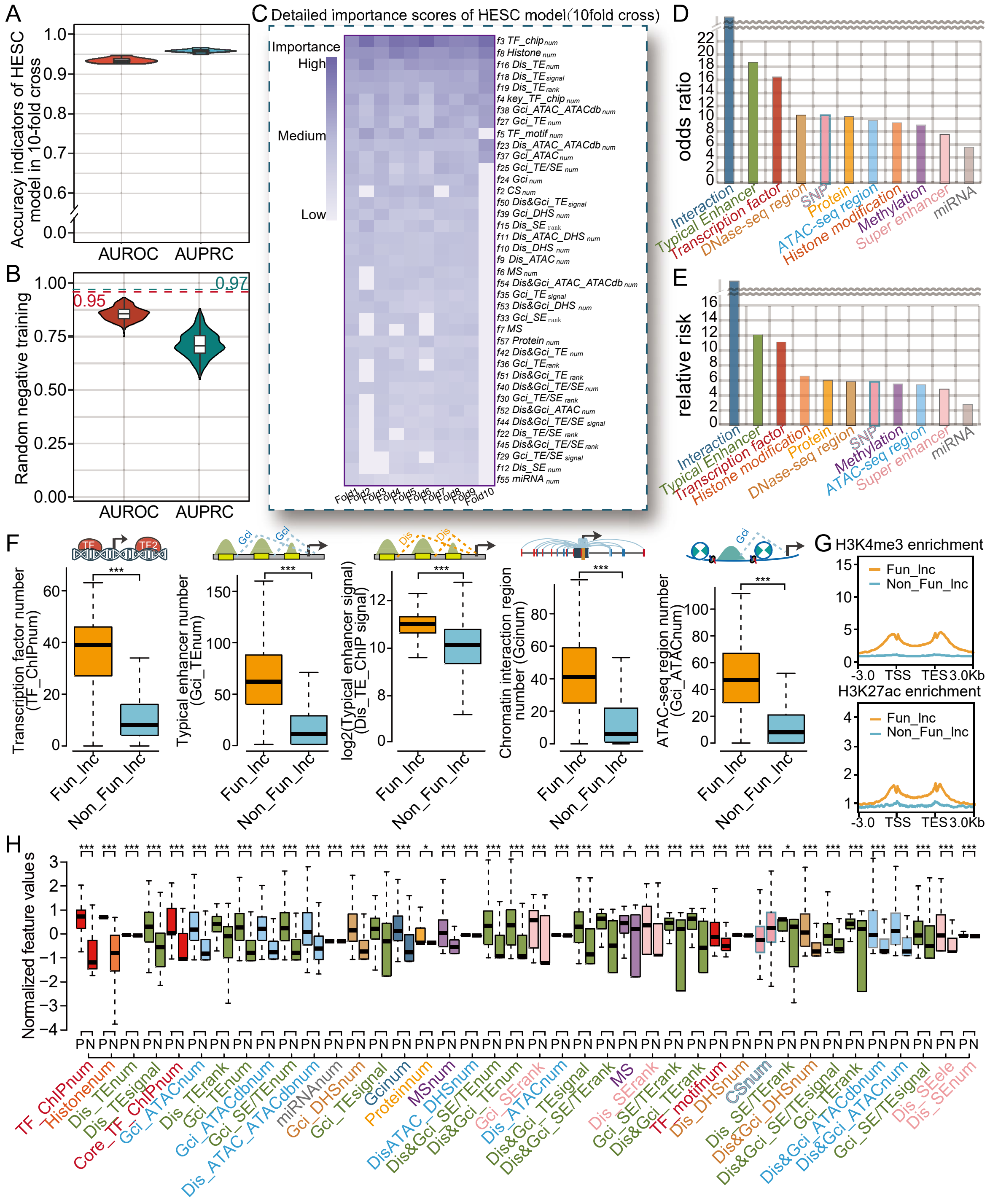

Supplementary Figure 2. Evaluation and analysis for HESC model of FunlncModel. (A) Box plot showed AUROC and AUPRC performance indicators obtained from 10-fold cross validation experiment of HESC model. (B) The accuracy of models constructed by random negative training sets. (C) The heatmap showed importance scores of each feature generated from MDG values of random forest in 10-fold cross validation experiment of HESC model. (D) The odds ratios of features of each class for model classification outcome. (E) The relative risks of features of each class for model classification outcome. (F) Box plot showed two categories lncRNAs distribution of occuping by TFs. The TEs number of regulating two categories lncRNAs, where their regulatory relationships were identified by Chromatin 3D interaction analysis. The normalized ChIP-seq signal values of TEs, where regulatory relationships between TEs and two categories lncRNAs were identified by ROSE. The number of regions interacted with two categories lncRNAs identified by Chromatin 3D interaction analysis. The number of ATAC-seq regions of regulating two categories lncRNAs, where their regulatory relationships were identified by Chromatin 3D interaction analysis. (G) The H3K4me3 and H3K27ac ChIP-seq signals enrichment in promoter regions of two categories lncRNAs. (H) Box plots showed distinction between positive lncRNAs and negative lncRNAs for these features.

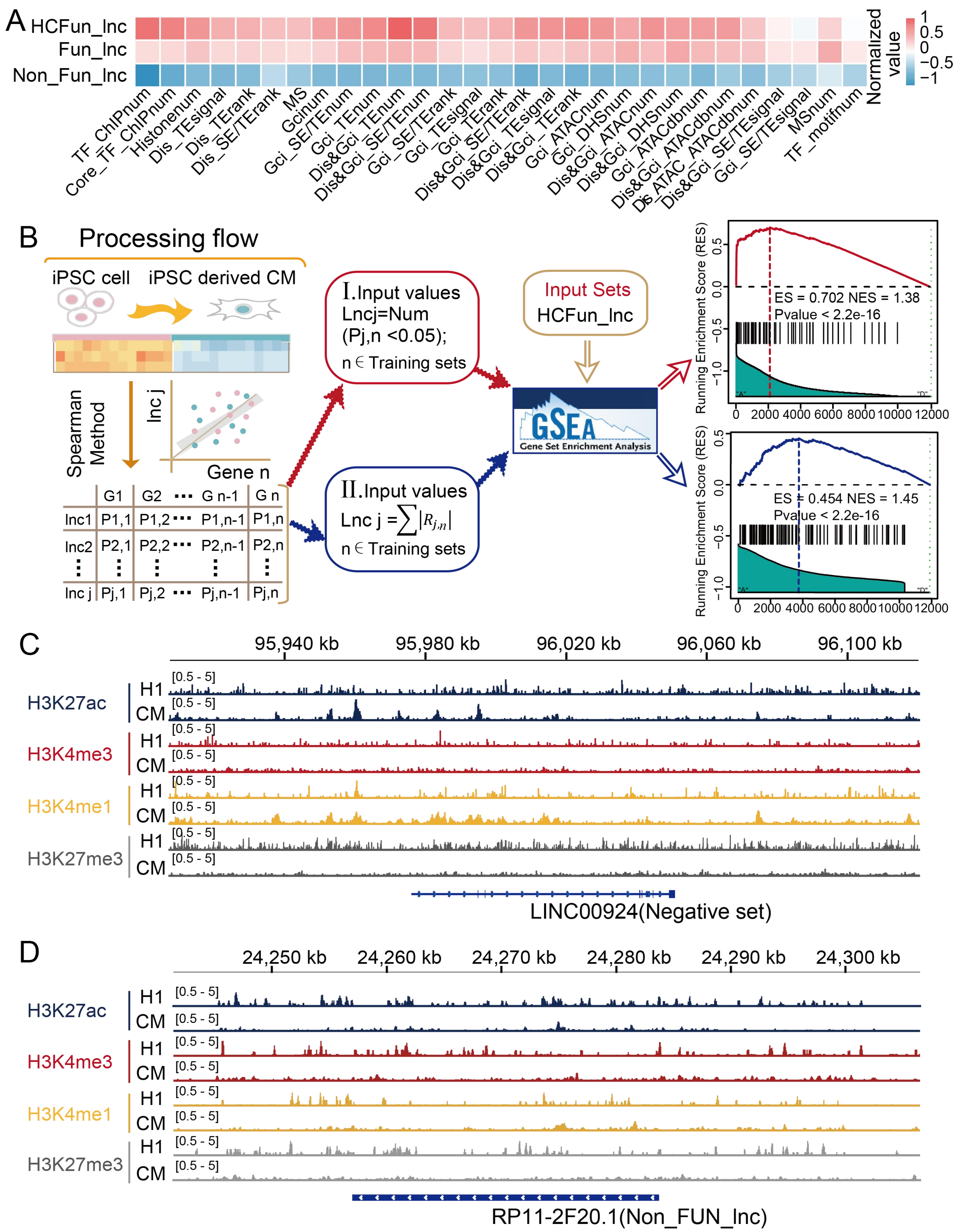

Supplementary Figure 3. Analysis for the powerful functional lncRNAs of HESC model. (A) The heatmap showed the mean feature values of three lncRNA categories for these features with importance scores over 2, where the mean feature values have been standardized. (B) Processing flow chart and analysis result of HCFun_lncs (as Input Sets), the number lncRNAs (* significantly correlated with positive lncRNA sets) were calculated as input values (red). Processing flow chart and analysis result of HCFun_lncs (as Input Sets), the sum of absolute value of correlation coefficients with positive lncRNA sets was assigned as input value for each lncRNA (blue). (C-D) IGV plots for the diverse histone marks in lncRNA promoter regions (LINC00924, RP11-2F20.1) among HESC and HESC-derived-cardiomyocyte samples.

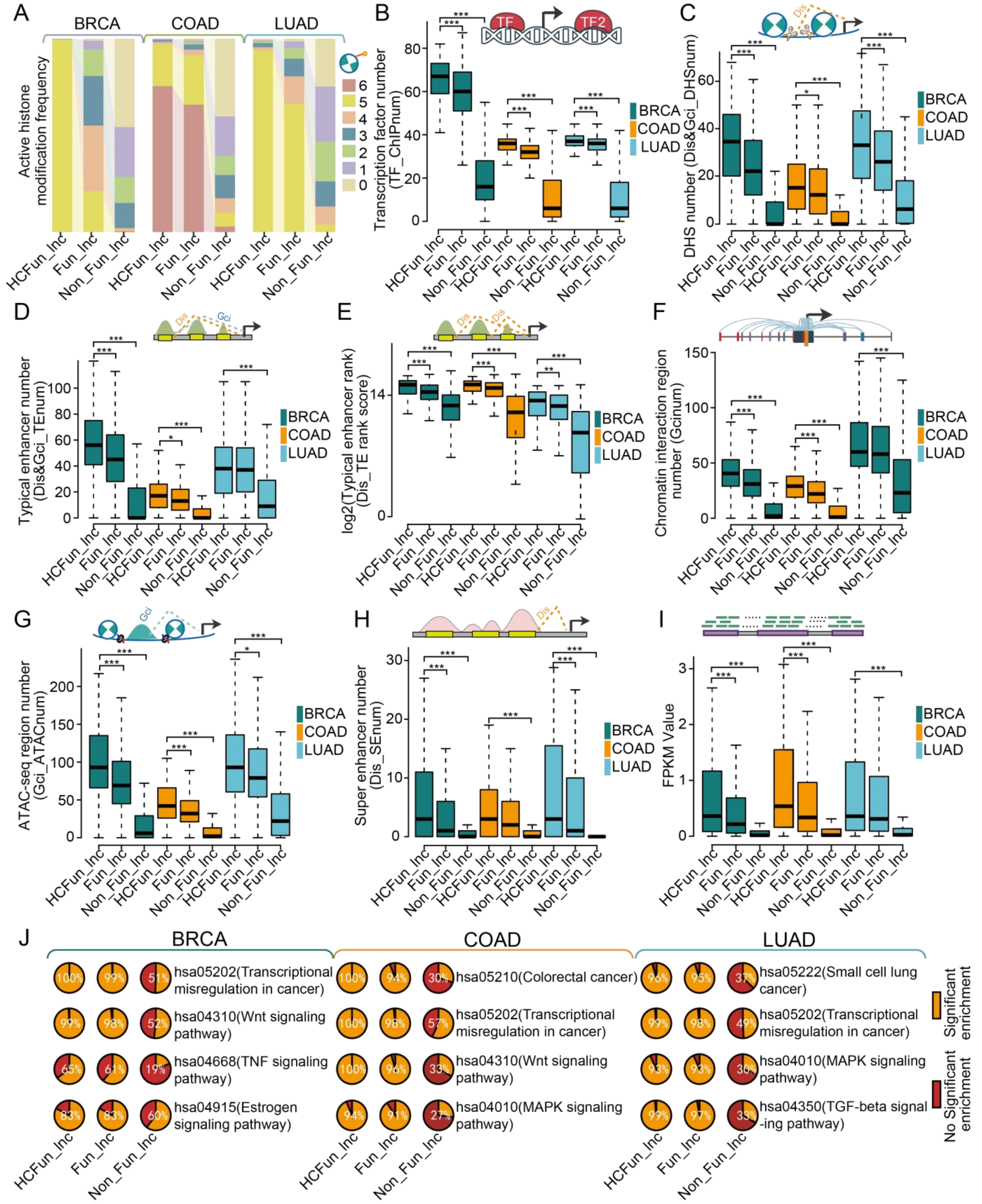

Supplementary Figure 4. Evaluating the HESC model's predictive ability by independent cancer test datasets. (A) Histogram showed the three categories lncRNAs distribution for different histone modifications enrichment among diverse cancer types. (B) The number of TFs occupied in promoter regions of three categories lncRNAs among diverse cancer types. (C-E) The number of DNase-seq regions, TEs and normalized rank scores of TEs of regulating three categories lncRNAs, where their regulatory relationships were identified by ROSE. (F) The number of Chromatin 3D interaction regions. (G) The number of ATAC-seq regions of regulating three categories lncRNAs, where their regulatory relationships were identified by Chromatin 3D interaction analysis. (H) The number of super enhancers of regulating three categories lncRNAs, where their regulatory relationships were identified by ROSE. (I) The expression level of three categories lncRNAs among diverse cancer types. (J) The distribution of cancer important pathway enrichment, the ratio represented proportion of each set that was significantly enriched to the corresponding pathway by TFs of lncRNA promoter region.

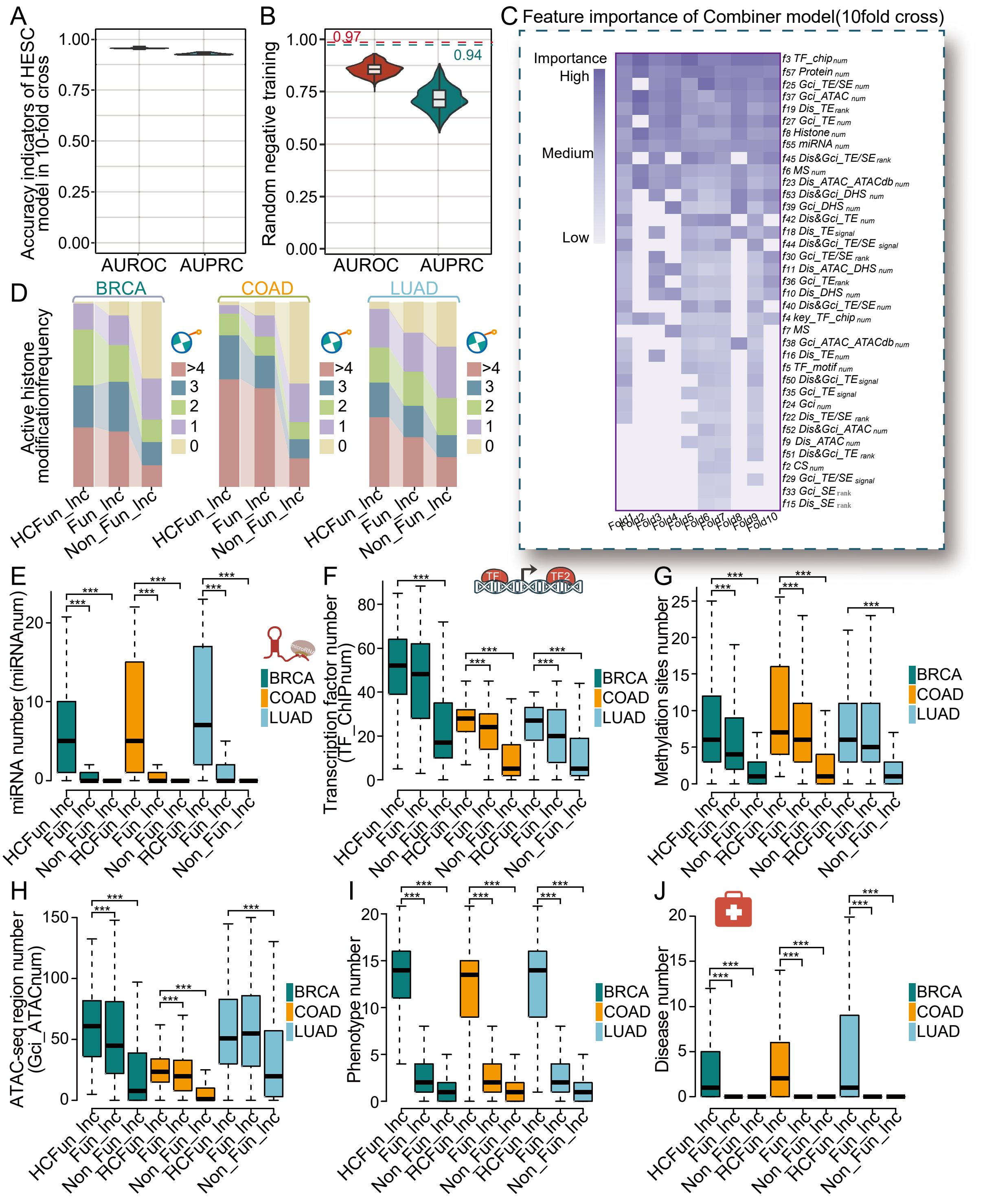

Supplementary Figure 5. Analysis of the Combiner model. (A) Box plot showed AUROC and AUPRC performance indicators obtained from 10-fold cross validation experiment of Combiner model. (B) The accuracy of models constructed by random negative training sets. (C) The heatmap showed importance scores of each feature generated from random forest in 10-fold cross validation experiment of Combiner model. (D) Histogram showed three categories lncRNAs distribution for histone modifications. (E-H) The related miRNA, TF, methylation site and ATAC-seq region number of three categories lncRNAs. (I) The related cancer phenotype number of three categories lncRNAs. (J) The related disease number of three categories lncRNAs.

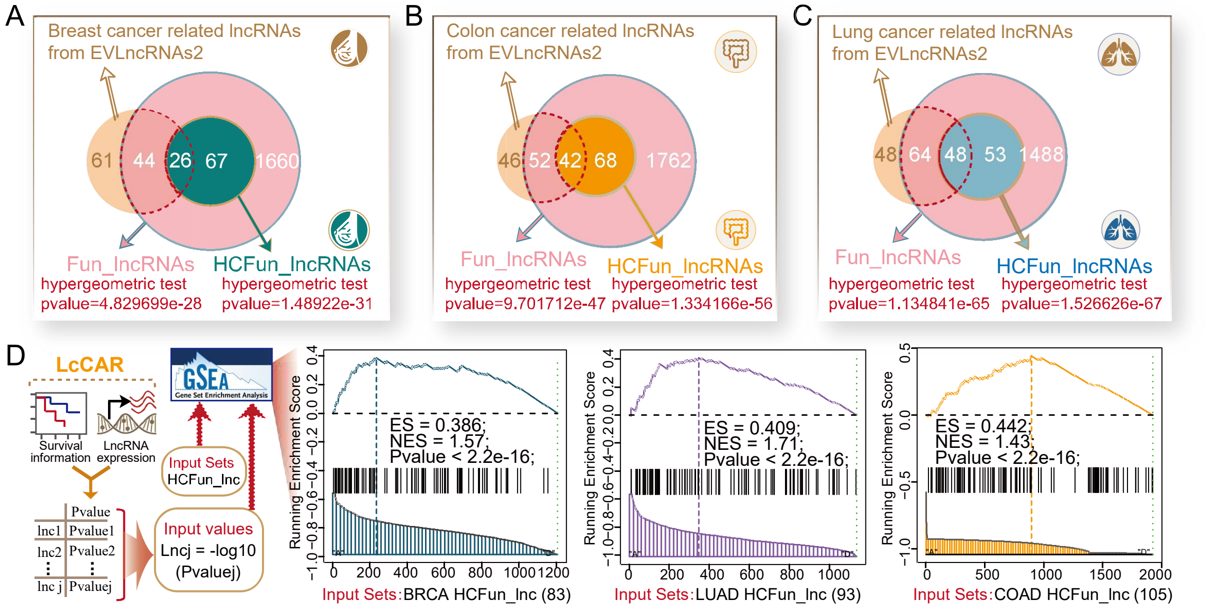

Supplementary Figure 6. Function analysis for the high-confidence functional lncRNAs of the Combiner model. (A) The overlapping between predicting outcome of Combiner model and breast cancer known lncRNAs from EVLncRNAs2, where hypergeometric test Pvalue of two categories lncRNAs were marked, respectively. (B) The overlapping between predicting outcome of Combiner model and colon cancer known lncRNAs from EVLncRNAs2, where hypergeometric test pvalues of two categories lncRNAs were marked, respectively. (C) The overlapping between predicting outcome of Combiner model and lung cancer known lncRNAs from EVLncRNAs2, where hypergeometric test Pvalue of two categories lncRNAs were marked, respectively. (D) Processing flow chart and the analysis result of HCFun_lncs by correlation with cancer survival, where their enrichment scores and normalized enrichment scores have been marked.

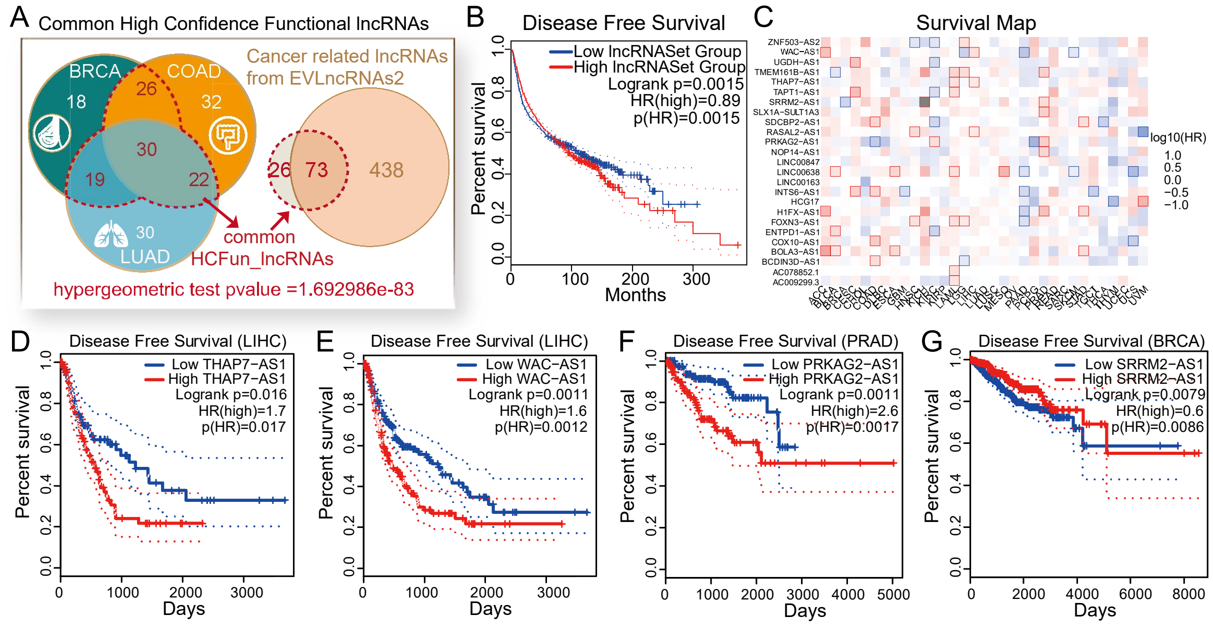

Supplementary Figure 7. Function analysis for the common high-confidence functional lncRNAs of the Combiner model.

(A) The overlapping between common lncRNAs of Combiner model and cancer known lncRNAs from EVLncRNAs2, where hypergeometric test Pvalue was marked. (B) The survival outcome of 26 not-proven commom lncRNAs. (C) The survival map of 26 not-proven commom lncRNAs for 33 cancer types. (D-G) The survival outcomes of 4 lncRNAs in LIHC, PRAD, and BRCA, respectively.

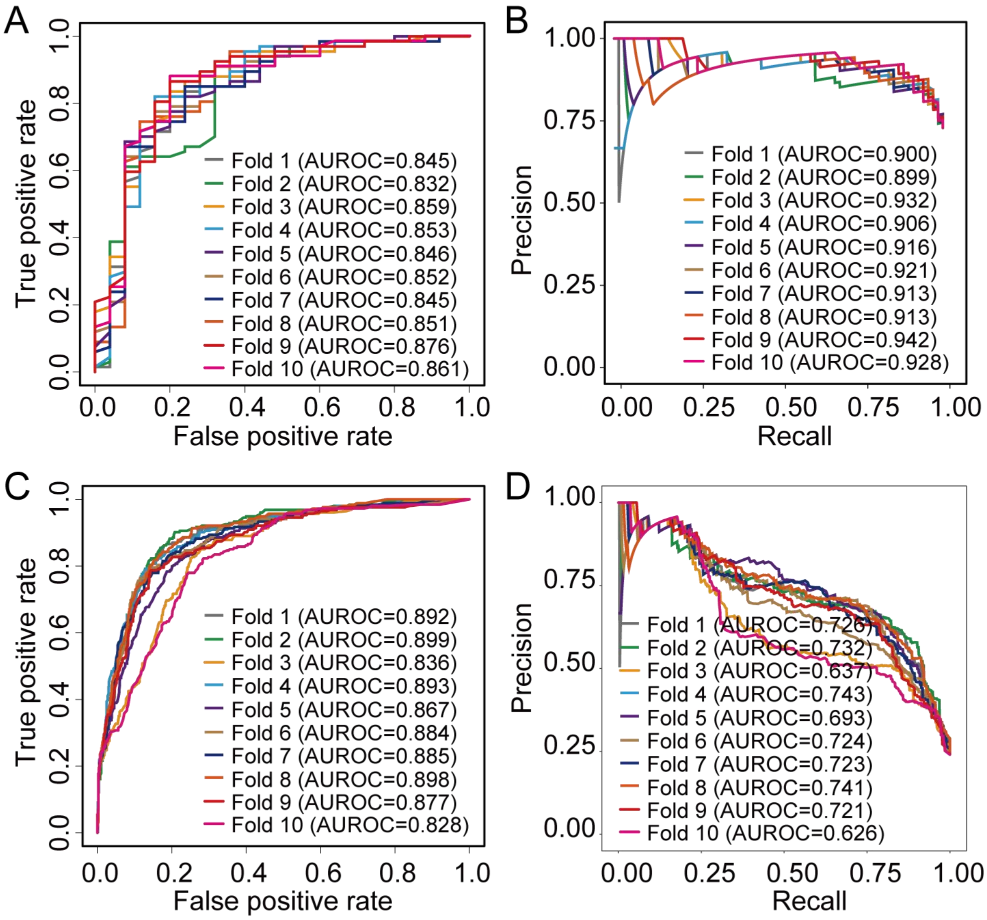

Supplementary Figure 8. The predictive performance of experiments involving data segmentation based on lncRNA sequence similarity. (A-B) The ROC and PRC curves of test sets segmented based on sequence similarity. (C-D) The ROC and PRC curves of independent test sets consisting of cancer-related lncRNAs.

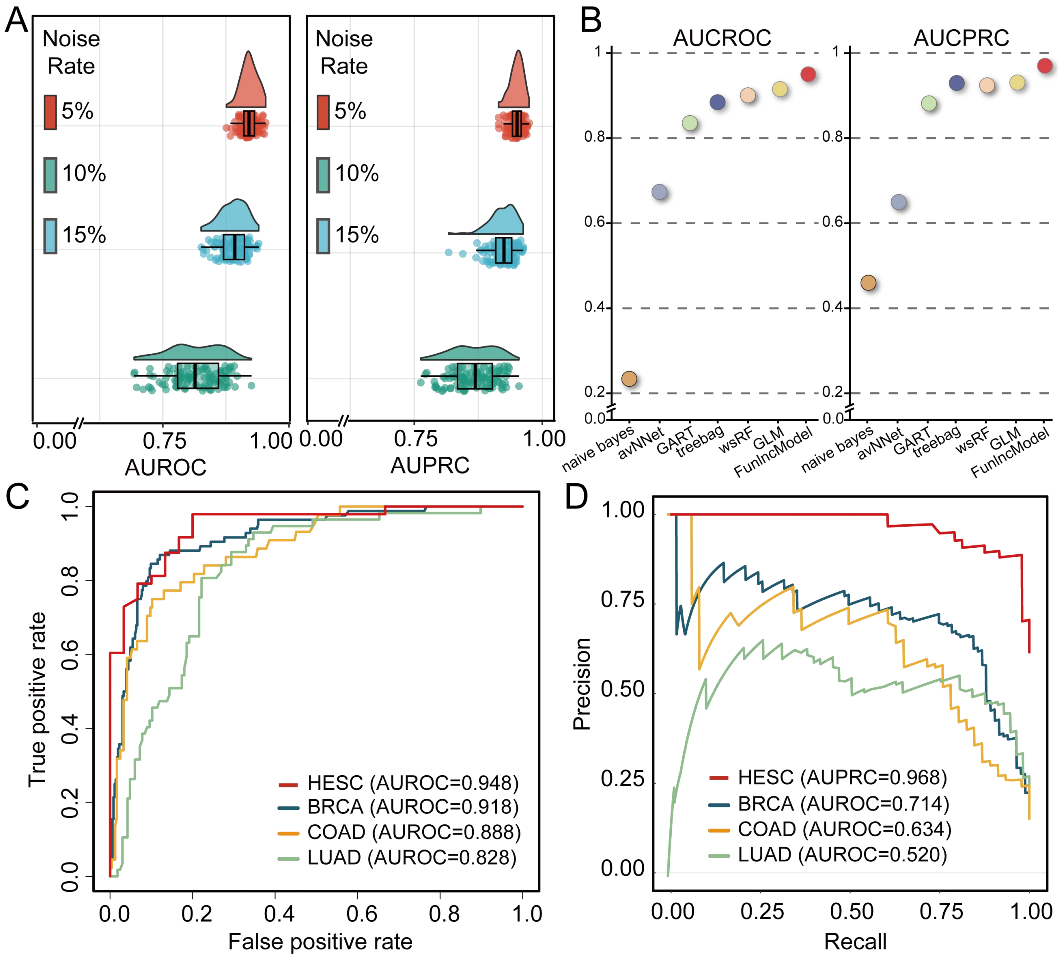

Supplementary Figure 9. The predictive performance of experiments involving data segmentation based on lncRNA sequence similarity. (A) The predictive performance of FunlncModel in the presence of noise during training. (B) The method comparison results where models constructed using all selected features. (C-D) The predictive performances of FunlncModel based on the Random Permutation Score.

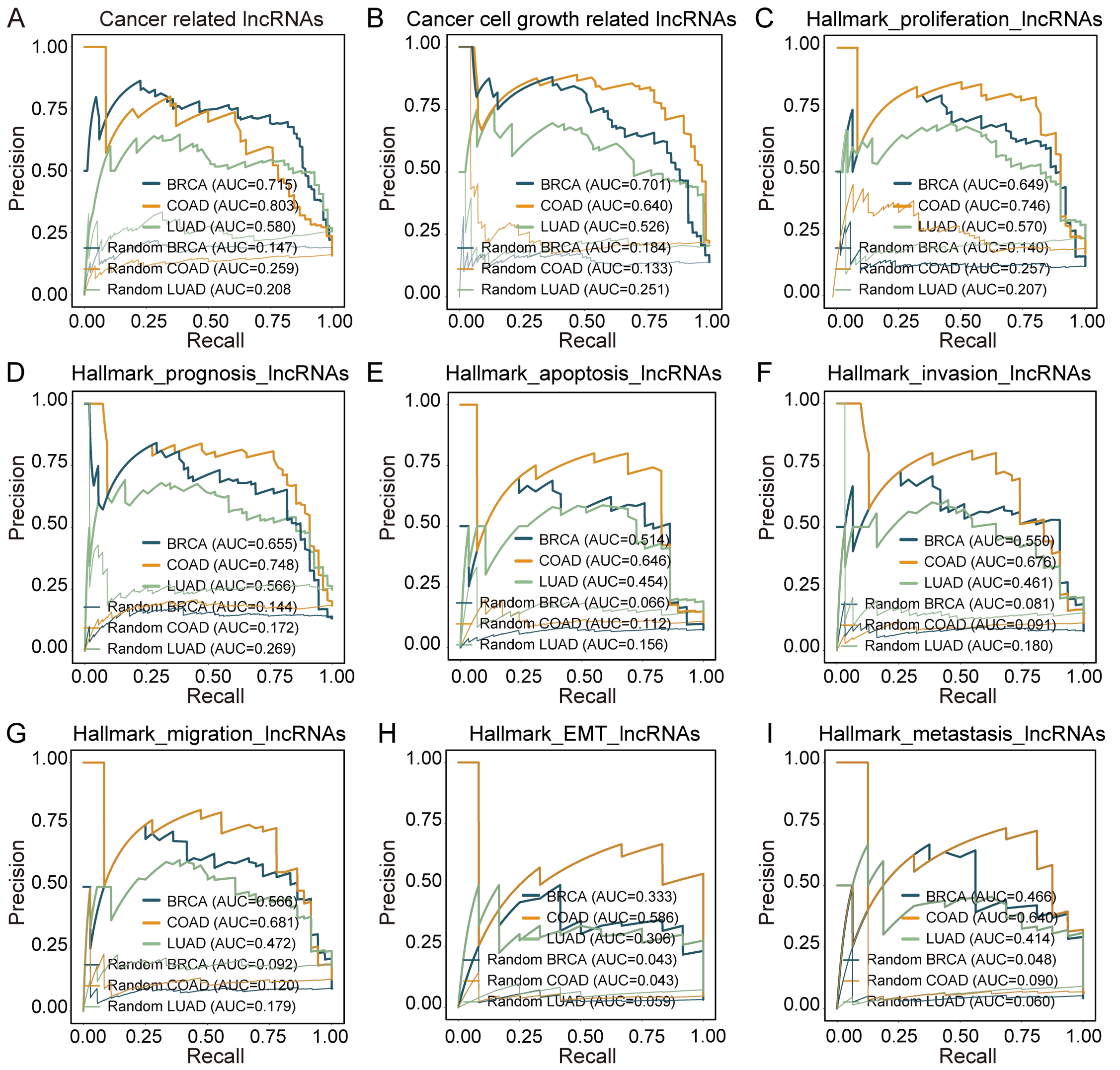

Supplementary Figure 10. Evaluating the HESC model's predictive ability on independent cancer test sets. (A) The PRC curves of known cancer-related lncRNA sets and the control group. (B) The PRC curves of lncRNAs related to cancer cell growth and the control group. (C-I) The PRC curves of 7 cancer hallmark lncRNA sets, including apoptosis, invasion, metastasis, migration, prognosis, EMT, and proliferation, and their control group.
