# Supplementary material for: FunlncModel: integrating multi-omic features from upstream and downstream regulatory networks into a machine learning framework to identify functional lncRNAs
Source: Brief Bioinform. 2024 Nov 27;26(1):bbae623. doi: 10.1093/bib/bbae623 (PMC11601888; doi:10.1093/bib/bbae623)
Supplement: Supplementary_Table1_bbae623 [file supplementary_table1_bbae623.docx]

**Supplementary Table Legends**

**Supplementary Table 1. Detailed description of feature variables.**

| Number | Genomic features | Feature variables | Description |
| --- | --- | --- | --- |
| $f_{1}$ | RiskSNP | ${RS}_{num}$ | The number of riskSNP within lncRNA promoter region. |
| $f_{2}$ | commonSNP | ${CS}_{num}$ | The number of commonSNP within lncRNA promoter region. |
| $f_{3}$ | TF(ChIP-seq) | ${TF\_ChIP}_{num}$ | The number of transcription factors, whose ChIP-seq peak overlapped with the lncRNA promoter region. |
| $f_{4}$ | Core TF(ChIP-seq) | $Core\_{TF\_ChIP}_{num}$ | The number of core transcription factors (POU5F1, SOX2, MYC, KLF4), whose ChIP-seq peak overlapped with the lncRNA promoter region. |
| $f_{5}$ | TF(motif) | ${TF\_motif}_{num}$ | The number of transcription factors, which were identified by FIMO of the MEME suite with significant threshold (1e−6). |
| $f_{6}$ | Methylation site | ${MS}_{num}$ | The normalized number of methylations sits (beta-values > 0.2) within lncRNA promoter region. |
| $f_{7}$ | Methylation value | $MS$ | The normalized value (beta-values *10) of methylation sits within lncRNA promoter region. |
| $f_{8}$ | Histone modification | ${Histone}_{num}$ | The number of transcriptional activation histone modifications, whose ChIP-seq peak overlapped with the lncRNA promoter region. |
| $f_{9}$ | ATAC-seq peak of cistrome & gene_mapper | ${Dis\_ATAC}_{num}$ | The number of ATAC-seq peak associated with lncRNAs, the regulatory relationships were identified via the python script from ROSE (ROSE_geneMapper.py), and the ATAC-seq peak were collected from cistrome. |
| $f_{10}$ | DHS & gene_mapper | ${Dis\_DHS}_{num}$ | The number of DHS associated with lncRNAs, the regulatory relationships were identified via the python script from ROSE (ROSE_geneMapper.py), and the DHS were collected from ENCODE and Roadmap. |
| $f_{11}$ | ATAC of cistrome & DHS & gene_mapper | ${Dis\_ATAC\_DHS}_{num}$ | The total number of DHS and ATAC-seq peak associated with lncRNAs. |
| $f_{12}$ | SE & gene_mapper | ${Dis\_SE}_{num}$ | The number of super enhancers associated with lncRNAs, the regulatory relationships were identified via the python script from ROSE (ROSE_geneMapper.py), and the super enhancers were collected from SEdb. |
| $f_{13}$ | Element number of SE & gene_mapper | ${Dis\_SE}_{ele}$ | The mean element number of super enhancers associated with lncRNAs, the regulatory relationships were identified via the python script from ROSE (ROSE_geneMapper.py). |
| $f_{14}$ | Case ChIP signal of SE & gene_mapper | ${Dis\_SE}_{signal}$ | The mean ChIP-seq signal of super enhancers associated with lncRNAs, the regulatory relationships were identified via the python script from ROSE (ROSE_geneMapper.py). |
| $f_{15}$ | Normalized rank of SE & gene_mapper | ${Dis\_SE}_{rank}$ | The normalized rank was the difference value calculated by the max rank value of all super enhancers and the median rank value of super enhancers associated with lncRNAs. The regulatory relationships were identified via the python script from ROSE (ROSE_geneMapper.py). |
| $f_{16}$ | TE & gene_mapper | ${Dis\_TE}_{num}$ | The number of typical enhancers associated with lncRNAs, the regulatory relationships were identified via the python script from ROSE (ROSE_geneMapper.py), and the enhancers were collected from SEdb. |
| $f_{17}$ | Element number of TE & gene_mapper | ${Dis\_TE}_{ele}$ | The mean element number of typical enhancers associated with lncRNAs, the regulatory relationships were identified via the python script from ROSE (ROSE_geneMapper.py). |
| $f_{18}$ | Case ChIP signal of TE & gene_mapper | ${Dis\_TE}_{signal}$ | The mean ChIP-seq signal of typical enhancers associated with lncRNAs, the regulatory relationships were identified via the python script from ROSE (ROSE_geneMapper.py). |
| $f_{19}$ | Normalized rank of TE & gene_mapper | ${Dis\_TE}_{rank}$ | The normalized rank was the difference value calculated by the max rank value of all typical enhancers and the median rank value of typical enhancers associated with lncRNAs. The regulatory relationships were identified via the python script from ROSE (ROSE_geneMapper.py). |
| $f_{20}$ | Element number of TE/SE & gene_mapper | ${Dis\_TE/SE}_{ele}$ | The mean element number of super enhancers/typical enhancers associated with lncRNAs, the regulatory relationships were identified via the python script from ROSE (ROSE_geneMapper.py). |
| $f_{21}$ | Case ChIP signal of TE/SE & gene_mapper | ${Dis\_TE/SE}_{signal}$ | The mean ChIP-seq signal of super enhancers/typical enhancers associated with lncRNAs, the regulatory relationships were identified via the python script from ROSE (ROSE_geneMapper.py). |
| $f_{22}$ | Normalized rank of TE/SE & gene_mapper | ${Dis\_TE/SE}_{rank}$ | The normalized rank was the difference value calculated by the max rank value of all super enhancers/typical enhancers and the median rank value of super enhancers/typical enhancers associated with lncRNAs. The regulatory relationships were identified via the python script from ROSE (ROSE_geneMapper.py). |
| $f_{23}$ | ATAC of cistrome & ATAC of ATACdb & gene_mapper | ${Dis\_ATAC\_ATACdb}_{num}$ | The number of ATAC-seq peak associated with lncRNAs, the regulatory relationships were identified via the python script from ROSE (ROSE_geneMapper.py). The ATAC-seq peak were the overlapped region between the data of cistrome and the data of ATACdb. |
| $f_{24}$ | Global chromatin interaction | ${Gci}_{num}$ | The number of chromatin interaction region associated with lncRNAs. |
| $f_{25}$ | SE/TE & interaction | ${Gci\_TE/SE}_{num}$ | The number of super enhancers/typical enhancers associated with lncRNAs, the regulatory relationships were identified via the chromatin interaction. |
| $f_{26}$ | SE & interaction | ${Gci\_SE}_{num}$ | The number of super enhancers associated with lncRNAs; the regulatory relationships were identified via the chromatin interaction. |
| $f_{27}$ | TE & interaction | ${Gci\_TE}_{num}$ | The number of typical enhancers associated with lncRNAs, the regulatory relationships were identified via the chromatin interaction. |
| $f_{28}$ | Element number of SE/TE & interaction | ${Gci\_TE/SE}_{ele}$ | The mean element number of super enhancers/typical enhancers associated with lncRNAs, the regulatory relationships were identified via the chromatin interaction. |
| $f_{29}$ | Case ChIP signal of SE/TE & interaction | ${Gci\_TE/SE}_{signal}$ | The mean ChIP-seq signal of super enhancers/typical enhancers associated with lncRNAs, the regulatory relationships were identified via the chromatin interaction. |
| $f_{30}$ | Normalized rank of SE/TE & interaction | ${Gci\_TE/SE}_{rank}$ | The normalized rank was the difference value calculated by the max rank value of all super enhancers/typical enhancers and the median rank value of super enhancers/typical enhancers associated with lncRNAs. The regulatory relationships were identified via the chromatin interaction. |
| $f_{31}$ | Element number of SE & interaction | ${Gci\_SE}_{ele}$ | The mean element number of super enhancers associated with lncRNAs; the regulatory relationships were identified via the chromatin interaction. |
| $f_{32}$ | Case ChIP signal of SE & interaction | ${Gci\_SE}_{signal}$ | The mean ChIP-seq signal of super enhancers associated with lncRNAs, the regulatory relationships were identified via the chromatin interaction. |
| $f_{33}$ | Normalized rank of SE & interaction | ${Gci\_SE}_{rank}$ | The normalized rank was the difference value calculated by the max rank value of all super enhancers and the median rank value of super enhancers associated with lncRNAs. The regulatory relationships were identified via the chromatin interaction. |
| $f_{34}$ | Element number of TE & interaction | ${Gci\_TE}_{ele}$ | The mean element number of typical enhancers associated with lncRNAs, the regulatory relationships were identified via the chromatin interaction. |
| $f_{35}$ | Case ChIP signal of TE & interaction | ${Gci\_TE}_{signal}$ | The mean ChIP-seq signal of typical enhancers associated with lncRNAs, the regulatory relationships were identified via the chromatin interaction. |
| $f_{36}$ | Normalized rank of TE & interaction | ${Gci\_TE}_{rank}$ | The normalized rank was the difference value calculated by the max rank value of all typical enhancers and the median rank value of typical enhancers associated with lncRNAs. The regulatory relationships were identified via the chromatin interaction. |
| $f_{37}$ | ATAC-seq peak of cistrome & interaction | ${Gci\_ATAC}_{num}$ | The number of ATAC-seq peak associated with lncRNAs, the regulatory relationships were identified via the chromatin interaction. The ATAC-seq peak were collected from cistrome. |
| $f_{38}$ | ATAC of ATACdb & interaction | ${Gci\_ATAC\_ATACdb}_{num}$ | The number of ATAC-seq peak associated with lncRNAs, the regulatory relationships were identified via the chromatin interaction. The ATAC-seq peak were collected from ATACdb. |
| $f_{39}$ | DHS & interaction | ${Gci\_DHS}_{num}$ | The number of DHS peak associated with lncRNAs, the regulatory relationships were identified via the chromatin interaction. |
| $f_{40}$ | SE/TE & interaction & gene_mapper | ${Dis\cap Gci\_TE/SE}_{num}$ | The number of super enhancers/typical enhancers associated with lncRNAs, the regulatory relationships were identified via the chromatin interaction and the python script from ROSE (ROSE_geneMapper.py). |
| $f_{41}$ | SE & interaction & gene_mapper | ${Dis\cap Gci\_SE}_{num}$ | The number of super enhancers associated with lncRNAs, the regulatory relationships were identified via the chromatin interaction and the python script from ROSE (ROSE_geneMapper.py). |
| $f_{42}$ | TE & interaction & gene_mapper | ${Dis\cap Gci\_TE}_{num}$ | The number of typical enhancers associated with lncRNAs, the regulatory relationships were identified via the chromatin interaction and the python script from ROSE (ROSE_geneMapper.py). |
| $f_{43}$ | Element number of SE/TE & interaction & gene_mapper | ${Dis\cap Gci\_TE/SE}_{ele}$ | The mean element number of super enhancers/typical enhancers associated with lncRNAs, the regulatory relationships were identified via the chromatin interaction and the python script from ROSE (ROSE_geneMapper.py). |
| $f_{44}$ | Case ChIP signal of SE/TE & interaction & gene_mapper | ${Dis\cap Gci\_TE/SE}_{signal}$ | The mean ChIP-seq signal of super enhancers/typical enhancers associated with lncRNAs, the regulatory relationships were identified via the chromatin interaction and the python script from ROSE (ROSE_geneMapper.py). |
| $f_{45}$ | Normalized rank of SE/TE & interaction & gene_mapper | ${Dis\cap Gci\_TE/SE}_{rank}$ | The normalized rank was the difference value calculated by the max rank value of all super enhancers/typical enhancers and the median rank value of super enhancers/typical enhancers associated with lncRNAs. The regulatory relationships were identified via the chromatin interaction and the python script from ROSE (ROSE_geneMapper.py). |
| $f_{46}$ | Element number of SE & interaction & gene_mapper | ${Dis\cap Gci\_SE}_{ele}$ | The mean element number of super enhancers associated with lncRNAs, the regulatory relationships were identified via the chromatin interaction and the python script from ROSE (ROSE_geneMapper.py). |
| $f_{47}$ | Case ChIP signal of SE & interaction & gene_mapper | ${Dis\cap Gci\_SE}_{signal}$ | The mean ChIP-seq signal of super enhancers associated with lncRNAs, the regulatory relationships were identified via the chromatin interaction and the python script from ROSE (ROSE_geneMapper.py). |
| $f_{48}$ | Normalized rank of SE & interaction & gene_mapper | ${Dis\cap Gci\_SE}_{rank}$ | The normalized rank were the difference value calculated by the max rank value of all super enhancers and the median rank value of super enhancers associated with lncRNAs. The regulatory relationships were identified via the chromatin interaction and the python script from ROSE (ROSE_geneMapper.py). |
| $f_{49}$ | Element number of TE & interaction & gene_mapper | ${Dis\cap Gci\_TE}_{ele}$ | The mean element number of typical enhancers associated with lncRNAs, the regulatory relationships were identified via the chromatin interaction and the python script from ROSE (ROSE_geneMapper.py). |
| $f_{50}$ | Case ChIP signal of TE & interaction & gene_mapper | ${Dis\cap Gci\_TE}_{signal}$ | The mean ChIP-seq signal of typical enhancers associated with lncRNAs, the regulatory relationships were identified via the chromatin interaction and the python script from ROSE (ROSE_geneMapper.py). |
| $f_{51}$ | Normalized rank of TE & interaction & gene_mapper | ${Dis\cap Gci\_TE}_{rank}$ | The normalized rank was the difference value calculated by the max rank value of all typical enhancers and the median rank value of typical enhancers associated with lncRNAs. The regulatory relationships were identified via the chromatin interaction and the python script from ROSE (ROSE_geneMapper.py). |
| $f_{52}$ | ATAC-seq peak of cistrome & interaction & gene_mapper | ${Dis\cap Gci\_ATAC}_{num}$ | The number of ATAC-seq peak associated with lncRNAs, the regulatory relationships were identified via the chromatin interaction and the python script from ROSE (ROSE_geneMapper.py). The ATAC-seq peak were collected from cistrome. |
| $f_{53}$ | DHS & interaction & gene_mapper | ${Dis\cap Gci\_DHS}_{num}$ | The number of DHS peak associated with lncRNAs, the regulatory relationships were identified via the chromatin interaction and the python script from ROSE (ROSE_geneMapper.py). |
| $f_{54}$ | ATAC of ATACdb & interaction & gene_mapper | ${Dis\cap Gci\_ATAC\_ATACdb}_{num}$ | The number of ATAC-seq peak associated with lncRNAs, the regulatory relationships were identified via the chromatin interaction and the python script from ROSE (ROSE_geneMapper.py). The ATAC-seq peak were the overlapped region between the data of cistrome and the data of ATACdb. |
| $f_{55}$ | miRNA | ${miRNA}_{num}$ | The number of miRNAs associated with lncRNAs in network. |
| $f_{56}$ | mRNA | ${mRNA}_{num}$ | The number of mRNAs associated with lncRNAs in network. |
| $f_{57}$ | Protein | ${Protein}_{num}$ | The number of proteins associated with lncRNAs in network. |
