# Supplementary material for: FunlncModel: integrating multi-omic features from upstream and downstream regulatory networks into a machine learning framework to identify functional lncRNAs
Source: Brief Bioinform. 2024 Nov 27;26(1):bbae623. doi: 10.1093/bib/bbae623 (PMC11601888; doi:10.1093/bib/bbae623)
Supplement: Supplementary_Table2_bbae623 [file supplementary_table2_bbae623.docx]

**Supplementary Table 2. The data statistics.**

| **Element** | **Number** | **Sample number** | **Source** |
| --- | --- | --- | --- |
| LncRNA | 13,523 | - | GENCODE |
| Super enhancer | 51,274 | 82 | NCBI, ENCODE, Roadmap and GGR |
| Enhancer | 1,175,517 | 82 | NCBI, ENCODE, Roadmap and GGR |
| Chromatin accessibility region | 3,883,869 | 124 | NCBI, ENCODE, Roadmap and Cistrome |
| Transcription factor | 227 | 2096 | ENCODE, Remap, Cistrome, ChIP-Atlas and GTRD |
| Histone modification | 6 | 79 | ENCODE and Roadmap |
| Methylation site | 280,000 | 7 | ENCODE |
| Chromatin interaction | 9,274,186 | 88 | 4DGenome, Oncobase, 3D Genome Browser |
