# Supplementary material for: FunlncModel: integrating multi-omic features from upstream and downstream regulatory networks into a machine learning framework to identify functional lncRNAs
Source: Brief Bioinform. 2024 Nov 27;26(1):bbae623. doi: 10.1093/bib/bbae623 (PMC11601888; doi:10.1093/bib/bbae623)
Supplement: Supplementary_Table3_bbae623 [file supplementary_table3_bbae623.docx]

| **Supplementary Table 3. H3K27ac ChIP-seq datasets** | | | | | |
| --- | --- | --- | --- | --- | --- |
| **Sample type** | **Sample name** | **Series** | **H3K27ac** | **Input** | **Data sources** |
| HESC | AzH-G2/M | GSE61176 | GSM1498907 | GSM1498915 | NCBI GEO/SRA |
| HESC | AzL-S phase | GSE61176 | GSM1498906 | GSM1498914 | NCBI GEO/SRA |
| HESC | DN-Early G1 | GSE61176 | GSM1498904 | GSM1498912 | NCBI GEO/SRA |
| HESC | Embryonic stem cells | GSE69646 | GSM1705251 | GSM1705255 | NCBI GEO/SRA |
| HESC | Embryonic stem cells | GSE69646 | GSM1705260 | GSM1705264 | NCBI GEO/SRA |
| HESC | H1-hESC | GSE16256 | ENCSR880SUY | ENCSR569NEE | Roadmap |
| HESC | H7 | GSE52657 | GSM1273645 | GSM1273642 | NCBI GEO/SRA |
| HESC | H9 | GSE16256 | ENCSR876RGF | ENCSR214MNL | Roadmap |
| HESC | H9 | NA\|GSE96273 | ENCSR566DVZ | ENCSR625CEM | ENCODE |
| HESC | H9_ESC | GSE24447 | GSM602294 | GSM602292 | NCBI GEO/SRA |
| HESC | H9 | GSE76626 | GSM2029351 | GSM2029353 | NCBI GEO/SRA |
| HESC | H9 | GSE99627 | GSM2648925 | GSM2648929 | NCBI GEO/SRA |
| HESC | H9 | GSE99627 | GSM2648926 | GSM2648930 | NCBI GEO/SRA |
| HESC | H9 | GSE76626 | GSM2029356 | GSM2029358 | NCBI GEO/SRA |
| HESC | H9 | GSE76626 | GSM2029361 | GSM2029363 | NCBI GEO/SRA |
| HESC | H9 | GSE76626 | GSM2029366 | GSM2029368 | NCBI GEO/SRA |
| HESC | KO2-Late G1 | GSE61176 | GSM1498905 | GSM1498913 | NCBI GEO/SRA |
| Lung_Cancer | A549_ethanol_1h | GSM1003578\|GSM1003541 | ENCSR000AUI | ENCSR000ASS | ENCODE |
| Lung_Cancer | A549_dexamethasone_1h | GSM1003493\|GSM1003540 | ENCSR000AVF | ENCSR000ASR | ENCODE |
| Lung_Cancer | A549_TGFβ_72h | GSE90565 | GSM2406904 | GSM2406898 | NCBI GEO/SRA |
| Lung_Cancer | A549_dexamethasone_4h | GSE91282\|GSE91347 | ENCSR543ZVZ | ENCSR872BBM | GGR |
| Lung_Cancer | A549_dexamethasone_0.5h | GSE91201\|GSE91274 | ENCSR102XUM | ENCSR491TAD | GGR |
| Lung_Cancer | A549_dexamethasone_12h | GSE91263\|GSE91286 | ENCSR435JKM | ENCSR549SVK | GGR |
| Lung_Cancer | A549_dexamethasone_7h | GSE91292\|GSE91241 | ENCSR569IBY | ENCSR317VER | GGR |
| Lung_Cancer | A549_dexamethasone_3h | GSE91248\|GSE91242 | ENCSR350EFV | ENCSR325PXS | GGR |
| Lung_Cancer | A549_dexamethasone_1h | GSE91225\|GSE91285 | ENCSR242TBH | ENCSR548SPY | GGR |
| Lung_Cancer | A549_dexamethasone_5h | GSE91326\|GSE91304 | ENCSR716XDB | ENCSR627RBH | GGR |
| Lung_Cancer | A549 | GSE91337\|GSE91343 | ENCSR783SNV | ENCSR838ZAU | GGR |
| Lung_Cancer | A549_dexamethasone_2h | GSE91301\|GSE91246 | ENCSR614NPG | ENCSR344ERJ | GGR |
| Lung_Cancer | A549_dexamethasone_8h | GSE91226\|GSE91232 | ENCSR250EHC | ENCSR274VPI | GGR |
| Lung_Cancer | A549_dexamethasone_6h | GSE91245\|GSE91349 | ENCSR340NAL | ENCSR887SZF | GGR |
| Lung_Cancer | Calu-3 | GSE63398 | GSM1548075 | GSM1548073 | NCBI GEO/SRA |
| Lung_Cancer | NCI-H1299_EGFP-NEO_reporter_cells_DMSO | GSE81322 | GSM2150362 | GSM2150369 | NCBI GEO/SRA |
| Lung_Cancer | NCI-H1299_EGFP-NEO_reporter_cells_SAHA | GSE81322 | GSM2150370 | GSM2150377 | NCBI GEO/SRA |
| Lung_Cancer | NCI-H1299_EGFP-NEO_reporter_cells_SB939 | GSE81322 | GSM2150378 | GSM2150385 | NCBI GEO/SRA |
| Lung_Cancer | NCI-H1299_EGFP-NEO_reporter_cells_DACSB | GSE81322 | GSM2150354 | GSM2150361 | NCBI GEO/SRA |
| Lung_Cancer | NCI-H1299_EGFP-NEO_reporter_cells_DAC | GSE81322 | GSM2150346 | GSM2150353 | NCBI GEO/SRA |
| Colon_Cancer | Caco-2 | GSE96069 | GSM2532773 | GSM2532774 | NCBI GEO/SRA |
| Colon_Cancer | Colo205 | GSE73319 | GSM1890738 | GSM1890739 | NCBI GEO/SRA |
| Colon_Cancer | Colo741 | GSE73319 | GSM1890757 | GSM1890759 | NCBI GEO/SRA |
| Colon_Cancer | HCT116 | GSE96299\|GSE86688 | ENCSR661KMA | ENCSR198WIH | ENCODE |
| Colon_Cancer | HCT116_Heterozygous_ARID1A-KO | GSE71510 | GSM1835983 | GSM1835994 | NCBI GEO/SRA |
| Colon_Cancer | HCT116_Homozygous_ARID1A-KO | GSE71510 | GSM1835984 | GSM1835995 | NCBI GEO/SRA |
| Colon_Cancer | HCT116_Parental | GSE71510 | GSM1835982 | GSM1835993 | NCBI GEO/SRA |
| Colon_Cancer | HCT116 | GSE73319 | GSM1890730 | GSM1890731 | NCBI GEO/SRA |
| Colon_Cancer | HCT116_NONT | GSE97527 | GSM2667958 | GSM2571023 | NCBI GEO/SRA |
| Colon_Cancer | HCT116_shPAF1 | GSE97527 | GSM2667960 | GSM2571026 | NCBI GEO/SRA |
| Colon_Cancer | LS174T | GSE49320 | GSM1365900 | GSM1197323 | NCBI GEO/SRA |
| Colon_Cancer | LS180 | GSE73319 | GSM1890754 | GSM1890756 | NCBI GEO/SRA |
| Breast_Cancer | BT-549 | GSE65201 | GSM1589476 | GSM1589475 | NCBI GEO/SRA |
| Breast_Cancer | CAL51 | GSE69112 | GSM1693015 | GSM1693027 | NCBI GEO/SRA |
| Breast_Cancer | CAL51-MCF-7 | GSE69112 | GSM1693016 | GSM1693028 | NCBI GEO/SRA |
| Breast_Cancer | HCC1954_Parental | GSE72956 | GSM2066435 | GSM2066431 | NCBI GEO/SRA |
| Breast_Cancer | HCC1954_LCC2 | GSE72956 | GSM2066436 | GSM2066432 | NCBI GEO/SRA |
| Breast_Cancer | MCF-7 | GSE96352\|GSE86749 | ENCSR752UOD | ENCSR768LHG | ENCODE |
| Breast_Cancer | MCF-7_10Gygamma_2.5h | GSE100099 | GSM2671298 | GSM2671302 | NCBI GEO/SRA |
| Breast_Cancer | MCF-7_10Gygamma_5h | GSE100099 | GSM2671299 | GSM2671303 | NCBI GEO/SRA |
| Breast_Cancer | MCF-7_10Gygamma_7.5h | GSE100099 | GSM2671300 | GSM2671304 | NCBI GEO/SRA |
| Breast_Cancer | MCF-7_EtOH | GSE57436 | GSM1382472 | GSM1382474 | NCBI GEO/SRA |
| Breast_Cancer | MCF-7_E2 | GSE57436 | GSM1382482 | GSM1382484 | NCBI GEO/SRA |
| Breast_Cancer | MCF-7 | GSE69112 | GSM1693017 | GSM1693029 | NCBI GEO/SRA |
| Breast_Cancer | MCF-7_NS | GSE76732 | GSM2036927 | GSM2036933 | NCBI GEO/SRA |
| Breast_Cancer | MCF-7_PMA | GSE76732 | GSM2036928 | GSM2036934 | NCBI GEO/SRA |
| Breast_Cancer | MCF-7_estradiol | GSE78913 | GSM2175784 | GSM2175786 | NCBI GEO/SRA |
| Breast_Cancer | MDA-MB-231_untreat | GSE69112 | GSM1693018 | GSM1693030 | NCBI GEO/SRA |
| Breast_Cancer | MDA-MB-231_GATA3 | GSE72141 | GSM1855993 | GSM1855999 | NCBI GEO/SRA |
| Breast_Cancer | MDA-MB-231_TA1del | GSE72141 | GSM1855995 | GSM1856001 | NCBI GEO/SRA |
| Breast_Cancer | MDA-MB-231 | GSE76732 | GSM2036929 | GSM2036935 | NCBI GEO/SRA |
| Breast_Cancer | MDA-MB-231-MCF-7 | GSE69112 | GSM1693019 | GSM1693031 | NCBI GEO/SRA |
| Breast_Cancer | MDA-MB-468 | GSE65201 | GSM1589470 | GSM1589471 | NCBI GEO/SRA |
| Breast_Cancer | SUM159_DMSO_24h | GSE87418 | GSM2330561 | GSM2330573 | NCBI GEO/SRA |
| Breast_Cancer | SUM159PT-BT-474 | GSE69112 | GSM1693020 | GSM1693032 | NCBI GEO/SRA |
| Breast_Cancer | SUM159PT-MCF-7 | GSE69112 | GSM1693022 | GSM1693033 | NCBI GEO/SRA |
| Breast_Cancer | SUM159PT-T47D | GSE69112 | GSM1693023 | GSM1693034 | NCBI GEO/SRA |
| Breast_Cancer | SUM159PT-ZR-75-1 | GSE69112 | GSM1693024 | GSM1693035 | NCBI GEO/SRA |
| Breast_Cancer | T-47D_1 | GSE63109 | GSM1541449 | GSM1541454 | NCBI GEO/SRA |
| Breast_Cancer | T-47D_2 | GSE65201 | GSM1589474 | GSM1589473 | NCBI GEO/SRA |
| Breast_Cancer | T-47D_untreat | GSE69112 | GSM1693025 | GSM1693036 | NCBI GEO/SRA |
| Breast_Cancer | ZR-75-1_1 | GSE65201 | GSM1589472 | GSM1589477 | NCBI GEO/SRA |
| Breast_Cancer | ZR-75-1_2 | GSE69112 | GSM1693026 | GSM1693037 | NCBI GEO/SRA |
| Breast_Cancer | ZR-75-30_parental | GSE71323 | GSM1832646 | GSM1832650 | NCBI GEO/SRA |
| Breast_Cancer | ZR-75-30_KO | GSE71323 | GSM1832647 | GSM1832651 | NCBI GEO/SRA |
