# Supplementary material for: FunlncModel: integrating multi-omic features from upstream and downstream regulatory networks into a machine learning framework to identify functional lncRNAs
Source: Brief Bioinform. 2024 Nov 27;26(1):bbae623. doi: 10.1093/bib/bbae623 (PMC11601888; doi:10.1093/bib/bbae623)
Supplement: Supplementary_Table4_bbae623 [file supplementary_table4_bbae623.docx]

| **Supplementary Table 4. Chromatin accessibility datasets** | | | | |
| --- | --- | --- | --- | --- |
| **Sample type** | **Sample name** | **Class** | **Series** | **Data sources** |
| HESC | H9 | ATAC-seq | GSM3391512 | ATACdb |
| HESC | H9 | ATAC-seq | GSM3391513 | ATACdb |
| HESC | H1 | ATAC-seq | GSM2286656 | ATACdb |
| HESC | H1 | ATAC-seq | GSM2286657 | ATACdb |
| HESC | H9 | ATAC-seq | GSM2829006 | ATACdb |
| HESC | H9 | ATAC-seq | GSM2829007 | ATACdb |
| HESC | H9 | ATAC-seq | GSM2829008 | ATACdb |
| HESC | H9 | ATAC-seq | GSM2829009 | ATACdb |
| HESC | H9 | ATAC-seq | GSM2829010 | ATACdb |
| HESC | H9 | ATAC-seq | GSM2829011 | ATACdb |
| HESC | H9 | ATAC-seq | GSM2829012 | ATACdb |
| HESC | H9 | ATAC-seq | GSM2829013 | ATACdb |
| HESC | HESC | ATAC-seq | GSM2584741 | ATACdb |
| HESC | HESC | ATAC-seq | GSM2584742 | ATACdb |
| HESC | HESC | ATAC-seq | GSM2584743 | ATACdb |
| HESC | HESC | ATAC-seq | GSM2584744 | ATACdb |
| HESC | HESC | ATAC-seq | GSM2584745 | ATACdb |
| HESC | Embryo | ATAC-seq | GSM2140799 | ATACdb |
| HESC | Embryo | ATAC-seq | GSM2140808 | ATACdb |
| HESC | Embryo | ATAC-seq | GSM2386576 | ATACdb |
| HESC | Embryo | ATAC-seq | GSM2386577 | ATACdb |
| HESC | Embryo | ATAC-seq | GSM2386578 | ATACdb |
| HESC | Embryo | ATAC-seq | GSM2386580 | ATACdb |
| HESC | Embryo | ATAC-seq | GSM2386582 | ATACdb |
| HESC | Embryo | ATAC-seq | GSM2386583 | ATACdb |
| HESC | H9 | ATAC-seq | GSM2893676 | ATACdb |
| HESC | H9 | ATAC-seq | GSM2893677 | ATACdb |
| HESC | H9 | ATAC-seq | GSM2893678 | ATACdb |
| HESC | H9 | ATAC-seq | GSM2893679 | ATACdb |
| HESC | H9 | ATAC-seq | GSM2893680 | ATACdb |
| HESC | H9 | ATAC-seq | GSM3074520 | ATACdb |
| HESC | H9 | ATAC-seq | GSM3146457 | ATACdb |
| HESC | H9 | ATAC-seq | GSM3146458 | ATACdb |
| HESC | H9 | ATAC-seq | GSM3146459 | ATACdb |
| HESC | H9 | ATAC-seq | GSM3146460 | ATACdb |
| HESC | H9 | ATAC-seq | GSM3146461 | ATACdb |
| HESC | H9 | ATAC-seq | GSM3146462 | ATACdb |
| HESC | H9 | ATAC-seq | GSM3146464 | ATACdb |
| HESC | H9 | ATAC-seq | GSM3146465 | ATACdb |
| HESC | H9 | ATAC-seq | GSM3146466 | ATACdb |
| HESC | H9 | ATAC-seq | GSM3146467 | ATACdb |
| HESC | H9 | ATAC-seq | GSM3146468 | ATACdb |
| HESC | H9 | ATAC-seq | GSM3444160 | ATACdb |
| HESC | H9 | ATAC-seq | GSM3444161 | ATACdb |
| HESC | H9 | ATAC-seq | GSM3444162 | ATACdb |
| HESC | H9 | ATAC-seq | GSM3444163 | ATACdb |
| HESC | H9 | ATAC-seq | GSM3444164 | ATACdb |
| HESC | H9 | ATAC-seq | GSM3444165 | ATACdb |
| Colon_Cancer | YB5 | ATAC-seq | GSM3110079 | ATACdb |
| Colon_Cancer | YB5 | ATAC-seq | GSM3110080 | ATACdb |
| Colon_Cancer | YB5 | ATAC-seq | GSM3110081 | ATACdb |
| Colon_Cancer | YB5 | ATAC-seq | GSM3110082 | ATACdb |
| Colon_Cancer | YB5 | ATAC-seq | GSM3110083 | ATACdb |
| Colon_Cancer | YB5 | ATAC-seq | GSM3110084 | ATACdb |
| Colon_Cancer | HCT116 | ATAC-seq | GSM2572568 | ATACdb |
| Colon_Cancer | HCT116 cell | ATAC-seq | GSM2719724 | ATACdb |
| Colon_Cancer | HCT116 cell | ATAC-seq | GSM2719725 | ATACdb |
| Colon_Cancer | HCT116 cell | ATAC-seq | GSM2719726 | ATACdb |
| Colon_Cancer | HCT116 cell | ATAC-seq | GSM2719727 | ATACdb |
| Colon_Cancer | HCT116 cell | ATAC-seq | GSM2719730 | ATACdb |
| Colon_Cancer | HCT116 cell | ATAC-seq | GSM2719731 | ATACdb |
| Lung_Cancer | H1703 | ATAC-seq | GSM3445655 | ATACdb |
| Lung_Cancer | H1703 | ATAC-seq | GSM3445657 | ATACdb |
| Lung_Cancer | PC9 | ATAC-seq | GSM2692559 | ATACdb |
| Lung_Cancer | PC9 | ATAC-seq | GSM2692560 | ATACdb |
| Lung_Cancer | PC9 | ATAC-seq | GSM2692561 | ATACdb |
| Lung_Cancer | PC9 | ATAC-seq | GSM2692562 | ATACdb |
| Lung_Cancer | PC9 | ATAC-seq | GSM2692563 | ATACdb |
| Lung_Cancer | PC9 | ATAC-seq | GSM2692564 | ATACdb |
| Lung_Cancer | PC9 | ATAC-seq | GSM2692565 | ATACdb |
| Lung_Cancer | PC9 | ATAC-seq | GSM2692566 | ATACdb |
| Lung_Cancer | PC9 | ATAC-seq | GSM2692567 | ATACdb |
| Lung_Cancer | PC9 | ATAC-seq | GSM2692568 | ATACdb |
| Lung_Cancer | PC9 | ATAC-seq | GSM2692569 | ATACdb |
| Lung_Cancer | PC9 | ATAC-seq | GSM2692570 | ATACdb |
| Lung_Cancer | PC9 | ATAC-seq | GSM2692571 | ATACdb |
| Lung_Cancer | PC9 | ATAC-seq | GSM2692572 | ATACdb |
| Lung_Cancer | PC9 | ATAC-seq | GSM2692573 | ATACdb |
| Lung_Cancer | PC9 | ATAC-seq | GSM2692574 | ATACdb |
| Lung_Cancer | PC9 | ATAC-seq | GSM2692575 | ATACdb |
| Lung_Cancer | PC9 | ATAC-seq | GSM2692576 | ATACdb |
| Lung_Cancer | PC9 | ATAC-seq | GSM1904729 | ATACdb |
| Lung_Cancer | PC9 | ATAC-seq | GSM1904730 | ATACdb |
| Lung_Cancer | PC9 | ATAC-seq | GSM1904731 | ATACdb |
| Breast_Cancer | T47D | ATAC-seq | GSM2692628 | ATACdb |
| Breast_Cancer | T47D | ATAC-seq | GSM2692629 | ATACdb |
| Breast_Cancer | T47D | ATAC-seq | GSM2692630 | ATACdb |
| Breast_Cancer | T47D | ATAC-seq | GSM2692631 | ATACdb |
| Breast_Cancer | T47D | ATAC-seq | GSM2692632 | ATACdb |
| Breast_Cancer | T47D | ATAC-seq | GSM2692633 | ATACdb |
| Breast_Cancer | MCF10a | ATAC-seq | GSM2357378 | ATACdb |
| Breast_Cancer | MCF10a | ATAC-seq | GSM2357380 | ATACdb |
| HESC | H7 | ATAC-seq | GSM2257293 | Cistrome |
| HESC | H9 | ATAC-seq | GSM2425099 | Cistrome |
| HESC | HUES64 | ATAC-seq | GSM2977489 | Cistrome |
| HESC | iPSC | ATAC-seq | GSM2898830 | Cistrome |
| HESC | iPSC-CM | ATAC-seq | GSM2898851 | Cistrome |
| Lung_Cancer | HRE | DNase-seq | 45005_sort_peaks.narrowPeak.bed | Cistrome |
| Colon_Cancer | HCT116 | ATAC-seq | GSM2719731 | Cistrome |
| Breast_Cancer | MCF-7 | ATAC-seq | GSM2714247 | Cistrome |
| Breast_Cancer | MDA-MB-231 | ATAC-seq | GSM1855976 | Cistrome |
| Breast_Cancer | T-47D | ATAC-seq | GSM2644562 | Cistrome |
| Breast_Cancer | MDA-MB-231 | DNase-seq | 78266_sort_peaks.narrowPeak.bed | Cistrome |
| Breast_Cancer | ZR-75-1 | DNase-seq | 78686_sort_peaks.narrowPeak.bed | Cistrome |
| HESC | H1 | DNase-seq | - | ENCODE |
| HESC | H7 | DNase-seq | - | ENCODE |
| HESC | H9 | DNase-seq | - | ENCODE |
| HESC | iPS-DF-19.11 | DNase-seq | - | ENCODE |
| HESC | iPS-DF-4.7 | DNase-seq | - | ENCODE |
| Breast_Cancer | MCF-7 | DNase-seq | - | ENCODE |
| Breast_Cancer | T-47D | DNase-seq | - | ENCODE |
| HESC | H1 | ATAC-seq | GSM3094771 | NCBI |
| HESC | HUES48 | DNase-seq | - | Roadmap |
| HESC | HUES6 | DNase-seq | - | Roadmap |
| HESC | HUES64 | DNase-seq | - | Roadmap |
| HESC | iPS-15b | DNase-seq | - | Roadmap |
| HESC | iPS-18 | DNase-seq | - | Roadmap |
| HESC | iPS-20b | DNase-seq | - | Roadmap |
| HESC | iPS-DF-6.9 | DNase-seq | - | Roadmap |
| Lung_Cancer | A549 | DNase-seq | - | Roadmap |
| Lung_Cancer | LUAD | ATAC-seq | - | TCGA |
| Lung_Cancer | LUSC | ATAC-seq | - | TCGA |
| Colon_Cancer | COAD | ATAC-seq | - | TCGA |
| Breast_Cancer | BRCA | ATAC-seq | - | TCGA |
