# Supplementary material for: FunlncModel: integrating multi-omic features from upstream and downstream regulatory networks into a machine learning framework to identify functional lncRNAs
Source: Brief Bioinform. 2024 Nov 27;26(1):bbae623. doi: 10.1093/bib/bbae623 (PMC11601888; doi:10.1093/bib/bbae623)
Supplement: Supplementary_Table5_bbae623 [file supplementary_table5_bbae623.docx]

| **Supplementary Table 5. TF ChIP-seq datasets** | | | | | | |
| --- | --- | --- | --- | --- | --- | --- |
| **Sample type** | **TF** | **TFclass** | **Sample name** | **Series** | **TFbedfilename** | **Data sources** |
| HESC | SRF | alpha-Helices exposed by beta-structures | HUES64 | GSM1505772 | 51590_sort_peaks.narrowPeak.bed.bed | Cistrome |
| HESC | SRF | alpha-Helices exposed by beta-structures | HUES64 | GSM1505773 | 51591_sort_peaks.narrowPeak.bed.bed | Cistrome |
| HESC | SRF | alpha-Helices exposed by beta-structures | HUES64 | GSM1505774 | 51592_sort_peaks.narrowPeak.bed.bed | Cistrome |
| HESC | SRF | alpha-Helices exposed by beta-structures | HUES64 | GSM1505776 | 51594_sort_peaks.narrowPeak.bed.bed | Cistrome |
| HESC | SRF | alpha-Helices exposed by beta-structures | HUES64 | GSM1505777 | 51595_sort_peaks.narrowPeak.bed.bed | Cistrome |
| HESC | SRF | alpha-Helices exposed by beta-structures | HUES64 | GSM1505778 | 51596_sort_peaks.narrowPeak.bed.bed | Cistrome |
| HESC | SRF | alpha-Helices exposed by beta-structures | H1 | GSM803425 | 46167_sort_peaks.narrowPeak.bed.bed | ENCODE |
| HESC | MYC | Basic domains | H1 | GSM748531 | 8096_sort_peaks.narrowPeak.bed.bed | Cistrome |
| HESC | MAX | Basic domains | H1 | GSM935348 | 45682_sort_peaks.narrowPeak.bed.bed | Cistrome |
| HESC | USF2 | Basic domains | H1 | GSM935380 | 45694_sort_peaks.narrowPeak.bed.bed | Cistrome |
| HESC | JUND | Basic domains | H1 | GSM935434 | 45696_sort_peaks.narrowPeak.bed.bed | Cistrome |
| HESC | MYC | Basic domains | H1 | GSM935509 | 45691_sort_peaks.narrowPeak.bed.bed | Cistrome |
| HESC | BACH1 | Basic domains | H1 | GSM935580 | 45690_sort_peaks.narrowPeak.bed.bed | Cistrome |
| HESC | JUN | Basic domains | H1 | GSM935614 | 45685_sort_peaks.narrowPeak.bed.bed | Cistrome |
| HESC | TFAP2C | Basic domains | H9 | GSM2817670 | 81808_sort_peaks.narrowPeak.bed.bed | Cistrome |
| HESC | TFAP2A | Basic domains | H9 | GSM2817671 | 81809_sort_peaks.narrowPeak.bed.bed | Cistrome |
| HESC | TFAP2A | Basic domains | H9 | GSM2817672 | 81807_sort_peaks.narrowPeak.bed.bed | Cistrome |
| HESC | TFAP2C | Basic domains | H9 | GSM2817674 | 81806_sort_peaks.narrowPeak.bed.bed | Cistrome |
| HESC | TFAP2A | Basic domains | H9 | GSM2817676 | 81805_sort_peaks.narrowPeak.bed.bed | Cistrome |
| HESC | HEY1 | Basic domains | HUES64 | GSM1505679 | 51497_sort_peaks.narrowPeak.bed.bed | Cistrome |
| HESC | TAL1 | Basic domains | HUES64 | GSM1505785 | 51603_sort_peaks.narrowPeak.bed.bed | Cistrome |
| HESC | TAL1 | Basic domains | HUES64 | GSM1505786 | 51604_sort_peaks.narrowPeak.bed.bed | Cistrome |
| HESC | TCF4 | Basic domains | HUES64 | GSM1505790 | 51608_sort_peaks.narrowPeak.bed.bed | Cistrome |
| HESC | TCF4 | Basic domains | HUES64 | GSM1505791 | 51609_sort_peaks.narrowPeak.bed.bed | Cistrome |
| HESC | MYC | Basic domains | HUES64 | GSM1505809 | 51627_sort_peaks.narrowPeak.bed.bed | Cistrome |
| HESC | MYC | Basic domains | HUES64 | GSM1505810 | 51628_sort_peaks.narrowPeak.bed.bed | Cistrome |
| HESC | MYC | Basic domains | iPS | GSM2183797 | 88363_sort_peaks.narrowPeak.bed.bed | Cistrome |
| HESC | MYC | Basic domains | iPS | GSM2183798 | 88371_sort_peaks.narrowPeak.bed.bed | Cistrome |
| HESC | MAFK | Basic domains | H1 | GSM935292 | 45687_sort_peaks.narrowPeak.bed.bed | ENCODE |
| HESC | MXI1 | Basic domains | H1 | GSM935293 | 45695_sort_peaks.narrowPeak.bed.bed | ENCODE |
| HESC | CEBPB | Basic domains | H1 | GSM935295 | 45681_sort_peaks.narrowPeak.bed.bed | ENCODE |
| HESC | NRF1 | Basic domains | H1 | GSM935308 | 45697_sort_peaks.narrowPeak.bed.bed | ENCODE |
| HESC | ATF2 | Basic domains | H1 | GSM1010742 | 46193_sort_peaks.narrowPeak.bed.bed | ENCODE |
| HESC | CREB1 | Basic domains | H1 | GSM1010896 | 46182_sort_peaks.narrowPeak.bed.bed | ENCODE |
| HESC | MAX | Basic domains | H1 | GSM1010898 | 46180_sort_peaks.narrowPeak.bed.bed | ENCODE |
| HESC | FOSL1 | Basic domains | H1 | GSM803382 | 46175_sort_peaks.narrowPeak.bed.bed | ENCODE |
| HESC | USF1 | Basic domains | H1 | GSM803426 | 46171_sort_peaks.narrowPeak.bed.bed | ENCODE |
| HESC | TCF12 | Basic domains | H1 | GSM803427 | 46166_sort_peaks.narrowPeak.bed.bed | ENCODE |
| HESC | MYC | Basic domains | H1 | GSM822274 | 35837_sort_peaks.narrowPeak.bed.bed | ENCODE |
| HESC | ATF3 | Basic domains | H1 | GSM803512 | 46174_sort_peaks.narrowPeak.bed.bed | ENCODE |
| HESC | JUND | Basic domains | H1 | GSM803529 | 46191_sort_peaks.narrowPeak.bed.bed | ENCODE |
| HESC | MYC | Basic domains | H1 | GSM447585 | GSE17917.MYC.ESC_remap2018_hg19__v1_2.bed | Remap |
| HESC | SMAD2 | beta-Hairpin exposed by an alpha/beta-scaffold | H1 | GSM2635691 | SRX2844311.bed | ChIP-Atlas |
| HESC | SMAD2 | beta-Hairpin exposed by an alpha/beta-scaffold | H1 | GSM2635692 | SRX2844312.bed | ChIP-Atlas |
| HESC | SMAD2 | beta-Hairpin exposed by an alpha/beta-scaffold | H9 | GSM761755 | 5363_sort_peaks.narrowPeak.bed.bed | Cistrome |
| HESC | SMAD2 | beta-Hairpin exposed by an alpha/beta-scaffold | H9 | GSM761756 | 5364_sort_peaks.narrowPeak.bed.bed | Cistrome |
| HESC | SMAD2 | beta-Hairpin exposed by an alpha/beta-scaffold | H9 | GSM761757 | 5365_sort_peaks.narrowPeak.bed.bed | Cistrome |
| HESC | SMAD2 | beta-Hairpin exposed by an alpha/beta-scaffold | H9 | GSM761758 | 5366_sort_peaks.narrowPeak.bed.bed | Cistrome |
| HESC | SMAD3 | beta-Hairpin exposed by an alpha/beta-scaffold | BG03 | GSM539547 | 276_sort_peaks.narrowPeak.bed.bed | Cistrome |
| HESC | SMAD3 | beta-Hairpin exposed by an alpha/beta-scaffold | BG03 | GSM539548 | 278_sort_peaks.narrowPeak.bed.bed | Cistrome |
| HESC | SMAD3 | beta-Hairpin exposed by an alpha/beta-scaffold | BG03 | GSM897116 | 34012_sort_peaks.narrowPeak.bed.bed | Cistrome |
| HESC | SMAD3 | beta-Hairpin exposed by an alpha/beta-scaffold | BG03 | GSM897117 | 34016_sort_peaks.narrowPeak.bed.bed | Cistrome |
| HESC | SMAD3 | beta-Hairpin exposed by an alpha/beta-scaffold | BG03 | GSM897118 | 34017_sort_peaks.narrowPeak.bed.bed | Cistrome |
| HESC | SMAD1 | beta-Hairpin exposed by an alpha/beta-scaffold | HUES64 | GSM1505736 | 51554_sort_peaks.narrowPeak.bed.bed | Cistrome |
| HESC | SMAD1 | beta-Hairpin exposed by an alpha/beta-scaffold | HUES64 | GSM1505737 | 51555_sort_peaks.narrowPeak.bed.bed | Cistrome |
| HESC | SMAD1 | beta-Hairpin exposed by an alpha/beta-scaffold | HUES64 | GSM1505738 | 51556_sort_peaks.narrowPeak.bed.bed | Cistrome |
| HESC | SMAD1 | beta-Hairpin exposed by an alpha/beta-scaffold | HUES64 | GSM1505739 | 51557_sort_peaks.narrowPeak.bed.bed | Cistrome |
| HESC | SMAD1 | beta-Hairpin exposed by an alpha/beta-scaffold | HUES64 | GSM1505740 | 51558_sort_peaks.narrowPeak.bed.bed | Cistrome |
| HESC | SMAD1 | beta-Hairpin exposed by an alpha/beta-scaffold | HUES64 | GSM1505741 | 51559_sort_peaks.narrowPeak.bed.bed | Cistrome |
| HESC | SMAD1 | beta-Hairpin exposed by an alpha/beta-scaffold | HUES64 | GSM1505742 | 51560_sort_peaks.narrowPeak.bed.bed | Cistrome |
| HESC | SMAD1 | beta-Hairpin exposed by an alpha/beta-scaffold | HUES64 | GSM1505743 | 51561_sort_peaks.narrowPeak.bed.bed | Cistrome |
| HESC | SMAD1 | beta-Hairpin exposed by an alpha/beta-scaffold | HUES64 | GSM1505744 | 51562_sort_peaks.narrowPeak.bed.bed | Cistrome |
| HESC | SMAD1 | beta-Hairpin exposed by an alpha/beta-scaffold | HUES64 | GSM1505745 | 51563_sort_peaks.narrowPeak.bed.bed | Cistrome |
| HESC | SMAD1 | beta-Hairpin exposed by an alpha/beta-scaffold | HUES64 | GSM1505746 | 51564_sort_peaks.narrowPeak.bed.bed | Cistrome |
| HESC | SMAD4 | beta-Hairpin exposed by an alpha/beta-scaffold | HUES64 | GSM1505751 | 51569_sort_peaks.narrowPeak.bed.bed | Cistrome |
| HESC | SMAD4 | beta-Hairpin exposed by an alpha/beta-scaffold | HUES64 | GSM1505754 | 51572_sort_peaks.narrowPeak.bed.bed | Cistrome |
| HESC | SMAD4 | beta-Hairpin exposed by an alpha/beta-scaffold | HUES64 | GSM1505755 | 51573_sort_peaks.narrowPeak.bed.bed | Cistrome |
| HESC | SMAD4 | beta-Hairpin exposed by an alpha/beta-scaffold | HUES64 | GSM1505756 | 51574_sort_peaks.narrowPeak.bed.bed | Cistrome |
| HESC | SMAD4 | beta-Hairpin exposed by an alpha/beta-scaffold | HUES64 | GSM1505757 | 51575_sort_peaks.narrowPeak.bed.bed | Cistrome |
| HESC | SMAD2 | beta-Hairpin exposed by an alpha/beta-scaffold | WA09 | GSM1266817 | 43302_sort_peaks.narrowPeak.bed.bed | Cistrome |
| HESC | SMAD2 | beta-Hairpin exposed by an alpha/beta-scaffold | WA09 | GSM1266818 | 43303_sort_peaks.narrowPeak.bed.bed | Cistrome |
| HESC | SMAD2 | beta-Hairpin exposed by an alpha/beta-scaffold | H9 | None | GSE29422.SMAD2.ESC_remap2018_hg19__v1_2.bed | Remap |
| HESC | SMAD3 | beta-Hairpin exposed by an alpha/beta-scaffold | H9 | None | GSE29422.SMAD3.ESC_remap2018_hg19__v1_2.bed | Remap |
| HESC | SMAD4 | beta-Hairpin exposed by an alpha/beta-scaffold | H9 | None | GSE29422.SMAD4.ESC_remap2018_hg19__v1_2.bed | Remap |
| HESC | SMAD3 | beta-Hairpin exposed by an alpha/beta-scaffold | H1 | GSM1949005 | GSE75297.SMAD3.ESC_remap2018_hg19__v1_2.bed | Remap |
| HESC | SMAD3 | beta-Hairpin exposed by an alpha/beta-scaffold | H1 | GSM1949007 | GSE75297.SMAD3.ESC_DIFF_D0_remap2018_hg19__v1_2.bed | Remap |
| HESC | SMAD3 | beta-Hairpin exposed by an alpha/beta-scaffold | H1 | GSM1949009 | GSE75297.SMAD3.ESC_DIFF_D1_remap2018_hg19__v1_2.bed | Remap |
| HESC | SMAD3 | beta-Hairpin exposed by an alpha/beta-scaffold | H1 | GSM1949011 | GSE75297.SMAD3.ESC_DIFF_D2_remap2018_hg19__v1_2.bed | Remap |
| HESC | TBP | beta-Sheet binding to DNA | H1 | GSM935303 | 45684_sort_peaks.narrowPeak.bed.bed | ENCODE |
| HESC | POLR2A | ENCODE: TF | H1 | GSM1008649 | 38292_sort_peaks.narrowPeak.bed.bed | Cistrome |
| HESC | TRIM28 | ENCODE: TF | H1 | GSM1399258 | 48923_sort_peaks.narrowPeak.bed.bed | Cistrome |
| HESC | TRIM28 | ENCODE: TF | H1 | GSM1399265 | 48930_sort_peaks.narrowPeak.bed.bed | Cistrome |
| HESC | POLR2A | ENCODE: TF | H9 | GSM1466841 | 49547_sort_peaks.narrowPeak.bed.bed | Cistrome |
| HESC | POLR2A | ENCODE: TF | H9 | GSM1466842 | 49548_sort_peaks.narrowPeak.bed.bed | Cistrome |
| HESC | TRIM28 | ENCODE: TF | Embryonic_stem_cells | GSM1467734 | 49606_sort_peaks.narrowPeak.bed.bed | Cistrome |
| HESC | TRIM28 | ENCODE: TF | Embryonic_stem_cells | GSM1467736 | 49608_sort_peaks.narrowPeak.bed.bed | Cistrome |
| HESC | TRIM28 | ENCODE: TF | Embryonic_stem_cells | GSM1467737 | 49609_sort_peaks.narrowPeak.bed.bed | Cistrome |
| HESC | POLR2A | ENCODE: TF | H1 | GSM1503827 | 49743_sort_peaks.narrowPeak.bed.bed | Cistrome |
| HESC | POLR2A | ENCODE: TF | H1 | GSM1579351 | 53658_sort_peaks.narrowPeak.bed.bed | Cistrome |
| HESC | POLR2A | ENCODE: TF | H1 | GSM1579352 | 53659_sort_peaks.narrowPeak.bed.bed | Cistrome |
| HESC | POLR2A | ENCODE: TF | H1 | GSM1579353 | 53660_sort_peaks.narrowPeak.bed.bed | Cistrome |
| HESC | POLR2A | ENCODE: TF | H1 | GSM1579354 | 53661_sort_peaks.narrowPeak.bed.bed | Cistrome |
| HESC | POLR2A | ENCODE: TF | H1 | GSM1579355 | 53662_sort_peaks.narrowPeak.bed.bed | Cistrome |
| HESC | POLR2A | ENCODE: TF | H1 | GSM1579356 | 53663_sort_peaks.narrowPeak.bed.bed | Cistrome |
| HESC | POLR2A | ENCODE: TF | H1 | GSM1579357 | 53664_sort_peaks.narrowPeak.bed.bed | Cistrome |
| HESC | POLR2A | ENCODE: TF | H1 | GSM1579358 | 53665_sort_peaks.narrowPeak.bed.bed | Cistrome |
| HESC | POLR2A | ENCODE: TF | H1 | GSM1579359 | 53666_sort_peaks.narrowPeak.bed.bed | Cistrome |
| HESC | POLR2A | ENCODE: TF | H1 | GSM1579360 | 53667_sort_peaks.narrowPeak.bed.bed | Cistrome |
| HESC | POLR2A | ENCODE: TF | H1 | GSM1579361 | 53668_sort_peaks.narrowPeak.bed.bed | Cistrome |
| HESC | POLR2A | ENCODE: TF | H1 | GSM1579362 | 53669_sort_peaks.narrowPeak.bed.bed | Cistrome |
| HESC | EP300 | ENCODE: TF | H9 | GSM602291 | 1007_sort_peaks.narrowPeak.bed.bed | Cistrome |
| HESC | POLR2A | ENCODE: TF | H1 | GSM748532 | 8097_sort_peaks.narrowPeak.bed.bed | Cistrome |
| HESC | GTF2F1 | ENCODE: TF | H1 | GSM935581 | 45683_sort_peaks.narrowPeak.bed.bed | Cistrome |
| HESC | TRIM28 | ENCODE: TF | H1 | GSM2067350 | 73982_sort_peaks.narrowPeak.bed.bed | Cistrome |
| HESC | SMC3 | ENCODE: TF | BG03 | GSM897119 | 34011_sort_peaks.narrowPeak.bed.bed | Cistrome |
| HESC | POLR2A | ENCODE: TF | BG03 | GSM897115 | 34013_sort_peaks.narrowPeak.bed.bed | Cistrome |
| HESC | POLR2A | ENCODE: TF | BG03 | GSM937544 | 34073_sort_peaks.narrowPeak.bed.bed | Cistrome |
| HESC | POLR2A | ENCODE: TF | BG03 | GSM937545 | 34079_sort_peaks.narrowPeak.bed.bed | Cistrome |
| HESC | POLR2A | ENCODE: TF | ES | GSM535981 | 40284_sort_peaks.narrowPeak.bed.bed | Cistrome |
| HESC | POLR2A | ENCODE: TF | HUES64 | GSM1505716 | 51534_sort_peaks.narrowPeak.bed.bed | Cistrome |
| HESC | POLR2A | ENCODE: TF | HUES64 | GSM1505717 | 51535_sort_peaks.narrowPeak.bed.bed | Cistrome |
| HESC | POLR2A | ENCODE: TF | HUES64 | GSM1505718 | 51536_sort_peaks.narrowPeak.bed.bed | Cistrome |
| HESC | POLR2A | ENCODE: TF | HUES64 | GSM1505719 | 51537_sort_peaks.narrowPeak.bed.bed | Cistrome |
| HESC | POLR2A | ENCODE: TF | HUES64 | GSM1505720 | 51538_sort_peaks.narrowPeak.bed.bed | Cistrome |
| HESC | POLR2A | ENCODE: TF | HUES64 | GSM1505721 | 51539_sort_peaks.narrowPeak.bed.bed | Cistrome |
| HESC | TRIM28 | ENCODE: TF | HUES64 | GSM1505798 | 51616_sort_peaks.narrowPeak.bed.bed | Cistrome |
| HESC | TRIM28 | ENCODE: TF | HUES64 | GSM1505799 | 51617_sort_peaks.narrowPeak.bed.bed | Cistrome |
| HESC | TRIM28 | ENCODE: TF | HUES64 | GSM1505800 | 51618_sort_peaks.narrowPeak.bed.bed | Cistrome |
| HESC | POLR2A | ENCODE: TF | iPS | GSM2183737 | 86939_sort_peaks.narrowPeak.bed.bed | Cistrome |
| HESC | POLR2A | ENCODE: TF | iPS | GSM2183739 | 86944_sort_peaks.narrowPeak.bed.bed | Cistrome |
| HESC | POLR2A | ENCODE: TF | iPS | GSM2183738 | 86945_sort_peaks.narrowPeak.bed.bed | Cistrome |
| HESC | SMC3 | ENCODE: TF | H1 | GSM560344 | 2898_sort_peaks.narrowPeak.bed.bed | Cistrome |
| HESC | SMC3 | ENCODE: TF | H1 | GSM560343 | 2899_sort_peaks.narrowPeak.bed.bed | Cistrome |
| HESC | TRIM28 | ENCODE: TF | WIBR3 | GSM2232854 | 70221_sort_peaks.narrowPeak.bed.bed | Cistrome |
| HESC | TRIM28 | ENCODE: TF | WIBR3 | GSM2232853 | 70222_sort_peaks.narrowPeak.bed.bed | Cistrome |
| HESC | CHD1 | ENCODE: TF | H1 | GSM1003444 | 45237_sort_peaks.narrowPeak.bed.bed | ENCODE |
| HESC | EP300 | ENCODE: TF | H1 | GSM1003513 | 45242_sort_peaks.narrowPeak.bed.bed | ENCODE |
| HESC | SAP30 | ENCODE: TF | H1 | GSM1003572 | 45234_sort_peaks.narrowPeak.bed.bed | ENCODE |
| HESC | CHD1 | ENCODE: TF | H1 | GSM935296 | 45686_sort_peaks.narrowPeak.bed.bed | ENCODE |
| HESC | CHD2 | ENCODE: TF | H1 | GSM935297 | 45701_sort_peaks.narrowPeak.bed.bed | ENCODE |
| HESC | POLR2A | ENCODE: TF | H1 | GSM1010803 | 46199_sort_peaks.narrowPeak.bed.bed | ENCODE |
| HESC | POLR2A | ENCODE: TF | H1 | GSM803366 | 46187_sort_peaks.narrowPeak.bed.bed | ENCODE |
| HESC | POLR2A | ENCODE: TF | H1 | GSM822300 | 35269_sort_peaks.narrowPeak.bed.bed | ENCODE |
| HESC | POLR2A | ENCODE: TF | H1 | GSM803484 | 46168_sort_peaks.narrowPeak.bed.bed | ENCODE |
| HESC | EP300 | ENCODE: TF | H1 | GSM803542 | 46165_sort_peaks.narrowPeak.bed.bed | ENCODE |
| HESC | CHD1 | ENCODE: TF | H1 | GSM831025 | 8806_sort_peaks.narrowPeak.bed.bed | ENCODE |
| HESC | CHD1 | ENCODE: TF | H1 | GSM831026 | 8807_sort_peaks.narrowPeak.bed.bed | ENCODE |
| HESC | EP300 | ENCODE: TF | H1 | GSM831036 | 8817_sort_peaks.narrowPeak.bed.bed | ENCODE |
| HESC | SAP30 | ENCODE: TF | H1 | GSM831040 | 8821_sort_peaks.narrowPeak.bed.bed | ENCODE |
| HESC | EP300 | ENCODE: TF | H1 | GSM447587 | GSE17917.EP300.ESC_remap2018_hg19__v1_2.bed | Remap |
| HESC | NKX2-5 | Helix-turn-helix domains | Embryonic_stem_cells | GSM2372592 | SRX2321774.bed | ChIP-Atlas |
| HESC | NKX2-5 | Helix-turn-helix domains | Embryonic_stem_cells | GSM2372593 | SRX2321775.bed | ChIP-Atlas |
| HESC | NKX2-5 | Helix-turn-helix domains | Embryonic_stem_cells | GSM2372594 | SRX2321776.bed | ChIP-Atlas |
| HESC | PAX7 | Helix-turn-helix domains | H9 | GSM2629527 | SRX2830156.bed | ChIP-Atlas |
| HESC | TEAD4 | Helix-turn-helix domains | H1 | GSM2635693 | SRX2844313.bed | ChIP-Atlas |
| HESC | NANOG | Helix-turn-helix domains | H9 | GSM2648931 | SRX2881137.bed | ChIP-Atlas |
| HESC | NANOG | Helix-turn-helix domains | H9 | GSM2648932 | SRX2881138.bed | ChIP-Atlas |
| HESC | POU5F1 | Helix-turn-helix domains | H9 | GSM2648933 | SRX2881139.bed | ChIP-Atlas |
| HESC | POU5F1 | Helix-turn-helix domains | H9 | GSM2648934 | SRX2881140.bed | ChIP-Atlas |
| HESC | PDX1 | Helix-turn-helix domains | CyT49 | GSM1316337 | 52147_sort_peaks.narrowPeak.bed.bed | Cistrome |
| HESC | FOXA1 | Helix-turn-helix domains | CyT49 | GSM1551464 | 52677_sort_peaks.narrowPeak.bed.bed | Cistrome |
| HESC | FOXA1 | Helix-turn-helix domains | CyT49 | GSM1551465 | 52678_sort_peaks.narrowPeak.bed.bed | Cistrome |
| HESC | FOXA1 | Helix-turn-helix domains | CyT49 | GSM1551466 | 52679_sort_peaks.narrowPeak.bed.bed | Cistrome |
| HESC | FOXA1 | Helix-turn-helix domains | CyT49 | GSM1551467 | 52680_sort_peaks.narrowPeak.bed.bed | Cistrome |
| HESC | FOXA2 | Helix-turn-helix domains | CyT49 | GSM1551468 | 52681_sort_peaks.narrowPeak.bed.bed | Cistrome |
| HESC | FOXA2 | Helix-turn-helix domains | CyT49 | GSM1551469 | 52682_sort_peaks.narrowPeak.bed.bed | Cistrome |
| HESC | FOXA2 | Helix-turn-helix domains | CyT49 | GSM1551470 | 52683_sort_peaks.narrowPeak.bed.bed | Cistrome |
| HESC | FOXA2 | Helix-turn-helix domains | CyT49 | GSM1551471 | 52684_sort_peaks.narrowPeak.bed.bed | Cistrome |
| HESC | NANOG | Helix-turn-helix domains | H9 | GSM1901445 | 57188_sort_peaks.narrowPeak.bed.bed | Cistrome |
| HESC | POU5F1 | Helix-turn-helix domains | H1 | GSM447582 | 4924_sort_peaks.narrowPeak.bed.bed | Cistrome |
| HESC | POU5F1 | Helix-turn-helix domains | H1 | GSM447583 | 4925_sort_peaks.narrowPeak.bed.bed | Cistrome |
| HESC | NANOG | Helix-turn-helix domains | H1 | GSM518374 | 955_sort_peaks.narrowPeak.bed.bed | Cistrome |
| HESC | POU5F1 | Helix-turn-helix domains | H9 | GSM545202 | 5753_sort_peaks.narrowPeak.bed.bed | Cistrome |
| HESC | POU5F1 | Helix-turn-helix domains | H9 | GSM545203 | 5754_sort_peaks.narrowPeak.bed.bed | Cistrome |
| HESC | POU5F1 | Helix-turn-helix domains | H9 | GSM545204 | 5755_sort_peaks.narrowPeak.bed.bed | Cistrome |
| HESC | FOXH1 | Helix-turn-helix domains | H9 | GSM727564 | 1011_sort_peaks.narrowPeak.bed.bed | Cistrome |
| HESC | FOXH1 | Helix-turn-helix domains | H9 | GSM727589 | 1010_sort_peaks.narrowPeak.bed.bed | Cistrome |
| HESC | RFX5 | Helix-turn-helix domains | H1 | GSM935382 | 45692_sort_peaks.narrowPeak.bed.bed | Cistrome |
| HESC | SPI1 | Helix-turn-helix domains | BG01 | GSM1272760 | 43342_sort_peaks.narrowPeak.bed.bed | Cistrome |
| HESC | NANOG | Helix-turn-helix domains | HUES64 | GSM1124071 | 34089_sort_peaks.narrowPeak.bed.bed | Cistrome |
| HESC | POU5F1 | Helix-turn-helix domains | HUES64 | GSM1124067 | 34100_sort_peaks.narrowPeak.bed.bed | Cistrome |
| HESC | NANOG | Helix-turn-helix domains | HUES64 | GSM1124070 | 34102_sort_peaks.narrowPeak.bed.bed | Cistrome |
| HESC | CDX2 | Helix-turn-helix domains | HUES64 | GSM1505619 | 51437_sort_peaks.narrowPeak.bed.bed | Cistrome |
| HESC | FOXA1 | Helix-turn-helix domains | HUES64 | GSM1505632 | 51450_sort_peaks.narrowPeak.bed.bed | Cistrome |
| HESC | FOXA1 | Helix-turn-helix domains | HUES64 | GSM1505633 | 51451_sort_peaks.narrowPeak.bed.bed | Cistrome |
| HESC | FOXA1 | Helix-turn-helix domains | HUES64 | GSM1505635 | 51453_sort_peaks.narrowPeak.bed.bed | Cistrome |
| HESC | FOXA2 | Helix-turn-helix domains | HUES64 | GSM1505637 | 51455_sort_peaks.narrowPeak.bed.bed | Cistrome |
| HESC | FOXA2 | Helix-turn-helix domains | HUES64 | GSM1505639 | 51457_sort_peaks.narrowPeak.bed.bed | Cistrome |
| HESC | FOXA2 | Helix-turn-helix domains | HUES64 | GSM1505640 | 51458_sort_peaks.narrowPeak.bed.bed | Cistrome |
| HESC | FOXA2 | Helix-turn-helix domains | HUES64 | GSM1505641 | 51459_sort_peaks.narrowPeak.bed.bed | Cistrome |
| HESC | HNF1B | Helix-turn-helix domains | HUES64 | GSM1505680 | 51498_sort_peaks.narrowPeak.bed.bed | Cistrome |
| HESC | HNF1B | Helix-turn-helix domains | HUES64 | GSM1505681 | 51499_sort_peaks.narrowPeak.bed.bed | Cistrome |
| HESC | HNF1B | Helix-turn-helix domains | HUES64 | GSM1505682 | 51500_sort_peaks.narrowPeak.bed.bed | Cistrome |
| HESC | HNF1B | Helix-turn-helix domains | HUES64 | GSM1505683 | 51501_sort_peaks.narrowPeak.bed.bed | Cistrome |
| HESC | NANOG | Helix-turn-helix domains | HUES64 | GSM1505695 | 51513_sort_peaks.narrowPeak.bed.bed | Cistrome |
| HESC | NANOG | Helix-turn-helix domains | HUES64 | GSM1505696 | 51514_sort_peaks.narrowPeak.bed.bed | Cistrome |
| HESC | NANOG | Helix-turn-helix domains | HUES64 | GSM1505698 | 51516_sort_peaks.narrowPeak.bed.bed | Cistrome |
| HESC | NANOG | Helix-turn-helix domains | HUES64 | GSM1505700 | 51518_sort_peaks.narrowPeak.bed.bed | Cistrome |
| HESC | NANOG | Helix-turn-helix domains | HUES64 | GSM1505702 | 51520_sort_peaks.narrowPeak.bed.bed | Cistrome |
| HESC | NANOG | Helix-turn-helix domains | HUES64 | GSM1505703 | 51521_sort_peaks.narrowPeak.bed.bed | Cistrome |
| HESC | OTX2 | Helix-turn-helix domains | HUES64 | GSM1505704 | 51522_sort_peaks.narrowPeak.bed.bed | Cistrome |
| HESC | OTX2 | Helix-turn-helix domains | HUES64 | GSM1505706 | 51524_sort_peaks.narrowPeak.bed.bed | Cistrome |
| HESC | OTX2 | Helix-turn-helix domains | HUES64 | GSM1505707 | 51525_sort_peaks.narrowPeak.bed.bed | Cistrome |
| HESC | OTX2 | Helix-turn-helix domains | HUES64 | GSM1505709 | 51527_sort_peaks.narrowPeak.bed.bed | Cistrome |
| HESC | OTX2 | Helix-turn-helix domains | HUES64 | GSM1505711 | 51529_sort_peaks.narrowPeak.bed.bed | Cistrome |
| HESC | OTX2 | Helix-turn-helix domains | HUES64 | GSM1505712 | 51530_sort_peaks.narrowPeak.bed.bed | Cistrome |
| HESC | OTX2 | Helix-turn-helix domains | HUES64 | GSM1505713 | 51531_sort_peaks.narrowPeak.bed.bed | Cistrome |
| HESC | PAX6 | Helix-turn-helix domains | HUES64 | GSM1505714 | 51532_sort_peaks.narrowPeak.bed.bed | Cistrome |
| HESC | POU5F1 | Helix-turn-helix domains | HUES64 | GSM1505724 | 51542_sort_peaks.narrowPeak.bed.bed | Cistrome |
| HESC | POU5F1 | Helix-turn-helix domains | HUES64 | GSM1505725 | 51543_sort_peaks.narrowPeak.bed.bed | Cistrome |
| HESC | POU5F1 | Helix-turn-helix domains | HUES64 | GSM1505726 | 51544_sort_peaks.narrowPeak.bed.bed | Cistrome |
| HESC | POU5F1 | Helix-turn-helix domains | HUES64 | GSM1505727 | 51545_sort_peaks.narrowPeak.bed.bed | Cistrome |
| HESC | POU5F1 | Helix-turn-helix domains | HUES64 | GSM1505728 | 51546_sort_peaks.narrowPeak.bed.bed | Cistrome |
| HESC | POU5F1 | Helix-turn-helix domains | iPS | GSM2183773 | 86395_sort_peaks.narrowPeak.bed.bed | Cistrome |
| HESC | POU5F1 | Helix-turn-helix domains | iPS | GSM2183774 | 86398_sort_peaks.narrowPeak.bed.bed | Cistrome |
| HESC | SPI1 | Helix-turn-helix domains | iPS-C1 | GSM1272748 | 43330_sort_peaks.narrowPeak.bed.bed | Cistrome |
| HESC | SPI1 | Helix-turn-helix domains | iPS-C1 | GSM1272754 | 43336_sort_peaks.narrowPeak.bed.bed | Cistrome |
| HESC | FOXM1 | Helix-turn-helix domains | VAL-3 | GSM2101199 | 68837_sort_peaks.narrowPeak.bed.bed | Cistrome |
| HESC | TEAD4 | Helix-turn-helix domains | WA09 | GSM1266815 | 43300_sort_peaks.narrowPeak.bed.bed | Cistrome |
| HESC | TEAD4 | Helix-turn-helix domains | WA09 | GSM1266816 | 43301_sort_peaks.narrowPeak.bed.bed | Cistrome |
| HESC | SPI1 | Helix-turn-helix domains | WIBR3 | GSM1272766 | 43348_sort_peaks.narrowPeak.bed.bed | Cistrome |
| HESC | SPI1 | Helix-turn-helix domains | WIBR3 | GSM1272772 | 43354_sort_peaks.narrowPeak.bed.bed | Cistrome |
| HESC | SPI1 | Helix-turn-helix domains | WIS2 | GSM1571912 | 50918_sort_peaks.narrowPeak.bed.bed | Cistrome |
| HESC | SPI1 | Helix-turn-helix domains | WIS2 | GSM1571918 | 50924_sort_peaks.narrowPeak.bed.bed | Cistrome |
| HESC | KDM5A | Helix-turn-helix domains | H1 | GSM1003446 | 45244_sort_peaks.narrowPeak.bed.bed | ENCODE |
| HESC | KDM5B | Helix-turn-helix domains | H1 | GSM1003457 | 45238_sort_peaks.narrowPeak.bed.bed | ENCODE |
| HESC | TEAD4 | Helix-turn-helix domains | H1 | GSM1010845 | 46189_sort_peaks.narrowPeak.bed.bed | ENCODE |
| HESC | E2F6 | Helix-turn-helix domains | H1 | GSM1010899 | 46177_sort_peaks.narrowPeak.bed.bed | ENCODE |
| HESC | SIX5 | Helix-turn-helix domains | H1 | GSM803405 | 46188_sort_peaks.narrowPeak.bed.bed | ENCODE |
| HESC | GABPA | Helix-turn-helix domains | H1 | GSM803424 | 46185_sort_peaks.narrowPeak.bed.bed | ENCODE |
| HESC | NANOG | Helix-turn-helix domains | H1 | GSM803437 | 46173_sort_peaks.narrowPeak.bed.bed | ENCODE |
| HESC | POU5F1 | Helix-turn-helix domains | H1 | GSM803438 | 46194_sort_peaks.narrowPeak.bed.bed | ENCODE |
| HESC | KDM5A | Helix-turn-helix domains | H1 | GSM831031 | 8812_sort_peaks.narrowPeak.bed.bed | ENCODE |
| HESC | KDM5A | Helix-turn-helix domains | H1 | GSM831032 | 8813_sort_peaks.narrowPeak.bed.bed | ENCODE |
| HESC | KDM5B | Helix-turn-helix domains | H1 | GSM831033 | 8814_sort_peaks.narrowPeak.bed.bed | ENCODE |
| HESC | KDM5C | Helix-turn-helix domains | H1 | GSM831034 | 8815_sort_peaks.narrowPeak.bed.bed | ENCODE |
| HESC | NANOG | Helix-turn-helix domains | H1 | None | GSE18292.NANOG.ESC_remap2018_hg19__v1_2.bed | Remap |
| HESC | POU5F1 | Helix-turn-helix domains | H1 | GSM518373 | GSE20650.POU5F1.ESC_remap2018_hg19__v1_2.bed | Remap |
| HESC | FOXP1 | Helix-turn-helix domains | H9 | None | GSE31006.FOXP1.ESC_H9_remap2018_hg19__v1_2.bed | Remap |
| HESC | T | Immunoglobulin fold | H9 | GSM1483192 | 53339_sort_peaks.narrowPeak.bed.bed | Cistrome |
| HESC | T | Immunoglobulin fold | H9 | GSM1483193 | 53340_sort_peaks.narrowPeak.bed.bed | Cistrome |
| HESC | STAT5B | Immunoglobulin fold | H9 | GSM1577752 | 53635_sort_peaks.narrowPeak.bed.bed | Cistrome |
| HESC | EOMES | Immunoglobulin fold | HUES64 | GSM1505627 | 51445_sort_peaks.narrowPeak.bed.bed | Cistrome |
| HESC | EOMES | Immunoglobulin fold | HUES64 | GSM1505629 | 51447_sort_peaks.narrowPeak.bed.bed | Cistrome |
| HESC | EOMES | Immunoglobulin fold | HUES64 | GSM1505630 | 51448_sort_peaks.narrowPeak.bed.bed | Cistrome |
| HESC | EOMES | Immunoglobulin fold | HUES64 | GSM1505631 | 51449_sort_peaks.narrowPeak.bed.bed | Cistrome |
| HESC | STAT3 | Immunoglobulin fold | HUES64 | GSM1505779 | 51597_sort_peaks.narrowPeak.bed.bed | Cistrome |
| HESC | STAT3 | Immunoglobulin fold | HUES64 | GSM1505780 | 51598_sort_peaks.narrowPeak.bed.bed | Cistrome |
| HESC | STAT3 | Immunoglobulin fold | HUES64 | GSM1505781 | 51599_sort_peaks.narrowPeak.bed.bed | Cistrome |
| HESC | T | Immunoglobulin fold | HUES64 | GSM1505782 | 51600_sort_peaks.narrowPeak.bed.bed | Cistrome |
| HESC | T | Immunoglobulin fold | HUES64 | GSM1505784 | 51602_sort_peaks.narrowPeak.bed.bed | Cistrome |
| HESC | EOMES | Immunoglobulin fold | H9 | GSM640691 | GSE26097.EOMES.ESC_remap2018_hg19__v1_2.bed | Remap |
| HESC | TP53 | Immunoglobulin fold | H9 | None | GSE39912.TP53.ESC_H9_remap2018_hg19__v1_2.bed | Remap |
| HESC | TCF7L1 | Other all-alpha-helical DNA-binding domains | H9 | GSM2124632 | SRX1707570.bed | ChIP-Atlas |
| HESC | TCF7L1 | Other all-alpha-helical DNA-binding domains | H9 | GSM2124633 | SRX1707571.bed | ChIP-Atlas |
| HESC | TCF7L1 | Other all-alpha-helical DNA-binding domains | H9 | GSM2124634 | SRX1707572.bed | ChIP-Atlas |
| HESC | SOX9 | Other all-alpha-helical DNA-binding domains | CyT49 | GSM1517602 | 54616_sort_peaks.narrowPeak.bed.bed | Cistrome |
| HESC | LEF1 | Other all-alpha-helical DNA-binding domains | H1 | GSM1579343 | 53650_sort_peaks.narrowPeak.bed.bed | Cistrome |
| HESC | LEF1 | Other all-alpha-helical DNA-binding domains | H1 | GSM1579344 | 53651_sort_peaks.narrowPeak.bed.bed | Cistrome |
| HESC | SOX2 | Other all-alpha-helical DNA-binding domains | HUES64 | GSM1124068 | 34092_sort_peaks.narrowPeak.bed.bed | Cistrome |
| HESC | SOX2 | Other all-alpha-helical DNA-binding domains | HUES64 | GSM1124069 | 34118_sort_peaks.narrowPeak.bed.bed | Cistrome |
| HESC | SOX17 | Other all-alpha-helical DNA-binding domains | HUES64 | GSM1505760 | 51578_sort_peaks.narrowPeak.bed.bed | Cistrome |
| HESC | SOX17 | Other all-alpha-helical DNA-binding domains | HUES64 | GSM1505762 | 51580_sort_peaks.narrowPeak.bed.bed | Cistrome |
| HESC | SOX17 | Other all-alpha-helical DNA-binding domains | HUES64 | GSM1505763 | 51581_sort_peaks.narrowPeak.bed.bed | Cistrome |
| HESC | SOX2 | Other all-alpha-helical DNA-binding domains | HUES64 | GSM1505764 | 51582_sort_peaks.narrowPeak.bed.bed | Cistrome |
| HESC | SOX2 | Other all-alpha-helical DNA-binding domains | HUES64 | GSM1505766 | 51584_sort_peaks.narrowPeak.bed.bed | Cistrome |
| HESC | SOX2 | Other all-alpha-helical DNA-binding domains | HUES64 | GSM1505767 | 51585_sort_peaks.narrowPeak.bed.bed | Cistrome |
| HESC | SOX2 | Other all-alpha-helical DNA-binding domains | HUES64 | GSM1505768 | 51586_sort_peaks.narrowPeak.bed.bed | Cistrome |
| HESC | SOX2 | Other all-alpha-helical DNA-binding domains | iPS | GSM2183781 | 84257_sort_peaks.narrowPeak.bed.bed | Cistrome |
| HESC | SOX2 | Other all-alpha-helical DNA-binding domains | iPS | GSM2183782 | 84258_sort_peaks.narrowPeak.bed.bed | Cistrome |
| HESC | TAF1 | Other all-alpha-helical DNA-binding domains | H1 | GSM1010805 | 46197_sort_peaks.narrowPeak.bed.bed | ENCODE |
| HESC | TAF1 | Other all-alpha-helical DNA-binding domains | H1 | GSM803450 | 46196_sort_peaks.narrowPeak.bed.bed | ENCODE |
| HESC | SOX2 | Other all-alpha-helical DNA-binding domains | H9 | GSM1973976 | EXP037175_liftoverhg19.bed | GTRD |
| HESC | SOX2 | Other all-alpha-helical DNA-binding domains | H1 | GSM456570 | GSE18292.SOX2.ESC_remap2018_hg19__v1_2.bed | Remap |
| HESC | SOX2 | Other all-alpha-helical DNA-binding domains | H9 | GSM1139041 | GSE46837.SOX2.ESC_H9_remap2018_hg19__v1_2.bed | Remap |
| HESC | SOX2 | Other all-alpha-helical DNA-binding domains | H9 | GSM1701825 | GSE69479.SOX2.ESC_remap2018_hg19__v1_2.bed | Remap |
| HESC | UBTF | Other all-alpha-helical DNA-binding domains | H9 | GSM2028274 | GSE76586.UBTF.ESC_remap2018_hg19__v1_2.bed | Remap |
| HESC | UBTF | Other all-alpha-helical DNA-binding domains | H9 | GSM2028275 | GSE76586.UBTF.ESC_ACTIVINA_remap2018_hg19__v1_2.bed | Remap |
| HESC | SMARCA4 | TcoF: TF | H9 | GSM602297 | 5227_sort_peaks.narrowPeak.bed.bed | Cistrome |
| HESC | BRCA1 | TcoF: TF | H1 | GSM935517 | 45698_sort_peaks.narrowPeak.bed.bed | Cistrome |
| HESC | EZH2 | TcoF: TF | H9 | GSM2029369 | 73191_sort_peaks.narrowPeak.bed.bed | Cistrome |
| HESC | ZNF483 | TcoF: TF | BG01V | GSM2563969 | 81217_sort_peaks.narrowPeak.bed.bed | Cistrome |
| HESC | ZNF483 | TcoF: TF | BG01V | GSM2563965 | 81222_sort_peaks.narrowPeak.bed.bed | Cistrome |
| HESC | ZNF483 | TcoF: TF | BG01V | GSM2563981 | 83048_sort_peaks.narrowPeak.bed.bed | Cistrome |
| HESC | ZNF483 | TcoF: TF | BG01V | GSM2563977 | 85327_sort_peaks.narrowPeak.bed.bed | Cistrome |
| HESC | HAND2 | TcoF: TF | HUES64 | GSM1505811 | 51629_sort_peaks.narrowPeak.bed.bed | Cistrome |
| HESC | HAND1 | TcoF: TF | HUES64 | GSM1505813 | 51631_sort_peaks.narrowPeak.bed.bed | Cistrome |
| HESC | HAND1 | TcoF: TF | HUES64 | GSM1505814 | 51632_sort_peaks.narrowPeak.bed.bed | Cistrome |
| HESC | EZH2 | TcoF: TF | H1 | GSM1003524 | 45229_sort_peaks.narrowPeak.bed.bed | ENCODE |
| HESC | EZH2 | TcoF: TF | H1 | GSM831028 | 8809_sort_peaks.narrowPeak.bed.bed | ENCODE |
| HESC | EZH2 | TcoF: TF | H9 | GSM327665 | GSE13084.EZH2.ESC_remap2018_hg19__v1_2.bed | Remap |
| HESC | CTCF | Zinc-coordinating DNA-binding domains | Embryonic_stem_cells | GSM1939276 | SRX1430078.bed | ChIP-Atlas |
| HESC | CTCF | Zinc-coordinating DNA-binding domains | H1 | GSM1267206 | 50987_sort_peaks.narrowPeak.bed.bed | Cistrome |
| HESC | CTCF | Zinc-coordinating DNA-binding domains | H1 | GSM1267207 | 50988_sort_peaks.narrowPeak.bed.bed | Cistrome |
| HESC | CTCF | Zinc-coordinating DNA-binding domains | H1 | GSM1267208 | 50989_sort_peaks.narrowPeak.bed.bed | Cistrome |
| HESC | CTCF | Zinc-coordinating DNA-binding domains | H1 | GSM1267209 | 50990_sort_peaks.narrowPeak.bed.bed | Cistrome |
| HESC | CTCF | Zinc-coordinating DNA-binding domains | H1 | GSM1267210 | 50991_sort_peaks.narrowPeak.bed.bed | Cistrome |
| HESC | CTCF | Zinc-coordinating DNA-binding domains | H1 | GSM518375 | 832_sort_peaks.narrowPeak.bed.bed | Cistrome |
| HESC | PRDM14 | Zinc-coordinating DNA-binding domains | H1 | GSM563088 | 966_sort_peaks.narrowPeak.bed.bed | Cistrome |
| HESC | ZNF143 | Zinc-coordinating DNA-binding domains | H1 | GSM935514 | 45688_sort_peaks.narrowPeak.bed.bed | Cistrome |
| HESC | GATA3 | Zinc-coordinating DNA-binding domains | H9 | GSM2817659 | 82055_sort_peaks.narrowPeak.bed.bed | Cistrome |
| HESC | ZNF114 | Zinc-coordinating DNA-binding domains | BG01V | GSM2563968 | 81216_sort_peaks.narrowPeak.bed.bed | Cistrome |
| HESC | ZNF57 | Zinc-coordinating DNA-binding domains | BG01V | GSM2563963 | 81218_sort_peaks.narrowPeak.bed.bed | Cistrome |
| HESC | ZNF589 | Zinc-coordinating DNA-binding domains | BG01V | GSM2563966 | 81219_sort_peaks.narrowPeak.bed.bed | Cistrome |
| HESC | ZNF57 | Zinc-coordinating DNA-binding domains | BG01V | GSM2563967 | 81220_sort_peaks.narrowPeak.bed.bed | Cistrome |
| HESC | ZNF114 | Zinc-coordinating DNA-binding domains | BG01V | GSM2563964 | 81221_sort_peaks.narrowPeak.bed.bed | Cistrome |
| HESC | ZNF114 | Zinc-coordinating DNA-binding domains | BG01V | GSM2563980 | 83047_sort_peaks.narrowPeak.bed.bed | Cistrome |
| HESC | ZNF589 | Zinc-coordinating DNA-binding domains | BG01V | GSM2563982 | 83049_sort_peaks.narrowPeak.bed.bed | Cistrome |
| HESC | ZNF114 | Zinc-coordinating DNA-binding domains | BG01V | GSM2563985 | 83050_sort_peaks.narrowPeak.bed.bed | Cistrome |
| HESC | ZNF589 | Zinc-coordinating DNA-binding domains | BG01V | GSM2563986 | 83051_sort_peaks.narrowPeak.bed.bed | Cistrome |
| HESC | ZNF589 | Zinc-coordinating DNA-binding domains | BG01V | GSM2563978 | 85319_sort_peaks.narrowPeak.bed.bed | Cistrome |
| HESC | ZNF589 | Zinc-coordinating DNA-binding domains | BG01V | GSM2563970 | 85323_sort_peaks.narrowPeak.bed.bed | Cistrome |
| HESC | ZNF114 | Zinc-coordinating DNA-binding domains | BG01V | GSM2563973 | 85324_sort_peaks.narrowPeak.bed.bed | Cistrome |
| HESC | ZNF57 | Zinc-coordinating DNA-binding domains | BG01V | GSM2563972 | 85325_sort_peaks.narrowPeak.bed.bed | Cistrome |
| HESC | ZNF589 | Zinc-coordinating DNA-binding domains | BG01V | GSM2563974 | 85326_sort_peaks.narrowPeak.bed.bed | Cistrome |
| HESC | ZNF114 | Zinc-coordinating DNA-binding domains | BG01V | GSM2563976 | 85328_sort_peaks.narrowPeak.bed.bed | Cistrome |
| HESC | PRDM1 | Zinc-coordinating DNA-binding domains | HUES64 | GSM1505615 | 51433_sort_peaks.narrowPeak.bed.bed | Cistrome |
| HESC | PRDM1 | Zinc-coordinating DNA-binding domains | HUES64 | GSM1505616 | 51434_sort_peaks.narrowPeak.bed.bed | Cistrome |
| HESC | PRDM1 | Zinc-coordinating DNA-binding domains | HUES64 | GSM1505617 | 51435_sort_peaks.narrowPeak.bed.bed | Cistrome |
| HESC | PRDM1 | Zinc-coordinating DNA-binding domains | HUES64 | GSM1505618 | 51436_sort_peaks.narrowPeak.bed.bed | Cistrome |
| HESC | CTCF | Zinc-coordinating DNA-binding domains | HUES64 | GSM1505620 | 51438_sort_peaks.narrowPeak.bed.bed | Cistrome |
| HESC | CTCF | Zinc-coordinating DNA-binding domains | HUES64 | GSM1505621 | 51439_sort_peaks.narrowPeak.bed.bed | Cistrome |
| HESC | CTCF | Zinc-coordinating DNA-binding domains | HUES64 | GSM1505623 | 51441_sort_peaks.narrowPeak.bed.bed | Cistrome |
| HESC | CTCF | Zinc-coordinating DNA-binding domains | HUES64 | GSM1505624 | 51442_sort_peaks.narrowPeak.bed.bed | Cistrome |
| HESC | CTCF | Zinc-coordinating DNA-binding domains | HUES64 | GSM1505625 | 51443_sort_peaks.narrowPeak.bed.bed | Cistrome |
| HESC | CTCF | Zinc-coordinating DNA-binding domains | HUES64 | GSM1505626 | 51444_sort_peaks.narrowPeak.bed.bed | Cistrome |
| HESC | GATA4 | Zinc-coordinating DNA-binding domains | HUES64 | GSM1505642 | 51460_sort_peaks.narrowPeak.bed.bed | Cistrome |
| HESC | GATA4 | Zinc-coordinating DNA-binding domains | HUES64 | GSM1505645 | 51463_sort_peaks.narrowPeak.bed.bed | Cistrome |
| HESC | GATA4 | Zinc-coordinating DNA-binding domains | HUES64 | GSM1505646 | 51464_sort_peaks.narrowPeak.bed.bed | Cistrome |
| HESC | GATA4 | Zinc-coordinating DNA-binding domains | HUES64 | GSM1505647 | 51465_sort_peaks.narrowPeak.bed.bed | Cistrome |
| HESC | GATA4 | Zinc-coordinating DNA-binding domains | HUES64 | GSM1505648 | 51466_sort_peaks.narrowPeak.bed.bed | Cistrome |
| HESC | GATA4 | Zinc-coordinating DNA-binding domains | HUES64 | GSM1505649 | 51467_sort_peaks.narrowPeak.bed.bed | Cistrome |
| HESC | GATA4 | Zinc-coordinating DNA-binding domains | HUES64 | GSM1505650 | 51468_sort_peaks.narrowPeak.bed.bed | Cistrome |
| HESC | GATA4 | Zinc-coordinating DNA-binding domains | HUES64 | GSM1505651 | 51469_sort_peaks.narrowPeak.bed.bed | Cistrome |
| HESC | GATA4 | Zinc-coordinating DNA-binding domains | HUES64 | GSM1505652 | 51470_sort_peaks.narrowPeak.bed.bed | Cistrome |
| HESC | GATA4 | Zinc-coordinating DNA-binding domains | HUES64 | GSM1505653 | 51471_sort_peaks.narrowPeak.bed.bed | Cistrome |
| HESC | GATA4 | Zinc-coordinating DNA-binding domains | HUES64 | GSM1505654 | 51472_sort_peaks.narrowPeak.bed.bed | Cistrome |
| HESC | GATA4 | Zinc-coordinating DNA-binding domains | HUES64 | GSM1505656 | 51474_sort_peaks.narrowPeak.bed.bed | Cistrome |
| HESC | GATA4 | Zinc-coordinating DNA-binding domains | HUES64 | GSM1505657 | 51475_sort_peaks.narrowPeak.bed.bed | Cistrome |
| HESC | GATA6 | Zinc-coordinating DNA-binding domains | HUES64 | GSM1505658 | 51476_sort_peaks.narrowPeak.bed.bed | Cistrome |
| HESC | GATA6 | Zinc-coordinating DNA-binding domains | HUES64 | GSM1505660 | 51478_sort_peaks.narrowPeak.bed.bed | Cistrome |
| HESC | GATA6 | Zinc-coordinating DNA-binding domains | HUES64 | GSM1505661 | 51479_sort_peaks.narrowPeak.bed.bed | Cistrome |
| HESC | GATA6 | Zinc-coordinating DNA-binding domains | HUES64 | GSM1505662 | 51480_sort_peaks.narrowPeak.bed.bed | Cistrome |
| HESC | GATA6 | Zinc-coordinating DNA-binding domains | HUES64 | GSM1505663 | 51481_sort_peaks.narrowPeak.bed.bed | Cistrome |
| HESC | GATA6 | Zinc-coordinating DNA-binding domains | HUES64 | GSM1505664 | 51482_sort_peaks.narrowPeak.bed.bed | Cistrome |
| HESC | HNF4A | Zinc-coordinating DNA-binding domains | HUES64 | GSM1505686 | 51504_sort_peaks.narrowPeak.bed.bed | Cistrome |
| HESC | KLF5 | Zinc-coordinating DNA-binding domains | HUES64 | GSM1505690 | 51508_sort_peaks.narrowPeak.bed.bed | Cistrome |
| HESC | NR5A2 | Zinc-coordinating DNA-binding domains | HUES64 | GSM1505693 | 51511_sort_peaks.narrowPeak.bed.bed | Cistrome |
| HESC | NR5A2 | Zinc-coordinating DNA-binding domains | HUES64 | GSM1505694 | 51512_sort_peaks.narrowPeak.bed.bed | Cistrome |
| HESC | ZFP42 | Zinc-coordinating DNA-binding domains | HUES64 | GSM1505730 | 51548_sort_peaks.narrowPeak.bed.bed | Cistrome |
| HESC | ZFP42 | Zinc-coordinating DNA-binding domains | HUES64 | GSM1505731 | 51549_sort_peaks.narrowPeak.bed.bed | Cistrome |
| HESC | SALL4 | Zinc-coordinating DNA-binding domains | HUES64 | GSM1505732 | 51550_sort_peaks.narrowPeak.bed.bed | Cistrome |
| HESC | SALL4 | Zinc-coordinating DNA-binding domains | HUES64 | GSM1505733 | 51551_sort_peaks.narrowPeak.bed.bed | Cistrome |
| HESC | SNAI2 | Zinc-coordinating DNA-binding domains | HUES64 | GSM1505758 | 51576_sort_peaks.narrowPeak.bed.bed | Cistrome |
| HESC | SP1 | Zinc-coordinating DNA-binding domains | HUES64 | GSM1505769 | 51587_sort_peaks.narrowPeak.bed.bed | Cistrome |
| HESC | SP1 | Zinc-coordinating DNA-binding domains | HUES64 | GSM1505770 | 51588_sort_peaks.narrowPeak.bed.bed | Cistrome |
| HESC | THAP11 | Zinc-coordinating DNA-binding domains | HUES64 | GSM1505793 | 51611_sort_peaks.narrowPeak.bed.bed | Cistrome |
| HESC | THAP11 | Zinc-coordinating DNA-binding domains | HUES64 | GSM1505794 | 51612_sort_peaks.narrowPeak.bed.bed | Cistrome |
| HESC | THAP11 | Zinc-coordinating DNA-binding domains | HUES64 | GSM1505795 | 51613_sort_peaks.narrowPeak.bed.bed | Cistrome |
| HESC | TET1 | Zinc-coordinating DNA-binding domains | HUES8-hESCs | GSM2642522 | 82036_sort_peaks.narrowPeak.bed.bed | Cistrome |
| HESC | DNMT3B | Zinc-coordinating DNA-binding domains | HUES8-hESCs | GSM2642523 | 82037_sort_peaks.narrowPeak.bed.bed | Cistrome |
| HESC | KLF4 | Zinc-coordinating DNA-binding domains | iPS | GSM2183789 | 84036_sort_peaks.narrowPeak.bed.bed | Cistrome |
| HESC | KLF4 | Zinc-coordinating DNA-binding domains | iPS | GSM2183790 | 88370_sort_peaks.narrowPeak.bed.bed | Cistrome |
| HESC | ZNF274 | Zinc-coordinating DNA-binding domains | H1 | GSM1003619 | 45702_sort_peaks.narrowPeak.bed.bed | ENCODE |
| HESC | CTCF | Zinc-coordinating DNA-binding domains | H1 | GSM624077 | 8086_sort_peaks.narrowPeak.bed.bed | ENCODE |
| HESC | CTCF | Zinc-coordinating DNA-binding domains | H1 | GSM646334 | 36578_sort_peaks.narrowPeak.bed.bed | ENCODE |
| HESC | CTCF | Zinc-coordinating DNA-binding domains | H1 | GSM646335 | 36632_sort_peaks.narrowPeak.bed.bed | ENCODE |
| HESC | CTCF | Zinc-coordinating DNA-binding domains | H1 | GSM733672 | 45230_sort_peaks.narrowPeak.bed.bed | ENCODE |
| HESC | SP4 | Zinc-coordinating DNA-binding domains | H1 | GSM1010743 | 46179_sort_peaks.narrowPeak.bed.bed | ENCODE |
| HESC | SP2 | Zinc-coordinating DNA-binding domains | H1 | GSM1010776 | 46176_sort_peaks.narrowPeak.bed.bed | ENCODE |
| HESC | REST | Zinc-coordinating DNA-binding domains | H1 | GSM1010804 | 46198_sort_peaks.narrowPeak.bed.bed | ENCODE |
| HESC | REST | Zinc-coordinating DNA-binding domains | H1 | GSM803365 | 46172_sort_peaks.narrowPeak.bed.bed | ENCODE |
| HESC | SP1 | Zinc-coordinating DNA-binding domains | H1 | GSM803377 | 46190_sort_peaks.narrowPeak.bed.bed | ENCODE |
| HESC | BCL11A | Zinc-coordinating DNA-binding domains | H1 | GSM803396 | 46178_sort_peaks.narrowPeak.bed.bed | ENCODE |
| HESC | CTCF | Zinc-coordinating DNA-binding domains | H1 | GSM803419 | 46184_sort_peaks.narrowPeak.bed.bed | ENCODE |
| HESC | EGR1 | Zinc-coordinating DNA-binding domains | H1 | GSM803430 | 46169_sort_peaks.narrowPeak.bed.bed | ENCODE |
| HESC | CTCF | Zinc-coordinating DNA-binding domains | H1 | GSM822297 | 35846_sort_peaks.narrowPeak.bed.bed | ENCODE |
| HESC | BCL11A | Zinc-coordinating DNA-binding domains | H1 | GSM803476 | 46186_sort_peaks.narrowPeak.bed.bed | ENCODE |
| HESC | RXRA | Zinc-coordinating DNA-binding domains | H1 | GSM803506 | 46192_sort_peaks.narrowPeak.bed.bed | ENCODE |
| HESC | YY1 | Zinc-coordinating DNA-binding domains | H1 | GSM803513 | 46170_sort_peaks.narrowPeak.bed.bed | ENCODE |
| HESC | KLF4 | Zinc-coordinating DNA-binding domains | H1 | GSM447584 | GSE17917.KLF4.ESC_remap2018_hg19__v1_2.bed | Remap |
| HESC | YY1 | Zinc-coordinating DNA-binding domains | H1 | GSM956127 | GSE39096.YY1.ESC_H1_remap2018_hg19__v1_2.bed | Remap |
| Lung_Cancer | HIF1A | Basic domains | NCI-H1299 | GSM2037452 | SRX1528430.bed | ChIP-Atlas |
| Lung_Cancer | JUN | Basic domains | Calu-3 | GSM2266291 | SRX2010842.bed | ChIP-Atlas |
| Lung_Cancer | JUN | Basic domains | Calu-3 | GSM2266292 | SRX2010843.bed | ChIP-Atlas |
| Lung_Cancer | JUND | Basic domains | Calu-3 | GSM2266293 | SRX2010844.bed | ChIP-Atlas |
| Lung_Cancer | JUND | Basic domains | Calu-3 | GSM2266294 | SRX2010845.bed | ChIP-Atlas |
| Lung_Cancer | MAX | Basic domains | A549 | GSM935298 | 45553_sort_peaks.narrowPeak.bed.bed | Cistrome |
| Lung_Cancer | MYC | Basic domains | A549 | GSM991046 | 50033_sort_peaks.narrowPeak.bed.bed | Cistrome |
| Lung_Cancer | MYC | Basic domains | A549 | GSM991047 | 50034_sort_peaks.narrowPeak.bed.bed | Cistrome |
| Lung_Cancer | MAX | Basic domains | H2171 | GSM894103 | 37173_sort_peaks.narrowPeak.bed.bed | Cistrome |
| Lung_Cancer | MAX | Basic domains | H2171 | GSM1008617 | 38287_sort_peaks.narrowPeak.bed.bed | Cistrome |
| Lung_Cancer | MYC | Basic domains | H2171 | GSM1008620 | 38288_sort_peaks.narrowPeak.bed.bed | Cistrome |
| Lung_Cancer | JUN | Basic domains | HCC827 | GSM2516998 | 83583_sort_peaks.narrowPeak.bed.bed | Cistrome |
| Lung_Cancer | JUN | Basic domains | HCC827 | GSM2517000 | 83923_sort_peaks.narrowPeak.bed.bed | Cistrome |
| Lung_Cancer | ASCL1 | Basic domains | NCI-H128 | GSM1499401 | 49724_sort_peaks.narrowPeak.bed.bed | Cistrome |
| Lung_Cancer | ASCL1 | Basic domains | NCI-H1755 | GSM1499402 | 49725_sort_peaks.narrowPeak.bed.bed | Cistrome |
| Lung_Cancer | ASCL1 | Basic domains | NCI-H2107 | GSM1499403 | 49726_sort_peaks.narrowPeak.bed.bed | Cistrome |
| Lung_Cancer | ASCL1 | Basic domains | NCI-H2107 | GSM1499404 | 49727_sort_peaks.narrowPeak.bed.bed | Cistrome |
| Lung_Cancer | ASCL1 | Basic domains | NCI-H2107 | GSM1700638 | 70121_sort_peaks.narrowPeak.bed.bed | Cistrome |
| Lung_Cancer | MYC | Basic domains | A549 | GSM1003607 | 45560_sort_peaks.narrowPeak.bed.bed | ENCODE |
| Lung_Cancer | CEBPB | Basic domains | A549 | GSM935630 | 45559_sort_peaks.narrowPeak.bed.bed | ENCODE |
| Lung_Cancer | CREB1 | Basic domains | A549 | GSM1010719 | 37349_sort_peaks.narrowPeak.bed.bed | ENCODE |
| Lung_Cancer | CREB1 | Basic domains | A549 | GSM1010720 | 37305_sort_peaks.narrowPeak.bed.bed | ENCODE |
| Lung_Cancer | JUN | Basic domains | A549 | GSM1010723 | 37354_sort_peaks.narrowPeak.bed.bed | ENCODE |
| Lung_Cancer | CREB1 | Basic domains | A549 | GSM1010726 | 37334_sort_peaks.narrowPeak.bed.bed | ENCODE |
| Lung_Cancer | MAX | Basic domains | A549 | GSM1010728 | 46070_sort_peaks.narrowPeak.bed.bed | ENCODE |
| Lung_Cancer | TCF12 | Basic domains | A549 | GSM1010746 | 46066_sort_peaks.narrowPeak.bed.bed | ENCODE |
| Lung_Cancer | FOSL2 | Basic domains | A549 | GSM1010748 | 46072_sort_peaks.narrowPeak.bed.bed | ENCODE |
| Lung_Cancer | ATF3 | Basic domains | A549 | GSM1010789 | 46055_sort_peaks.narrowPeak.bed.bed | ENCODE |
| Lung_Cancer | USF1 | Basic domains | A549 | GSM1010828 | 46064_sort_peaks.narrowPeak.bed.bed | ENCODE |
| Lung_Cancer | CEBPB | Basic domains | A549 | GSM1010871 | 46074_sort_peaks.narrowPeak.bed.bed | ENCODE |
| Lung_Cancer | USF1 | Basic domains | A549 | GSM803429 | 46062_sort_peaks.narrowPeak.bed.bed | ENCODE |
| Lung_Cancer | USF1 | Basic domains | A549 | GSM803457 | 46078_sort_peaks.narrowPeak.bed.bed | ENCODE |
| Lung_Cancer | FOSL2 | Basic domains | A549 | ENCSR067XTW_1 | 62626_sort_peaks.narrowPeak.bed.bed | ENCODE |
| Lung_Cancer | FOSL2 | Basic domains | A549 | ENCSR067XTW_2 | 62627_sort_peaks.narrowPeak.bed.bed | ENCODE |
| Lung_Cancer | FOSL2 | Basic domains | A549 | ENCSR067XTW_3 | 62628_sort_peaks.narrowPeak.bed.bed | ENCODE |
| Lung_Cancer | FOSL2 | Basic domains | A549 | ENCSR103UVU_1 | 62717_sort_peaks.narrowPeak.bed.bed | ENCODE |
| Lung_Cancer | FOSL2 | Basic domains | A549 | ENCSR103UVU_2 | 62718_sort_peaks.narrowPeak.bed.bed | ENCODE |
| Lung_Cancer | FOSL2 | Basic domains | A549 | ENCSR111ZXV_1 | 62753_sort_peaks.narrowPeak.bed.bed | ENCODE |
| Lung_Cancer | FOSL2 | Basic domains | A549 | ENCSR111ZXV_2 | 62754_sort_peaks.narrowPeak.bed.bed | ENCODE |
| Lung_Cancer | FOSL2 | Basic domains | A549 | ENCSR111ZXV_3 | 62755_sort_peaks.narrowPeak.bed.bed | ENCODE |
| Lung_Cancer | CEBPB | Basic domains | A549 | ENCSR182OZC_1 | 62928_sort_peaks.narrowPeak.bed.bed | ENCODE |
| Lung_Cancer | CEBPB | Basic domains | A549 | ENCSR182OZC_2 | 62929_sort_peaks.narrowPeak.bed.bed | ENCODE |
| Lung_Cancer | CEBPB | Basic domains | A549 | ENCSR190VMK_1 | 62938_sort_peaks.narrowPeak.bed.bed | ENCODE |
| Lung_Cancer | CEBPB | Basic domains | A549 | ENCSR190VMK_2 | 62939_sort_peaks.narrowPeak.bed.bed | ENCODE |
| Lung_Cancer | CEBPB | Basic domains | A549 | ENCSR190VMK_3 | 62940_sort_peaks.narrowPeak.bed.bed | ENCODE |
| Lung_Cancer | CEBPB | Basic domains | A549 | ENCSR216GEB_1 | 63003_sort_peaks.narrowPeak.bed.bed | ENCODE |
| Lung_Cancer | CEBPB | Basic domains | A549 | ENCSR216GEB_2 | 63004_sort_peaks.narrowPeak.bed.bed | ENCODE |
| Lung_Cancer | FOSL2 | Basic domains | A549 | ENCSR242EWU_1 | 63079_sort_peaks.narrowPeak.bed.bed | ENCODE |
| Lung_Cancer | FOSL2 | Basic domains | A549 | ENCSR242EWU_2 | 63080_sort_peaks.narrowPeak.bed.bed | ENCODE |
| Lung_Cancer | FOSL2 | Basic domains | A549 | ENCSR242EWU_3 | 63081_sort_peaks.narrowPeak.bed.bed | ENCODE |
| Lung_Cancer | CEBPB | Basic domains | A549 | ENCSR269IUA_1 | 63152_sort_peaks.narrowPeak.bed.bed | ENCODE |
| Lung_Cancer | CEBPB | Basic domains | A549 | ENCSR269IUA_2 | 63153_sort_peaks.narrowPeak.bed.bed | ENCODE |
| Lung_Cancer | CEBPB | Basic domains | A549 | ENCSR269IUA_3 | 63154_sort_peaks.narrowPeak.bed.bed | ENCODE |
| Lung_Cancer | FOSL2 | Basic domains | A549 | ENCSR274WRI_1 | 63169_sort_peaks.narrowPeak.bed.bed | ENCODE |
| Lung_Cancer | FOSL2 | Basic domains | A549 | ENCSR274WRI_2 | 63170_sort_peaks.narrowPeak.bed.bed | ENCODE |
| Lung_Cancer | FOSL2 | Basic domains | A549 | ENCSR274WRI_3 | 63171_sort_peaks.narrowPeak.bed.bed | ENCODE |
| Lung_Cancer | CEBPB | Basic domains | A549 | ENCSR299ILN_1 | 63230_sort_peaks.narrowPeak.bed.bed | ENCODE |
| Lung_Cancer | CEBPB | Basic domains | A549 | ENCSR299ILN_2 | 63231_sort_peaks.narrowPeak.bed.bed | ENCODE |
| Lung_Cancer | CEBPB | Basic domains | A549 | ENCSR299ILN_3 | 63232_sort_peaks.narrowPeak.bed.bed | ENCODE |
| Lung_Cancer | FOSL2 | Basic domains | A549 | ENCSR330IJE_1 | 63298_sort_peaks.narrowPeak.bed.bed | ENCODE |
| Lung_Cancer | FOSL2 | Basic domains | A549 | ENCSR330IJE_2 | 63299_sort_peaks.narrowPeak.bed.bed | ENCODE |
| Lung_Cancer | FOSL2 | Basic domains | A549 | ENCSR330IJE_3 | 63300_sort_peaks.narrowPeak.bed.bed | ENCODE |
| Lung_Cancer | CEBPB | Basic domains | A549 | ENCSR375BUB_1 | 63414_sort_peaks.narrowPeak.bed.bed | ENCODE |
| Lung_Cancer | CEBPB | Basic domains | A549 | ENCSR375BUB_2 | 63415_sort_peaks.narrowPeak.bed.bed | ENCODE |
| Lung_Cancer | CEBPB | Basic domains | A549 | ENCSR447ZMS_1 | 63605_sort_peaks.narrowPeak.bed.bed | ENCODE |
| Lung_Cancer | CEBPB | Basic domains | A549 | ENCSR447ZMS_2 | 63606_sort_peaks.narrowPeak.bed.bed | ENCODE |
| Lung_Cancer | CEBPB | Basic domains | A549 | ENCSR447ZMS_3 | 63607_sort_peaks.narrowPeak.bed.bed | ENCODE |
| Lung_Cancer | CEBPB | Basic domains | A549 | ENCSR474DCX_1 | 63665_sort_peaks.narrowPeak.bed.bed | ENCODE |
| Lung_Cancer | CEBPB | Basic domains | A549 | ENCSR474DCX_2 | 63666_sort_peaks.narrowPeak.bed.bed | ENCODE |
| Lung_Cancer | CEBPB | Basic domains | A549 | ENCSR474DCX_3 | 63667_sort_peaks.narrowPeak.bed.bed | ENCODE |
| Lung_Cancer | FOSL2 | Basic domains | A549 | ENCSR535HFQ_1 | 63827_sort_peaks.narrowPeak.bed.bed | ENCODE |
| Lung_Cancer | FOSL2 | Basic domains | A549 | ENCSR535HFQ_2 | 63828_sort_peaks.narrowPeak.bed.bed | ENCODE |
| Lung_Cancer | FOSL2 | Basic domains | A549 | ENCSR535HFQ_3 | 63829_sort_peaks.narrowPeak.bed.bed | ENCODE |
| Lung_Cancer | FOSL2 | Basic domains | A549 | ENCSR563GCV_1 | 63917_sort_peaks.narrowPeak.bed.bed | ENCODE |
| Lung_Cancer | FOSL2 | Basic domains | A549 | ENCSR563GCV_2 | 63918_sort_peaks.narrowPeak.bed.bed | ENCODE |
| Lung_Cancer | FOSL2 | Basic domains | A549 | ENCSR563GCV_3 | 63919_sort_peaks.narrowPeak.bed.bed | ENCODE |
| Lung_Cancer | FOSL2 | Basic domains | A549 | ENCSR593DGU_1 | 63983_sort_peaks.narrowPeak.bed.bed | ENCODE |
| Lung_Cancer | FOSL2 | Basic domains | A549 | ENCSR593DGU_2 | 63984_sort_peaks.narrowPeak.bed.bed | ENCODE |
| Lung_Cancer | CEBPB | Basic domains | A549 | ENCSR606ZTC_1 | 64016_sort_peaks.narrowPeak.bed.bed | ENCODE |
| Lung_Cancer | CEBPB | Basic domains | A549 | ENCSR606ZTC_2 | 64017_sort_peaks.narrowPeak.bed.bed | ENCODE |
| Lung_Cancer | FOSL2 | Basic domains | A549 | ENCSR686HIU_1 | 64222_sort_peaks.narrowPeak.bed.bed | ENCODE |
| Lung_Cancer | FOSL2 | Basic domains | A549 | ENCSR686HIU_2 | 64223_sort_peaks.narrowPeak.bed.bed | ENCODE |
| Lung_Cancer | CEBPB | Basic domains | A549 | ENCSR701TCU_1 | 64280_sort_peaks.narrowPeak.bed.bed | ENCODE |
| Lung_Cancer | CEBPB | Basic domains | A549 | ENCSR701TCU_2 | 64281_sort_peaks.narrowPeak.bed.bed | ENCODE |
| Lung_Cancer | CEBPB | Basic domains | A549 | ENCSR752AQK_1 | 64398_sort_peaks.narrowPeak.bed.bed | ENCODE |
| Lung_Cancer | CEBPB | Basic domains | A549 | ENCSR752AQK_2 | 64399_sort_peaks.narrowPeak.bed.bed | ENCODE |
| Lung_Cancer | CEBPB | Basic domains | A549 | ENCSR752AQK_3 | 64400_sort_peaks.narrowPeak.bed.bed | ENCODE |
| Lung_Cancer | FOSL2 | Basic domains | A549 | ENCSR775UFR_1 | 64484_sort_peaks.narrowPeak.bed.bed | ENCODE |
| Lung_Cancer | FOSL2 | Basic domains | A549 | ENCSR775UFR_2 | 64485_sort_peaks.narrowPeak.bed.bed | ENCODE |
| Lung_Cancer | FOSL2 | Basic domains | A549 | ENCSR775UFR_3 | 64486_sort_peaks.narrowPeak.bed.bed | ENCODE |
| Lung_Cancer | CEBPB | Basic domains | A549 | ENCSR853DWE_1 | 64669_sort_peaks.narrowPeak.bed.bed | ENCODE |
| Lung_Cancer | CEBPB | Basic domains | A549 | ENCSR853DWE_2 | 64670_sort_peaks.narrowPeak.bed.bed | ENCODE |
| Lung_Cancer | CEBPB | Basic domains | A549 | ENCSR853DWE_3 | 64671_sort_peaks.narrowPeak.bed.bed | ENCODE |
| Lung_Cancer | FOSL2 | Basic domains | A549 | ENCSR900RUE_1 | 64788_sort_peaks.narrowPeak.bed.bed | ENCODE |
| Lung_Cancer | FOSL2 | Basic domains | A549 | ENCSR900RUE_2 | 64789_sort_peaks.narrowPeak.bed.bed | ENCODE |
| Lung_Cancer | FOSL2 | Basic domains | A549 | ENCSR900RUE_3 | 64790_sort_peaks.narrowPeak.bed.bed | ENCODE |
| Lung_Cancer | SMAD3 | beta-Hairpin exposed by an alpha/beta-scaffold | A549 | GSM1246721 | 47584_sort_peaks.narrowPeak.bed.bed | Cistrome |
| Lung_Cancer | SMAD3 | beta-Hairpin exposed by an alpha/beta-scaffold | NCI-H441 | GSM1246713 | 47576_sort_peaks.narrowPeak.bed.bed | Cistrome |
| Lung_Cancer | SMAD3 | beta-Hairpin exposed by an alpha/beta-scaffold | NCI-H441 | GSM1246714 | 47577_sort_peaks.narrowPeak.bed.bed | Cistrome |
| Lung_Cancer | SMAD4 | beta-Hairpin exposed by an alpha/beta-scaffold | NCI-H441 | GSM1246718 | 47581_sort_peaks.narrowPeak.bed.bed | Cistrome |
| Lung_Cancer | SMAD4 | beta-Hairpin exposed by an alpha/beta-scaffold | NCI-H441 | GSM1246719 | 47582_sort_peaks.narrowPeak.bed.bed | Cistrome |
| Lung_Cancer | POLR2A | ENCODE: TF | A549 | GSM874633 | 36345_sort_peaks.narrowPeak.bed.bed | Cistrome |
| Lung_Cancer | POLR2A | ENCODE: TF | A549 | GSM874634 | 36351_sort_peaks.narrowPeak.bed.bed | Cistrome |
| Lung_Cancer | CHD1 | ENCODE: TF | A549 | GSM874641 | 36348_sort_peaks.narrowPeak.bed.bed | Cistrome |
| Lung_Cancer | POLR2A | ENCODE: TF | A549 | GSM2113332 | 70567_sort_peaks.narrowPeak.bed.bed | Cistrome |
| Lung_Cancer | POLR2A | ENCODE: TF | A549 | GSM2113333 | 70566_sort_peaks.narrowPeak.bed.bed | Cistrome |
| Lung_Cancer | POLR2A | ENCODE: TF | A549 | GSM2113334 | 70565_sort_peaks.narrowPeak.bed.bed | Cistrome |
| Lung_Cancer | POLR2A | ENCODE: TF | A549 | GSM2113335 | 70564_sort_peaks.narrowPeak.bed.bed | Cistrome |
| Lung_Cancer | POLR2A | ENCODE: TF | H2171 | GSM894104 | 37165_sort_peaks.narrowPeak.bed.bed | Cistrome |
| Lung_Cancer | POLR2A | ENCODE: TF | H2171 | GSM1195568 | 40219_sort_peaks.narrowPeak.bed.bed | Cistrome |
| Lung_Cancer | POLR2A | ENCODE: TF | H2171 | GSM1195570 | 40231_sort_peaks.narrowPeak.bed.bed | Cistrome |
| Lung_Cancer | POLR2A | ENCODE: TF | NCI-H2087 | GSM2066437 | 72395_sort_peaks.narrowPeak.bed.bed | Cistrome |
| Lung_Cancer | POLR2A | ENCODE: TF | A549 | GSM935299 | 45555_sort_peaks.narrowPeak.bed.bed | ENCODE |
| Lung_Cancer | EP300 | ENCODE: TF | A549 | GSM1010827 | 46056_sort_peaks.narrowPeak.bed.bed | ENCODE |
| Lung_Cancer | POLR2A | ENCODE: TF | A549 | GSM803360 | 46083_sort_peaks.narrowPeak.bed.bed | ENCODE |
| Lung_Cancer | POLR2A | ENCODE: TF | A549 | GSM803361 | 46061_sort_peaks.narrowPeak.bed.bed | ENCODE |
| Lung_Cancer | POLR2A | ENCODE: TF | A549 | GSM822288 | 45552_sort_peaks.narrowPeak.bed.bed | ENCODE |
| Lung_Cancer | EP300 | ENCODE: TF | A549 | ENCSR047EVQ_1 | 62601_sort_peaks.narrowPeak.bed.bed | ENCODE |
| Lung_Cancer | EP300 | ENCODE: TF | A549 | ENCSR047EVQ_2 | 62602_sort_peaks.narrowPeak.bed.bed | ENCODE |
| Lung_Cancer | EP300 | ENCODE: TF | A549 | ENCSR124VXG_1 | 62786_sort_peaks.narrowPeak.bed.bed | ENCODE |
| Lung_Cancer | EP300 | ENCODE: TF | A549 | ENCSR124VXG_2 | 62787_sort_peaks.narrowPeak.bed.bed | ENCODE |
| Lung_Cancer | EP300 | ENCODE: TF | A549 | ENCSR124VXG_3 | 62788_sort_peaks.narrowPeak.bed.bed | ENCODE |
| Lung_Cancer | EP300 | ENCODE: TF | A549 | ENCSR145YCX_1 | 62850_sort_peaks.narrowPeak.bed.bed | ENCODE |
| Lung_Cancer | EP300 | ENCODE: TF | A549 | ENCSR145YCX_2 | 62851_sort_peaks.narrowPeak.bed.bed | ENCODE |
| Lung_Cancer | EP300 | ENCODE: TF | A549 | ENCSR260WCE_1 | 63124_sort_peaks.narrowPeak.bed.bed | ENCODE |
| Lung_Cancer | EP300 | ENCODE: TF | A549 | ENCSR260WCE_2 | 63125_sort_peaks.narrowPeak.bed.bed | ENCODE |
| Lung_Cancer | EP300 | ENCODE: TF | A549 | ENCSR260WCE_3 | 63126_sort_peaks.narrowPeak.bed.bed | ENCODE |
| Lung_Cancer | EP300 | ENCODE: TF | A549 | ENCSR358ELZ_1 | 63378_sort_peaks.narrowPeak.bed.bed | ENCODE |
| Lung_Cancer | EP300 | ENCODE: TF | A549 | ENCSR358ELZ_2 | 63379_sort_peaks.narrowPeak.bed.bed | ENCODE |
| Lung_Cancer | EP300 | ENCODE: TF | A549 | ENCSR467VXG_1 | 63649_sort_peaks.narrowPeak.bed.bed | ENCODE |
| Lung_Cancer | EP300 | ENCODE: TF | A549 | ENCSR467VXG_2 | 63650_sort_peaks.narrowPeak.bed.bed | ENCODE |
| Lung_Cancer | EP300 | ENCODE: TF | A549 | ENCSR467VXG_3 | 63651_sort_peaks.narrowPeak.bed.bed | ENCODE |
| Lung_Cancer | SMC3 | ENCODE: TF | A549 | ENCSR481YWD_1 | 63687_sort_peaks.narrowPeak.bed.bed | ENCODE |
| Lung_Cancer | SMC3 | ENCODE: TF | A549 | ENCSR481YWD_2 | 63688_sort_peaks.narrowPeak.bed.bed | ENCODE |
| Lung_Cancer | EP300 | ENCODE: TF | A549 | ENCSR561ZRE_1 | 63909_sort_peaks.narrowPeak.bed.bed | ENCODE |
| Lung_Cancer | EP300 | ENCODE: TF | A549 | ENCSR561ZRE_2 | 63910_sort_peaks.narrowPeak.bed.bed | ENCODE |
| Lung_Cancer | EP300 | ENCODE: TF | A549 | ENCSR561ZRE_3 | 63911_sort_peaks.narrowPeak.bed.bed | ENCODE |
| Lung_Cancer | EP300 | ENCODE: TF | A549 | ENCSR610RKF_1 | 64028_sort_peaks.narrowPeak.bed.bed | ENCODE |
| Lung_Cancer | EP300 | ENCODE: TF | A549 | ENCSR610RKF_2 | 64029_sort_peaks.narrowPeak.bed.bed | ENCODE |
| Lung_Cancer | EP300 | ENCODE: TF | A549 | ENCSR610RKF_3 | 64030_sort_peaks.narrowPeak.bed.bed | ENCODE |
| Lung_Cancer | EP300 | ENCODE: TF | A549 | ENCSR686BQM_1 | 64219_sort_peaks.narrowPeak.bed.bed | ENCODE |
| Lung_Cancer | EP300 | ENCODE: TF | A549 | ENCSR686BQM_2 | 64220_sort_peaks.narrowPeak.bed.bed | ENCODE |
| Lung_Cancer | EP300 | ENCODE: TF | A549 | ENCSR686BQM_3 | 64221_sort_peaks.narrowPeak.bed.bed | ENCODE |
| Lung_Cancer | EP300 | ENCODE: TF | A549 | ENCSR770OTI_1 | 64458_sort_peaks.narrowPeak.bed.bed | ENCODE |
| Lung_Cancer | EP300 | ENCODE: TF | A549 | ENCSR770OTI_2 | 64459_sort_peaks.narrowPeak.bed.bed | ENCODE |
| Lung_Cancer | EP300 | ENCODE: TF | A549 | ENCSR770OTI_3 | 64460_sort_peaks.narrowPeak.bed.bed | ENCODE |
| Lung_Cancer | EP300 | ENCODE: TF | A549 | ENCSR792VMN_1 | 64534_sort_peaks.narrowPeak.bed.bed | ENCODE |
| Lung_Cancer | EP300 | ENCODE: TF | A549 | ENCSR792VMN_2 | 64535_sort_peaks.narrowPeak.bed.bed | ENCODE |
| Lung_Cancer | EP300 | ENCODE: TF | A549 | ENCSR841ASB_1 | 64638_sort_peaks.narrowPeak.bed.bed | ENCODE |
| Lung_Cancer | EP300 | ENCODE: TF | A549 | ENCSR841ASB_2 | 64639_sort_peaks.narrowPeak.bed.bed | ENCODE |
| Lung_Cancer | EP300 | ENCODE: TF | A549 | ENCSR841ASB_3 | 64640_sort_peaks.narrowPeak.bed.bed | ENCODE |
| Lung_Cancer | NKX2-1 | Helix-turn-helix domains | A549 | GSM2310996 | SRX2164786.bed | ChIP-Atlas |
| Lung_Cancer | NKX2-1 | Helix-turn-helix domains | A549 | GSM2310998 | SRX2164788.bed | ChIP-Atlas |
| Lung_Cancer | SPDEF | Helix-turn-helix domains | A549 | GSM2311000 | SRX2164790.bed | ChIP-Atlas |
| Lung_Cancer | SPDEF | Helix-turn-helix domains | A549 | GSM2311002 | SRX2164792.bed | ChIP-Atlas |
| Lung_Cancer | FOXA2 | Helix-turn-helix domains | A549 | GSM2401464 | SRX2368896.bed | ChIP-Atlas |
| Lung_Cancer | FOXA2 | Helix-turn-helix domains | A549 | GSM2401465 | SRX2368897.bed | ChIP-Atlas |
| Lung_Cancer | FOXD3 | Helix-turn-helix domains | A549 | GSM2451236 | SRX2480568.bed | ChIP-Atlas |
| Lung_Cancer | POU5F1 | Helix-turn-helix domains | A549 | GSM1412053 | 51139_sort_peaks.narrowPeak.bed.bed | Cistrome |
| Lung_Cancer | POU5F1 | Helix-turn-helix domains | A549 | GSM1412055 | 51141_sort_peaks.narrowPeak.bed.bed | Cistrome |
| Lung_Cancer | POU5F1 | Helix-turn-helix domains | A549 | GSM1412056 | 51142_sort_peaks.narrowPeak.bed.bed | Cistrome |
| Lung_Cancer | EHF | Helix-turn-helix domains | Calu-3 | GSM1548071 | 50819_sort_peaks.narrowPeak.bed.bed | Cistrome |
| Lung_Cancer | EHF | Helix-turn-helix domains | Calu-3 | GSM1548072 | 50820_sort_peaks.narrowPeak.bed.bed | Cistrome |
| Lung_Cancer | POU5F1 | Helix-turn-helix domains | A549 | GSM1412054 | 51140_sort_peaks.narrowPeak.bed.bed | Cistrome |
| Lung_Cancer | NKX2-1 | Helix-turn-helix domains | A549 | GSM2310997 | 74051_sort_peaks.narrowPeak.bed.bed | Cistrome |
| Lung_Cancer | NKX2-1 | Helix-turn-helix domains | A549 | GSM2310999 | 74049_sort_peaks.narrowPeak.bed.bed | Cistrome |
| Lung_Cancer | ZEB1 | Helix-turn-helix domains | H1975 | GSM2857243 | 84925_sort_peaks.narrowPeak.bed.bed | Cistrome |
| Lung_Cancer | ZEB1 | Helix-turn-helix domains | H1975 | GSM2857242 | 84926_sort_peaks.narrowPeak.bed.bed | Cistrome |
| Lung_Cancer | NKX2-1 | Helix-turn-helix domains | NCI-H1819 | GSM983100 | 35471_sort_peaks.narrowPeak.bed.bed | Cistrome |
| Lung_Cancer | NKX2-1 | Helix-turn-helix domains | NCI-H2087 | GSM983102 | 35475_sort_peaks.narrowPeak.bed.bed | Cistrome |
| Lung_Cancer | FOXA1 | Helix-turn-helix domains | NCI-H3122 | GSM983105 | 35473_sort_peaks.narrowPeak.bed.bed | Cistrome |
| Lung_Cancer | NKX2-1 | Helix-turn-helix domains | NCI-H3122 | GSM983104 | 35474_sort_peaks.narrowPeak.bed.bed | Cistrome |
| Lung_Cancer | NKX2-1 | Helix-turn-helix domains | NCI-H3122 | GSM983098 | 36411_sort_peaks.narrowPeak.bed.bed | Cistrome |
| Lung_Cancer | TTF1 | Helix-turn-helix domains | NCI-H441 | GSM1246715 | 47578_sort_peaks.narrowPeak.bed.bed | Cistrome |
| Lung_Cancer | TTF1 | Helix-turn-helix domains | NCI-H441 | GSM1246716 | 47579_sort_peaks.narrowPeak.bed.bed | Cistrome |
| Lung_Cancer | FOXA2 | Helix-turn-helix domains | A549 | GSM1010724 | 37366_sort_peaks.narrowPeak.bed.bed | ENCODE |
| Lung_Cancer | PBX3 | Helix-turn-helix domains | A549 | GSM1010755 | 46082_sort_peaks.narrowPeak.bed.bed | ENCODE |
| Lung_Cancer | E2F6 | Helix-turn-helix domains | A549 | GSM1010766 | 46071_sort_peaks.narrowPeak.bed.bed | ENCODE |
| Lung_Cancer | ELF1 | Helix-turn-helix domains | A549 | GSM1010790 | 46059_sort_peaks.narrowPeak.bed.bed | ENCODE |
| Lung_Cancer | FOXA1 | Helix-turn-helix domains | A549 | GSM1010826 | 46067_sort_peaks.narrowPeak.bed.bed | ENCODE |
| Lung_Cancer | ETS1 | Helix-turn-helix domains | A549 | GSM1010829 | 46068_sort_peaks.narrowPeak.bed.bed | ENCODE |
| Lung_Cancer | GABPA | Helix-turn-helix domains | A549 | GSM1010833 | 46080_sort_peaks.narrowPeak.bed.bed | ENCODE |
| Lung_Cancer | TEAD4 | Helix-turn-helix domains | A549 | GSM1010868 | 46075_sort_peaks.narrowPeak.bed.bed | ENCODE |
| Lung_Cancer | SIX5 | Helix-turn-helix domains | A549 | GSM1010879 | 46054_sort_peaks.narrowPeak.bed.bed | ENCODE |
| Lung_Cancer | RELA | Immunoglobulin fold | A549 | GSM847876 | 5388_sort_peaks.narrowPeak.bed.bed | Cistrome |
| Lung_Cancer | RELA | Immunoglobulin fold | A549 | GSM847877 | 5389_sort_peaks.narrowPeak.bed.bed | Cistrome |
| Lung_Cancer | STAT3 | Immunoglobulin fold | H358 | GSM2752894 | 87919_sort_peaks.narrowPeak.bed.bed | Cistrome |
| Lung_Cancer | STAT1 | Immunoglobulin fold | H358 | GSM2752893 | 87922_sort_peaks.narrowPeak.bed.bed | Cistrome |
| Lung_Cancer | BCL3 | Immunoglobulin fold | A549 | GSM1010775 | 46051_sort_peaks.narrowPeak.bed.bed | ENCODE |
| Lung_Cancer | BCL3 | Immunoglobulin fold | A549 | ENCSR013KHB_2 | 62534_sort_peaks.narrowPeak.bed.bed | ENCODE |
| Lung_Cancer | BCL3 | Immunoglobulin fold | A549 | ENCSR013KHB_3 | 62535_sort_peaks.narrowPeak.bed.bed | ENCODE |
| Lung_Cancer | BCL3 | Immunoglobulin fold | A549 | ENCSR186IQL_1 | 62932_sort_peaks.narrowPeak.bed.bed | ENCODE |
| Lung_Cancer | BCL3 | Immunoglobulin fold | A549 | ENCSR186IQL_2 | 62933_sort_peaks.narrowPeak.bed.bed | ENCODE |
| Lung_Cancer | BCL3 | Immunoglobulin fold | A549 | ENCSR186IQL_3 | 62934_sort_peaks.narrowPeak.bed.bed | ENCODE |
| Lung_Cancer | BCL3 | Immunoglobulin fold | A549 | ENCSR215UXN_1 | 63000_sort_peaks.narrowPeak.bed.bed | ENCODE |
| Lung_Cancer | BCL3 | Immunoglobulin fold | A549 | ENCSR215UXN_2 | 63001_sort_peaks.narrowPeak.bed.bed | ENCODE |
| Lung_Cancer | BCL3 | Immunoglobulin fold | A549 | ENCSR215UXN_3 | 63002_sort_peaks.narrowPeak.bed.bed | ENCODE |
| Lung_Cancer | BCL3 | Immunoglobulin fold | A549 | ENCSR288EPV_1 | 63199_sort_peaks.narrowPeak.bed.bed | ENCODE |
| Lung_Cancer | BCL3 | Immunoglobulin fold | A549 | ENCSR288EPV_2 | 63200_sort_peaks.narrowPeak.bed.bed | ENCODE |
| Lung_Cancer | BCL3 | Immunoglobulin fold | A549 | ENCSR446SPT_1 | 63601_sort_peaks.narrowPeak.bed.bed | ENCODE |
| Lung_Cancer | BCL3 | Immunoglobulin fold | A549 | ENCSR446SPT_2 | 63602_sort_peaks.narrowPeak.bed.bed | ENCODE |
| Lung_Cancer | BCL3 | Immunoglobulin fold | A549 | ENCSR473CRE_1 | 63663_sort_peaks.narrowPeak.bed.bed | ENCODE |
| Lung_Cancer | BCL3 | Immunoglobulin fold | A549 | ENCSR473CRE_2 | 63664_sort_peaks.narrowPeak.bed.bed | ENCODE |
| Lung_Cancer | BCL3 | Immunoglobulin fold | A549 | ENCSR544GUO_1 | 63853_sort_peaks.narrowPeak.bed.bed | ENCODE |
| Lung_Cancer | BCL3 | Immunoglobulin fold | A549 | ENCSR544GUO_2 | 63854_sort_peaks.narrowPeak.bed.bed | ENCODE |
| Lung_Cancer | BCL3 | Immunoglobulin fold | A549 | ENCSR544GUO_3 | 63855_sort_peaks.narrowPeak.bed.bed | ENCODE |
| Lung_Cancer | BCL3 | Immunoglobulin fold | A549 | ENCSR555AYD_1 | 63883_sort_peaks.narrowPeak.bed.bed | ENCODE |
| Lung_Cancer | BCL3 | Immunoglobulin fold | A549 | ENCSR555AYD_2 | 63884_sort_peaks.narrowPeak.bed.bed | ENCODE |
| Lung_Cancer | BCL3 | Immunoglobulin fold | A549 | ENCSR555AYD_3 | 63885_sort_peaks.narrowPeak.bed.bed | ENCODE |
| Lung_Cancer | BCL3 | Immunoglobulin fold | A549 | ENCSR639KFO_1 | 64101_sort_peaks.narrowPeak.bed.bed | ENCODE |
| Lung_Cancer | BCL3 | Immunoglobulin fold | A549 | ENCSR639KFO_2 | 64102_sort_peaks.narrowPeak.bed.bed | ENCODE |
| Lung_Cancer | BCL3 | Immunoglobulin fold | A549 | ENCSR639KFO_3 | 64103_sort_peaks.narrowPeak.bed.bed | ENCODE |
| Lung_Cancer | BCL3 | Immunoglobulin fold | A549 | ENCSR673OGC_1 | 64183_sort_peaks.narrowPeak.bed.bed | ENCODE |
| Lung_Cancer | BCL3 | Immunoglobulin fold | A549 | ENCSR673OGC_2 | 64184_sort_peaks.narrowPeak.bed.bed | ENCODE |
| Lung_Cancer | BCL3 | Immunoglobulin fold | A549 | ENCSR904OTM_1 | 64800_sort_peaks.narrowPeak.bed.bed | ENCODE |
| Lung_Cancer | BCL3 | Immunoglobulin fold | A549 | ENCSR904OTM_2 | 64801_sort_peaks.narrowPeak.bed.bed | ENCODE |
| Lung_Cancer | BCL3 | Immunoglobulin fold | A549 | ENCSR904OTM_3 | 64802_sort_peaks.narrowPeak.bed.bed | ENCODE |
| Lung_Cancer | BCL3 | Immunoglobulin fold | A549 | ENCSR948JXU_1 | 64912_sort_peaks.narrowPeak.bed.bed | ENCODE |
| Lung_Cancer | BCL3 | Immunoglobulin fold | A549 | ENCSR948JXU_2 | 64913_sort_peaks.narrowPeak.bed.bed | ENCODE |
| Lung_Cancer | BCL3 | Immunoglobulin fold | A549 | ENCSR948JXU_3 | 64914_sort_peaks.narrowPeak.bed.bed | ENCODE |
| Lung_Cancer | BCL3 | Immunoglobulin fold | A549 | ENCSR013KHB_1 | 62533_sort_peaks.narrowPeak.bed.bed | ENCODE |
| Lung_Cancer | TAF1 | Other all-alpha-helical DNA-binding domains | A549 | GSM1010812 | 46077_sort_peaks.narrowPeak.bed.bed | ENCODE |
| Lung_Cancer | BHLHE40 | TcoF: TF | A549 | GSM935440 | 45557_sort_peaks.narrowPeak.bed.bed | ENCODE |
| Lung_Cancer | NR3C1 | Zinc-coordinating DNA-binding domains | A549 | GSM2095208 | SRX1650254.bed | ChIP-Atlas |
| Lung_Cancer | NR3C1 | Zinc-coordinating DNA-binding domains | A549 | GSM2095209 | SRX1650255.bed | ChIP-Atlas |
| Lung_Cancer | NR3C1 | Zinc-coordinating DNA-binding domains | A549 | GSM2095210 | SRX1650256.bed | ChIP-Atlas |
| Lung_Cancer | NR3C1 | Zinc-coordinating DNA-binding domains | A549 | GSM2095218 | SRX1650398.bed | ChIP-Atlas |
| Lung_Cancer | NR3C1 | Zinc-coordinating DNA-binding domains | A549 | GSM2095219 | SRX1650399.bed | ChIP-Atlas |
| Lung_Cancer | NR3C1 | Zinc-coordinating DNA-binding domains | A549 | GSM2095220 | SRX1650400.bed | ChIP-Atlas |
| Lung_Cancer | NR3C1 | Zinc-coordinating DNA-binding domains | A549 | GSM2095221 | SRX1650401.bed | ChIP-Atlas |
| Lung_Cancer | GATA4 | Zinc-coordinating DNA-binding domains | A549 | GSM2255735 | SRX1989717.bed | ChIP-Atlas |
| Lung_Cancer | KLF10 | Zinc-coordinating DNA-binding domains | A549 | GSM2406901 | 75165_sort_peaks.narrowPeak.bed.bed | Cistrome |
| Lung_Cancer | KLF10 | Zinc-coordinating DNA-binding domains | A549 | GSM2406902 | 75164_sort_peaks.narrowPeak.bed.bed | Cistrome |
| Lung_Cancer | KLF10 | Zinc-coordinating DNA-binding domains | A549 | GSM2406903 | 75163_sort_peaks.narrowPeak.bed.bed | Cistrome |
| Lung_Cancer | PGR | Zinc-coordinating DNA-binding domains | AB32 | GSM770759 | 33569_sort_peaks.narrowPeak.bed.bed | Cistrome |
| Lung_Cancer | PGR | Zinc-coordinating DNA-binding domains | AB32 | GSM770758 | 33571_sort_peaks.narrowPeak.bed.bed | Cistrome |
| Lung_Cancer | SF1 | Zinc-coordinating DNA-binding domains | H295R | GSM1080949 | 33850_sort_peaks.narrowPeak.bed.bed | Cistrome |
| Lung_Cancer | SF1 | Zinc-coordinating DNA-binding domains | H295R | GSM1080947 | 33851_sort_peaks.narrowPeak.bed.bed | Cistrome |
| Lung_Cancer | SF1 | Zinc-coordinating DNA-binding domains | H295R | GSM1080948 | 33852_sort_peaks.narrowPeak.bed.bed | Cistrome |
| Lung_Cancer | SF1 | Zinc-coordinating DNA-binding domains | H295R | GSM1080950 | 33853_sort_peaks.narrowPeak.bed.bed | Cistrome |
| Lung_Cancer | REST | Zinc-coordinating DNA-binding domains | H295R | GSM1192175 | 35605_sort_peaks.narrowPeak.bed.bed | Cistrome |
| Lung_Cancer | REST | Zinc-coordinating DNA-binding domains | H295R | GSM1192174 | 35607_sort_peaks.narrowPeak.bed.bed | Cistrome |
| Lung_Cancer | NR0B1 | Zinc-coordinating DNA-binding domains | H460 | GSM2383939 | 82635_sort_peaks.narrowPeak.bed.bed | Cistrome |
| Lung_Cancer | CTCF | Zinc-coordinating DNA-binding domains | HRE | GSM749737 | 45844_sort_peaks.narrowPeak.bed.bed | Cistrome |
| Lung_Cancer | CTCF | Zinc-coordinating DNA-binding domains | HRE | GSM749727 | 45845_sort_peaks.narrowPeak.bed.bed | Cistrome |
| Lung_Cancer | PPARG | Zinc-coordinating DNA-binding domains | NCI-H1993 | GSM1409657 | 49099_sort_peaks.narrowPeak.bed.bed | Cistrome |
| Lung_Cancer | PPARG | Zinc-coordinating DNA-binding domains | NCI-H2347 | GSM1409659 | 49101_sort_peaks.narrowPeak.bed.bed | Cistrome |
| Lung_Cancer | CTCF | Zinc-coordinating DNA-binding domains | A549 | GSM1003581 | 45146_sort_peaks.narrowPeak.bed.bed | ENCODE |
| Lung_Cancer | CTCF | Zinc-coordinating DNA-binding domains | A549 | GSM1003582 | 45144_sort_peaks.narrowPeak.bed.bed | ENCODE |
| Lung_Cancer | CTCF | Zinc-coordinating DNA-binding domains | A549 | GSM1003606 | 45556_sort_peaks.narrowPeak.bed.bed | ENCODE |
| Lung_Cancer | CTCF | Zinc-coordinating DNA-binding domains | A549 | GSM1022639 | 45551_sort_peaks.narrowPeak.bed.bed | ENCODE |
| Lung_Cancer | CTCF | Zinc-coordinating DNA-binding domains | A549 | GSM1022640 | 45550_sort_peaks.narrowPeak.bed.bed | ENCODE |
| Lung_Cancer | GATA3 | Zinc-coordinating DNA-binding domains | A549 | GSM1010727 | 46058_sort_peaks.narrowPeak.bed.bed | ENCODE |
| Lung_Cancer | REST | Zinc-coordinating DNA-binding domains | A549 | GSM1010749 | 46069_sort_peaks.narrowPeak.bed.bed | ENCODE |
| Lung_Cancer | YY1 | Zinc-coordinating DNA-binding domains | A549 | GSM1010794 | 46065_sort_peaks.narrowPeak.bed.bed | ENCODE |
| Lung_Cancer | SP1 | Zinc-coordinating DNA-binding domains | A549 | GSM1010813 | 46060_sort_peaks.narrowPeak.bed.bed | ENCODE |
| Lung_Cancer | ZBTB33 | Zinc-coordinating DNA-binding domains | A549 | GSM1010832 | 46073_sort_peaks.narrowPeak.bed.bed | ENCODE |
| Lung_Cancer | NR3C1 | Zinc-coordinating DNA-binding domains | A549 | GSM803357 | 46076_sort_peaks.narrowPeak.bed.bed | ENCODE |
| Lung_Cancer | NR3C1 | Zinc-coordinating DNA-binding domains | A549 | GSM803358 | 46079_sort_peaks.narrowPeak.bed.bed | ENCODE |
| Lung_Cancer | NR3C1 | Zinc-coordinating DNA-binding domains | A549 | GSM803359 | 46081_sort_peaks.narrowPeak.bed.bed | ENCODE |
| Lung_Cancer | NR3C1 | Zinc-coordinating DNA-binding domains | A549 | GSM803371 | 46052_sort_peaks.narrowPeak.bed.bed | ENCODE |
| Lung_Cancer | CTCF | Zinc-coordinating DNA-binding domains | A549 | GSM822289 | 45558_sort_peaks.narrowPeak.bed.bed | ENCODE |
| Lung_Cancer | CTCF | Zinc-coordinating DNA-binding domains | A549 | GSM803453 | 46053_sort_peaks.narrowPeak.bed.bed | ENCODE |
| Lung_Cancer | CTCF | Zinc-coordinating DNA-binding domains | A549 | GSM803456 | 46063_sort_peaks.narrowPeak.bed.bed | ENCODE |
| Lung_Cancer | NR3C1 | Zinc-coordinating DNA-binding domains | A549 | ENCSR255CTA_1 | 63108_sort_peaks.narrowPeak.bed.bed | ENCODE |
| Lung_Cancer | NR3C1 | Zinc-coordinating DNA-binding domains | A549 | ENCSR255CTA_2 | 63109_sort_peaks.narrowPeak.bed.bed | ENCODE |
| Lung_Cancer | NR3C1 | Zinc-coordinating DNA-binding domains | A549 | ENCSR255CTA_3 | 63110_sort_peaks.narrowPeak.bed.bed | ENCODE |
| Lung_Cancer | NR3C1 | Zinc-coordinating DNA-binding domains | A549 | ENCSR287FWE_1 | 63195_sort_peaks.narrowPeak.bed.bed | ENCODE |
| Lung_Cancer | NR3C1 | Zinc-coordinating DNA-binding domains | A549 | ENCSR287FWE_2 | 63196_sort_peaks.narrowPeak.bed.bed | ENCODE |
| Lung_Cancer | NR3C1 | Zinc-coordinating DNA-binding domains | A549 | ENCSR287FWE_3 | 63197_sort_peaks.narrowPeak.bed.bed | ENCODE |
| Lung_Cancer | NR3C1 | Zinc-coordinating DNA-binding domains | A549 | ENCSR385RUW_1 | 63442_sort_peaks.narrowPeak.bed.bed | ENCODE |
| Lung_Cancer | NR3C1 | Zinc-coordinating DNA-binding domains | A549 | ENCSR385RUW_2 | 63443_sort_peaks.narrowPeak.bed.bed | ENCODE |
| Lung_Cancer | NR3C1 | Zinc-coordinating DNA-binding domains | A549 | ENCSR385RUW_3 | 63444_sort_peaks.narrowPeak.bed.bed | ENCODE |
| Lung_Cancer | NR3C1 | Zinc-coordinating DNA-binding domains | A549 | ENCSR424JOE_1 | 63548_sort_peaks.narrowPeak.bed.bed | ENCODE |
| Lung_Cancer | NR3C1 | Zinc-coordinating DNA-binding domains | A549 | ENCSR424JOE_2 | 63549_sort_peaks.narrowPeak.bed.bed | ENCODE |
| Lung_Cancer | NR3C1 | Zinc-coordinating DNA-binding domains | A549 | ENCSR424JOE_3 | 63550_sort_peaks.narrowPeak.bed.bed | ENCODE |
| Lung_Cancer | NR3C1 | Zinc-coordinating DNA-binding domains | A549 | ENCSR516IVY_1 | 63758_sort_peaks.narrowPeak.bed.bed | ENCODE |
| Lung_Cancer | NR3C1 | Zinc-coordinating DNA-binding domains | A549 | ENCSR516IVY_2 | 63759_sort_peaks.narrowPeak.bed.bed | ENCODE |
| Lung_Cancer | NR3C1 | Zinc-coordinating DNA-binding domains | A549 | ENCSR660RYY_1 | 64154_sort_peaks.narrowPeak.bed.bed | ENCODE |
| Lung_Cancer | NR3C1 | Zinc-coordinating DNA-binding domains | A549 | ENCSR660RYY_2 | 64155_sort_peaks.narrowPeak.bed.bed | ENCODE |
| Lung_Cancer | NR3C1 | Zinc-coordinating DNA-binding domains | A549 | ENCSR660RYY_3 | 64156_sort_peaks.narrowPeak.bed.bed | ENCODE |
| Lung_Cancer | NR3C1 | Zinc-coordinating DNA-binding domains | A549 | ENCSR686PFM_1 | 64224_sort_peaks.narrowPeak.bed.bed | ENCODE |
| Lung_Cancer | NR3C1 | Zinc-coordinating DNA-binding domains | A549 | ENCSR686PFM_2 | 64225_sort_peaks.narrowPeak.bed.bed | ENCODE |
| Lung_Cancer | NR3C1 | Zinc-coordinating DNA-binding domains | A549 | ENCSR686PFM_3 | 64226_sort_peaks.narrowPeak.bed.bed | ENCODE |
| Lung_Cancer | NR3C1 | Zinc-coordinating DNA-binding domains | A549 | ENCSR691GRA_1 | 64249_sort_peaks.narrowPeak.bed.bed | ENCODE |
| Lung_Cancer | NR3C1 | Zinc-coordinating DNA-binding domains | A549 | ENCSR691GRA_2 | 64250_sort_peaks.narrowPeak.bed.bed | ENCODE |
| Lung_Cancer | NR3C1 | Zinc-coordinating DNA-binding domains | A549 | ENCSR691GRA_3 | 64251_sort_peaks.narrowPeak.bed.bed | ENCODE |
| Lung_Cancer | NR3C1 | Zinc-coordinating DNA-binding domains | A549 | ENCSR720DXT_1 | 64331_sort_peaks.narrowPeak.bed.bed | ENCODE |
| Lung_Cancer | NR3C1 | Zinc-coordinating DNA-binding domains | A549 | ENCSR720DXT_2 | 64332_sort_peaks.narrowPeak.bed.bed | ENCODE |
| Lung_Cancer | NR3C1 | Zinc-coordinating DNA-binding domains | A549 | ENCSR720DXT_3 | 64333_sort_peaks.narrowPeak.bed.bed | ENCODE |
| Lung_Cancer | NR3C1 | Zinc-coordinating DNA-binding domains | A549 | ENCSR773NQB_1 | 64471_sort_peaks.narrowPeak.bed.bed | ENCODE |
| Lung_Cancer | NR3C1 | Zinc-coordinating DNA-binding domains | A549 | ENCSR773NQB_2 | 64472_sort_peaks.narrowPeak.bed.bed | ENCODE |
| Lung_Cancer | NR3C1 | Zinc-coordinating DNA-binding domains | A549 | ENCSR773NQB_3 | 64473_sort_peaks.narrowPeak.bed.bed | ENCODE |
| Lung_Cancer | NR3C1 | Zinc-coordinating DNA-binding domains | A549 | ENCSR919OXR_1 | 64862_sort_peaks.narrowPeak.bed.bed | ENCODE |
| Lung_Cancer | NR3C1 | Zinc-coordinating DNA-binding domains | A549 | ENCSR919OXR_2 | 64863_sort_peaks.narrowPeak.bed.bed | ENCODE |
| Lung_Cancer | NR3C1 | Zinc-coordinating DNA-binding domains | A549 | ENCSR919OXR_3 | 64864_sort_peaks.narrowPeak.bed.bed | ENCODE |
| Lung_Cancer | NR3C1 | Zinc-coordinating DNA-binding domains | A549 | ENCSR952GOL_1 | 64930_sort_peaks.narrowPeak.bed.bed | ENCODE |
| Lung_Cancer | NR3C1 | Zinc-coordinating DNA-binding domains | A549 | ENCSR952GOL_2 | 64931_sort_peaks.narrowPeak.bed.bed | ENCODE |
| Lung_Cancer | NR3C1 | Zinc-coordinating DNA-binding domains | A549 | ENCSR952GOL_3 | 64932_sort_peaks.narrowPeak.bed.bed | ENCODE |
| Colon_Cancer | SRF | alpha-Helices exposed by beta-structures | HCT116 | GSM1010851 | 46211_sort_peaks.narrowPeak.bed.bed | ENCODE |
| Colon_Cancer | TCF4 | Basic domains | HCT116 | GSM1535697 | 54661_sort_peaks.narrowPeak.bed.bed | Cistrome |
| Colon_Cancer | TCF4 | Basic domains | HCT116 | GSM1535698 | 54662_sort_peaks.narrowPeak.bed.bed | Cistrome |
| Colon_Cancer | TCF4 | Basic domains | HCT116 | GSM1535699 | 54663_sort_peaks.narrowPeak.bed.bed | Cistrome |
| Colon_Cancer | TCF4 | Basic domains | HCT116 | GSM1535700 | 54664_sort_peaks.narrowPeak.bed.bed | Cistrome |
| Colon_Cancer | TCF4 | Basic domains | HCT116 | GSM1535703 | 54667_sort_peaks.narrowPeak.bed.bed | Cistrome |
| Colon_Cancer | TCF4 | Basic domains | HCT116 | GSM1535704 | 54668_sort_peaks.narrowPeak.bed.bed | Cistrome |
| Colon_Cancer | TCF4 | Basic domains | HCT116 | GSM1535705 | 54669_sort_peaks.narrowPeak.bed.bed | Cistrome |
| Colon_Cancer | TCF4 | Basic domains | HCT116 | GSM1535706 | 54670_sort_peaks.narrowPeak.bed.bed | Cistrome |
| Colon_Cancer | TCF4 | Basic domains | LS174T | GSM460122 | 11_sort_peaks.narrowPeak.bed.bed | Cistrome |
| Colon_Cancer | TCF4 | Basic domains | LS174T | GSM460124 | 9_sort_peaks.narrowPeak.bed.bed | Cistrome |
| Colon_Cancer | TCF4 | Basic domains | LS174T | GSM460125 | 8_sort_peaks.narrowPeak.bed.bed | Cistrome |
| Colon_Cancer | TCF4 | Basic domains | LS174T | GSM460127 | 6_sort_peaks.narrowPeak.bed.bed | Cistrome |
| Colon_Cancer | MYC | Basic domains | HCT116 | GSM2576763 | 76186_sort_peaks.narrowPeak.bed.bed | Cistrome |
| Colon_Cancer | MYC | Basic domains | HCT116 | GSM2065880 | 70810_sort_peaks.narrowPeak.bed.bed | Cistrome |
| Colon_Cancer | MYC | Basic domains | HCT116 | GSM2065881 | 70809_sort_peaks.narrowPeak.bed.bed | Cistrome |
| Colon_Cancer | MYC | Basic domains | HCT116 | GSM2065882 | 70808_sort_peaks.narrowPeak.bed.bed | Cistrome |
| Colon_Cancer | MYC | Basic domains | HCT116 | GSM2065883 | 70807_sort_peaks.narrowPeak.bed.bed | Cistrome |
| Colon_Cancer | MYC | Basic domains | HCT116 | GSM2065884 | 70806_sort_peaks.narrowPeak.bed.bed | Cistrome |
| Colon_Cancer | MYC | Basic domains | HCT116 | GSM2065885 | 70805_sort_peaks.narrowPeak.bed.bed | Cistrome |
| Colon_Cancer | JUND | Basic domains | GP5d | GSM1240819 | 42924_sort_peaks.narrowPeak.bed.bed | Cistrome |
| Colon_Cancer | MYC | Basic domains | GP5d | GSM1240821 | 42926_sort_peaks.narrowPeak.bed.bed | Cistrome |
| Colon_Cancer | JUND | Basic domains | GP5d | GSM1240833 | 42938_sort_peaks.narrowPeak.bed.bed | Cistrome |
| Colon_Cancer | MYC | Basic domains | GP5d | GSM1240835 | 42940_sort_peaks.narrowPeak.bed.bed | Cistrome |
| Colon_Cancer | FOSL1 | Basic domains | HCT116 | GSM1010756 | 46212_sort_peaks.narrowPeak.bed.bed | ENCODE |
| Colon_Cancer | ATF3 | Basic domains | HCT116 | GSM1010757 | 46217_sort_peaks.narrowPeak.bed.bed | ENCODE |
| Colon_Cancer | USF1 | Basic domains | HCT116 | GSM1010836 | 46205_sort_peaks.narrowPeak.bed.bed | ENCODE |
| Colon_Cancer | JUND | Basic domains | HCT116 | GSM1010847 | 46213_sort_peaks.narrowPeak.bed.bed | ENCODE |
| Colon_Cancer | CEBPB | Basic domains | HCT116 | GSM1010852 | 46206_sort_peaks.narrowPeak.bed.bed | ENCODE |
| Colon_Cancer | MAX | Basic domains | HCT116 | GSM1010904 | 46216_sort_peaks.narrowPeak.bed.bed | ENCODE |
| Colon_Cancer | TCF3 | Basic domains | HCT116 | GSM2065887 | EXP036152_liftoverhg19.bed | GTRD |
| Colon_Cancer | CEBPB | Basic domains | LS180 | GSE31939 | GSE31939.CEBPB.LS180_remap2018_hg19__v1_2.bed | Remap |
| Colon_Cancer | TCF4 | Basic domains | LS180 | GSE31939 | GSE31939.TCF4.LS180_remap2018_hg19__v1_2.bed | Remap |
| Colon_Cancer | NCOA1 | Basic domains | LS180 | GSE39277 | GSE39277.NCOA1.LS180_remap2018_hg19__v1_2.bed | Remap |
| Colon_Cancer | MYC | Basic domains | LS174T | GSE59223 | GSE59223.MYC.LS174T_BI8622_remap2018_hg19__v1_2.bed | Remap |
| Colon_Cancer | MYC | Basic domains | LS174T | GSE59223 | GSE59223.MYC.LS174T_DMSO_remap2018_hg19__v1_2.bed | Remap |
| Colon_Cancer | ATF3 | Basic domains | HCT116 | GSE74355 | GSE74355.ATF3.HCT116_CAMP_remap2018_hg19__v1_2.bed | Remap |
| Colon_Cancer | ATF3 | Basic domains | HCT116 | GSE74355 | GSE74355.ATF3.HCT116_DMSO_remap2018_hg19__v1_2.bed | Remap |
| Colon_Cancer | ATF3 | Basic domains | HCT116 | GSE74355 | GSE74355.ATF3.HCT116_DMSO_KOATF3_remap2018_hg19__v1_2.bed | Remap |
| Colon_Cancer | POLR2A | ENCODE: TF | HCT116 | GSM1065010 | 38576_sort_peaks.narrowPeak.bed.bed | Cistrome |
| Colon_Cancer | POLR2A | ENCODE: TF | HCT116 | GSM1154513 | 34398_sort_peaks.narrowPeak.bed.bed | Cistrome |
| Colon_Cancer | POLR2A | ENCODE: TF | HCT116 | GSM1162752 | 39917_sort_peaks.narrowPeak.bed.bed | Cistrome |
| Colon_Cancer | POLR2A | ENCODE: TF | HCT116 | GSM1162755 | 39921_sort_peaks.narrowPeak.bed.bed | Cistrome |
| Colon_Cancer | EP300 | ENCODE: TF | HCT116 | GSM1240110 | 42907_sort_peaks.narrowPeak.bed.bed | Cistrome |
| Colon_Cancer | POLR2A | ENCODE: TF | HCT116 | GSM1465032 | 49533_sort_peaks.narrowPeak.bed.bed | Cistrome |
| Colon_Cancer | POLR2A | ENCODE: TF | HCT116 | GSM1465033 | 49534_sort_peaks.narrowPeak.bed.bed | Cistrome |
| Colon_Cancer | POLR2A | ENCODE: TF | HCT116 | GSM1465034 | 49535_sort_peaks.narrowPeak.bed.bed | Cistrome |
| Colon_Cancer | POLR2A | ENCODE: TF | HCT116 | GSM1545657 | 51843_sort_peaks.narrowPeak.bed.bed | Cistrome |
| Colon_Cancer | POLR2A | ENCODE: TF | HCT116 | GSM1545658 | 51844_sort_peaks.narrowPeak.bed.bed | Cistrome |
| Colon_Cancer | POLR2A | ENCODE: TF | HCT116 | GSM1545659 | 51845_sort_peaks.narrowPeak.bed.bed | Cistrome |
| Colon_Cancer | POLR2A | ENCODE: TF | HCT116 | GSM1545660 | 51846_sort_peaks.narrowPeak.bed.bed | Cistrome |
| Colon_Cancer | POLR2A | ENCODE: TF | HCT116 | GSM1545661 | 51847_sort_peaks.narrowPeak.bed.bed | Cistrome |
| Colon_Cancer | POLR2A | ENCODE: TF | HCT116 | GSM1545662 | 51848_sort_peaks.narrowPeak.bed.bed | Cistrome |
| Colon_Cancer | POLR2A | ENCODE: TF | HCT116 | GSM1545663 | 51849_sort_peaks.narrowPeak.bed.bed | Cistrome |
| Colon_Cancer | POLR2A | ENCODE: TF | HCT116 | GSM1545664 | 51850_sort_peaks.narrowPeak.bed.bed | Cistrome |
| Colon_Cancer | POLR2A | ENCODE: TF | HCT116 | GSM1545668 | 51854_sort_peaks.narrowPeak.bed.bed | Cistrome |
| Colon_Cancer | POLR2A | ENCODE: TF | HCT116 | GSM1545669 | 51855_sort_peaks.narrowPeak.bed.bed | Cistrome |
| Colon_Cancer | POLR2A | ENCODE: TF | HCT116 | GSM1545670 | 51856_sort_peaks.narrowPeak.bed.bed | Cistrome |
| Colon_Cancer | POLR2A | ENCODE: TF | HCT116 | GSM1545671 | 51857_sort_peaks.narrowPeak.bed.bed | Cistrome |
| Colon_Cancer | POLR2A | ENCODE: TF | HCT116 | GSM1545672 | 51858_sort_peaks.narrowPeak.bed.bed | Cistrome |
| Colon_Cancer | POLR2A | ENCODE: TF | HCT116 | GSM1545673 | 51859_sort_peaks.narrowPeak.bed.bed | Cistrome |
| Colon_Cancer | POLR2A | ENCODE: TF | HCT116 | GSM1727092 | 55662_sort_peaks.narrowPeak.bed.bed | Cistrome |
| Colon_Cancer | POLR2A | ENCODE: TF | HCT116 | GSM1727093 | 55663_sort_peaks.narrowPeak.bed.bed | Cistrome |
| Colon_Cancer | POLR2A | ENCODE: TF | HCT116 | GSM1727100 | 55670_sort_peaks.narrowPeak.bed.bed | Cistrome |
| Colon_Cancer | POLR2A | ENCODE: TF | HCT116 | GSM1727101 | 55671_sort_peaks.narrowPeak.bed.bed | Cistrome |
| Colon_Cancer | POLR2A | ENCODE: TF | HCT116 | GSM1727102 | 55672_sort_peaks.narrowPeak.bed.bed | Cistrome |
| Colon_Cancer | POLR2A | ENCODE: TF | HCT116 | GSM1727103 | 55673_sort_peaks.narrowPeak.bed.bed | Cistrome |
| Colon_Cancer | POLR2A | ENCODE: TF | HCT116 | GSM1727104 | 55674_sort_peaks.narrowPeak.bed.bed | Cistrome |
| Colon_Cancer | POLR2A | ENCODE: TF | HCT116 | GSM1727105 | 55675_sort_peaks.narrowPeak.bed.bed | Cistrome |
| Colon_Cancer | POLR2A | ENCODE: TF | HCT116 | GSM1727107 | 55677_sort_peaks.narrowPeak.bed.bed | Cistrome |
| Colon_Cancer | POLR2A | ENCODE: TF | HCT116 | GSM1727108 | 55678_sort_peaks.narrowPeak.bed.bed | Cistrome |
| Colon_Cancer | POLR2A | ENCODE: TF | HCT116 | GSM1727109 | 55679_sort_peaks.narrowPeak.bed.bed | Cistrome |
| Colon_Cancer | POLR2A | ENCODE: TF | HCT116 | GSM1727110 | 55680_sort_peaks.narrowPeak.bed.bed | Cistrome |
| Colon_Cancer | POLR2A | ENCODE: TF | HCT116 | GSM1866693 | 57088_sort_peaks.narrowPeak.bed.bed | Cistrome |
| Colon_Cancer | POLR2A | ENCODE: TF | HCT116 | GSM1866694 | 57089_sort_peaks.narrowPeak.bed.bed | Cistrome |
| Colon_Cancer | TRIM28 | ENCODE: TF | HCT116 | GSM1866695 | 57090_sort_peaks.narrowPeak.bed.bed | Cistrome |
| Colon_Cancer | POLR2A | ENCODE: TF | HCT116 | GSM749817 | 1152_sort_peaks.narrowPeak.bed.bed | Cistrome |
| Colon_Cancer | POLR2A | ENCODE: TF | HCT116 | GSM749821 | 1151_sort_peaks.narrowPeak.bed.bed | Cistrome |
| Colon_Cancer | POLR2A | ENCODE: TF | HCT116 | GSM832460 | 5800_sort_peaks.narrowPeak.bed.bed | Cistrome |
| Colon_Cancer | POLR2A | ENCODE: TF | HCT116 | GSM832461 | 5801_sort_peaks.narrowPeak.bed.bed | Cistrome |
| Colon_Cancer | POLR2A | ENCODE: TF | HCT116 | GSM833286 | 5661_sort_peaks.narrowPeak.bed.bed | Cistrome |
| Colon_Cancer | POLR2A | ENCODE: TF | LS174T | GSM889410 | 8542_sort_peaks.narrowPeak.bed.bed | Cistrome |
| Colon_Cancer | POLR2A | ENCODE: TF | LS174T | GSM889411 | 8543_sort_peaks.narrowPeak.bed.bed | Cistrome |
| Colon_Cancer | POLR2A | ENCODE: TF | LS174T | GSM889412 | 8544_sort_peaks.narrowPeak.bed.bed | Cistrome |
| Colon_Cancer | POLR2A | ENCODE: TF | LS174T | GSM889413 | 8545_sort_peaks.narrowPeak.bed.bed | Cistrome |
| Colon_Cancer | POLR2A | ENCODE: TF | LS174T | GSM889414 | 8546_sort_peaks.narrowPeak.bed.bed | Cistrome |
| Colon_Cancer | POLR2A | ENCODE: TF | LS174T | GSM889415 | 8547_sort_peaks.narrowPeak.bed.bed | Cistrome |
| Colon_Cancer | POLR2A | ENCODE: TF | HCT116 | GSM935426 | 45715_sort_peaks.narrowPeak.bed.bed | Cistrome |
| Colon_Cancer | MCM2 | ENCODE: TF | HCT116 | GSM2474865 | 83886_sort_peaks.narrowPeak.bed.bed | Cistrome |
| Colon_Cancer | POLR2A | ENCODE: TF | HCT116 | GSM2058662 | 68785_sort_peaks.narrowPeak.bed.bed | Cistrome |
| Colon_Cancer | POLR2A | ENCODE: TF | HCT116 | GSM2058663 | 68784_sort_peaks.narrowPeak.bed.bed | Cistrome |
| Colon_Cancer | POLR2A | ENCODE: TF | HCT116 | GSM2058671 | 68779_sort_peaks.narrowPeak.bed.bed | Cistrome |
| Colon_Cancer | POLR2A | ENCODE: TF | HCT116 | GSM2058672 | 68778_sort_peaks.narrowPeak.bed.bed | Cistrome |
| Colon_Cancer | POLR2A | ENCODE: TF | HCT116 | GSM2305574 | 76192_sort_peaks.narrowPeak.bed.bed | Cistrome |
| Colon_Cancer | POLR2A | ENCODE: TF | HCT116 | GSM2305575 | 76191_sort_peaks.narrowPeak.bed.bed | Cistrome |
| Colon_Cancer | POLR2A | ENCODE: TF | HCT116 | GSM2305576 | 76190_sort_peaks.narrowPeak.bed.bed | Cistrome |
| Colon_Cancer | POLR2A | ENCODE: TF | HCT116 | GSM2305577 | 76189_sort_peaks.narrowPeak.bed.bed | Cistrome |
| Colon_Cancer | POLR2A | ENCODE: TF | HCT116 | GSM2305578 | 76188_sort_peaks.narrowPeak.bed.bed | Cistrome |
| Colon_Cancer | POLR2A | ENCODE: TF | HCT116 | GSM2305579 | 76187_sort_peaks.narrowPeak.bed.bed | Cistrome |
| Colon_Cancer | POLR2A | ENCODE: TF | HCT116 | GSM2571036 | 88903_sort_peaks.narrowPeak.bed.bed | Cistrome |
| Colon_Cancer | POLR2A | ENCODE: TF | HCT116 | GSM2571037 | 88902_sort_peaks.narrowPeak.bed.bed | Cistrome |
| Colon_Cancer | POLR2A | ENCODE: TF | HCT116 | GSM2571038 | 88911_sort_peaks.narrowPeak.bed.bed | Cistrome |
| Colon_Cancer | POLR2A | ENCODE: TF | HCT116 | GSM2571039 | 88910_sort_peaks.narrowPeak.bed.bed | Cistrome |
| Colon_Cancer | POLR2A | ENCODE: TF | HCT116 | GSM2571041 | 83953_sort_peaks.narrowPeak.bed.bed | Cistrome |
| Colon_Cancer | POLR2A | ENCODE: TF | HCT116 | GSM2571042 | 83950_sort_peaks.narrowPeak.bed.bed | Cistrome |
| Colon_Cancer | POLR2A | ENCODE: TF | HCT116 | GSM2571043 | 83951_sort_peaks.narrowPeak.bed.bed | Cistrome |
| Colon_Cancer | POLR2A | ENCODE: TF | HCT116 | GSM2571044 | 83956_sort_peaks.narrowPeak.bed.bed | Cistrome |
| Colon_Cancer | POLR2A | ENCODE: TF | HCT116 | GSM2571045 | 83957_sort_peaks.narrowPeak.bed.bed | Cistrome |
| Colon_Cancer | POLR2A | ENCODE: TF | HCT116 | GSM2571047 | 83955_sort_peaks.narrowPeak.bed.bed | Cistrome |
| Colon_Cancer | POLR2A | ENCODE: TF | HCT116 | GSM2571048 | 83958_sort_peaks.narrowPeak.bed.bed | Cistrome |
| Colon_Cancer | POLR2A | ENCODE: TF | HCT116 | GSM2571049 | 83959_sort_peaks.narrowPeak.bed.bed | Cistrome |
| Colon_Cancer | POLR2A | ENCODE: TF | HCT116 | GSM2571050 | 88092_sort_peaks.narrowPeak.bed.bed | Cistrome |
| Colon_Cancer | POLR2A | ENCODE: TF | HCT116 | GSM2571051 | 88091_sort_peaks.narrowPeak.bed.bed | Cistrome |
| Colon_Cancer | POLR2A | ENCODE: TF | HCT116 | GSM2571052 | 88094_sort_peaks.narrowPeak.bed.bed | Cistrome |
| Colon_Cancer | POLR2A | ENCODE: TF | HCT116 | GSM2571053 | 88093_sort_peaks.narrowPeak.bed.bed | Cistrome |
| Colon_Cancer | POLR2A | ENCODE: TF | HCT116 | GSM2571054 | 88096_sort_peaks.narrowPeak.bed.bed | Cistrome |
| Colon_Cancer | POLR2A | ENCODE: TF | HCT116 | GSM2571055 | 88095_sort_peaks.narrowPeak.bed.bed | Cistrome |
| Colon_Cancer | POLR2A | ENCODE: TF | HCT116 | GSM2571056 | 88098_sort_peaks.narrowPeak.bed.bed | Cistrome |
| Colon_Cancer | POLR2A | ENCODE: TF | HCT116 | GSM2571057 | 88097_sort_peaks.narrowPeak.bed.bed | Cistrome |
| Colon_Cancer | POLR2A | ENCODE: TF | HCT116 | GSM2571058 | 88100_sort_peaks.narrowPeak.bed.bed | Cistrome |
| Colon_Cancer | POLR2A | ENCODE: TF | HCT116 | GSM2571059 | 88099_sort_peaks.narrowPeak.bed.bed | Cistrome |
| Colon_Cancer | POLR2A | ENCODE: TF | HCT116 | GSM2571060 | 84236_sort_peaks.narrowPeak.bed.bed | Cistrome |
| Colon_Cancer | POLR2A | ENCODE: TF | HCT116 | GSM2571061 | 84237_sort_peaks.narrowPeak.bed.bed | Cistrome |
| Colon_Cancer | POLR2A | ENCODE: TF | HCT116 | GSM2571062 | 84238_sort_peaks.narrowPeak.bed.bed | Cistrome |
| Colon_Cancer | POLR2A | ENCODE: TF | HCT116 | GSM2571063 | 84239_sort_peaks.narrowPeak.bed.bed | Cistrome |
| Colon_Cancer | POLR2A | ENCODE: TF | HCT116 | GSM2667962 | 82333_sort_peaks.narrowPeak.bed.bed | Cistrome |
| Colon_Cancer | POLR2A | ENCODE: TF | HCT116 | GSM2667963 | 82332_sort_peaks.narrowPeak.bed.bed | Cistrome |
| Colon_Cancer | POLR2A | ENCODE: TF | HCT116 | GSM2667964 | 82337_sort_peaks.narrowPeak.bed.bed | Cistrome |
| Colon_Cancer | POLR2A | ENCODE: TF | HCT116 | GSM2667965 | 82336_sort_peaks.narrowPeak.bed.bed | Cistrome |
| Colon_Cancer | POLR2A | ENCODE: TF | HCT116 | GSM2670975 | 83342_sort_peaks.narrowPeak.bed.bed | Cistrome |
| Colon_Cancer | POLR2A | ENCODE: TF | HCT116 | GSM2670976 | 83343_sort_peaks.narrowPeak.bed.bed | Cistrome |
| Colon_Cancer | POLR2A | ENCODE: TF | HCT116 | GSM2670977 | 83344_sort_peaks.narrowPeak.bed.bed | Cistrome |
| Colon_Cancer | POLR2A | ENCODE: TF | HCT116 | GSM2670978 | 83349_sort_peaks.narrowPeak.bed.bed | Cistrome |
| Colon_Cancer | POLR2A | ENCODE: TF | HCT116 | GSM2670979 | 83350_sort_peaks.narrowPeak.bed.bed | Cistrome |
| Colon_Cancer | POLR2A | ENCODE: TF | HCT116 | GSM2670981 | 82395_sort_peaks.narrowPeak.bed.bed | Cistrome |
| Colon_Cancer | POLR2A | ENCODE: TF | HCT116 | GSM2670982 | 86354_sort_peaks.narrowPeak.bed.bed | Cistrome |
| Colon_Cancer | POLR2A | ENCODE: TF | HCT116 | GSM2670983 | 82396_sort_peaks.narrowPeak.bed.bed | Cistrome |
| Colon_Cancer | POLR2A | ENCODE: TF | HCT116 | GSM2670984 | 82394_sort_peaks.narrowPeak.bed.bed | Cistrome |
| Colon_Cancer | POLR2A | ENCODE: TF | HCT116 | GSM2670985 | 86619_sort_peaks.narrowPeak.bed.bed | Cistrome |
| Colon_Cancer | POLR2A | ENCODE: TF | HCT116 | GSM2670986 | 86622_sort_peaks.narrowPeak.bed.bed | Cistrome |
| Colon_Cancer | POLR2A | ENCODE: TF | HCT116 | GSM2670987 | 86621_sort_peaks.narrowPeak.bed.bed | Cistrome |
| Colon_Cancer | POLR2A | ENCODE: TF | HCT116 | GSM2670988 | 82412_sort_peaks.narrowPeak.bed.bed | Cistrome |
| Colon_Cancer | POLR2A | ENCODE: TF | HCT116 | GSM2712770 | 86928_sort_peaks.narrowPeak.bed.bed | Cistrome |
| Colon_Cancer | POLR2A | ENCODE: TF | HCT116 | GSM2712771 | 86927_sort_peaks.narrowPeak.bed.bed | Cistrome |
| Colon_Cancer | POLR2A | ENCODE: TF | HCT116 | GSM2712772 | 86929_sort_peaks.narrowPeak.bed.bed | Cistrome |
| Colon_Cancer | SMC3 | ENCODE: TF | GP5d | GSM1240826 | 42931_sort_peaks.narrowPeak.bed.bed | Cistrome |
| Colon_Cancer | SMC3 | ENCODE: TF | GP5d | GSM1240840 | 42945_sort_peaks.narrowPeak.bed.bed | Cistrome |
| Colon_Cancer | POLR2A | ENCODE: TF | HCT116 | GSM970210 | 33548_sort_peaks.narrowPeak.bed.bed | ENCODE |
| Colon_Cancer | POLR2A | ENCODE: TF | HCT116 | GSM803474 | 46201_sort_peaks.narrowPeak.bed.bed | ENCODE |
| Colon_Cancer | HNF1A | Helix-turn-helix domains | Caco-2 | GSM1655058 | SRX986358.bed | ChIP-Atlas |
| Colon_Cancer | SMARCC1 | Helix-turn-helix domains | HCT116 | GSM1835990 | SRX1123965.bed | ChIP-Atlas |
| Colon_Cancer | SMARCC1 | Helix-turn-helix domains | HCT116 | GSM1835992 | SRX1123967.bed | ChIP-Atlas |
| Colon_Cancer | APC | Helix-turn-helix domains | HCT116 | GSM2785713 | SRX3189055.bed | ChIP-Atlas |
| Colon_Cancer | HSF1 | Helix-turn-helix domains | HCT116 | GSM1382049 | 51107_sort_peaks.narrowPeak.bed.bed | Cistrome |
| Colon_Cancer | HSF1 | Helix-turn-helix domains | HCT116 | GSM1382050 | 51108_sort_peaks.narrowPeak.bed.bed | Cistrome |
| Colon_Cancer | HSF1 | Helix-turn-helix domains | HCT116 | GSM1382052 | 51110_sort_peaks.narrowPeak.bed.bed | Cistrome |
| Colon_Cancer | HSF1 | Helix-turn-helix domains | HCT116 | GSM1382054 | 51112_sort_peaks.narrowPeak.bed.bed | Cistrome |
| Colon_Cancer | HSF1 | Helix-turn-helix domains | HCT116 | GSM1382055 | 51113_sort_peaks.narrowPeak.bed.bed | Cistrome |
| Colon_Cancer | HSF1 | Helix-turn-helix domains | HCT116 | GSM1382057 | 51115_sort_peaks.narrowPeak.bed.bed | Cistrome |
| Colon_Cancer | FOXA2 | Helix-turn-helix domains | Caco-2 | GSM1617408 | 55321_sort_peaks.narrowPeak.bed.bed | Cistrome |
| Colon_Cancer | FOXA2 | Helix-turn-helix domains | Caco-2 | GSM1617409 | 55322_sort_peaks.narrowPeak.bed.bed | Cistrome |
| Colon_Cancer | CDX2 | Helix-turn-helix domains | Caco-2 | GSM575224 | 357_sort_peaks.narrowPeak.bed.bed | Cistrome |
| Colon_Cancer | CDX2 | Helix-turn-helix domains | Caco-2 | GSM575225 | 356_sort_peaks.narrowPeak.bed.bed | Cistrome |
| Colon_Cancer | NCOR1 | Helix-turn-helix domains | LS180 | GSM959631 | 39656_sort_peaks.narrowPeak.bed.bed | Cistrome |
| Colon_Cancer | NCOR1 | Helix-turn-helix domains | LS180 | GSM959632 | 39655_sort_peaks.narrowPeak.bed.bed | Cistrome |
| Colon_Cancer | ARID1A | Helix-turn-helix domains | HCT116 | GSM2719736 | 88980_sort_peaks.narrowPeak.bed.bed | Cistrome |
| Colon_Cancer | ARID1A | Helix-turn-helix domains | HCT116 | GSM2719737 | 88981_sort_peaks.narrowPeak.bed.bed | Cistrome |
| Colon_Cancer | ARID1A | Helix-turn-helix domains | HCT116 | GSM2719738 | 88976_sort_peaks.narrowPeak.bed.bed | Cistrome |
| Colon_Cancer | ARID1A | Helix-turn-helix domains | HCT116 | GSM2719766 | 85741_sort_peaks.narrowPeak.bed.bed | Cistrome |
| Colon_Cancer | ARID1A | Helix-turn-helix domains | HCT116 | GSM2719767 | 85740_sort_peaks.narrowPeak.bed.bed | Cistrome |
| Colon_Cancer | RFX2 | Helix-turn-helix domains | GP5d | GSM1240825 | 42930_sort_peaks.narrowPeak.bed.bed | Cistrome |
| Colon_Cancer | RFX2 | Helix-turn-helix domains | GP5d | GSM1240839 | 42944_sort_peaks.narrowPeak.bed.bed | Cistrome |
| Colon_Cancer | CDX2 | Helix-turn-helix domains | LS174T | GSM2560479 | 75366_sort_peaks.narrowPeak.bed.bed | Cistrome |
| Colon_Cancer | ELF1 | Helix-turn-helix domains | HCT116 | GSM1010765 | 46202_sort_peaks.narrowPeak.bed.bed | ENCODE |
| Colon_Cancer | TEAD4 | Helix-turn-helix domains | HCT116 | GSM1010772 | 46210_sort_peaks.narrowPeak.bed.bed | ENCODE |
| Colon_Cancer | CDX2 | Helix-turn-helix domains | LS180 | GSE31939 | GSE31939.CDX2.LS180_remap2018_hg19__v1_2.bed | Remap |
| Colon_Cancer | NCOR2 | Helix-turn-helix domains | LS180 | GSE39277 | GSE39277.NCOR2.LS180_remap2018_hg19__v1_2.bed | Remap |
| Colon_Cancer | NCOR | Helix-turn-helix domains | LS180 | GSE39277 | GSE39277.NCOR.LS180_remap2018_hg19__v1_2.bed | Remap |
| Colon_Cancer | TP53 | Immunoglobulin fold | HCT116 | GSM2296271 | SRX2060918.bed | ChIP-Atlas |
| Colon_Cancer | TP53 | Immunoglobulin fold | HCT116 | GSM2296272 | SRX2060919.bed | ChIP-Atlas |
| Colon_Cancer | TP53 | Immunoglobulin fold | HCT116 | GSM1412743 | 50344_sort_peaks.narrowPeak.bed.bed | Cistrome |
| Colon_Cancer | TP53 | Immunoglobulin fold | HCT116 | GSM1412744 | 50345_sort_peaks.narrowPeak.bed.bed | Cistrome |
| Colon_Cancer | TP53 | Immunoglobulin fold | HCT116 | GSM1417250 | 50346_sort_peaks.narrowPeak.bed.bed | Cistrome |
| Colon_Cancer | TP53 | Immunoglobulin fold | HCT116 | GSM1468849 | 53285_sort_peaks.narrowPeak.bed.bed | Cistrome |
| Colon_Cancer | TP53 | Immunoglobulin fold | HCT116 | GSM1468850 | 53286_sort_peaks.narrowPeak.bed.bed | Cistrome |
| Colon_Cancer | TP53 | Immunoglobulin fold | HCT116 | GSE74355 | GSE74355.TP53.HCT116_CAMP_remap2018_hg19__v1_2.bed | Remap |
| Colon_Cancer | TP53 | Immunoglobulin fold | HCT116 | GSE74355 | GSE74355.TP53.HCT116_DMSO_KOATF3_remap2018_hg19__v1_2.bed | Remap |
| Colon_Cancer | TCF7L1 | Other all-alpha-helical DNA-binding domains | HCT116 | GSM2065886 | 70804_sort_peaks.narrowPeak.bed.bed | Cistrome |
| Colon_Cancer | TCF7L1 | Other all-alpha-helical DNA-binding domains | HCT116 | GSM2065888 | 70802_sort_peaks.narrowPeak.bed.bed | Cistrome |
| Colon_Cancer | TCF7L1 | Other all-alpha-helical DNA-binding domains | HCT116 | GSM2065901 | 70801_sort_peaks.narrowPeak.bed.bed | Cistrome |
| Colon_Cancer | TCF7L1 | Other all-alpha-helical DNA-binding domains | HCT116 | GSM2065902 | 70800_sort_peaks.narrowPeak.bed.bed | Cistrome |
| Colon_Cancer | TCF7L1 | Other all-alpha-helical DNA-binding domains | HCT116 | GSM2065903 | 70799_sort_peaks.narrowPeak.bed.bed | Cistrome |
| Colon_Cancer | TCF7L2 | Other all-alpha-helical DNA-binding domains | HCT116 | GSM782123 | 45714_sort_peaks.narrowPeak.bed.bed | ENCODE |
| Colon_Cancer | EZH2 | TcoF: TF | HCT116 | GSM1575431 | SRX825988.bed | ChIP-Atlas |
| Colon_Cancer | SMARCA4 | TcoF: TF | HCT116 | GSM1835989 | SRX1123964.bed | ChIP-Atlas |
| Colon_Cancer | SMARCA4 | TcoF: TF | HCT116 | GSM1835991 | SRX1123966.bed | ChIP-Atlas |
| Colon_Cancer | MTA2 | TcoF: TF | HCT116 | GSM969795 | SRX160744.bed | ChIP-Atlas |
| Colon_Cancer | EZH2 | TcoF: TF | HCT116 | GSM969796 | SRX160745.bed | ChIP-Atlas |
| Colon_Cancer | TET2 | TcoF: TF | HCT116 | GSM1152879 | 50981_sort_peaks.narrowPeak.bed.bed | Cistrome |
| Colon_Cancer | MECP2 | TcoF: TF | HCT116 | GSM1154509 | 34399_sort_peaks.narrowPeak.bed.bed | Cistrome |
| Colon_Cancer | MECP2 | TcoF: TF | HCT116 | GSM1154510 | 34400_sort_peaks.narrowPeak.bed.bed | Cistrome |
| Colon_Cancer | AFF4 | TcoF: TF | HCT116 | GSM1162745 | 39916_sort_peaks.narrowPeak.bed.bed | Cistrome |
| Colon_Cancer | AFF4 | TcoF: TF | HCT116 | GSM1727069 | 55639_sort_peaks.narrowPeak.bed.bed | Cistrome |
| Colon_Cancer | AFF4 | TcoF: TF | HCT116 | GSM1727070 | 55640_sort_peaks.narrowPeak.bed.bed | Cistrome |
| Colon_Cancer | AFF4 | TcoF: TF | HCT116 | GSM1727071 | 55641_sort_peaks.narrowPeak.bed.bed | Cistrome |
| Colon_Cancer | AFF4 | TcoF: TF | HCT116 | GSM1727072 | 55642_sort_peaks.narrowPeak.bed.bed | Cistrome |
| Colon_Cancer | AFF4 | TcoF: TF | HCT116 | GSM749819 | 1141_sort_peaks.narrowPeak.bed.bed | Cistrome |
| Colon_Cancer | AFF4 | TcoF: TF | HCT116 | GSM749823 | 1140_sort_peaks.narrowPeak.bed.bed | Cistrome |
| Colon_Cancer | AFF4 | TcoF: TF | HCT116 | GSE30267 | GSE30267.AFF4.HCT116_SERUM_remap2018_hg19__v1_2.bed | Remap |
| Colon_Cancer | AFF4 | TcoF: TF | HCT116 | GSE30267 | GSE30267.AFF4.HCT116_STARVED_remap2018_hg19__v1_2.bed | Remap |
| Colon_Cancer | CREBBP | TcoF: TF | LS180 | GSE39277 | GSE39277.CREBBP.LS180_remap2018_hg19__v1_2.bed | Remap |
| Colon_Cancer | CTCF | Zinc-coordinating DNA-binding domains | HCT116 | GSM2439226 | SRX2442516.bed | ChIP-Atlas |
| Colon_Cancer | ZFX | Zinc-coordinating DNA-binding domains | HCT116 | GSM2741772 | SRX3091912.bed | ChIP-Atlas |
| Colon_Cancer | ZFX | Zinc-coordinating DNA-binding domains | HCT116 | GSM2741773 | SRX3091913.bed | ChIP-Atlas |
| Colon_Cancer | ZC3H8 | Zinc-coordinating DNA-binding domains | HCT116 | GSM1162749 | 39918_sort_peaks.narrowPeak.bed.bed | Cistrome |
| Colon_Cancer | CTCF | Zinc-coordinating DNA-binding domains | HCT116 | GSM1224649 | 42148_sort_peaks.narrowPeak.bed.bed | Cistrome |
| Colon_Cancer | CTCF | Zinc-coordinating DNA-binding domains | HCT116 | GSM1224650 | 42149_sort_peaks.narrowPeak.bed.bed | Cistrome |
| Colon_Cancer | CTCF | Zinc-coordinating DNA-binding domains | HCT116 | GSM1224651 | 42150_sort_peaks.narrowPeak.bed.bed | Cistrome |
| Colon_Cancer | CTCF | Zinc-coordinating DNA-binding domains | HCT116 | GSM1224652 | 42151_sort_peaks.narrowPeak.bed.bed | Cistrome |
| Colon_Cancer | CTCF | Zinc-coordinating DNA-binding domains | HCT116 | GSM1224653 | 42152_sort_peaks.narrowPeak.bed.bed | Cistrome |
| Colon_Cancer | CTCF | Zinc-coordinating DNA-binding domains | HCT116 | GSM1224654 | 42153_sort_peaks.narrowPeak.bed.bed | Cistrome |
| Colon_Cancer | CTCF | Zinc-coordinating DNA-binding domains | HCT116 | GSM1224655 | 42154_sort_peaks.narrowPeak.bed.bed | Cistrome |
| Colon_Cancer | KMT2B | Zinc-coordinating DNA-binding domains | HCT116 | GSM1240109 | 42906_sort_peaks.narrowPeak.bed.bed | Cistrome |
| Colon_Cancer | ZBTB17 | Zinc-coordinating DNA-binding domains | LS174T | GSM1430921 | 49276_sort_peaks.narrowPeak.bed.bed | Cistrome |
| Colon_Cancer | ZBTB17 | Zinc-coordinating DNA-binding domains | LS174T | GSM1430924 | 49279_sort_peaks.narrowPeak.bed.bed | Cistrome |
| Colon_Cancer | HNF4A | Zinc-coordinating DNA-binding domains | HCT116 | GSM1535696 | 54660_sort_peaks.narrowPeak.bed.bed | Cistrome |
| Colon_Cancer | HNF4A | Zinc-coordinating DNA-binding domains | HCT116 | GSM1535701 | 54665_sort_peaks.narrowPeak.bed.bed | Cistrome |
| Colon_Cancer | HNF4A | Zinc-coordinating DNA-binding domains | HCT116 | GSM1535702 | 54666_sort_peaks.narrowPeak.bed.bed | Cistrome |
| Colon_Cancer | HNF4A | Zinc-coordinating DNA-binding domains | HCT116 | GSM1535709 | 54673_sort_peaks.narrowPeak.bed.bed | Cistrome |
| Colon_Cancer | HNF4A | Zinc-coordinating DNA-binding domains | HCT116 | GSM1535710 | 54674_sort_peaks.narrowPeak.bed.bed | Cistrome |
| Colon_Cancer | NR0B2 | Zinc-coordinating DNA-binding domains | HCT116 | GSM1727090 | 55660_sort_peaks.narrowPeak.bed.bed | Cistrome |
| Colon_Cancer | GATA6 | Zinc-coordinating DNA-binding domains | Caco-2 | GSM575226 | 362_sort_peaks.narrowPeak.bed.bed | Cistrome |
| Colon_Cancer | GATA6 | Zinc-coordinating DNA-binding domains | Caco-2 | GSM575227 | 361_sort_peaks.narrowPeak.bed.bed | Cistrome |
| Colon_Cancer | HNF4A | Zinc-coordinating DNA-binding domains | Caco-2 | GSM575228 | 372_sort_peaks.narrowPeak.bed.bed | Cistrome |
| Colon_Cancer | HNF4A | Zinc-coordinating DNA-binding domains | Caco-2 | GSM575229 | 371_sort_peaks.narrowPeak.bed.bed | Cistrome |
| Colon_Cancer | RXRA | Zinc-coordinating DNA-binding domains | LS180 | GSM791405 | 5452_sort_peaks.narrowPeak.bed.bed | Cistrome |
| Colon_Cancer | RXRA | Zinc-coordinating DNA-binding domains | LS180 | GSM791406 | 5453_sort_peaks.narrowPeak.bed.bed | Cistrome |
| Colon_Cancer | SP1 | Zinc-coordinating DNA-binding domains | HCT116 | GSM855444 | 5390_sort_peaks.narrowPeak.bed.bed | Cistrome |
| Colon_Cancer | SP1 | Zinc-coordinating DNA-binding domains | HCT116 | GSM855445 | 5391_sort_peaks.narrowPeak.bed.bed | Cistrome |
| Colon_Cancer | SP1 | Zinc-coordinating DNA-binding domains | HCT116 | GSM855447 | 5393_sort_peaks.narrowPeak.bed.bed | Cistrome |
| Colon_Cancer | SP1 | Zinc-coordinating DNA-binding domains | HCT116 | GSM855449 | 5395_sort_peaks.narrowPeak.bed.bed | Cistrome |
| Colon_Cancer | SP1 | Zinc-coordinating DNA-binding domains | HCT116 | GSM2065874 | 70816_sort_peaks.narrowPeak.bed.bed | Cistrome |
| Colon_Cancer | SP1 | Zinc-coordinating DNA-binding domains | HCT116 | GSM2065875 | 70815_sort_peaks.narrowPeak.bed.bed | Cistrome |
| Colon_Cancer | SP1 | Zinc-coordinating DNA-binding domains | HCT116 | GSM2065876 | 70814_sort_peaks.narrowPeak.bed.bed | Cistrome |
| Colon_Cancer | SP1 | Zinc-coordinating DNA-binding domains | HCT116 | GSM2065877 | 70813_sort_peaks.narrowPeak.bed.bed | Cistrome |
| Colon_Cancer | SP1 | Zinc-coordinating DNA-binding domains | HCT116 | GSM2065878 | 70812_sort_peaks.narrowPeak.bed.bed | Cistrome |
| Colon_Cancer | SP1 | Zinc-coordinating DNA-binding domains | HCT116 | GSM2065879 | 70811_sort_peaks.narrowPeak.bed.bed | Cistrome |
| Colon_Cancer | CTCF | Zinc-coordinating DNA-binding domains | HCT116 | GSM2809613 | 85287_sort_peaks.narrowPeak.bed.bed | Cistrome |
| Colon_Cancer | CTCF | Zinc-coordinating DNA-binding domains | HCT116 | GSM2809614 | 85286_sort_peaks.narrowPeak.bed.bed | Cistrome |
| Colon_Cancer | CTCF | Zinc-coordinating DNA-binding domains | HCT116 | GSM2809615 | 85285_sort_peaks.narrowPeak.bed.bed | Cistrome |
| Colon_Cancer | CTCF | Zinc-coordinating DNA-binding domains | HCT116 | GSM2809616 | 85284_sort_peaks.narrowPeak.bed.bed | Cistrome |
| Colon_Cancer | CTCF | Zinc-coordinating DNA-binding domains | GP5d | GSM1240813 | 42918_sort_peaks.narrowPeak.bed.bed | Cistrome |
| Colon_Cancer | HNF4A | Zinc-coordinating DNA-binding domains | GP5d | GSM1240815 | 42920_sort_peaks.narrowPeak.bed.bed | Cistrome |
| Colon_Cancer | KLF5 | Zinc-coordinating DNA-binding domains | GP5d | GSM1240820 | 42925_sort_peaks.narrowPeak.bed.bed | Cistrome |
| Colon_Cancer | REST | Zinc-coordinating DNA-binding domains | GP5d | GSM1240824 | 42929_sort_peaks.narrowPeak.bed.bed | Cistrome |
| Colon_Cancer | CTCF | Zinc-coordinating DNA-binding domains | GP5d | GSM1240827 | 42932_sort_peaks.narrowPeak.bed.bed | Cistrome |
| Colon_Cancer | HNF4A | Zinc-coordinating DNA-binding domains | GP5d | GSM1240829 | 42934_sort_peaks.narrowPeak.bed.bed | Cistrome |
| Colon_Cancer | KLF5 | Zinc-coordinating DNA-binding domains | GP5d | GSM1240834 | 42939_sort_peaks.narrowPeak.bed.bed | Cistrome |
| Colon_Cancer | REST | Zinc-coordinating DNA-binding domains | GP5d | GSM1240838 | 42943_sort_peaks.narrowPeak.bed.bed | Cistrome |
| Colon_Cancer | CTCF | Zinc-coordinating DNA-binding domains | HCT116 | GSM1022651 | 45717_sort_peaks.narrowPeak.bed.bed | ENCODE |
| Colon_Cancer | CTCF | Zinc-coordinating DNA-binding domains | HCT116 | GSM1022652 | 45716_sort_peaks.narrowPeak.bed.bed | ENCODE |
| Colon_Cancer | EGR1 | Zinc-coordinating DNA-binding domains | HCT116 | GSM1010846 | 46214_sort_peaks.narrowPeak.bed.bed | ENCODE |
| Colon_Cancer | REST | Zinc-coordinating DNA-binding domains | HCT116 | GSM1010869 | 46203_sort_peaks.narrowPeak.bed.bed | ENCODE |
| Colon_Cancer | SP1 | Zinc-coordinating DNA-binding domains | HCT116 | GSM1010902 | 46215_sort_peaks.narrowPeak.bed.bed | ENCODE |
| Colon_Cancer | CTCF | Zinc-coordinating DNA-binding domains | HCT116 | GSM1010903 | 46218_sort_peaks.narrowPeak.bed.bed | ENCODE |
| Colon_Cancer | YY1 | Zinc-coordinating DNA-binding domains | HCT116 | GSM803354 | 46204_sort_peaks.narrowPeak.bed.bed | ENCODE |
| Colon_Cancer | ZBTB33 | Zinc-coordinating DNA-binding domains | HCT116 | GSM803458 | 46200_sort_peaks.narrowPeak.bed.bed | ENCODE |
| Colon_Cancer | CTCF | Zinc-coordinating DNA-binding domains | Caco-2 | GSM749689 | 45577_sort_peaks.narrowPeak.bed.bed | ENCODE |
| Colon_Cancer | CTCF | Zinc-coordinating DNA-binding domains | Caco-2 | GSM749748 | 45578_sort_peaks.narrowPeak.bed.bed | ENCODE |
| Colon_Cancer | RXR | Zinc-coordinating DNA-binding domains | LS180 | GSE31939 | GSE31939.RXR.LS180_remap2018_hg19__v1_2.bed | Remap |
| Colon_Cancer | VDR | Zinc-coordinating DNA-binding domains | LS180 | GSE31939 | GSE31939.VDR.LS180_remap2018_hg19__v1_2.bed | Remap |
| Colon_Cancer | GATA6 | Zinc-coordinating DNA-binding domains | LS174T | GSE49320 | GSE49320.GATA6.LS174T_remap2018_hg19__v1_2.bed | Remap |
| Breast_Cancer | SRF | alpha-Helices exposed by beta-structures | MCF-7 | GSM1010839 | 46312_sort_peaks.narrowPeak.bed.bed | ENCODE |
| Breast_Cancer | MAX | Basic domains | MDA-MB-468 | GSM2151470 | SRX1757996.bed | ChIP-Atlas |
| Breast_Cancer | MAX | Basic domains | MDA-MB-468 | GSM2151471 | SRX1757997.bed | ChIP-Atlas |
| Breast_Cancer | MYC | Basic domains | MDA-MB-468 | GSM2151472 | SRX1757998.bed | ChIP-Atlas |
| Breast_Cancer | MYC | Basic domains | MDA-MB-468 | GSM2151473 | SRX1757999.bed | ChIP-Atlas |
| Breast_Cancer | AHRR | Basic domains | MCF-7 | GSM2406724 | SRX2378692.bed | ChIP-Atlas |
| Breast_Cancer | AHRR | Basic domains | MCF-7 | GSM2406725 | SRX2378693.bed | ChIP-Atlas |
| Breast_Cancer | FOSL1 | Basic domains | MDA-MB-231 | GSM2501565 | SRX2585779.bed | ChIP-Atlas |
| Breast_Cancer | MYC | Basic domains | MDA-MB-231 | GSM2501566 | SRX2585780.bed | ChIP-Atlas |
| Breast_Cancer | TFAP2A | Basic domains | MCF-7 | GSM1081381 | 44122_sort_peaks.narrowPeak.bed.bed | Cistrome |
| Breast_Cancer | XBP1 | Basic domains | MDA-MB-231 | GSM1276189 | 43398_sort_peaks.narrowPeak.bed.bed | Cistrome |
| Breast_Cancer | XBP1 | Basic domains | MDA-MB-231 | GSM1276190 | 43399_sort_peaks.narrowPeak.bed.bed | Cistrome |
| Breast_Cancer | TFAP2C | Basic domains | MCF-7 | GSM1469997 | 49629_sort_peaks.narrowPeak.bed.bed | Cistrome |
| Breast_Cancer | TFAP2C | Basic domains | MCF-7 | GSM1469998 | 49630_sort_peaks.narrowPeak.bed.bed | Cistrome |
| Breast_Cancer | JUN | Basic domains | MDA-MB-231 | GSM1700784 | 56632_sort_peaks.narrowPeak.bed.bed | Cistrome |
| Breast_Cancer | JUN | Basic domains | MDA-MB-231 | GSM1700785 | 56633_sort_peaks.narrowPeak.bed.bed | Cistrome |
| Breast_Cancer | JUN | Basic domains | MDA-MB-231 | GSM1700786 | 56634_sort_peaks.narrowPeak.bed.bed | Cistrome |
| Breast_Cancer | TFAP2A | Basic domains | MCF-7 | GSM588927 | 2402_sort_peaks.narrowPeak.bed.bed | Cistrome |
| Breast_Cancer | TFAP2A | Basic domains | MCF-7 | GSM588928 | 2401_sort_peaks.narrowPeak.bed.bed | Cistrome |
| Breast_Cancer | FOS | Basic domains | MCF-7 | GSM659789 | 2318_sort_peaks.narrowPeak.bed.bed | Cistrome |
| Breast_Cancer | FOS | Basic domains | MCF-7 | GSM659790 | 2317_sort_peaks.narrowPeak.bed.bed | Cistrome |
| Breast_Cancer | JUN | Basic domains | MCF-7 | GSM659791 | 2367_sort_peaks.narrowPeak.bed.bed | Cistrome |
| Breast_Cancer | JUN | Basic domains | MCF-7 | GSM659792 | 2366_sort_peaks.narrowPeak.bed.bed | Cistrome |
| Breast_Cancer | FOSL1 | Basic domains | BT-549 | GSM1125261 | 47287_sort_peaks.narrowPeak.bed.bed | Cistrome |
| Breast_Cancer | JUN | Basic domains | BT-549 | GSM1125262 | 47288_sort_peaks.narrowPeak.bed.bed | Cistrome |
| Breast_Cancer | JUN | Basic domains | BT-549 | GSM1848883 | 67574_sort_peaks.narrowPeak.bed.bed | Cistrome |
| Breast_Cancer | JUN | Basic domains | BT-549 | GSM1848885 | 67573_sort_peaks.narrowPeak.bed.bed | Cistrome |
| Breast_Cancer | AHR | Basic domains | MCF-7 | GSM2406722 | 86160_sort_peaks.narrowPeak.bed.bed | Cistrome |
| Breast_Cancer | AHR | Basic domains | MCF-7 | GSM2406723 | 87747_sort_peaks.narrowPeak.bed.bed | Cistrome |
| Breast_Cancer | NRF1 | Basic domains | MCF-7 | GSM2574812 | 74638_sort_peaks.narrowPeak.bed.bed | Cistrome |
| Breast_Cancer | NRF1 | Basic domains | MCF-7 | GSM2574813 | 74637_sort_peaks.narrowPeak.bed.bed | Cistrome |
| Breast_Cancer | TFAP2C | Basic domains | BT-474 | GSM889426 | 9508_sort_peaks.narrowPeak.bed.bed | Cistrome |
| Breast_Cancer | CREB1 | Basic domains | H3396 | GSM801049 | 5548_sort_peaks.narrowPeak.bed.bed | Cistrome |
| Breast_Cancer | CREB1 | Basic domains | H3396 | GSM801050 | 5549_sort_peaks.narrowPeak.bed.bed | Cistrome |
| Breast_Cancer | CREB1 | Basic domains | H3396 | GSM801051 | 5550_sort_peaks.narrowPeak.bed.bed | Cistrome |
| Breast_Cancer | CREB1 | Basic domains | H3396 | GSM801052 | 5551_sort_peaks.narrowPeak.bed.bed | Cistrome |
| Breast_Cancer | CREB1 | Basic domains | H3396 | GSM801053 | 5552_sort_peaks.narrowPeak.bed.bed | Cistrome |
| Breast_Cancer | CREB1 | Basic domains | H3396 | GSM801054 | 5553_sort_peaks.narrowPeak.bed.bed | Cistrome |
| Breast_Cancer | CREB1 | Basic domains | H3396 | GSM801055 | 5554_sort_peaks.narrowPeak.bed.bed | Cistrome |
| Breast_Cancer | MYC | Basic domains | MDA-MB-453 | GSM1099029 | 36844_sort_peaks.narrowPeak.bed.bed | Cistrome |
| Breast_Cancer | TFAP2C | Basic domains | MDA-MB-453 | GSM889427 | 9509_sort_peaks.narrowPeak.bed.bed | Cistrome |
| Breast_Cancer | TFAP2C | Basic domains | SKBR-3 | GSM889428 | 9510_sort_peaks.narrowPeak.bed.bed | Cistrome |
| Breast_Cancer | CEBPB | Basic domains | SUM159PT | GSM2330568 | 73533_sort_peaks.narrowPeak.bed.bed | Cistrome |
| Breast_Cancer | CEBPB | Basic domains | SUM159PT | GSM2330567 | 73534_sort_peaks.narrowPeak.bed.bed | Cistrome |
| Breast_Cancer | CEBPB | Basic domains | SUM159PT | GSM2330566 | 73535_sort_peaks.narrowPeak.bed.bed | Cistrome |
| Breast_Cancer | CEBPB | Basic domains | SUM159PT | GSM2330565 | 73536_sort_peaks.narrowPeak.bed.bed | Cistrome |
| Breast_Cancer | JUND | Basic domains | T-47D | GSM1010718 | 46372_sort_peaks.narrowPeak.bed.bed | ENCODE |
| Breast_Cancer | FOSL2 | Basic domains | MCF-7 | GSM1010768 | 46317_sort_peaks.narrowPeak.bed.bed | ENCODE |
| Breast_Cancer | TCF12 | Basic domains | MCF-7 | GSM1010861 | 46321_sort_peaks.narrowPeak.bed.bed | ENCODE |
| Breast_Cancer | MAX | Basic domains | MCF-7 | GSM1010863 | 46324_sort_peaks.narrowPeak.bed.bed | ENCODE |
| Breast_Cancer | CEBPB | Basic domains | MCF-7 | GSM1010889 | 46316_sort_peaks.narrowPeak.bed.bed | ENCODE |
| Breast_Cancer | JUND | Basic domains | MCF-7 | GSM1010892 | 46308_sort_peaks.narrowPeak.bed.bed | ENCODE |
| Breast_Cancer | MYC | Basic domains | MCF-7 | GSM822301 | 45989_sort_peaks.narrowPeak.bed.bed | ENCODE |
| Breast_Cancer | MYC | Basic domains | MCF-7 | GSM822304 | 45990_sort_peaks.narrowPeak.bed.bed | ENCODE |
| Breast_Cancer | MYC | Basic domains | MCF-7 | GSM1006866 | 45996_sort_peaks.narrowPeak.bed.bed | ENCODE |
| Breast_Cancer | MYC | Basic domains | MCF-7 | GSM1006877 | 45980_sort_peaks.narrowPeak.bed.bed | ENCODE |
| Breast_Cancer | MYC | Basic domains | MCF-7 | GSM808754 | 38193_sort_peaks.narrowPeak.bed.bed | ENCODE |
| Breast_Cancer | MYC | Basic domains | MCF-7 | GSM808755 | 38183_sort_peaks.narrowPeak.bed.bed | ENCODE |
| Breast_Cancer | CEBPG | Basic domains | MCF-7 | ENCSR094ZCF_1 | 62690_sort_peaks.narrowPeak.bed.bed | ENCODE |
| Breast_Cancer | CEBPG | Basic domains | MCF-7 | ENCSR094ZCF_2 | 62691_sort_peaks.narrowPeak.bed.bed | ENCODE |
| Breast_Cancer | NRF1 | Basic domains | MCF-7 | ENCSR135ANT_1 | 62819_sort_peaks.narrowPeak.bed.bed | ENCODE |
| Breast_Cancer | NRF1 | Basic domains | MCF-7 | ENCSR135ANT_2 | 62820_sort_peaks.narrowPeak.bed.bed | ENCODE |
| Breast_Cancer | SREBF1 | Basic domains | MCF-7 | ENCSR197DJH_1 | 62956_sort_peaks.narrowPeak.bed.bed | ENCODE |
| Breast_Cancer | SREBF1 | Basic domains | MCF-7 | ENCSR197DJH_2 | 62957_sort_peaks.narrowPeak.bed.bed | ENCODE |
| Breast_Cancer | FOSL2 | Basic domains | MCF-7 | ENCSR546KCN_1 | 63858_sort_peaks.narrowPeak.bed.bed | ENCODE |
| Breast_Cancer | FOSL2 | Basic domains | MCF-7 | ENCSR546KCN_2 | 63859_sort_peaks.narrowPeak.bed.bed | ENCODE |
| Breast_Cancer | AHR | Basic domains | MCF-7 | GSM2406726 | EXP038077_liftoverhg19.bed | GTRD |
| Breast_Cancer | AHR | Basic domains | MCF-7 | GSM2406727 | EXP038078_liftoverhg19.bed | GTRD |
| Breast_Cancer | TFAP2C | Basic domains | MCF-7 | GSE21234 | GSE21234.TFAP2C.MCF7_remap2018_hg19__v1_2.bed | Remap |
| Breast_Cancer | TFAP2C | Basic domains | MCF-7 | GSM889425 | GSE36351.TFAP2C.MCF7_remap2018_hg19__v1_2.bed | Remap |
| Breast_Cancer | AHR | Basic domains | MCF-7 | GSE41820 | GSE41820.AHR.MCF7_remap2018_hg19__v1_2.bed | Remap |
| Breast_Cancer | ARNT | Basic domains | MCF-7 | GSE41820 | GSE41820.ARNT.MCF7_remap2018_hg19__v1_2.bed | Remap |
| Breast_Cancer | MYC | Basic domains | MDA-MB-231 | GSE48602 | GSE48602.MYC.MDAMB231_remap2018_hg19__v1_2.bed | Remap |
| Breast_Cancer | XBP1 | Basic domains | T-47D | GSE49952 | GSE49952.XBP1.T47D_HYPO_GLUDEP_remap2018_hg19__v1_2.bed | Remap |
| Breast_Cancer | ARNT | Basic domains | T-47D | GSE59935 | GSE59935.ARNT.T47D_HAEGIN2_remap2018_hg19__v1_2.bed | Remap |
| Breast_Cancer | HIF1A | Basic domains | T-47D | GSE59935 | GSE59935.HIF1A.T47D_HAEGIN2_remap2018_hg19__v1_2.bed | Remap |
| Breast_Cancer | NRF1 | Basic domains | T-47D | GSE59935 | GSE59935.NRF1.T47D_HAEGIN2_remap2018_hg19__v1_2.bed | Remap |
| Breast_Cancer | JUN | Basic domains | MDA-MB-231 | GSE66081 | GSE66081.JUN.MDAMB231_remap2018_hg19__v1_2.bed | Remap |
| Breast_Cancer | NRF1 | Basic domains | HCC1954 | GSE67867 | GSE67867.NRF1.HCC1954_remap2018_hg19__v1_2.bed | Remap |
| Breast_Cancer | YBX1 | beta-Barrel DNA-binding domains | MCF-7 | ENCSR864KNH_1 | 64704_sort_peaks.narrowPeak.bed.bed | ENCODE |
| Breast_Cancer | YBX1 | beta-Barrel DNA-binding domains | MCF-7 | ENCSR864KNH_2 | 64705_sort_peaks.narrowPeak.bed.bed | ENCODE |
| Breast_Cancer | SMAD3 | beta-Hairpin exposed by an alpha/beta-scaffold | MDA-MB-231 | GSM2429817 | SRX2425860.bed | ChIP-Atlas |
| Breast_Cancer | SMAD1 | beta-Hairpin exposed by an alpha/beta-scaffold | MDA-MB-231 | GSM2429818 | SRX2425861.bed | ChIP-Atlas |
| Breast_Cancer | SMAD3 | beta-Hairpin exposed by an alpha/beta-scaffold | MDA-MB-231 | GSM2429819 | SRX2425862.bed | ChIP-Atlas |
| Breast_Cancer | SMAD1 | beta-Hairpin exposed by an alpha/beta-scaffold | MDA-MB-231 | GSM2429820 | SRX2425863.bed | ChIP-Atlas |
| Breast_Cancer | TRIM28 | ENCODE: TF | MCF-7 | GSM1978809 | SRX1497516.bed | ChIP-Atlas |
| Breast_Cancer | EP300 | ENCODE: TF | ZR-75-30 | GSM2029580 | SRX1521303.bed | ChIP-Atlas |
| Breast_Cancer | EP300 | ENCODE: TF | ZR-75-30 | GSM2029581 | SRX1521304.bed | ChIP-Atlas |
| Breast_Cancer | EP300 | ENCODE: TF | MCF-7 | GSM1059392 | 33736_sort_peaks.narrowPeak.bed.bed | Cistrome |
| Breast_Cancer | POLR2A | ENCODE: TF | MCF-7 | GSM1091914 | 47111_sort_peaks.narrowPeak.bed.bed | Cistrome |
| Breast_Cancer | POLR2A | ENCODE: TF | MCF-7 | GSM1091915 | 47112_sort_peaks.narrowPeak.bed.bed | Cistrome |
| Breast_Cancer | POLR2A | ENCODE: TF | MCF-7 | GSM1091916 | 47113_sort_peaks.narrowPeak.bed.bed | Cistrome |
| Breast_Cancer | POLR2A | ENCODE: TF | MCF-7 | GSM1091917 | 47114_sort_peaks.narrowPeak.bed.bed | Cistrome |
| Breast_Cancer | POLR2A | ENCODE: TF | MCF-7 | GSM1091918 | 47115_sort_peaks.narrowPeak.bed.bed | Cistrome |
| Breast_Cancer | POLR2A | ENCODE: TF | MCF-7 | GSM1091919 | 47116_sort_peaks.narrowPeak.bed.bed | Cistrome |
| Breast_Cancer | POLR2A | ENCODE: TF | MCF-7 | GSM1091920 | 47117_sort_peaks.narrowPeak.bed.bed | Cistrome |
| Breast_Cancer | POLR2A | ENCODE: TF | MCF-7 | GSM1091921 | 47118_sort_peaks.narrowPeak.bed.bed | Cistrome |
| Breast_Cancer | POLR2A | ENCODE: TF | MCF-7 | GSM1116655 | 35214_sort_peaks.narrowPeak.bed.bed | Cistrome |
| Breast_Cancer | POLR2A | ENCODE: TF | MCF-7 | GSM1116656 | 35232_sort_peaks.narrowPeak.bed.bed | Cistrome |
| Breast_Cancer | POLR2A | ENCODE: TF | MCF-7 | GSM1116660 | 35215_sort_peaks.narrowPeak.bed.bed | Cistrome |
| Breast_Cancer | POLR2A | ENCODE: TF | MCF-7 | GSM1116661 | 35226_sort_peaks.narrowPeak.bed.bed | Cistrome |
| Breast_Cancer | POLR2A | ENCODE: TF | MCF-7 | GSM1143125 | 41517_sort_peaks.narrowPeak.bed.bed | Cistrome |
| Breast_Cancer | POLR2A | ENCODE: TF | MCF-7 | GSM1276019 | 47786_sort_peaks.narrowPeak.bed.bed | Cistrome |
| Breast_Cancer | POLR2A | ENCODE: TF | MCF-7 | GSM1276020 | 47787_sort_peaks.narrowPeak.bed.bed | Cistrome |
| Breast_Cancer | POLR2A | ENCODE: TF | MCF-7 | GSM1276021 | 47788_sort_peaks.narrowPeak.bed.bed | Cistrome |
| Breast_Cancer | POLR2A | ENCODE: TF | MCF-7 | GSM1276022 | 47789_sort_peaks.narrowPeak.bed.bed | Cistrome |
| Breast_Cancer | POLR2A | ENCODE: TF | MCF-7 | GSM1276023 | 47790_sort_peaks.narrowPeak.bed.bed | Cistrome |
| Breast_Cancer | POLR2A | ENCODE: TF | MCF-7 | GSM1276024 | 47791_sort_peaks.narrowPeak.bed.bed | Cistrome |
| Breast_Cancer | POLR2A | ENCODE: TF | MCF-7 | GSM1276025 | 47792_sort_peaks.narrowPeak.bed.bed | Cistrome |
| Breast_Cancer | POLR2A | ENCODE: TF | MCF-7 | GSM1383863 | 48743_sort_peaks.narrowPeak.bed.bed | Cistrome |
| Breast_Cancer | POLR2A | ENCODE: TF | MCF-7 | GSM1388123 | 48823_sort_peaks.narrowPeak.bed.bed | Cistrome |
| Breast_Cancer | POLR2A | ENCODE: TF | MCF-7 | GSM1388128 | 48828_sort_peaks.narrowPeak.bed.bed | Cistrome |
| Breast_Cancer | POLR2A | ENCODE: TF | MCF-7 | GSM1388129 | 48829_sort_peaks.narrowPeak.bed.bed | Cistrome |
| Breast_Cancer | POLR2A | ENCODE: TF | MCF-7 | GSM1388130 | 48830_sort_peaks.narrowPeak.bed.bed | Cistrome |
| Breast_Cancer | EP300 | ENCODE: TF | MCF-7 | GSM1470013 | 49645_sort_peaks.narrowPeak.bed.bed | Cistrome |
| Breast_Cancer | EP300 | ENCODE: TF | MCF-7 | GSM1470014 | 49646_sort_peaks.narrowPeak.bed.bed | Cistrome |
| Breast_Cancer | EP300 | ENCODE: TF | MCF-7 | GSM1470015 | 49647_sort_peaks.narrowPeak.bed.bed | Cistrome |
| Breast_Cancer | EP300 | ENCODE: TF | MCF-7 | GSM1470016 | 49648_sort_peaks.narrowPeak.bed.bed | Cistrome |
| Breast_Cancer | EP300 | ENCODE: TF | MCF-7 | GSM1523072 | 54636_sort_peaks.narrowPeak.bed.bed | Cistrome |
| Breast_Cancer | EP300 | ENCODE: TF | MCF-7 | GSM1523073 | 54637_sort_peaks.narrowPeak.bed.bed | Cistrome |
| Breast_Cancer | EP300 | ENCODE: TF | MCF-7 | GSM1523074 | 54638_sort_peaks.narrowPeak.bed.bed | Cistrome |
| Breast_Cancer | EP300 | ENCODE: TF | MCF-7 | GSM1523075 | 54639_sort_peaks.narrowPeak.bed.bed | Cistrome |
| Breast_Cancer | EP300 | ENCODE: TF | MCF-7 | GSM1523076 | 54640_sort_peaks.narrowPeak.bed.bed | Cistrome |
| Breast_Cancer | POLR2A | ENCODE: TF | MCF-7 | GSM1523077 | 54641_sort_peaks.narrowPeak.bed.bed | Cistrome |
| Breast_Cancer | POLR2A | ENCODE: TF | MCF-7 | GSM1523078 | 54642_sort_peaks.narrowPeak.bed.bed | Cistrome |
| Breast_Cancer | POLR2A | ENCODE: TF | MCF-7 | GSM1523079 | 54643_sort_peaks.narrowPeak.bed.bed | Cistrome |
| Breast_Cancer | POLR2A | ENCODE: TF | MCF-7 | GSM1523080 | 54644_sort_peaks.narrowPeak.bed.bed | Cistrome |
| Breast_Cancer | POLR2A | ENCODE: TF | MCF-7 | GSM1533404 | 52588_sort_peaks.narrowPeak.bed.bed | Cistrome |
| Breast_Cancer | POLR2A | ENCODE: TF | MCF-7 | GSM1533405 | 52589_sort_peaks.narrowPeak.bed.bed | Cistrome |
| Breast_Cancer | POLR2A | ENCODE: TF | MCF-7 | GSM1533406 | 52590_sort_peaks.narrowPeak.bed.bed | Cistrome |
| Breast_Cancer | POLR2A | ENCODE: TF | MCF-7 | GSM1533407 | 52591_sort_peaks.narrowPeak.bed.bed | Cistrome |
| Breast_Cancer | POLR2A | ENCODE: TF | MCF-7 | GSM1533408 | 52592_sort_peaks.narrowPeak.bed.bed | Cistrome |
| Breast_Cancer | POLR2A | ENCODE: TF | MCF-7 | GSM1533409 | 52593_sort_peaks.narrowPeak.bed.bed | Cistrome |
| Breast_Cancer | POLR2A | ENCODE: TF | MCF-7 | GSM1533410 | 52594_sort_peaks.narrowPeak.bed.bed | Cistrome |
| Breast_Cancer | POLR2A | ENCODE: TF | MCF-7 | GSM1533411 | 52595_sort_peaks.narrowPeak.bed.bed | Cistrome |
| Breast_Cancer | POLR2A | ENCODE: TF | MCF-7 | GSM1533412 | 52596_sort_peaks.narrowPeak.bed.bed | Cistrome |
| Breast_Cancer | POLR2A | ENCODE: TF | MCF-7 | GSM1533413 | 52597_sort_peaks.narrowPeak.bed.bed | Cistrome |
| Breast_Cancer | POLR2A | ENCODE: TF | ZR-75-1 | GSM1537297 | 51805_sort_peaks.narrowPeak.bed.bed | Cistrome |
| Breast_Cancer | POLR2A | ENCODE: TF | MCF-7 | GSM1636933 | 52956_sort_peaks.narrowPeak.bed.bed | Cistrome |
| Breast_Cancer | POLR2A | ENCODE: TF | MCF-7 | GSM1636934 | 52957_sort_peaks.narrowPeak.bed.bed | Cistrome |
| Breast_Cancer | EP300 | ENCODE: TF | T-47D | GSM1669023 | 53967_sort_peaks.narrowPeak.bed.bed | Cistrome |
| Breast_Cancer | EP300 | ENCODE: TF | T-47D | GSM1669024 | 53968_sort_peaks.narrowPeak.bed.bed | Cistrome |
| Breast_Cancer | EP300 | ENCODE: TF | T-47D | GSM1669025 | 53969_sort_peaks.narrowPeak.bed.bed | Cistrome |
| Breast_Cancer | EP300 | ENCODE: TF | T-47D | GSM1669026 | 53970_sort_peaks.narrowPeak.bed.bed | Cistrome |
| Breast_Cancer | EP300 | ENCODE: TF | T-47D | GSM1669027 | 53971_sort_peaks.narrowPeak.bed.bed | Cistrome |
| Breast_Cancer | EP300 | ENCODE: TF | T-47D | GSM1669028 | 53972_sort_peaks.narrowPeak.bed.bed | Cistrome |
| Breast_Cancer | EP300 | ENCODE: TF | T-47D | GSM1669029 | 53973_sort_peaks.narrowPeak.bed.bed | Cistrome |
| Breast_Cancer | EP300 | ENCODE: TF | T-47D | GSM1669030 | 53974_sort_peaks.narrowPeak.bed.bed | Cistrome |
| Breast_Cancer | EP300 | ENCODE: TF | T-47D | GSM1669031 | 53975_sort_peaks.narrowPeak.bed.bed | Cistrome |
| Breast_Cancer | EP300 | ENCODE: TF | MCF-7 | GSM1669041 | 53985_sort_peaks.narrowPeak.bed.bed | Cistrome |
| Breast_Cancer | EP300 | ENCODE: TF | MCF-7 | GSM1669054 | 53998_sort_peaks.narrowPeak.bed.bed | Cistrome |
| Breast_Cancer | EP300 | ENCODE: TF | MCF-7 | GSM1669055 | 53999_sort_peaks.narrowPeak.bed.bed | Cistrome |
| Breast_Cancer | EP300 | ENCODE: TF | MCF-7 | GSM1669056 | 54000_sort_peaks.narrowPeak.bed.bed | Cistrome |
| Breast_Cancer | EP300 | ENCODE: TF | MCF-7 | GSM1669057 | 54001_sort_peaks.narrowPeak.bed.bed | Cistrome |
| Breast_Cancer | EP300 | ENCODE: TF | MCF-7 | GSM1669058 | 54002_sort_peaks.narrowPeak.bed.bed | Cistrome |
| Breast_Cancer | EP300 | ENCODE: TF | MCF-7 | GSM1669059 | 54003_sort_peaks.narrowPeak.bed.bed | Cistrome |
| Breast_Cancer | EP300 | ENCODE: TF | MCF-7 | GSM1669060 | 54004_sort_peaks.narrowPeak.bed.bed | Cistrome |
| Breast_Cancer | EP300 | ENCODE: TF | MCF-7 | GSM1669061 | 54005_sort_peaks.narrowPeak.bed.bed | Cistrome |
| Breast_Cancer | EP300 | ENCODE: TF | MCF-7 | GSM1669062 | 54006_sort_peaks.narrowPeak.bed.bed | Cistrome |
| Breast_Cancer | EP300 | ENCODE: TF | MCF-7 | GSM1669063 | 54007_sort_peaks.narrowPeak.bed.bed | Cistrome |
| Breast_Cancer | EP300 | ENCODE: TF | MCF-7 | GSM1669064 | 54008_sort_peaks.narrowPeak.bed.bed | Cistrome |
| Breast_Cancer | EP300 | ENCODE: TF | MCF-7 | GSM1669065 | 54009_sort_peaks.narrowPeak.bed.bed | Cistrome |
| Breast_Cancer | EP300 | ENCODE: TF | MCF-7 | GSM1669066 | 54010_sort_peaks.narrowPeak.bed.bed | Cistrome |
| Breast_Cancer | POLR2A | ENCODE: TF | MCF-7 | GSM1727113 | 55683_sort_peaks.narrowPeak.bed.bed | Cistrome |
| Breast_Cancer | POLR2A | ENCODE: TF | MCF-7 | GSM365929 | 2361_sort_peaks.narrowPeak.bed.bed | Cistrome |
| Breast_Cancer | POLR2A | ENCODE: TF | MCF-7 | GSM365930 | 2384_sort_peaks.narrowPeak.bed.bed | Cistrome |
| Breast_Cancer | POLR2A | ENCODE: TF | MCF-7 | GSM365931 | 2383_sort_peaks.narrowPeak.bed.bed | Cistrome |
| Breast_Cancer | POLR2A | ENCODE: TF | MCF-7 | GSM365932 | 2382_sort_peaks.narrowPeak.bed.bed | Cistrome |
| Breast_Cancer | POLR2A | ENCODE: TF | MCF-7 | GSM451989 | 2381_sort_peaks.narrowPeak.bed.bed | Cistrome |
| Breast_Cancer | POLR2A | ENCODE: TF | MCF-7 | GSM451990 | 2380_sort_peaks.narrowPeak.bed.bed | Cistrome |
| Breast_Cancer | POLR2A | ENCODE: TF | MCF-7 | GSM451991 | 2379_sort_peaks.narrowPeak.bed.bed | Cistrome |
| Breast_Cancer | POLR2A | ENCODE: TF | MCF-7 | GSM529981 | 2378_sort_peaks.narrowPeak.bed.bed | Cistrome |
| Breast_Cancer | POLR2A | ENCODE: TF | MCF-7 | GSM529982 | 2377_sort_peaks.narrowPeak.bed.bed | Cistrome |
| Breast_Cancer | POLR2A | ENCODE: TF | MCF-7 | GSM588576 | 2376_sort_peaks.narrowPeak.bed.bed | Cistrome |
| Breast_Cancer | POLR2A | ENCODE: TF | MCF-7 | GSM588577 | 2375_sort_peaks.narrowPeak.bed.bed | Cistrome |
| Breast_Cancer | POLR2A | ENCODE: TF | MCF-7 | GSM640420 | 2374_sort_peaks.narrowPeak.bed.bed | Cistrome |
| Breast_Cancer | EP300 | ENCODE: TF | MCF-7 | GSM720424 | 2285_sort_peaks.narrowPeak.bed.bed | Cistrome |
| Breast_Cancer | EP300 | ENCODE: TF | MCF-7 | GSM720425 | 2284_sort_peaks.narrowPeak.bed.bed | Cistrome |
| Breast_Cancer | POLR2A | ENCODE: TF | MCF-7 | GSM832456 | 5796_sort_peaks.narrowPeak.bed.bed | Cistrome |
| Breast_Cancer | POLR2A | ENCODE: TF | MCF-7 | GSM832457 | 5797_sort_peaks.narrowPeak.bed.bed | Cistrome |
| Breast_Cancer | POLR2A | ENCODE: TF | MCF-7 | GSM832458 | 5798_sort_peaks.narrowPeak.bed.bed | Cistrome |
| Breast_Cancer | POLR2A | ENCODE: TF | MCF-7 | GSM840279 | 33482_sort_peaks.narrowPeak.bed.bed | Cistrome |
| Breast_Cancer | POLR2A | ENCODE: TF | HCC1954 | GSM2066439 | 72394_sort_peaks.narrowPeak.bed.bed | Cistrome |
| Breast_Cancer | POLR2A | ENCODE: TF | HCC1954 | GSM2066440 | 72393_sort_peaks.narrowPeak.bed.bed | Cistrome |
| Breast_Cancer | KDM6A | ENCODE: TF | MCF-7 | GSM2549503 | 83698_sort_peaks.narrowPeak.bed.bed | Cistrome |
| Breast_Cancer | POLR2A | ENCODE: TF | MCF-7 | GSM2135493 | 71062_sort_peaks.narrowPeak.bed.bed | Cistrome |
| Breast_Cancer | POLR2A | ENCODE: TF | MCF-7 | GSM2135494 | 71061_sort_peaks.narrowPeak.bed.bed | Cistrome |
| Breast_Cancer | POLR2A | ENCODE: TF | MCF-7 | GSM2135495 | 71060_sort_peaks.narrowPeak.bed.bed | Cistrome |
| Breast_Cancer | POLR2A | ENCODE: TF | MCF-7 | GSM2135496 | 71059_sort_peaks.narrowPeak.bed.bed | Cistrome |
| Breast_Cancer | POLR2A | ENCODE: TF | MCF-7 | GSM2151634 | 72416_sort_peaks.narrowPeak.bed.bed | Cistrome |
| Breast_Cancer | POLR2A | ENCODE: TF | MCF-7 | GSM2151635 | 72415_sort_peaks.narrowPeak.bed.bed | Cistrome |
| Breast_Cancer | POLR2A | ENCODE: TF | MCF-7 | GSM2151636 | 72414_sort_peaks.narrowPeak.bed.bed | Cistrome |
| Breast_Cancer | POLR2A | ENCODE: TF | MCF-7 | GSM2151637 | 72413_sort_peaks.narrowPeak.bed.bed | Cistrome |
| Breast_Cancer | POLR2A | ENCODE: TF | MCF-7 | GSM2151648 | 72412_sort_peaks.narrowPeak.bed.bed | Cistrome |
| Breast_Cancer | POLR2A | ENCODE: TF | MCF-7 | GSM2151649 | 72411_sort_peaks.narrowPeak.bed.bed | Cistrome |
| Breast_Cancer | POLR2A | ENCODE: TF | MCF-7 | GSM2151650 | 72410_sort_peaks.narrowPeak.bed.bed | Cistrome |
| Breast_Cancer | POLR2A | ENCODE: TF | MCF-7 | GSM2151651 | 72409_sort_peaks.narrowPeak.bed.bed | Cistrome |
| Breast_Cancer | POLR2A | ENCODE: TF | MCF-7 | GSM2151660 | 72408_sort_peaks.narrowPeak.bed.bed | Cistrome |
| Breast_Cancer | POLR2A | ENCODE: TF | MCF-7 | GSM2151661 | 72407_sort_peaks.narrowPeak.bed.bed | Cistrome |
| Breast_Cancer | POLR2A | ENCODE: TF | MCF-7 | GSM2151662 | 72406_sort_peaks.narrowPeak.bed.bed | Cistrome |
| Breast_Cancer | POLR2A | ENCODE: TF | MCF-7 | GSM2151663 | 72405_sort_peaks.narrowPeak.bed.bed | Cistrome |
| Breast_Cancer | POLR2A | ENCODE: TF | MCF-7 | GSM2151678 | 72404_sort_peaks.narrowPeak.bed.bed | Cistrome |
| Breast_Cancer | POLR2A | ENCODE: TF | MCF-7 | GSM2151679 | 72403_sort_peaks.narrowPeak.bed.bed | Cistrome |
| Breast_Cancer | POLR2A | ENCODE: TF | MCF-7 | GSM2151680 | 72402_sort_peaks.narrowPeak.bed.bed | Cistrome |
| Breast_Cancer | POLR2A | ENCODE: TF | MCF-7 | GSM2151681 | 72401_sort_peaks.narrowPeak.bed.bed | Cistrome |
| Breast_Cancer | POLR2A | ENCODE: TF | MCF-7 | GSM2151682 | 72400_sort_peaks.narrowPeak.bed.bed | Cistrome |
| Breast_Cancer | POLR2A | ENCODE: TF | MCF-7 | GSM2151683 | 72399_sort_peaks.narrowPeak.bed.bed | Cistrome |
| Breast_Cancer | ZMYM3 | ENCODE: TF | MCF-7 | GSM2574814 | 74636_sort_peaks.narrowPeak.bed.bed | Cistrome |
| Breast_Cancer | EP300 | ENCODE: TF | MCF-7 | GSM986085 | 33128_sort_peaks.narrowPeak.bed.bed | Cistrome |
| Breast_Cancer | EP300 | ENCODE: TF | MCF-7 | GSM986086 | 33126_sort_peaks.narrowPeak.bed.bed | Cistrome |
| Breast_Cancer | EP300 | ENCODE: TF | MCF-7 | GSM986087 | 33154_sort_peaks.narrowPeak.bed.bed | Cistrome |
| Breast_Cancer | EP300 | ENCODE: TF | MCF-7 | GSM986088 | 33155_sort_peaks.narrowPeak.bed.bed | Cistrome |
| Breast_Cancer | POLR2A | ENCODE: TF | MDA-MB-231 | GSM2309434 | 86164_sort_peaks.narrowPeak.bed.bed | Cistrome |
| Breast_Cancer | POLR2A | ENCODE: TF | MDA-MB-231 | GSM2309435 | 86165_sort_peaks.narrowPeak.bed.bed | Cistrome |
| Breast_Cancer | POLR2A | ENCODE: TF | MDA-MB-231 | GSM2913901 | 86992_sort_peaks.narrowPeak.bed.bed | Cistrome |
| Breast_Cancer | POLR2A | ENCODE: TF | MDA-MB-231 | GSM2913902 | 87261_sort_peaks.narrowPeak.bed.bed | Cistrome |
| Breast_Cancer | POLR2A | ENCODE: TF | MDA-MB-231 | GSM2913907 | 86946_sort_peaks.narrowPeak.bed.bed | Cistrome |
| Breast_Cancer | POLR2A | ENCODE: TF | MDA-MB-231 | GSM2913908 | 83423_sort_peaks.narrowPeak.bed.bed | Cistrome |
| Breast_Cancer | POLR2A | ENCODE: TF | T-47D | GSM2644607 | 88432_sort_peaks.narrowPeak.bed.bed | Cistrome |
| Breast_Cancer | POLR2A | ENCODE: TF | T-47D | GSM2644608 | 88437_sort_peaks.narrowPeak.bed.bed | Cistrome |
| Breast_Cancer | POLR2A | ENCODE: TF | T-47D | GSM2644609 | 88436_sort_peaks.narrowPeak.bed.bed | Cistrome |
| Breast_Cancer | POLR2A | ENCODE: TF | T-47D | GSM2644610 | 84351_sort_peaks.narrowPeak.bed.bed | Cistrome |
| Breast_Cancer | POLR2A | ENCODE: TF | T-47D | GSM2644611 | 84352_sort_peaks.narrowPeak.bed.bed | Cistrome |
| Breast_Cancer | POLR2A | ENCODE: TF | T-47D | GSM2644612 | 84349_sort_peaks.narrowPeak.bed.bed | Cistrome |
| Breast_Cancer | POLR2A | ENCODE: TF | T-47D | GSM2644613 | 84350_sort_peaks.narrowPeak.bed.bed | Cistrome |
| Breast_Cancer | POLR2A | ENCODE: TF | T-47D | GSM2644614 | 84348_sort_peaks.narrowPeak.bed.bed | Cistrome |
| Breast_Cancer | POLR2A | ENCODE: TF | T-47D | GSM1155984 | 35938_sort_peaks.narrowPeak.bed.bed | Cistrome |
| Breast_Cancer | POLR2A | ENCODE: TF | H3396 | GSM801061 | 5560_sort_peaks.narrowPeak.bed.bed | Cistrome |
| Breast_Cancer | POLR2A | ENCODE: TF | H3396 | GSM801062 | 5561_sort_peaks.narrowPeak.bed.bed | Cistrome |
| Breast_Cancer | POLR2A | ENCODE: TF | MDA-MB-436 | GSM1537296 | 51804_sort_peaks.narrowPeak.bed.bed | Cistrome |
| Breast_Cancer | POLR2A | ENCODE: TF | SKBR-3 | GSM1537295 | 51803_sort_peaks.narrowPeak.bed.bed | Cistrome |
| Breast_Cancer | EP300 | ENCODE: TF | SUM159PT | GSM2330586 | 73519_sort_peaks.narrowPeak.bed.bed | Cistrome |
| Breast_Cancer | EP300 | ENCODE: TF | SUM159PT | GSM2330585 | 73520_sort_peaks.narrowPeak.bed.bed | Cistrome |
| Breast_Cancer | EP300 | ENCODE: TF | SUM159PT | GSM2330584 | 73521_sort_peaks.narrowPeak.bed.bed | Cistrome |
| Breast_Cancer | EP300 | ENCODE: TF | SUM159PT | GSM2330583 | 73522_sort_peaks.narrowPeak.bed.bed | Cistrome |
| Breast_Cancer | EP300 | ENCODE: TF | SUM159PT | GSM2330560 | 73537_sort_peaks.narrowPeak.bed.bed | Cistrome |
| Breast_Cancer | EP300 | ENCODE: TF | SUM159PT | GSM2330559 | 73538_sort_peaks.narrowPeak.bed.bed | Cistrome |
| Breast_Cancer | EP300 | ENCODE: TF | SUM159PT | GSM2330558 | 73539_sort_peaks.narrowPeak.bed.bed | Cistrome |
| Breast_Cancer | EP300 | ENCODE: TF | SUM159PT | GSM2330557 | 73540_sort_peaks.narrowPeak.bed.bed | Cistrome |
| Breast_Cancer | EP300 | ENCODE: TF | T-47D | GSM803522 | 46370_sort_peaks.narrowPeak.bed.bed | ENCODE |
| Breast_Cancer | EP300 | ENCODE: TF | MCF-7 | GSM1010800 | 46322_sort_peaks.narrowPeak.bed.bed | ENCODE |
| Breast_Cancer | PML | ENCODE: TF | MCF-7 | GSM1010838 | 46307_sort_peaks.narrowPeak.bed.bed | ENCODE |
| Breast_Cancer | POLR2A | ENCODE: TF | MCF-7 | GSM822295 | 45986_sort_peaks.narrowPeak.bed.bed | ENCODE |
| Breast_Cancer | POLR2A | ENCODE: TF | MCF-7 | GSM1006865 | 45993_sort_peaks.narrowPeak.bed.bed | ENCODE |
| Breast_Cancer | POLR2A | ENCODE: TF | MCF-7 | GSM1006876 | 45985_sort_peaks.narrowPeak.bed.bed | ENCODE |
| Breast_Cancer | POLR2A | ENCODE: TF | MCF-7 | GSM808756 | 38201_sort_peaks.narrowPeak.bed.bed | ENCODE |
| Breast_Cancer | POLR2A | ENCODE: TF | MCF-7 | GSM808757 | 38199_sort_peaks.narrowPeak.bed.bed | ENCODE |
| Breast_Cancer | HDGF | ENCODE: TF | MCF-7 | ENCSR200CUA_1 | 62966_sort_peaks.narrowPeak.bed.bed | ENCODE |
| Breast_Cancer | HDGF | ENCODE: TF | MCF-7 | ENCSR200CUA_2 | 62967_sort_peaks.narrowPeak.bed.bed | ENCODE |
| Breast_Cancer | CHD1 | ENCODE: TF | MCF-7 | ENCSR360JOC_1 | 63386_sort_peaks.narrowPeak.bed.bed | ENCODE |
| Breast_Cancer | CHD1 | ENCODE: TF | MCF-7 | ENCSR360JOC_2 | 63387_sort_peaks.narrowPeak.bed.bed | ENCODE |
| Breast_Cancer | NBN | ENCODE: TF | MCF-7 | ENCSR591EBL_1 | 63978_sort_peaks.narrowPeak.bed.bed | ENCODE |
| Breast_Cancer | NBN | ENCODE: TF | MCF-7 | ENCSR591EBL_2 | 63979_sort_peaks.narrowPeak.bed.bed | ENCODE |
| Breast_Cancer | KDM5C | Helix-turn-helix domains | ZR-75-30 | GSM1832638 | SRX1117697.bed | ChIP-Atlas |
| Breast_Cancer | KDM5C | Helix-turn-helix domains | ZR-75-30 | GSM1832639 | SRX1117698.bed | ChIP-Atlas |
| Breast_Cancer | KDM5C | Helix-turn-helix domains | ZR-75-30 | GSM2029587 | SRX1521310.bed | ChIP-Atlas |
| Breast_Cancer | KDM5C | Helix-turn-helix domains | ZR-75-30 | GSM2029592 | SRX1521315.bed | ChIP-Atlas |
| Breast_Cancer | FOXR2 | Helix-turn-helix domains | MDA-MB-468 | GSM2151468 | SRX1757994.bed | ChIP-Atlas |
| Breast_Cancer | FOXR2 | Helix-turn-helix domains | MDA-MB-468 | GSM2151469 | SRX1757995.bed | ChIP-Atlas |
| Breast_Cancer | FOXA1 | Helix-turn-helix domains | T-47D | GSM2242428 | SRX1961157.bed | ChIP-Atlas |
| Breast_Cancer | FOXA1 | Helix-turn-helix domains | T-47D | GSM2242429 | SRX1961158.bed | ChIP-Atlas |
| Breast_Cancer | FOXA1 | Helix-turn-helix domains | T-47D | GSM2242433 | SRX1961163.bed | ChIP-Atlas |
| Breast_Cancer | FOXA1 | Helix-turn-helix domains | T-47D | GSM2242434 | SRX1961164.bed | ChIP-Atlas |
| Breast_Cancer | E2F1 | Helix-turn-helix domains | MDA-MB-231 | GSM2501567 | SRX2585781.bed | ChIP-Atlas |
| Breast_Cancer | FOXM1 | Helix-turn-helix domains | MCF-7 | GSM1000995 | 39443_sort_peaks.narrowPeak.bed.bed | Cistrome |
| Breast_Cancer | FOXM1 | Helix-turn-helix domains | MCF-7 | GSM1000996 | 39495_sort_peaks.narrowPeak.bed.bed | Cistrome |
| Breast_Cancer | FOXM1 | Helix-turn-helix domains | MCF-7 | GSM1000997 | 39484_sort_peaks.narrowPeak.bed.bed | Cistrome |
| Breast_Cancer | FOXM1 | Helix-turn-helix domains | MCF-7 | GSM1000998 | 39498_sort_peaks.narrowPeak.bed.bed | Cistrome |
| Breast_Cancer | FOXM1 | Helix-turn-helix domains | MDA-MB-231 | GSM1000999 | 39460_sort_peaks.narrowPeak.bed.bed | Cistrome |
| Breast_Cancer | FOXM1 | Helix-turn-helix domains | MDA-MB-231 | GSM1001000 | 34997_sort_peaks.narrowPeak.bed.bed | Cistrome |
| Breast_Cancer | FOXM1 | Helix-turn-helix domains | MDA-MB-231 | GSM1001001 | 35011_sort_peaks.narrowPeak.bed.bed | Cistrome |
| Breast_Cancer | FOXM1 | Helix-turn-helix domains | MDA-MB-231 | GSM1001002 | 34999_sort_peaks.narrowPeak.bed.bed | Cistrome |
| Breast_Cancer | FOXM1 | Helix-turn-helix domains | MCF-7 | GSM1001003 | 34998_sort_peaks.narrowPeak.bed.bed | Cistrome |
| Breast_Cancer | FOXM1 | Helix-turn-helix domains | MCF-7 | GSM1001004 | 35006_sort_peaks.narrowPeak.bed.bed | Cistrome |
| Breast_Cancer | FOXM1 | Helix-turn-helix domains | MCF-7 | GSM1001005 | 34995_sort_peaks.narrowPeak.bed.bed | Cistrome |
| Breast_Cancer | FOXM1 | Helix-turn-helix domains | MCF-7 | GSM1001006 | 35002_sort_peaks.narrowPeak.bed.bed | Cistrome |
| Breast_Cancer | E2F4 | Helix-turn-helix domains | MCF-7 | GSM1019122 | 36807_sort_peaks.narrowPeak.bed.bed | Cistrome |
| Breast_Cancer | E2F4 | Helix-turn-helix domains | MCF-7 | GSM1019123 | 36812_sort_peaks.narrowPeak.bed.bed | Cistrome |
| Breast_Cancer | HSF1 | Helix-turn-helix domains | MCF-7 | GSM1116653 | 35218_sort_peaks.narrowPeak.bed.bed | Cistrome |
| Breast_Cancer | HSF1 | Helix-turn-helix domains | MCF-7 | GSM1116654 | 35230_sort_peaks.narrowPeak.bed.bed | Cistrome |
| Breast_Cancer | HSF1 | Helix-turn-helix domains | MCF-7 | GSM1116658 | 35233_sort_peaks.narrowPeak.bed.bed | Cistrome |
| Breast_Cancer | HSF1 | Helix-turn-helix domains | MCF-7 | GSM1116659 | 35222_sort_peaks.narrowPeak.bed.bed | Cistrome |
| Breast_Cancer | KDM5B | Helix-turn-helix domains | MCF-7 | GSM1122651 | 47265_sort_peaks.narrowPeak.bed.bed | Cistrome |
| Breast_Cancer | KDM5B | Helix-turn-helix domains | MCF-7 | GSM1122652 | 47266_sort_peaks.narrowPeak.bed.bed | Cistrome |
| Breast_Cancer | KDM5B | Helix-turn-helix domains | MCF-7 | GSM1122653 | 47267_sort_peaks.narrowPeak.bed.bed | Cistrome |
| Breast_Cancer | SPDEF | Helix-turn-helix domains | MCF-7 | GSM1187119 | 35049_sort_peaks.narrowPeak.bed.bed | Cistrome |
| Breast_Cancer | SPDEF | Helix-turn-helix domains | MCF-7 | GSM1187120 | 35041_sort_peaks.narrowPeak.bed.bed | Cistrome |
| Breast_Cancer | SPDEF | Helix-turn-helix domains | MCF-7 | GSM1187121 | 35052_sort_peaks.narrowPeak.bed.bed | Cistrome |
| Breast_Cancer | SMARCC1 | Helix-turn-helix domains | MCF-7 | GSM1278641 | 44421_sort_peaks.narrowPeak.bed.bed | Cistrome |
| Breast_Cancer | SMARCC1 | Helix-turn-helix domains | MCF-7 | GSM1278643 | 44423_sort_peaks.narrowPeak.bed.bed | Cistrome |
| Breast_Cancer | SMARCC1 | Helix-turn-helix domains | MCF-7 | GSM1278645 | 44425_sort_peaks.narrowPeak.bed.bed | Cistrome |
| Breast_Cancer | SMARCC1 | Helix-turn-helix domains | MCF-7 | GSM1278647 | 44427_sort_peaks.narrowPeak.bed.bed | Cistrome |
| Breast_Cancer | FOXA1 | Helix-turn-helix domains | MCF-7 | GSM1470025 | 49657_sort_peaks.narrowPeak.bed.bed | Cistrome |
| Breast_Cancer | FOXA1 | Helix-turn-helix domains | MCF-7 | GSM1470026 | 49658_sort_peaks.narrowPeak.bed.bed | Cistrome |
| Breast_Cancer | FOXA1 | Helix-turn-helix domains | MCF-7 | GSM1534736 | 52622_sort_peaks.narrowPeak.bed.bed | Cistrome |
| Breast_Cancer | FOXA1 | Helix-turn-helix domains | MCF-7 | GSM1534737 | 52623_sort_peaks.narrowPeak.bed.bed | Cistrome |
| Breast_Cancer | FOXA1 | Helix-turn-helix domains | MCF-7 | GSM1534738 | 52624_sort_peaks.narrowPeak.bed.bed | Cistrome |
| Breast_Cancer | FOXA1 | Helix-turn-helix domains | MCF-7 | GSM1534739 | 52625_sort_peaks.narrowPeak.bed.bed | Cistrome |
| Breast_Cancer | FOXA1 | Helix-turn-helix domains | MCF-7 | GSM1534740 | 52626_sort_peaks.narrowPeak.bed.bed | Cistrome |
| Breast_Cancer | FOXA1 | Helix-turn-helix domains | MCF-7 | GSM1534741 | 52627_sort_peaks.narrowPeak.bed.bed | Cistrome |
| Breast_Cancer | FOXA1 | Helix-turn-helix domains | MCF-7 | GSM1534742 | 52628_sort_peaks.narrowPeak.bed.bed | Cistrome |
| Breast_Cancer | FOXA1 | Helix-turn-helix domains | MCF-7 | GSM1534743 | 52629_sort_peaks.narrowPeak.bed.bed | Cistrome |
| Breast_Cancer | FOXM1 | Helix-turn-helix domains | MCF-7 | GSM1876186 | 57117_sort_peaks.narrowPeak.bed.bed | Cistrome |
| Breast_Cancer | FOXM1 | Helix-turn-helix domains | MCF-7 | GSM1876187 | 57118_sort_peaks.narrowPeak.bed.bed | Cistrome |
| Breast_Cancer | FOXM1 | Helix-turn-helix domains | MCF-7 | GSM1876188 | 57119_sort_peaks.narrowPeak.bed.bed | Cistrome |
| Breast_Cancer | FOXA1 | Helix-turn-helix domains | MCF-7 | GSM588929 | 2322_sort_peaks.narrowPeak.bed.bed | Cistrome |
| Breast_Cancer | FOXA1 | Helix-turn-helix domains | MCF-7 | GSM588930 | 2321_sort_peaks.narrowPeak.bed.bed | Cistrome |
| Breast_Cancer | FOXA1 | Helix-turn-helix domains | MCF-7 | GSM631469 | 6566_sort_peaks.narrowPeak.bed.bed | Cistrome |
| Breast_Cancer | FOXA1 | Helix-turn-helix domains | MCF-7 | GSM631470 | 6567_sort_peaks.narrowPeak.bed.bed | Cistrome |
| Breast_Cancer | FOXA1 | Helix-turn-helix domains | MCF-7 | GSM631471 | 6568_sort_peaks.narrowPeak.bed.bed | Cistrome |
| Breast_Cancer | FOXA1 | Helix-turn-helix domains | ZR-75-1 | GSM631472 | 40376_sort_peaks.narrowPeak.bed.bed | Cistrome |
| Breast_Cancer | FOXA1 | Helix-turn-helix domains | MCF-7 | GSM631474 | 6571_sort_peaks.narrowPeak.bed.bed | Cistrome |
| Breast_Cancer | FOXA1 | Helix-turn-helix domains | MCF-7 | GSM659787 | 2320_sort_peaks.narrowPeak.bed.bed | Cistrome |
| Breast_Cancer | FOXA1 | Helix-turn-helix domains | MCF-7 | GSM659788 | 2319_sort_peaks.narrowPeak.bed.bed | Cistrome |
| Breast_Cancer | FOXA1 | Helix-turn-helix domains | MCF-7 | GSM798436 | 33510_sort_peaks.narrowPeak.bed.bed | Cistrome |
| Breast_Cancer | FOXA1 | Helix-turn-helix domains | MCF-7 | GSM798437 | 33496_sort_peaks.narrowPeak.bed.bed | Cistrome |
| Breast_Cancer | FOXA1 | Helix-turn-helix domains | MCF-7 | GSM798438 | 33518_sort_peaks.narrowPeak.bed.bed | Cistrome |
| Breast_Cancer | FOXA1 | Helix-turn-helix domains | MCF-7 | GSM798439 | 33489_sort_peaks.narrowPeak.bed.bed | Cistrome |
| Breast_Cancer | HSF1 | Helix-turn-helix domains | ZR-75-1 | GSM951870 | 37140_sort_peaks.narrowPeak.bed.bed | Cistrome |
| Breast_Cancer | HSF1 | Helix-turn-helix domains | ZR-75-1 | GSM951871 | 37148_sort_peaks.narrowPeak.bed.bed | Cistrome |
| Breast_Cancer | HSF1 | Helix-turn-helix domains | MCF-7 | GSM951882 | 37137_sort_peaks.narrowPeak.bed.bed | Cistrome |
| Breast_Cancer | HSF1 | Helix-turn-helix domains | MCF-7 | GSM951883 | 37142_sort_peaks.narrowPeak.bed.bed | Cistrome |
| Breast_Cancer | FOXA1 | Helix-turn-helix domains | MCF-7 | GSM2137769 | 69865_sort_peaks.narrowPeak.bed.bed | Cistrome |
| Breast_Cancer | FOXK2 | Helix-turn-helix domains | MCF-7 | GSM2230226 | 71755_sort_peaks.narrowPeak.bed.bed | Cistrome |
| Breast_Cancer | FOXN3 | Helix-turn-helix domains | MCF-7 | GSM2462038 | 82636_sort_peaks.narrowPeak.bed.bed | Cistrome |
| Breast_Cancer | FOXA1 | Helix-turn-helix domains | MCF-7 | GSM1945030 | 71808_sort_peaks.narrowPeak.bed.bed | Cistrome |
| Breast_Cancer | FOXA1 | Helix-turn-helix domains | MCF-7 | GSM2171829 | 73190_sort_peaks.narrowPeak.bed.bed | Cistrome |
| Breast_Cancer | FOXA1 | Helix-turn-helix domains | MCF-7 | GSM2171830 | 73189_sort_peaks.narrowPeak.bed.bed | Cistrome |
| Breast_Cancer | FOXA1 | Helix-turn-helix domains | MCF-7 | GSM2171831 | 73188_sort_peaks.narrowPeak.bed.bed | Cistrome |
| Breast_Cancer | FOXA1 | Helix-turn-helix domains | MCF-7 | GSM2171832 | 73187_sort_peaks.narrowPeak.bed.bed | Cistrome |
| Breast_Cancer | FOXA1 | Helix-turn-helix domains | MCF-7 | GSM2171833 | 73186_sort_peaks.narrowPeak.bed.bed | Cistrome |
| Breast_Cancer | FOXA1 | Helix-turn-helix domains | MCF-7 | GSM2257822 | 72996_sort_peaks.narrowPeak.bed.bed | Cistrome |
| Breast_Cancer | FOXA1 | Helix-turn-helix domains | MCF-7 | GSM2257823 | 72995_sort_peaks.narrowPeak.bed.bed | Cistrome |
| Breast_Cancer | FOXA1 | Helix-turn-helix domains | MCF-7 | GSM2257824 | 72994_sort_peaks.narrowPeak.bed.bed | Cistrome |
| Breast_Cancer | FOXA1 | Helix-turn-helix domains | MCF-7 | GSM2466691 | 73759_sort_peaks.narrowPeak.bed.bed | Cistrome |
| Breast_Cancer | FOXA1 | Helix-turn-helix domains | MCF-7 | GSM2466692 | 73758_sort_peaks.narrowPeak.bed.bed | Cistrome |
| Breast_Cancer | FOXA1 | Helix-turn-helix domains | T-47D | GSM631473 | 6570_sort_peaks.narrowPeak.bed.bed | Cistrome |
| Breast_Cancer | FOXA1 | Helix-turn-helix domains | T-47D | GSM2644575 | 83270_sort_peaks.narrowPeak.bed.bed | Cistrome |
| Breast_Cancer | FOXA1 | Helix-turn-helix domains | T-47D | GSM2644576 | 83269_sort_peaks.narrowPeak.bed.bed | Cistrome |
| Breast_Cancer | FOXA1 | Helix-turn-helix domains | T-47D | GSM2644577 | 83268_sort_peaks.narrowPeak.bed.bed | Cistrome |
| Breast_Cancer | FOXA1 | Helix-turn-helix domains | T-47D | GSM2644578 | 83259_sort_peaks.narrowPeak.bed.bed | Cistrome |
| Breast_Cancer | FOXA1 | Helix-turn-helix domains | T-47D | GSM659795 | 2736_sort_peaks.narrowPeak.bed.bed | Cistrome |
| Breast_Cancer | FOXA1 | Helix-turn-helix domains | T-47D | GSM659796 | 2735_sort_peaks.narrowPeak.bed.bed | Cistrome |
| Breast_Cancer | HOXB7 | Helix-turn-helix domains | BT-474 | GSM1145931 | 53088_sort_peaks.narrowPeak.bed.bed | Cistrome |
| Breast_Cancer | HOXB7 | Helix-turn-helix domains | BT-474 | GSM1145932 | 53089_sort_peaks.narrowPeak.bed.bed | Cistrome |
| Breast_Cancer | HOXB7 | Helix-turn-helix domains | BT-474 | GSM1145933 | 53090_sort_peaks.narrowPeak.bed.bed | Cistrome |
| Breast_Cancer | HOXB7 | Helix-turn-helix domains | BT-474 | GSM1145934 | 53091_sort_peaks.narrowPeak.bed.bed | Cistrome |
| Breast_Cancer | ZEB1 | Helix-turn-helix domains | MDA-231 | GSM2360622 | 87839_sort_peaks.narrowPeak.bed.bed | Cistrome |
| Breast_Cancer | FOXA1 | Helix-turn-helix domains | MDA-MB-453 | GSM1099031 | 36842_sort_peaks.narrowPeak.bed.bed | Cistrome |
| Breast_Cancer | HN1L | Helix-turn-helix domains | SUM159PT | GSM2826102 | 87657_sort_peaks.narrowPeak.bed.bed | Cistrome |
| Breast_Cancer | E2F1 | Helix-turn-helix domains | MCF-7 | GSM935477 | 45991_sort_peaks.narrowPeak.bed.bed | ENCODE |
| Breast_Cancer | FOXA1 | Helix-turn-helix domains | T-47D | GSM803409 | 46368_sort_peaks.narrowPeak.bed.bed | ENCODE |
| Breast_Cancer | E2F1 | Helix-turn-helix domains | MCF-7 | GSM699984 | 2283_sort_peaks.narrowPeak.bed.bed | ENCODE |
| Breast_Cancer | E2F1 | Helix-turn-helix domains | MCF-7 | GSM699985 | 2282_sort_peaks.narrowPeak.bed.bed | ENCODE |
| Breast_Cancer | E2F1 | Helix-turn-helix domains | MCF-7 | GSM699986 | 2281_sort_peaks.narrowPeak.bed.bed | ENCODE |
| Breast_Cancer | E2F1 | Helix-turn-helix domains | MCF-7 | GSM699987 | 2280_sort_peaks.narrowPeak.bed.bed | ENCODE |
| Breast_Cancer | E2F1 | Helix-turn-helix domains | MCF-7 | GSM699988 | 2279_sort_peaks.narrowPeak.bed.bed | ENCODE |
| Breast_Cancer | E2F1 | Helix-turn-helix domains | MCF-7 | GSM699989 | 2278_sort_peaks.narrowPeak.bed.bed | ENCODE |
| Breast_Cancer | ELF1 | Helix-turn-helix domains | MCF-7 | GSM1010764 | 46323_sort_peaks.narrowPeak.bed.bed | ENCODE |
| Breast_Cancer | FOXM1 | Helix-turn-helix domains | MCF-7 | GSM1010769 | 46311_sort_peaks.narrowPeak.bed.bed | ENCODE |
| Breast_Cancer | TEAD4 | Helix-turn-helix domains | MCF-7 | GSM1010860 | 46315_sort_peaks.narrowPeak.bed.bed | ENCODE |
| Breast_Cancer | GABPA | Helix-turn-helix domains | MCF-7 | GSM1010864 | 46314_sort_peaks.narrowPeak.bed.bed | ENCODE |
| Breast_Cancer | CUX1 | Helix-turn-helix domains | MCF-7 | ENCSR017CEO_1 | 62547_sort_peaks.narrowPeak.bed.bed | ENCODE |
| Breast_Cancer | CUX1 | Helix-turn-helix domains | MCF-7 | ENCSR017CEO_2 | 62548_sort_peaks.narrowPeak.bed.bed | ENCODE |
| Breast_Cancer | GABPA | Helix-turn-helix domains | MCF-7 | ENCSR038RXU_1 | 62589_sort_peaks.narrowPeak.bed.bed | ENCODE |
| Breast_Cancer | GABPA | Helix-turn-helix domains | MCF-7 | ENCSR038RXU_2 | 62590_sort_peaks.narrowPeak.bed.bed | ENCODE |
| Breast_Cancer | RFX1 | Helix-turn-helix domains | MCF-7 | ENCSR066TET_1 | 62623_sort_peaks.narrowPeak.bed.bed | ENCODE |
| Breast_Cancer | RFX1 | Helix-turn-helix domains | MCF-7 | ENCSR066TET_2 | 62624_sort_peaks.narrowPeak.bed.bed | ENCODE |
| Breast_Cancer | SIX4 | Helix-turn-helix domains | MCF-7 | ENCSR279IEM_1 | 63186_sort_peaks.narrowPeak.bed.bed | ENCODE |
| Breast_Cancer | SIX4 | Helix-turn-helix domains | MCF-7 | ENCSR279IEM_2 | 63187_sort_peaks.narrowPeak.bed.bed | ENCODE |
| Breast_Cancer | ELK1 | Helix-turn-helix domains | MCF-7 | ENCSR382WLL_1 | 63432_sort_peaks.narrowPeak.bed.bed | ENCODE |
| Breast_Cancer | ELK1 | Helix-turn-helix domains | MCF-7 | ENCSR382WLL_2 | 63433_sort_peaks.narrowPeak.bed.bed | ENCODE |
| Breast_Cancer | RCOR1 | Helix-turn-helix domains | MCF-7 | ENCSR391JII_1 | 63457_sort_peaks.narrowPeak.bed.bed | ENCODE |
| Breast_Cancer | RCOR1 | Helix-turn-helix domains | MCF-7 | ENCSR391JII_2 | 63458_sort_peaks.narrowPeak.bed.bed | ENCODE |
| Breast_Cancer | FOXK2 | Helix-turn-helix domains | MCF-7 | ENCSR465VLK_1 | 63642_sort_peaks.narrowPeak.bed.bed | ENCODE |
| Breast_Cancer | FOXK2 | Helix-turn-helix domains | MCF-7 | ENCSR465VLK_2 | 63643_sort_peaks.narrowPeak.bed.bed | ENCODE |
| Breast_Cancer | ELF1 | Helix-turn-helix domains | MCF-7 | ENCSR475SOC_1 | 63674_sort_peaks.narrowPeak.bed.bed | ENCODE |
| Breast_Cancer | ELF1 | Helix-turn-helix domains | MCF-7 | ENCSR475SOC_2 | 63675_sort_peaks.narrowPeak.bed.bed | ENCODE |
| Breast_Cancer | E2F4 | Helix-turn-helix domains | MCF-7 | ENCSR505NMN_1 | 63739_sort_peaks.narrowPeak.bed.bed | ENCODE |
| Breast_Cancer | E2F4 | Helix-turn-helix domains | MCF-7 | ENCSR505NMN_2 | 63740_sort_peaks.narrowPeak.bed.bed | ENCODE |
| Breast_Cancer | RFX1 | Helix-turn-helix domains | MCF-7 | ENCSR788XNX_1 | 64525_sort_peaks.narrowPeak.bed.bed | ENCODE |
| Breast_Cancer | RFX1 | Helix-turn-helix domains | MCF-7 | ENCSR788XNX_2 | 64526_sort_peaks.narrowPeak.bed.bed | ENCODE |
| Breast_Cancer | ZEB1 | Helix-turn-helix domains | MCF-7 | GSM2590071 | EXP038582_liftoverhg19.bed | GTRD |
| Breast_Cancer | PBX1 | Helix-turn-helix domains | MCF-7 | GSM989353 | EXP031466_liftoverhg19.bed | GTRD |
| Breast_Cancer | ERG | Helix-turn-helix domains | MCF-7 | GSE23730 | GSE23730.ERG.MCF7_remap2018_hg19__v1_2.bed | Remap |
| Breast_Cancer | ELF5 | Helix-turn-helix domains | T-47D | GSE31216 | GSE31216.ELF5.T47D_ELF5_48H_remap2018_hg19__v1_2.bed | Remap |
| Breast_Cancer | FOXA1 | Helix-turn-helix domains | MCF-7 | GSE40129 | GSE40129.FOXA1.MCF7_SICTR_remap2018_hg19__v1_2.bed | Remap |
| Breast_Cancer | FOXA1 | Helix-turn-helix domains | MCF-7 | GSE40129 | GSE40129.FOXA1.MCF7_SIGATA_remap2018_hg19__v1_2.bed | Remap |
| Breast_Cancer | HSF1 | Helix-turn-helix domains | MCF-7 | GSE45852 | GSE45852.HSF1.MCF7_remap2018_hg19__v1_2.bed | Remap |
| Breast_Cancer | KDM5B | Helix-turn-helix domains | MDA-MB-231 | GSM1122669 | GSE46055.KDM5B.MDAMB231_remap2018_hg19__v1_2.bed | Remap |
| Breast_Cancer | KDM5B | Helix-turn-helix domains | SUM159 | GSM1122664 | GSE46055.KDM5B.SUM159_remap2018_hg19__v1_2.bed | Remap |
| Breast_Cancer | KDM5B | Helix-turn-helix domains | T-47D | GSE46055 | GSE46055.KDM5B.T47D_remap2018_hg19__v1_2.bed | Remap |
| Breast_Cancer | HOXC11 | Helix-turn-helix domains | MCF-7 | GSE54027 | GSE54027.HOXC11.MCF7_LY2_remap2018_hg19__v1_2.bed | Remap |
| Breast_Cancer | HOXC11 | Helix-turn-helix domains | MCF-7 | GSE54027 | GSE54027.HOXC11.MCF7_LY2_TAMOXIFEN_remap2018_hg19__v1_2.bed | Remap |
| Breast_Cancer | TEAD4 | Helix-turn-helix domains | MDA-MB-231 | GSE66081 | GSE66081.TEAD4.MDAMB231_remap2018_hg19__v1_2.bed | Remap |
| Breast_Cancer | GABPA | Helix-turn-helix domains | MCF-7 | GSE72082 | GSE72082.GABPA.MCF7_remap2018_hg19__v1_2.bed | Remap |
| Breast_Cancer | FOXA1 | Helix-turn-helix domains | MCF-7 | GSE72249 | GSE72249.FOXA1.MCF7_remap2018_hg19__v1_2.bed | Remap |
| Breast_Cancer | FOXA1 | Helix-turn-helix domains | T-47D | GSE72249 | GSE72249.FOXA1.T47D_remap2018_hg19__v1_2.bed | Remap |
| Breast_Cancer | FOXA1 | Helix-turn-helix domains | ZR-75-1 | GSE72249 | GSE72249.FOXA1.ZR751_remap2018_hg19__v1_2.bed | Remap |
| Breast_Cancer | KDM5A | Helix-turn-helix domains | T-47D | GSE80593 | GSE80593.KDM5A.T47D_DMSO_remap2018_hg19__v1_2.bed | Remap |
| Breast_Cancer | KDM5A | Helix-turn-helix domains | T-47D | GSE80593 | GSE80593.KDM5A.T47D_MK2206_remap2018_hg19__v1_2.bed | Remap |
| Breast_Cancer | FOXA1 | Helix-turn-helix domains | MCF-7 | GSE80808 | GSE80808.FOXA1.MCF7_remap2018_hg19__v1_2.bed | Remap |
| Breast_Cancer | RUNX1 | Immunoglobulin fold | MCF-7 | GSM1592326 | SRX852985.bed | ChIP-Atlas |
| Breast_Cancer | RUNX1 | Immunoglobulin fold | MCF-7 | GSM1592327 | SRX852986.bed | ChIP-Atlas |
| Breast_Cancer | GRHL2 | Immunoglobulin fold | MCF-7 | GSM2171834 | SRX1787656.bed | ChIP-Atlas |
| Breast_Cancer | GRHL2 | Immunoglobulin fold | MCF-7 | GSM2171835 | SRX1787657.bed | ChIP-Atlas |
| Breast_Cancer | GRHL2 | Immunoglobulin fold | MCF-7 | GSM2171836 | SRX1787658.bed | ChIP-Atlas |
| Breast_Cancer | STAT1 | Immunoglobulin fold | MCF-7 | GSM2176791 | SRX1799015.bed | ChIP-Atlas |
| Breast_Cancer | STAT3 | Immunoglobulin fold | MDA-MB-231 | GSM2278002 | SRX2020840.bed | ChIP-Atlas |
| Breast_Cancer | STAT3 | Immunoglobulin fold | MDA-MB-231 | GSM2278003 | SRX2020841.bed | ChIP-Atlas |
| Breast_Cancer | STAT3 | Immunoglobulin fold | MDA-MB-468 | GSM2278008 | SRX2020846.bed | ChIP-Atlas |
| Breast_Cancer | STAT3 | Immunoglobulin fold | MDA-MB-468 | GSM2278009 | SRX2020847.bed | ChIP-Atlas |
| Breast_Cancer | TP53 | Immunoglobulin fold | MDA-MB-231 | GSM2501568 | SRX2585782.bed | ChIP-Atlas |
| Breast_Cancer | RBPJ | Immunoglobulin fold | MDA-MB-231 | GSM2679734 | SRX2947200.bed | ChIP-Atlas |
| Breast_Cancer | TP53 | Immunoglobulin fold | MCF-7 | GSM1429753 | 54519_sort_peaks.narrowPeak.bed.bed | Cistrome |
| Breast_Cancer | TP53 | Immunoglobulin fold | MDA-MB-468 | GSM1429755 | 54521_sort_peaks.narrowPeak.bed.bed | Cistrome |
| Breast_Cancer | RELA | Immunoglobulin fold | MCF-7 | GSM1534728 | 52614_sort_peaks.narrowPeak.bed.bed | Cistrome |
| Breast_Cancer | RELA | Immunoglobulin fold | MCF-7 | GSM1534729 | 52615_sort_peaks.narrowPeak.bed.bed | Cistrome |
| Breast_Cancer | RELA | Immunoglobulin fold | MCF-7 | GSM1534730 | 52616_sort_peaks.narrowPeak.bed.bed | Cistrome |
| Breast_Cancer | RELA | Immunoglobulin fold | MCF-7 | GSM1534731 | 52617_sort_peaks.narrowPeak.bed.bed | Cistrome |
| Breast_Cancer | RELA | Immunoglobulin fold | MCF-7 | GSM1534732 | 52618_sort_peaks.narrowPeak.bed.bed | Cistrome |
| Breast_Cancer | RELA | Immunoglobulin fold | MCF-7 | GSM1534733 | 52619_sort_peaks.narrowPeak.bed.bed | Cistrome |
| Breast_Cancer | RELA | Immunoglobulin fold | MCF-7 | GSM1534734 | 52620_sort_peaks.narrowPeak.bed.bed | Cistrome |
| Breast_Cancer | RELA | Immunoglobulin fold | MCF-7 | GSM1534735 | 52621_sort_peaks.narrowPeak.bed.bed | Cistrome |
| Breast_Cancer | RELA | Immunoglobulin fold | MDA-MB-231 | GSM1700787 | 56635_sort_peaks.narrowPeak.bed.bed | Cistrome |
| Breast_Cancer | RELA | Immunoglobulin fold | MDA-MB-231 | GSM1700788 | 56636_sort_peaks.narrowPeak.bed.bed | Cistrome |
| Breast_Cancer | RELA | Immunoglobulin fold | MDA-MB-231 | GSM1700789 | 56637_sort_peaks.narrowPeak.bed.bed | Cistrome |
| Breast_Cancer | TP53 | Immunoglobulin fold | BT-549 | GSM1429757 | 54523_sort_peaks.narrowPeak.bed.bed | Cistrome |
| Breast_Cancer | TP53 | Immunoglobulin fold | MCF-7 | GSM2714249 | 85923_sort_peaks.narrowPeak.bed.bed | Cistrome |
| Breast_Cancer | RELA | Immunoglobulin fold | MCF-7 | GSM1643956 | 74392_sort_peaks.narrowPeak.bed.bed | Cistrome |
| Breast_Cancer | RELA | Immunoglobulin fold | MCF-7 | GSM1643957 | 74391_sort_peaks.narrowPeak.bed.bed | Cistrome |
| Breast_Cancer | RELA | Immunoglobulin fold | MCF-7 | GSM1643958 | 74390_sort_peaks.narrowPeak.bed.bed | Cistrome |
| Breast_Cancer | RELA | Immunoglobulin fold | MCF-7 | GSM1643959 | 74389_sort_peaks.narrowPeak.bed.bed | Cistrome |
| Breast_Cancer | RELA | Immunoglobulin fold | MCF-7 | GSM1643960 | 74388_sort_peaks.narrowPeak.bed.bed | Cistrome |
| Breast_Cancer | RELA | Immunoglobulin fold | MCF-7 | GSM1643961 | 74387_sort_peaks.narrowPeak.bed.bed | Cistrome |
| Breast_Cancer | RELA | Immunoglobulin fold | MCF-7 | GSM1643962 | 74386_sort_peaks.narrowPeak.bed.bed | Cistrome |
| Breast_Cancer | RELA | Immunoglobulin fold | MCF-7 | GSM1643963 | 74385_sort_peaks.narrowPeak.bed.bed | Cistrome |
| Breast_Cancer | RELA | Immunoglobulin fold | MCF-7 | GSM1644000 | 74382_sort_peaks.narrowPeak.bed.bed | Cistrome |
| Breast_Cancer | RUNX1 | Immunoglobulin fold | MCF-7 | GSM1942111 | 70985_sort_peaks.narrowPeak.bed.bed | Cistrome |
| Breast_Cancer | RUNX1 | Immunoglobulin fold | MCF-7 | GSM1942113 | 70984_sort_peaks.narrowPeak.bed.bed | Cistrome |
| Breast_Cancer | RUNX2 | Immunoglobulin fold | MCF-7 | GSM2305318 | 74117_sort_peaks.narrowPeak.bed.bed | Cistrome |
| Breast_Cancer | RUNX2 | Immunoglobulin fold | MCF-7 | GSM2305319 | 74116_sort_peaks.narrowPeak.bed.bed | Cistrome |
| Breast_Cancer | TP53 | Immunoglobulin fold | MCF-7 | GSM2944126 | 87304_sort_peaks.narrowPeak.bed.bed | Cistrome |
| Breast_Cancer | TP53 | Immunoglobulin fold | MCF-7 | GSM2944127 | 87303_sort_peaks.narrowPeak.bed.bed | Cistrome |
| Breast_Cancer | TP53 | Immunoglobulin fold | MCF-7 | GSM2944128 | 87301_sort_peaks.narrowPeak.bed.bed | Cistrome |
| Breast_Cancer | TP53 | Immunoglobulin fold | MCF-7 | GSM2944129 | 87300_sort_peaks.narrowPeak.bed.bed | Cistrome |
| Breast_Cancer | GRHL2 | Immunoglobulin fold | MCF-7 | GSM2970414 | 87112_sort_peaks.narrowPeak.bed.bed | Cistrome |
| Breast_Cancer | GRHL2 | Immunoglobulin fold | MCF-7 | GSM2970415 | 85047_sort_peaks.narrowPeak.bed.bed | Cistrome |
| Breast_Cancer | GRHL2 | Immunoglobulin fold | MCF-7 | GSM2970416 | 87110_sort_peaks.narrowPeak.bed.bed | Cistrome |
| Breast_Cancer | GRHL2 | Immunoglobulin fold | MCF-7 | GSM2970417 | 87111_sort_peaks.narrowPeak.bed.bed | Cistrome |
| Breast_Cancer | GRHL2 | Immunoglobulin fold | MCF-7 | GSM2970418 | 87118_sort_peaks.narrowPeak.bed.bed | Cistrome |
| Breast_Cancer | GRHL2 | Immunoglobulin fold | MCF-7 | GSM2970419 | 87119_sort_peaks.narrowPeak.bed.bed | Cistrome |
| Breast_Cancer | T | Immunoglobulin fold | T-47D | GSM2476829 | 74832_sort_peaks.narrowPeak.bed.bed | Cistrome |
| Breast_Cancer | T | Immunoglobulin fold | T-47D | GSM2476830 | 74831_sort_peaks.narrowPeak.bed.bed | Cistrome |
| Breast_Cancer | T | Immunoglobulin fold | T-47D | GSM2476831 | 74830_sort_peaks.narrowPeak.bed.bed | Cistrome |
| Breast_Cancer | STAT3 | Immunoglobulin fold | HCC1143 | GSM2278010 | 88680_sort_peaks.narrowPeak.bed.bed | Cistrome |
| Breast_Cancer | TP53 | Immunoglobulin fold | HCC70 | GSM1429756 | 54522_sort_peaks.narrowPeak.bed.bed | Cistrome |
| Breast_Cancer | STAT3 | Immunoglobulin fold | HCC70 | GSM2278004 | 84620_sort_peaks.narrowPeak.bed.bed | Cistrome |
| Breast_Cancer | STAT3 | Immunoglobulin fold | HCC70 | GSM2278005 | 84621_sort_peaks.narrowPeak.bed.bed | Cistrome |
| Breast_Cancer | STAT3 | Immunoglobulin fold | MDA-MB-157 | GSM2278000 | 84616_sort_peaks.narrowPeak.bed.bed | Cistrome |
| Breast_Cancer | STAT3 | Immunoglobulin fold | MDA-MB-157 | GSM2278001 | 84617_sort_peaks.narrowPeak.bed.bed | Cistrome |
| Breast_Cancer | TP53 | Immunoglobulin fold | MDA-MB-175VII | GSM1429754 | 54520_sort_peaks.narrowPeak.bed.bed | Cistrome |
| Breast_Cancer | TP53 | Immunoglobulin fold | MCF-7 | GSM2296274 | EXP037748_liftoverhg19.bed | GTRD |
| Breast_Cancer | TP53 | Immunoglobulin fold | MCF-7 | GSM2296275 | EXP037749_liftoverhg19.bed | GTRD |
| Breast_Cancer | TP53 | Immunoglobulin fold | MCF-7 | GSM2671290 | EXP036824_liftoverhg19.bed | GTRD |
| Breast_Cancer | TP53 | Immunoglobulin fold | MCF-7 | GSM2671291 | EXP036825_liftoverhg19.bed | GTRD |
| Breast_Cancer | TP53 | Immunoglobulin fold | MCF-7 | GSM2671292 | EXP036826_liftoverhg19.bed | GTRD |
| Breast_Cancer | TP53 | Immunoglobulin fold | MCF-7 | GSM2671293 | EXP036827_liftoverhg19.bed | GTRD |
| Breast_Cancer | TP53 | Immunoglobulin fold | MCF-7 | GSM2671294 | EXP036828_liftoverhg19.bed | GTRD |
| Breast_Cancer | TP53 | Immunoglobulin fold | MCF-7 | GSM2671295 | EXP036829_liftoverhg19.bed | GTRD |
| Breast_Cancer | TP53 | Immunoglobulin fold | MCF-7 | GSM2671296 | EXP036830_liftoverhg19.bed | GTRD |
| Breast_Cancer | TP53 | Immunoglobulin fold | MCF-7 | GSM2714248 | EXP036872_liftoverhg19.bed | GTRD |
| Breast_Cancer | TP53 | Immunoglobulin fold | MCF-7 | GSM2714252 | EXP036875_liftoverhg19.bed | GTRD |
| Breast_Cancer | TP53 | Immunoglobulin fold | MCF-7 | GSM1146168 | GSE47041.TP53.MCF7_NUT_remap2018_hg19__v1_2.bed | Remap |
| Breast_Cancer | TP63 | Immunoglobulin fold | MDA-MB-231 | GSE72009 | GSE72009.TP63.MDAMB231_remap2018_hg19__v1_2.bed | Remap |
| Breast_Cancer | RUNX1 | Immunoglobulin fold | MCF-7 | GSE75070 | GSE75070.RUNX1.MCF7_remap2018_hg19__v1_2.bed | Remap |
| Breast_Cancer | TCF7L2 | Other all-alpha-helical DNA-binding domains | MDA-MB-453 | GSM1099033 | 36841_sort_peaks.narrowPeak.bed.bed | Cistrome |
| Breast_Cancer | TCF7L2 | Other all-alpha-helical DNA-binding domains | MDA-MB-453 | GSM1099032 | 36843_sort_peaks.narrowPeak.bed.bed | Cistrome |
| Breast_Cancer | TCF7L2 | Other all-alpha-helical DNA-binding domains | MCF-7 | GSM816438 | 45979_sort_peaks.narrowPeak.bed.bed | ENCODE |
| Breast_Cancer | TAF1 | Other all-alpha-helical DNA-binding domains | MCF-7 | GSM1010811 | 46320_sort_peaks.narrowPeak.bed.bed | ENCODE |
| Breast_Cancer | BPTF | TcoF: TF | T-47D | GSM1571861 | SRX822004.bed | ChIP-Atlas |
| Breast_Cancer | BPTF | TcoF: TF | T-47D | GSM1571862 | SRX822005.bed | ChIP-Atlas |
| Breast_Cancer | KMT2C | TcoF: TF | MCF-7 | GSM2171853 | SRX1787675.bed | ChIP-Atlas |
| Breast_Cancer | KMT2C | TcoF: TF | MCF-7 | GSM2171854 | SRX1787676.bed | ChIP-Atlas |
| Breast_Cancer | KMT2C | TcoF: TF | MCF-7 | GSM2171855 | SRX1787677.bed | ChIP-Atlas |
| Breast_Cancer | KMT2C | TcoF: TF | MCF-7 | GSM2171856 | SRX1787678.bed | ChIP-Atlas |
| Breast_Cancer | KMT2C | TcoF: TF | MCF-7 | GSM2171857 | SRX1787679.bed | ChIP-Atlas |
| Breast_Cancer | KMT2C | TcoF: TF | MCF-7 | GSM2171858 | SRX1787680.bed | ChIP-Atlas |
| Breast_Cancer | KMT2C | TcoF: TF | MCF-7 | GSM2171859 | SRX1787681.bed | ChIP-Atlas |
| Breast_Cancer | KMT2C | TcoF: TF | MCF-7 | GSM2171860 | SRX1787682.bed | ChIP-Atlas |
| Breast_Cancer | KMT2C | TcoF: TF | MCF-7 | GSM2171861 | SRX1787683.bed | ChIP-Atlas |
| Breast_Cancer | KMT2C | TcoF: TF | MCF-7 | GSM2171862 | SRX1787684.bed | ChIP-Atlas |
| Breast_Cancer | EZH2 | TcoF: TF | MDA-MB-231 | GSM2501569 | SRX2585783.bed | ChIP-Atlas |
| Breast_Cancer | L3MBTL3 | TcoF: TF | MDA-MB-231 | GSM2679735 | SRX2947201.bed | ChIP-Atlas |
| Breast_Cancer | MBD2 | TcoF: TF | MCF-7 | GSM1322266 | 48127_sort_peaks.narrowPeak.bed.bed | Cistrome |
| Breast_Cancer | MBD2 | TcoF: TF | MCF-7 | GSM1322268 | 48128_sort_peaks.narrowPeak.bed.bed | Cistrome |
| Breast_Cancer | MBD2 | TcoF: TF | MCF-7 | GSM1322270 | 48130_sort_peaks.narrowPeak.bed.bed | Cistrome |
| Breast_Cancer | MBD2 | TcoF: TF | MCF-7 | GSM1388122 | 48822_sort_peaks.narrowPeak.bed.bed | Cistrome |
| Breast_Cancer | MBD2 | TcoF: TF | MCF-7 | GSM1388124 | 48824_sort_peaks.narrowPeak.bed.bed | Cistrome |
| Breast_Cancer | MBD2 | TcoF: TF | MCF-7 | GSM1388125 | 48825_sort_peaks.narrowPeak.bed.bed | Cistrome |
| Breast_Cancer | MBD2 | TcoF: TF | MCF-7 | GSM1388126 | 48826_sort_peaks.narrowPeak.bed.bed | Cistrome |
| Breast_Cancer | MBD2 | TcoF: TF | MCF-7 | GSM1388127 | 48827_sort_peaks.narrowPeak.bed.bed | Cistrome |
| Breast_Cancer | PARP1 | TcoF: TF | MCF-7 | GSM1517305 | 56235_sort_peaks.narrowPeak.bed.bed | Cistrome |
| Breast_Cancer | MTA3 | TcoF: TF | MCF-7 | GSM1642517 | 52966_sort_peaks.narrowPeak.bed.bed | Cistrome |
| Breast_Cancer | MBD3 | TcoF: TF | MDA-MB-231 | GSM1974448 | 59412_sort_peaks.narrowPeak.bed.bed | Cistrome |
| Breast_Cancer | MBD3 | TcoF: TF | MDA-MB-231 | GSM1974449 | 59413_sort_peaks.narrowPeak.bed.bed | Cistrome |
| Breast_Cancer | WDHD1 | TcoF: TF | MCF-7 | GSM2574806 | 77260_sort_peaks.narrowPeak.bed.bed | Cistrome |
| Breast_Cancer | WDHD1 | TcoF: TF | MCF-7 | GSM2574807 | 77259_sort_peaks.narrowPeak.bed.bed | Cistrome |
| Breast_Cancer | DPF1 | TcoF: TF | MCF-7 | GSM2574811 | 77255_sort_peaks.narrowPeak.bed.bed | Cistrome |
| Breast_Cancer | PARP1 | TcoF: TF | MDA-MB-231 | GSM1517306 | 56236_sort_peaks.narrowPeak.bed.bed | Cistrome |
| Breast_Cancer | SMARCA4 | TcoF: TF | MDA-MB-231 | GSM1856032 | 67498_sort_peaks.narrowPeak.bed.bed | Cistrome |
| Breast_Cancer | SMARCA4 | TcoF: TF | MDA-MB-231 | GSM1856033 | 67497_sort_peaks.narrowPeak.bed.bed | Cistrome |
| Breast_Cancer | SMARCA4 | TcoF: TF | MDA-MB-231 | GSM1856034 | 67496_sort_peaks.narrowPeak.bed.bed | Cistrome |
| Breast_Cancer | PARP1 | TcoF: TF | T-47D | GSM1302194 | 43678_sort_peaks.narrowPeak.bed.bed | Cistrome |
| Breast_Cancer | PARP1 | TcoF: TF | T-47D | GSM1302195 | 43679_sort_peaks.narrowPeak.bed.bed | Cistrome |
| Breast_Cancer | MBD3 | TcoF: TF | MDA-231 | GSM1089819 | 41402_sort_peaks.narrowPeak.bed.bed | Cistrome |
| Breast_Cancer | PARP1 | TcoF: TF | T-47D | GSM1302196 | 43680_sort_peaks.narrowPeak.bed.bed | Cistrome |
| Breast_Cancer | ZNF207 | TcoF: TF | MCF-7 | ENCSR096KWU_1 | 62696_sort_peaks.narrowPeak.bed.bed | ENCODE |
| Breast_Cancer | ZNF207 | TcoF: TF | MCF-7 | ENCSR096KWU_2 | 62697_sort_peaks.narrowPeak.bed.bed | ENCODE |
| Breast_Cancer | DPF2 | TcoF: TF | MCF-7 | ENCSR234VCE_1 | 63060_sort_peaks.narrowPeak.bed.bed | ENCODE |
| Breast_Cancer | DPF2 | TcoF: TF | MCF-7 | ENCSR234VCE_2 | 63061_sort_peaks.narrowPeak.bed.bed | ENCODE |
| Breast_Cancer | MTA3 | TcoF: TF | MCF-7 | ENCSR391KQC_1 | 63459_sort_peaks.narrowPeak.bed.bed | ENCODE |
| Breast_Cancer | MTA3 | TcoF: TF | MCF-7 | ENCSR391KQC_2 | 63460_sort_peaks.narrowPeak.bed.bed | ENCODE |
| Breast_Cancer | MLLT1 | TcoF: TF | MCF-7 | ENCSR427BBI_1 | 63556_sort_peaks.narrowPeak.bed.bed | ENCODE |
| Breast_Cancer | MLLT1 | TcoF: TF | MCF-7 | ENCSR427BBI_2 | 63557_sort_peaks.narrowPeak.bed.bed | ENCODE |
| Breast_Cancer | MTA2 | TcoF: TF | MCF-7 | ENCSR551ZDZ_1 | 63873_sort_peaks.narrowPeak.bed.bed | ENCODE |
| Breast_Cancer | MTA2 | TcoF: TF | MCF-7 | ENCSR551ZDZ_2 | 63874_sort_peaks.narrowPeak.bed.bed | ENCODE |
| Breast_Cancer | MBD2 | TcoF: TF | MCF-7 | ENCSR940MHE_1 | 64900_sort_peaks.narrowPeak.bed.bed | ENCODE |
| Breast_Cancer | MBD2 | TcoF: TF | MCF-7 | ENCSR940MHE_2 | 64901_sort_peaks.narrowPeak.bed.bed | ENCODE |
| Breast_Cancer | MBD3 | TcoF: TF | MCF-7 | GSM1089817 | GSE44737.MBD3.MCF7_remap2018_hg19__v1_2.bed | Remap |
| Breast_Cancer | SFPQ | Yet undefined DNA-binding domains | MCF-7 | GSM1411215 | 49154_sort_peaks.narrowPeak.bed.bed | Cistrome |
| Breast_Cancer | NONO | Yet undefined DNA-binding domains | MCF-7 | ENCSR912NMR_1 | 64839_sort_peaks.narrowPeak.bed.bed | ENCODE |
| Breast_Cancer | NONO | Yet undefined DNA-binding domains | MCF-7 | ENCSR912NMR_2 | 64840_sort_peaks.narrowPeak.bed.bed | ENCODE |
| Breast_Cancer | RFXANK | Yet undefined DNA-binding domains | MCF-7 | GSM2714251 | EXP036874_liftoverhg19.bed | GTRD |
| Breast_Cancer | GATA3 | Zinc-coordinating DNA-binding domains | MDA-MB-231 | GSM1855964 | SRX1156472.bed | ChIP-Atlas |
| Breast_Cancer | GATA3 | Zinc-coordinating DNA-binding domains | MDA-MB-231 | GSM1855965 | SRX1156473.bed | ChIP-Atlas |
| Breast_Cancer | ESR1 | Zinc-coordinating DNA-binding domains | T-47D | GSM1908537 | SRX1336216.bed | ChIP-Atlas |
| Breast_Cancer | ZNF143 | Zinc-coordinating DNA-binding domains | MCF-7 | GSM2024873 | SRX1510969.bed | ChIP-Atlas |
| Breast_Cancer | ZNF143 | Zinc-coordinating DNA-binding domains | MCF-7 | GSM2024874 | SRX1510970.bed | ChIP-Atlas |
| Breast_Cancer | ZNF143 | Zinc-coordinating DNA-binding domains | MCF-7 | GSM2024875 | SRX1510971.bed | ChIP-Atlas |
| Breast_Cancer | ZNF143 | Zinc-coordinating DNA-binding domains | MCF-7 | GSM2024876 | SRX1510972.bed | ChIP-Atlas |
| Breast_Cancer | ESR1 | Zinc-coordinating DNA-binding domains | T-47D | GSM2125098 | SRX1709953.bed | ChIP-Atlas |
| Breast_Cancer | ESR1 | Zinc-coordinating DNA-binding domains | T-47D | GSM2125099 | SRX1709954.bed | ChIP-Atlas |
| Breast_Cancer | ESR1 | Zinc-coordinating DNA-binding domains | T-47D | GSM2125100 | SRX1709955.bed | ChIP-Atlas |
| Breast_Cancer | ESR1 | Zinc-coordinating DNA-binding domains | T-47D | GSM2125101 | SRX1709956.bed | ChIP-Atlas |
| Breast_Cancer | ESR1 | Zinc-coordinating DNA-binding domains | T-47D | GSM2125102 | SRX1709957.bed | ChIP-Atlas |
| Breast_Cancer | ESR1 | Zinc-coordinating DNA-binding domains | T-47D | GSM2125103 | SRX1709958.bed | ChIP-Atlas |
| Breast_Cancer | ESR1 | Zinc-coordinating DNA-binding domains | T-47D | GSM2125104 | SRX1709959.bed | ChIP-Atlas |
| Breast_Cancer | ESR1 | Zinc-coordinating DNA-binding domains | T-47D | GSM2125105 | SRX1709960.bed | ChIP-Atlas |
| Breast_Cancer | ESR1 | Zinc-coordinating DNA-binding domains | T-47D | GSM2125106 | SRX1709961.bed | ChIP-Atlas |
| Breast_Cancer | ESR1 | Zinc-coordinating DNA-binding domains | T-47D | GSM2125107 | SRX1709962.bed | ChIP-Atlas |
| Breast_Cancer | ESR1 | Zinc-coordinating DNA-binding domains | T-47D | GSM2125108 | SRX1709963.bed | ChIP-Atlas |
| Breast_Cancer | ESR1 | Zinc-coordinating DNA-binding domains | T-47D | GSM2125109 | SRX1709964.bed | ChIP-Atlas |
| Breast_Cancer | PGR | Zinc-coordinating DNA-binding domains | T-47D | GSM2125116 | SRX1709971.bed | ChIP-Atlas |
| Breast_Cancer | ESR1 | Zinc-coordinating DNA-binding domains | T-47D | GSM2125117 | SRX1709972.bed | ChIP-Atlas |
| Breast_Cancer | PGR | Zinc-coordinating DNA-binding domains | T-47D | GSM2125118 | SRX1709973.bed | ChIP-Atlas |
| Breast_Cancer | ESR1 | Zinc-coordinating DNA-binding domains | T-47D | GSM2125119 | SRX1709974.bed | ChIP-Atlas |
| Breast_Cancer | PGR | Zinc-coordinating DNA-binding domains | T-47D | GSM2125120 | SRX1709975.bed | ChIP-Atlas |
| Breast_Cancer | ESR1 | Zinc-coordinating DNA-binding domains | T-47D | GSM2125121 | SRX1709976.bed | ChIP-Atlas |
| Breast_Cancer | PGR | Zinc-coordinating DNA-binding domains | T-47D | GSM2125122 | SRX1709977.bed | ChIP-Atlas |
| Breast_Cancer | ESR1 | Zinc-coordinating DNA-binding domains | T-47D | GSM2125123 | SRX1709978.bed | ChIP-Atlas |
| Breast_Cancer | PGR | Zinc-coordinating DNA-binding domains | T-47D | GSM2125128 | SRX1709983.bed | ChIP-Atlas |
| Breast_Cancer | ESR1 | Zinc-coordinating DNA-binding domains | T-47D | GSM2125129 | SRX1709984.bed | ChIP-Atlas |
| Breast_Cancer | PGR | Zinc-coordinating DNA-binding domains | T-47D | GSM2125130 | SRX1709985.bed | ChIP-Atlas |
| Breast_Cancer | ESR1 | Zinc-coordinating DNA-binding domains | T-47D | GSM2125131 | SRX1709986.bed | ChIP-Atlas |
| Breast_Cancer | PGR | Zinc-coordinating DNA-binding domains | T-47D | GSM2125132 | SRX1709987.bed | ChIP-Atlas |
| Breast_Cancer | ESR1 | Zinc-coordinating DNA-binding domains | T-47D | GSM2125133 | SRX1709988.bed | ChIP-Atlas |
| Breast_Cancer | PGR | Zinc-coordinating DNA-binding domains | T-47D | GSM2125134 | SRX1709989.bed | ChIP-Atlas |
| Breast_Cancer | ESR1 | Zinc-coordinating DNA-binding domains | T-47D | GSM2125135 | SRX1709990.bed | ChIP-Atlas |
| Breast_Cancer | ESR1 | Zinc-coordinating DNA-binding domains | T-47D | GSM2242426 | SRX1961155.bed | ChIP-Atlas |
| Breast_Cancer | ESR1 | Zinc-coordinating DNA-binding domains | T-47D | GSM2242427 | SRX1961156.bed | ChIP-Atlas |
| Breast_Cancer | ESR1 | Zinc-coordinating DNA-binding domains | T-47D | GSM2242431 | SRX1961161.bed | ChIP-Atlas |
| Breast_Cancer | ESR1 | Zinc-coordinating DNA-binding domains | T-47D | GSM2242432 | SRX1961162.bed | ChIP-Atlas |
| Breast_Cancer | KMT2D | Zinc-coordinating DNA-binding domains | T-47D | GSM2508062 | SRX2588916.bed | ChIP-Atlas |
| Breast_Cancer | KMT2D | Zinc-coordinating DNA-binding domains | T-47D | GSM2508064 | SRX2588918.bed | ChIP-Atlas |
| Breast_Cancer | ZNF516 | Zinc-coordinating DNA-binding domains | MCF-7 | GSM2583862 | SRX2745728.bed | ChIP-Atlas |
| Breast_Cancer | DNMT3A | Zinc-coordinating DNA-binding domains | MCF-7 | GSM2735973 | SRX3075718.bed | ChIP-Atlas |
| Breast_Cancer | ZFX | Zinc-coordinating DNA-binding domains | MCF-7 | GSM2741762 | SRX3091902.bed | ChIP-Atlas |
| Breast_Cancer | ZFX | Zinc-coordinating DNA-binding domains | MCF-7 | GSM2741763 | SRX3091903.bed | ChIP-Atlas |
| Breast_Cancer | ZNF711 | Zinc-coordinating DNA-binding domains | MCF-7 | GSM2741764 | SRX3091904.bed | ChIP-Atlas |
| Breast_Cancer | ZNF711 | Zinc-coordinating DNA-binding domains | MCF-7 | GSM2741765 | SRX3091905.bed | ChIP-Atlas |
| Breast_Cancer | AR | Zinc-coordinating DNA-binding domains | MCF-7 | GSM2797088 | SRX3229134.bed | ChIP-Atlas |
| Breast_Cancer | NR3C1 | Zinc-coordinating DNA-binding domains | MCF-7 | GSM2797089 | SRX3229135.bed | ChIP-Atlas |
| Breast_Cancer | ESR1 | Zinc-coordinating DNA-binding domains | MCF-7 | GSM1019125 | 36825_sort_peaks.narrowPeak.bed.bed | Cistrome |
| Breast_Cancer | ESR1 | Zinc-coordinating DNA-binding domains | MCF-7 | GSM1019128 | 36834_sort_peaks.narrowPeak.bed.bed | Cistrome |
| Breast_Cancer | ESR1 | Zinc-coordinating DNA-binding domains | MCF-7 | GSM1019131 | 36819_sort_peaks.narrowPeak.bed.bed | Cistrome |
| Breast_Cancer | NR2F2 | Zinc-coordinating DNA-binding domains | MCF-7 | GSM1019133 | 36804_sort_peaks.narrowPeak.bed.bed | Cistrome |
| Breast_Cancer | KLF4 | Zinc-coordinating DNA-binding domains | MCF-7 | GSM1019134 | 36821_sort_peaks.narrowPeak.bed.bed | Cistrome |
| Breast_Cancer | RXRA | Zinc-coordinating DNA-binding domains | MCF-7 | GSM1019136 | 36820_sort_peaks.narrowPeak.bed.bed | Cistrome |
| Breast_Cancer | ESR1 | Zinc-coordinating DNA-binding domains | MCF-7 | GSM1115990 | 33101_sort_peaks.narrowPeak.bed.bed | Cistrome |
| Breast_Cancer | ESR1 | Zinc-coordinating DNA-binding domains | MCF-7 | GSM1115991 | 33100_sort_peaks.narrowPeak.bed.bed | Cistrome |
| Breast_Cancer | NR5A2 | Zinc-coordinating DNA-binding domains | MCF-7 | GSM1143122 | 41515_sort_peaks.narrowPeak.bed.bed | Cistrome |
| Breast_Cancer | ESR1 | Zinc-coordinating DNA-binding domains | MCF-7 | GSM1187116 | 35046_sort_peaks.narrowPeak.bed.bed | Cistrome |
| Breast_Cancer | ESR1 | Zinc-coordinating DNA-binding domains | MCF-7 | GSM1187117 | 35051_sort_peaks.narrowPeak.bed.bed | Cistrome |
| Breast_Cancer | ESR1 | Zinc-coordinating DNA-binding domains | MCF-7 | GSM1187118 | 35044_sort_peaks.narrowPeak.bed.bed | Cistrome |
| Breast_Cancer | ESR1 | Zinc-coordinating DNA-binding domains | MCF-7 | GSM1198711 | 33216_sort_peaks.narrowPeak.bed.bed | Cistrome |
| Breast_Cancer | ESR1 | Zinc-coordinating DNA-binding domains | MCF-7 | GSM1198712 | 33221_sort_peaks.narrowPeak.bed.bed | Cistrome |
| Breast_Cancer | ESR1 | Zinc-coordinating DNA-binding domains | MCF-7 | GSM1198713 | 33224_sort_peaks.narrowPeak.bed.bed | Cistrome |
| Breast_Cancer | ESR1 | Zinc-coordinating DNA-binding domains | MCF-7 | GSM1198714 | 33223_sort_peaks.narrowPeak.bed.bed | Cistrome |
| Breast_Cancer | NR5A2 | Zinc-coordinating DNA-binding domains | MCF-7 | GSM1198716 | 33220_sort_peaks.narrowPeak.bed.bed | Cistrome |
| Breast_Cancer | PGR | Zinc-coordinating DNA-binding domains | T-47D | GSM1245253 | 47573_sort_peaks.narrowPeak.bed.bed | Cistrome |
| Breast_Cancer | ESR1 | Zinc-coordinating DNA-binding domains | MCF-7 | GSM1295590 | 44437_sort_peaks.narrowPeak.bed.bed | Cistrome |
| Breast_Cancer | ESR1 | Zinc-coordinating DNA-binding domains | MCF-7 | GSM1295591 | 44438_sort_peaks.narrowPeak.bed.bed | Cistrome |
| Breast_Cancer | ESR1 | Zinc-coordinating DNA-binding domains | MCF-7 | GSM1325246 | 48167_sort_peaks.narrowPeak.bed.bed | Cistrome |
| Breast_Cancer | ESR1 | Zinc-coordinating DNA-binding domains | MCF-7 | GSM1325247 | 48168_sort_peaks.narrowPeak.bed.bed | Cistrome |
| Breast_Cancer | ESR1 | Zinc-coordinating DNA-binding domains | MCF-7 | GSM1325248 | 48169_sort_peaks.narrowPeak.bed.bed | Cistrome |
| Breast_Cancer | ESR1 | Zinc-coordinating DNA-binding domains | MCF-7 | GSM1325249 | 48170_sort_peaks.narrowPeak.bed.bed | Cistrome |
| Breast_Cancer | ESR1 | Zinc-coordinating DNA-binding domains | MCF-7 | GSM1325250 | 48171_sort_peaks.narrowPeak.bed.bed | Cistrome |
| Breast_Cancer | ESR1 | Zinc-coordinating DNA-binding domains | MCF-7 | GSM1325251 | 48172_sort_peaks.narrowPeak.bed.bed | Cistrome |
| Breast_Cancer | ESR1 | Zinc-coordinating DNA-binding domains | MCF-7 | GSM1348521 | 48453_sort_peaks.narrowPeak.bed.bed | Cistrome |
| Breast_Cancer | ESR1 | Zinc-coordinating DNA-binding domains | MCF-7 | GSM1348522 | 48454_sort_peaks.narrowPeak.bed.bed | Cistrome |
| Breast_Cancer | ESR1 | Zinc-coordinating DNA-binding domains | MCF-7 | GSM1348523 | 48455_sort_peaks.narrowPeak.bed.bed | Cistrome |
| Breast_Cancer | ESR1 | Zinc-coordinating DNA-binding domains | MCF-7 | GSM1348524 | 48456_sort_peaks.narrowPeak.bed.bed | Cistrome |
| Breast_Cancer | ESR1 | Zinc-coordinating DNA-binding domains | MCF-7 | GSM1348525 | 48457_sort_peaks.narrowPeak.bed.bed | Cistrome |
| Breast_Cancer | ESR1 | Zinc-coordinating DNA-binding domains | MCF-7 | GSM1348526 | 48458_sort_peaks.narrowPeak.bed.bed | Cistrome |
| Breast_Cancer | NR3C1 | Zinc-coordinating DNA-binding domains | MDA-MB-231 | GSM1350526 | 56100_sort_peaks.narrowPeak.bed.bed | Cistrome |
| Breast_Cancer | NR3C1 | Zinc-coordinating DNA-binding domains | MDA-MB-231 | GSM1350528 | 56102_sort_peaks.narrowPeak.bed.bed | Cistrome |
| Breast_Cancer | NR3C1 | Zinc-coordinating DNA-binding domains | MDA-MB-231 | GSM1350529 | 56103_sort_peaks.narrowPeak.bed.bed | Cistrome |
| Breast_Cancer | NR3C1 | Zinc-coordinating DNA-binding domains | MDA-MB-231 | GSM1350530 | 56104_sort_peaks.narrowPeak.bed.bed | Cistrome |
| Breast_Cancer | NR3C1 | Zinc-coordinating DNA-binding domains | MDA-MB-231 | GSM1350531 | 56105_sort_peaks.narrowPeak.bed.bed | Cistrome |
| Breast_Cancer | ESR1 | Zinc-coordinating DNA-binding domains | MCF-7 | GSM1469979 | 49611_sort_peaks.narrowPeak.bed.bed | Cistrome |
| Breast_Cancer | ESR1 | Zinc-coordinating DNA-binding domains | MCF-7 | GSM1469980 | 49612_sort_peaks.narrowPeak.bed.bed | Cistrome |
| Breast_Cancer | RARA | Zinc-coordinating DNA-binding domains | MCF-7 | GSM1469981 | 49613_sort_peaks.narrowPeak.bed.bed | Cistrome |
| Breast_Cancer | RARA | Zinc-coordinating DNA-binding domains | MCF-7 | GSM1469982 | 49614_sort_peaks.narrowPeak.bed.bed | Cistrome |
| Breast_Cancer | RARA | Zinc-coordinating DNA-binding domains | MCF-7 | GSM1469983 | 49615_sort_peaks.narrowPeak.bed.bed | Cistrome |
| Breast_Cancer | RARA | Zinc-coordinating DNA-binding domains | MCF-7 | GSM1469984 | 49616_sort_peaks.narrowPeak.bed.bed | Cistrome |
| Breast_Cancer | RARG | Zinc-coordinating DNA-binding domains | MCF-7 | GSM1469985 | 49617_sort_peaks.narrowPeak.bed.bed | Cistrome |
| Breast_Cancer | RARG | Zinc-coordinating DNA-binding domains | MCF-7 | GSM1469986 | 49618_sort_peaks.narrowPeak.bed.bed | Cistrome |
| Breast_Cancer | RARG | Zinc-coordinating DNA-binding domains | MCF-7 | GSM1469987 | 49619_sort_peaks.narrowPeak.bed.bed | Cistrome |
| Breast_Cancer | RARA | Zinc-coordinating DNA-binding domains | MCF-7 | GSM1469988 | 49620_sort_peaks.narrowPeak.bed.bed | Cistrome |
| Breast_Cancer | RARA | Zinc-coordinating DNA-binding domains | MCF-7 | GSM1469989 | 49621_sort_peaks.narrowPeak.bed.bed | Cistrome |
| Breast_Cancer | RARG | Zinc-coordinating DNA-binding domains | MCF-7 | GSM1469990 | 49622_sort_peaks.narrowPeak.bed.bed | Cistrome |
| Breast_Cancer | RARG | Zinc-coordinating DNA-binding domains | MCF-7 | GSM1469991 | 49623_sort_peaks.narrowPeak.bed.bed | Cistrome |
| Breast_Cancer | GATA3 | Zinc-coordinating DNA-binding domains | MCF-7 | GSM1469992 | 49624_sort_peaks.narrowPeak.bed.bed | Cistrome |
| Breast_Cancer | GATA3 | Zinc-coordinating DNA-binding domains | MCF-7 | GSM1469993 | 49625_sort_peaks.narrowPeak.bed.bed | Cistrome |
| Breast_Cancer | GATA3 | Zinc-coordinating DNA-binding domains | MCF-7 | GSM1469994 | 49626_sort_peaks.narrowPeak.bed.bed | Cistrome |
| Breast_Cancer | GATA3 | Zinc-coordinating DNA-binding domains | MCF-7 | GSM1469995 | 49627_sort_peaks.narrowPeak.bed.bed | Cistrome |
| Breast_Cancer | GATA3 | Zinc-coordinating DNA-binding domains | MCF-7 | GSM1469996 | 49628_sort_peaks.narrowPeak.bed.bed | Cistrome |
| Breast_Cancer | ESR1 | Zinc-coordinating DNA-binding domains | MCF-7 | GSM1470007 | 49639_sort_peaks.narrowPeak.bed.bed | Cistrome |
| Breast_Cancer | ESR1 | Zinc-coordinating DNA-binding domains | MCF-7 | GSM1470008 | 49640_sort_peaks.narrowPeak.bed.bed | Cistrome |
| Breast_Cancer | ESR1 | Zinc-coordinating DNA-binding domains | MCF-7 | GSM1470009 | 49641_sort_peaks.narrowPeak.bed.bed | Cistrome |
| Breast_Cancer | ESR1 | Zinc-coordinating DNA-binding domains | MCF-7 | GSM1470010 | 49642_sort_peaks.narrowPeak.bed.bed | Cistrome |
| Breast_Cancer | ESR1 | Zinc-coordinating DNA-binding domains | MCF-7 | GSM1470011 | 49643_sort_peaks.narrowPeak.bed.bed | Cistrome |
| Breast_Cancer | ESR1 | Zinc-coordinating DNA-binding domains | MCF-7 | GSM1470012 | 49644_sort_peaks.narrowPeak.bed.bed | Cistrome |
| Breast_Cancer | ESR1 | Zinc-coordinating DNA-binding domains | MCF-7 | GSM1470021 | 49653_sort_peaks.narrowPeak.bed.bed | Cistrome |
| Breast_Cancer | ESR1 | Zinc-coordinating DNA-binding domains | MCF-7 | GSM1470022 | 49654_sort_peaks.narrowPeak.bed.bed | Cistrome |
| Breast_Cancer | ESR1 | Zinc-coordinating DNA-binding domains | MCF-7 | GSM1470023 | 49655_sort_peaks.narrowPeak.bed.bed | Cistrome |
| Breast_Cancer | ESR1 | Zinc-coordinating DNA-binding domains | MCF-7 | GSM1470024 | 49656_sort_peaks.narrowPeak.bed.bed | Cistrome |
| Breast_Cancer | ESR1 | Zinc-coordinating DNA-binding domains | MCF-7 | GSM1523056 | 54620_sort_peaks.narrowPeak.bed.bed | Cistrome |
| Breast_Cancer | ESR1 | Zinc-coordinating DNA-binding domains | MCF-7 | GSM1523057 | 54621_sort_peaks.narrowPeak.bed.bed | Cistrome |
| Breast_Cancer | ESR1 | Zinc-coordinating DNA-binding domains | MCF-7 | GSM1523081 | 54645_sort_peaks.narrowPeak.bed.bed | Cistrome |
| Breast_Cancer | ESR1 | Zinc-coordinating DNA-binding domains | MCF-7 | GSM1523082 | 54646_sort_peaks.narrowPeak.bed.bed | Cistrome |
| Breast_Cancer | ESR1 | Zinc-coordinating DNA-binding domains | MCF-7 | GSM1523083 | 54647_sort_peaks.narrowPeak.bed.bed | Cistrome |
| Breast_Cancer | ESR1 | Zinc-coordinating DNA-binding domains | MCF-7 | GSM1523084 | 54648_sort_peaks.narrowPeak.bed.bed | Cistrome |
| Breast_Cancer | ESR1 | Zinc-coordinating DNA-binding domains | MCF-7 | GSM1534720 | 52606_sort_peaks.narrowPeak.bed.bed | Cistrome |
| Breast_Cancer | ESR1 | Zinc-coordinating DNA-binding domains | MCF-7 | GSM1534721 | 52607_sort_peaks.narrowPeak.bed.bed | Cistrome |
| Breast_Cancer | ESR1 | Zinc-coordinating DNA-binding domains | MCF-7 | GSM1534722 | 52608_sort_peaks.narrowPeak.bed.bed | Cistrome |
| Breast_Cancer | ESR1 | Zinc-coordinating DNA-binding domains | MCF-7 | GSM1534723 | 52609_sort_peaks.narrowPeak.bed.bed | Cistrome |
| Breast_Cancer | ESR1 | Zinc-coordinating DNA-binding domains | MCF-7 | GSM1534724 | 52610_sort_peaks.narrowPeak.bed.bed | Cistrome |
| Breast_Cancer | ESR1 | Zinc-coordinating DNA-binding domains | MCF-7 | GSM1534725 | 52611_sort_peaks.narrowPeak.bed.bed | Cistrome |
| Breast_Cancer | ESR1 | Zinc-coordinating DNA-binding domains | MCF-7 | GSM1534726 | 52612_sort_peaks.narrowPeak.bed.bed | Cistrome |
| Breast_Cancer | ESR1 | Zinc-coordinating DNA-binding domains | MCF-7 | GSM1534727 | 52613_sort_peaks.narrowPeak.bed.bed | Cistrome |
| Breast_Cancer | ESR1 | Zinc-coordinating DNA-binding domains | MCF-7 | GSM1534746 | 52632_sort_peaks.narrowPeak.bed.bed | Cistrome |
| Breast_Cancer | ESR1 | Zinc-coordinating DNA-binding domains | MCF-7 | GSM1534747 | 52633_sort_peaks.narrowPeak.bed.bed | Cistrome |
| Breast_Cancer | ESR1 | Zinc-coordinating DNA-binding domains | MCF-7 | GSM1534750 | 52636_sort_peaks.narrowPeak.bed.bed | Cistrome |
| Breast_Cancer | ESR1 | Zinc-coordinating DNA-binding domains | MCF-7 | GSM1534751 | 52637_sort_peaks.narrowPeak.bed.bed | Cistrome |
| Breast_Cancer | GATA3 | Zinc-coordinating DNA-binding domains | MCF-7 | GSM1642515 | 52964_sort_peaks.narrowPeak.bed.bed | Cistrome |
| Breast_Cancer | PGR | Zinc-coordinating DNA-binding domains | T-47D | GSM1669032 | 53976_sort_peaks.narrowPeak.bed.bed | Cistrome |
| Breast_Cancer | PGR | Zinc-coordinating DNA-binding domains | T-47D | GSM1669033 | 53977_sort_peaks.narrowPeak.bed.bed | Cistrome |
| Breast_Cancer | PGR | Zinc-coordinating DNA-binding domains | T-47D | GSM1669034 | 53978_sort_peaks.narrowPeak.bed.bed | Cistrome |
| Breast_Cancer | PGR | Zinc-coordinating DNA-binding domains | T-47D | GSM1669035 | 53979_sort_peaks.narrowPeak.bed.bed | Cistrome |
| Breast_Cancer | PGR | Zinc-coordinating DNA-binding domains | T-47D | GSM1669036 | 53980_sort_peaks.narrowPeak.bed.bed | Cistrome |
| Breast_Cancer | PGR | Zinc-coordinating DNA-binding domains | T-47D | GSM1669037 | 53981_sort_peaks.narrowPeak.bed.bed | Cistrome |
| Breast_Cancer | PGR | Zinc-coordinating DNA-binding domains | T-47D | GSM1669038 | 53982_sort_peaks.narrowPeak.bed.bed | Cistrome |
| Breast_Cancer | PGR | Zinc-coordinating DNA-binding domains | T-47D | GSM1669039 | 53983_sort_peaks.narrowPeak.bed.bed | Cistrome |
| Breast_Cancer | PGR | Zinc-coordinating DNA-binding domains | T-47D | GSM1669040 | 53984_sort_peaks.narrowPeak.bed.bed | Cistrome |
| Breast_Cancer | ESR1 | Zinc-coordinating DNA-binding domains | MCF-7 | GSM1669042 | 53986_sort_peaks.narrowPeak.bed.bed | Cistrome |
| Breast_Cancer | ESR1 | Zinc-coordinating DNA-binding domains | MCF-7 | GSM1669043 | 53987_sort_peaks.narrowPeak.bed.bed | Cistrome |
| Breast_Cancer | ESR1 | Zinc-coordinating DNA-binding domains | MCF-7 | GSM1669044 | 53988_sort_peaks.narrowPeak.bed.bed | Cistrome |
| Breast_Cancer | ESR1 | Zinc-coordinating DNA-binding domains | MCF-7 | GSM1669045 | 53989_sort_peaks.narrowPeak.bed.bed | Cistrome |
| Breast_Cancer | ESR1 | Zinc-coordinating DNA-binding domains | MCF-7 | GSM1669046 | 53990_sort_peaks.narrowPeak.bed.bed | Cistrome |
| Breast_Cancer | ESR1 | Zinc-coordinating DNA-binding domains | MCF-7 | GSM1669047 | 53991_sort_peaks.narrowPeak.bed.bed | Cistrome |
| Breast_Cancer | ESR1 | Zinc-coordinating DNA-binding domains | MCF-7 | GSM1669048 | 53992_sort_peaks.narrowPeak.bed.bed | Cistrome |
| Breast_Cancer | ESR1 | Zinc-coordinating DNA-binding domains | MCF-7 | GSM1669049 | 53993_sort_peaks.narrowPeak.bed.bed | Cistrome |
| Breast_Cancer | ESR1 | Zinc-coordinating DNA-binding domains | MCF-7 | GSM1669050 | 53994_sort_peaks.narrowPeak.bed.bed | Cistrome |
| Breast_Cancer | PGR | Zinc-coordinating DNA-binding domains | MCF-7 | GSM1669067 | 54011_sort_peaks.narrowPeak.bed.bed | Cistrome |
| Breast_Cancer | PGR | Zinc-coordinating DNA-binding domains | MCF-7 | GSM1669068 | 54012_sort_peaks.narrowPeak.bed.bed | Cistrome |
| Breast_Cancer | PGR | Zinc-coordinating DNA-binding domains | MCF-7 | GSM1669069 | 54013_sort_peaks.narrowPeak.bed.bed | Cistrome |
| Breast_Cancer | PGR | Zinc-coordinating DNA-binding domains | MCF-7 | GSM1669070 | 54014_sort_peaks.narrowPeak.bed.bed | Cistrome |
| Breast_Cancer | PGR | Zinc-coordinating DNA-binding domains | MCF-7 | GSM1669071 | 54015_sort_peaks.narrowPeak.bed.bed | Cistrome |
| Breast_Cancer | PGR | Zinc-coordinating DNA-binding domains | MCF-7 | GSM1669072 | 54016_sort_peaks.narrowPeak.bed.bed | Cistrome |
| Breast_Cancer | PGR | Zinc-coordinating DNA-binding domains | MCF-7 | GSM1669073 | 54017_sort_peaks.narrowPeak.bed.bed | Cistrome |
| Breast_Cancer | PGR | Zinc-coordinating DNA-binding domains | MCF-7 | GSM1669074 | 54018_sort_peaks.narrowPeak.bed.bed | Cistrome |
| Breast_Cancer | PGR | Zinc-coordinating DNA-binding domains | MCF-7 | GSM1669075 | 54019_sort_peaks.narrowPeak.bed.bed | Cistrome |
| Breast_Cancer | PGR | Zinc-coordinating DNA-binding domains | MCF-7 | GSM1669076 | 54020_sort_peaks.narrowPeak.bed.bed | Cistrome |
| Breast_Cancer | PGR | Zinc-coordinating DNA-binding domains | MCF-7 | GSM1669077 | 54021_sort_peaks.narrowPeak.bed.bed | Cistrome |
| Breast_Cancer | ESR1 | Zinc-coordinating DNA-binding domains | MCF-7 | GSM1669078 | 54022_sort_peaks.narrowPeak.bed.bed | Cistrome |
| Breast_Cancer | ESR1 | Zinc-coordinating DNA-binding domains | MCF-7 | GSM1669079 | 54023_sort_peaks.narrowPeak.bed.bed | Cistrome |
| Breast_Cancer | ESR1 | Zinc-coordinating DNA-binding domains | MCF-7 | GSM1669080 | 54024_sort_peaks.narrowPeak.bed.bed | Cistrome |
| Breast_Cancer | ESR1 | Zinc-coordinating DNA-binding domains | MCF-7 | GSM1669081 | 54025_sort_peaks.narrowPeak.bed.bed | Cistrome |
| Breast_Cancer | ESR1 | Zinc-coordinating DNA-binding domains | MCF-7 | GSM1669082 | 54026_sort_peaks.narrowPeak.bed.bed | Cistrome |
| Breast_Cancer | ESR1 | Zinc-coordinating DNA-binding domains | MCF-7 | GSM1669083 | 54027_sort_peaks.narrowPeak.bed.bed | Cistrome |
| Breast_Cancer | ESR1 | Zinc-coordinating DNA-binding domains | MCF-7 | GSM1669084 | 54028_sort_peaks.narrowPeak.bed.bed | Cistrome |
| Breast_Cancer | ESR1 | Zinc-coordinating DNA-binding domains | MCF-7 | GSM1669085 | 54029_sort_peaks.narrowPeak.bed.bed | Cistrome |
| Breast_Cancer | ESR1 | Zinc-coordinating DNA-binding domains | MCF-7 | GSM1669086 | 54030_sort_peaks.narrowPeak.bed.bed | Cistrome |
| Breast_Cancer | ESR1 | Zinc-coordinating DNA-binding domains | MCF-7 | GSM1669087 | 54031_sort_peaks.narrowPeak.bed.bed | Cistrome |
| Breast_Cancer | ESR1 | Zinc-coordinating DNA-binding domains | MCF-7 | GSM1669088 | 54032_sort_peaks.narrowPeak.bed.bed | Cistrome |
| Breast_Cancer | ESR1 | Zinc-coordinating DNA-binding domains | MCF-7 | GSM1669089 | 54033_sort_peaks.narrowPeak.bed.bed | Cistrome |
| Breast_Cancer | PGR | Zinc-coordinating DNA-binding domains | MCF-7 | GSM1669090 | 54034_sort_peaks.narrowPeak.bed.bed | Cistrome |
| Breast_Cancer | PGR | Zinc-coordinating DNA-binding domains | MCF-7 | GSM1669091 | 54035_sort_peaks.narrowPeak.bed.bed | Cistrome |
| Breast_Cancer | PGR | Zinc-coordinating DNA-binding domains | MCF-7 | GSM1669092 | 54036_sort_peaks.narrowPeak.bed.bed | Cistrome |
| Breast_Cancer | PGR | Zinc-coordinating DNA-binding domains | MCF-7 | GSM1669093 | 54037_sort_peaks.narrowPeak.bed.bed | Cistrome |
| Breast_Cancer | PGR | Zinc-coordinating DNA-binding domains | MCF-7 | GSM1669094 | 54038_sort_peaks.narrowPeak.bed.bed | Cistrome |
| Breast_Cancer | PGR | Zinc-coordinating DNA-binding domains | MCF-7 | GSM1669095 | 54039_sort_peaks.narrowPeak.bed.bed | Cistrome |
| Breast_Cancer | PGR | Zinc-coordinating DNA-binding domains | MCF-7 | GSM1669096 | 54040_sort_peaks.narrowPeak.bed.bed | Cistrome |
| Breast_Cancer | PGR | Zinc-coordinating DNA-binding domains | MCF-7 | GSM1669097 | 54041_sort_peaks.narrowPeak.bed.bed | Cistrome |
| Breast_Cancer | PGR | Zinc-coordinating DNA-binding domains | MCF-7 | GSM1669098 | 54042_sort_peaks.narrowPeak.bed.bed | Cistrome |
| Breast_Cancer | PGR | Zinc-coordinating DNA-binding domains | MCF-7 | GSM1669099 | 54043_sort_peaks.narrowPeak.bed.bed | Cistrome |
| Breast_Cancer | PGR | Zinc-coordinating DNA-binding domains | MCF-7 | GSM1669100 | 54044_sort_peaks.narrowPeak.bed.bed | Cistrome |
| Breast_Cancer | PGR | Zinc-coordinating DNA-binding domains | MCF-7 | GSM1669101 | 54045_sort_peaks.narrowPeak.bed.bed | Cistrome |
| Breast_Cancer | PGR | Zinc-coordinating DNA-binding domains | T-47D | GSM1669114 | 54058_sort_peaks.narrowPeak.bed.bed | Cistrome |
| Breast_Cancer | PGR | Zinc-coordinating DNA-binding domains | T-47D | GSM1669115 | 54059_sort_peaks.narrowPeak.bed.bed | Cistrome |
| Breast_Cancer | PGR | Zinc-coordinating DNA-binding domains | T-47D | GSM1669116 | 54060_sort_peaks.narrowPeak.bed.bed | Cistrome |
| Breast_Cancer | PGR | Zinc-coordinating DNA-binding domains | T-47D | GSM1669117 | 54061_sort_peaks.narrowPeak.bed.bed | Cistrome |
| Breast_Cancer | PGR | Zinc-coordinating DNA-binding domains | T-47D | GSM1669118 | 54062_sort_peaks.narrowPeak.bed.bed | Cistrome |
| Breast_Cancer | PGR | Zinc-coordinating DNA-binding domains | T-47D | GSM1669119 | 54063_sort_peaks.narrowPeak.bed.bed | Cistrome |
| Breast_Cancer | PGR | Zinc-coordinating DNA-binding domains | T-47D | GSM1669120 | 54064_sort_peaks.narrowPeak.bed.bed | Cistrome |
| Breast_Cancer | PGR | Zinc-coordinating DNA-binding domains | T-47D | GSM1669121 | 54065_sort_peaks.narrowPeak.bed.bed | Cistrome |
| Breast_Cancer | PGR | Zinc-coordinating DNA-binding domains | T-47D | GSM1669122 | 54066_sort_peaks.narrowPeak.bed.bed | Cistrome |
| Breast_Cancer | PGR | Zinc-coordinating DNA-binding domains | T-47D | GSM1669123 | 54067_sort_peaks.narrowPeak.bed.bed | Cistrome |
| Breast_Cancer | PGR | Zinc-coordinating DNA-binding domains | T-47D | GSM1669124 | 54068_sort_peaks.narrowPeak.bed.bed | Cistrome |
| Breast_Cancer | PGR | Zinc-coordinating DNA-binding domains | T-47D | GSM1669125 | 54069_sort_peaks.narrowPeak.bed.bed | Cistrome |
| Breast_Cancer | ESR1 | Zinc-coordinating DNA-binding domains | MCF-7 | GSM1669132 | 54076_sort_peaks.narrowPeak.bed.bed | Cistrome |
| Breast_Cancer | ESR1 | Zinc-coordinating DNA-binding domains | MCF-7 | GSM1669133 | 54077_sort_peaks.narrowPeak.bed.bed | Cistrome |
| Breast_Cancer | ESR1 | Zinc-coordinating DNA-binding domains | MCF-7 | GSM1669134 | 54078_sort_peaks.narrowPeak.bed.bed | Cistrome |
| Breast_Cancer | ESR1 | Zinc-coordinating DNA-binding domains | MCF-7 | GSM1669135 | 54079_sort_peaks.narrowPeak.bed.bed | Cistrome |
| Breast_Cancer | ESR1 | Zinc-coordinating DNA-binding domains | MCF-7 | GSM1669136 | 54080_sort_peaks.narrowPeak.bed.bed | Cistrome |
| Breast_Cancer | ESR1 | Zinc-coordinating DNA-binding domains | MCF-7 | GSM1669137 | 54081_sort_peaks.narrowPeak.bed.bed | Cistrome |
| Breast_Cancer | ESR1 | Zinc-coordinating DNA-binding domains | MCF-7 | GSM1669138 | 54082_sort_peaks.narrowPeak.bed.bed | Cistrome |
| Breast_Cancer | ESR1 | Zinc-coordinating DNA-binding domains | MCF-7 | GSM1669139 | 54083_sort_peaks.narrowPeak.bed.bed | Cistrome |
| Breast_Cancer | ESR1 | Zinc-coordinating DNA-binding domains | MCF-7 | GSM1669140 | 54084_sort_peaks.narrowPeak.bed.bed | Cistrome |
| Breast_Cancer | ESR1 | Zinc-coordinating DNA-binding domains | MCF-7 | GSM1669141 | 54085_sort_peaks.narrowPeak.bed.bed | Cistrome |
| Breast_Cancer | ESR1 | Zinc-coordinating DNA-binding domains | MCF-7 | GSM1669142 | 54086_sort_peaks.narrowPeak.bed.bed | Cistrome |
| Breast_Cancer | ESR1 | Zinc-coordinating DNA-binding domains | MCF-7 | GSM1669143 | 54087_sort_peaks.narrowPeak.bed.bed | Cistrome |
| Breast_Cancer | ESR1 | Zinc-coordinating DNA-binding domains | MCF-7 | GSM1669144 | 54088_sort_peaks.narrowPeak.bed.bed | Cistrome |
| Breast_Cancer | ESR1 | Zinc-coordinating DNA-binding domains | MCF-7 | GSM1669145 | 54089_sort_peaks.narrowPeak.bed.bed | Cistrome |
| Breast_Cancer | PGR | Zinc-coordinating DNA-binding domains | MCF-7 | GSM1669146 | 54090_sort_peaks.narrowPeak.bed.bed | Cistrome |
| Breast_Cancer | PGR | Zinc-coordinating DNA-binding domains | MCF-7 | GSM1669147 | 54091_sort_peaks.narrowPeak.bed.bed | Cistrome |
| Breast_Cancer | PGR | Zinc-coordinating DNA-binding domains | MCF-7 | GSM1669148 | 54092_sort_peaks.narrowPeak.bed.bed | Cistrome |
| Breast_Cancer | PGR | Zinc-coordinating DNA-binding domains | MCF-7 | GSM1669149 | 54093_sort_peaks.narrowPeak.bed.bed | Cistrome |
| Breast_Cancer | PGR | Zinc-coordinating DNA-binding domains | MCF-7 | GSM1669150 | 54094_sort_peaks.narrowPeak.bed.bed | Cistrome |
| Breast_Cancer | PGR | Zinc-coordinating DNA-binding domains | MCF-7 | GSM1669151 | 54095_sort_peaks.narrowPeak.bed.bed | Cistrome |
| Breast_Cancer | PGR | Zinc-coordinating DNA-binding domains | MCF-7 | GSM1669152 | 54096_sort_peaks.narrowPeak.bed.bed | Cistrome |
| Breast_Cancer | PGR | Zinc-coordinating DNA-binding domains | MCF-7 | GSM1669153 | 54097_sort_peaks.narrowPeak.bed.bed | Cistrome |
| Breast_Cancer | PGR | Zinc-coordinating DNA-binding domains | MCF-7 | GSM1669154 | 54098_sort_peaks.narrowPeak.bed.bed | Cistrome |
| Breast_Cancer | PGR | Zinc-coordinating DNA-binding domains | MCF-7 | GSM1669155 | 54099_sort_peaks.narrowPeak.bed.bed | Cistrome |
| Breast_Cancer | PGR | Zinc-coordinating DNA-binding domains | MCF-7 | GSM1669156 | 54100_sort_peaks.narrowPeak.bed.bed | Cistrome |
| Breast_Cancer | PGR | Zinc-coordinating DNA-binding domains | MCF-7 | GSM1669157 | 54101_sort_peaks.narrowPeak.bed.bed | Cistrome |
| Breast_Cancer | PGR | Zinc-coordinating DNA-binding domains | MCF-7 | GSM1669158 | 54102_sort_peaks.narrowPeak.bed.bed | Cistrome |
| Breast_Cancer | PGR | Zinc-coordinating DNA-binding domains | MCF-7 | GSM1669159 | 54103_sort_peaks.narrowPeak.bed.bed | Cistrome |
| Breast_Cancer | CTCF | Zinc-coordinating DNA-binding domains | MCF-7 | GSM1817665 | 55781_sort_peaks.narrowPeak.bed.bed | Cistrome |
| Breast_Cancer | CTCF | Zinc-coordinating DNA-binding domains | MCF-7 | GSM1817666 | 55782_sort_peaks.narrowPeak.bed.bed | Cistrome |
| Breast_Cancer | CTCF | Zinc-coordinating DNA-binding domains | MCF-7 | GSM1817667 | 55783_sort_peaks.narrowPeak.bed.bed | Cistrome |
| Breast_Cancer | CTCFL | Zinc-coordinating DNA-binding domains | MCF-7 | GSM1817668 | 55784_sort_peaks.narrowPeak.bed.bed | Cistrome |
| Breast_Cancer | CTCFL | Zinc-coordinating DNA-binding domains | MCF-7 | GSM1817669 | 55785_sort_peaks.narrowPeak.bed.bed | Cistrome |
| Breast_Cancer | ESR1 | Zinc-coordinating DNA-binding domains | MCF-7 | GSM1831736 | 56902_sort_peaks.narrowPeak.bed.bed | Cistrome |
| Breast_Cancer | ESR1 | Zinc-coordinating DNA-binding domains | MCF-7 | GSM1831737 | 56903_sort_peaks.narrowPeak.bed.bed | Cistrome |
| Breast_Cancer | ESR1 | Zinc-coordinating DNA-binding domains | MCF-7 | GSM1831738 | 56904_sort_peaks.narrowPeak.bed.bed | Cistrome |
| Breast_Cancer | ESR1 | Zinc-coordinating DNA-binding domains | MCF-7 | GSM1831739 | 56905_sort_peaks.narrowPeak.bed.bed | Cistrome |
| Breast_Cancer | ESR1 | Zinc-coordinating DNA-binding domains | MCF-7 | GSM1890761 | 58921_sort_peaks.narrowPeak.bed.bed | Cistrome |
| Breast_Cancer | ESR1 | Zinc-coordinating DNA-binding domains | MCF-7 | GSM1967545 | 59373_sort_peaks.narrowPeak.bed.bed | Cistrome |
| Breast_Cancer | ESR1 | Zinc-coordinating DNA-binding domains | MCF-7 | GSM1967546 | 59374_sort_peaks.narrowPeak.bed.bed | Cistrome |
| Breast_Cancer | ESR1 | Zinc-coordinating DNA-binding domains | MCF-7 | GSM1967547 | 59375_sort_peaks.narrowPeak.bed.bed | Cistrome |
| Breast_Cancer | ESR1 | Zinc-coordinating DNA-binding domains | MCF-7 | GSM365925 | 2362_sort_peaks.narrowPeak.bed.bed | Cistrome |
| Breast_Cancer | ESR1 | Zinc-coordinating DNA-binding domains | MCF-7 | GSM365926 | 2305_sort_peaks.narrowPeak.bed.bed | Cistrome |
| Breast_Cancer | ESR1 | Zinc-coordinating DNA-binding domains | MCF-7 | GSM365927 | 2304_sort_peaks.narrowPeak.bed.bed | Cistrome |
| Breast_Cancer | ESR1 | Zinc-coordinating DNA-binding domains | MCF-7 | GSM365928 | 2303_sort_peaks.narrowPeak.bed.bed | Cistrome |
| Breast_Cancer | ESR1 | Zinc-coordinating DNA-binding domains | MCF-7 | GSM470418 | 2302_sort_peaks.narrowPeak.bed.bed | Cistrome |
| Breast_Cancer | ESR1 | Zinc-coordinating DNA-binding domains | MCF-7 | GSM470419 | 2301_sort_peaks.narrowPeak.bed.bed | Cistrome |
| Breast_Cancer | ESR1 | Zinc-coordinating DNA-binding domains | MCF-7 | GSM589236 | 2299_sort_peaks.narrowPeak.bed.bed | Cistrome |
| Breast_Cancer | ESR1 | Zinc-coordinating DNA-binding domains | MCF-7 | GSM589237 | 2298_sort_peaks.narrowPeak.bed.bed | Cistrome |
| Breast_Cancer | ESR1 | Zinc-coordinating DNA-binding domains | MCF-7 | GSM594602 | 2297_sort_peaks.narrowPeak.bed.bed | Cistrome |
| Breast_Cancer | ESR1 | Zinc-coordinating DNA-binding domains | MCF-7 | GSM594603 | 2296_sort_peaks.narrowPeak.bed.bed | Cistrome |
| Breast_Cancer | ESR1 | Zinc-coordinating DNA-binding domains | MCF-7 | GSM614610 | 2295_sort_peaks.narrowPeak.bed.bed | Cistrome |
| Breast_Cancer | ESR1 | Zinc-coordinating DNA-binding domains | MCF-7 | GSM614611 | 2294_sort_peaks.narrowPeak.bed.bed | Cistrome |
| Breast_Cancer | CTCF | Zinc-coordinating DNA-binding domains | MCF-7 | GSM614614 | 2255_sort_peaks.narrowPeak.bed.bed | Cistrome |
| Breast_Cancer | CTCF | Zinc-coordinating DNA-binding domains | MCF-7 | GSM614615 | 2254_sort_peaks.narrowPeak.bed.bed | Cistrome |
| Breast_Cancer | ESR1 | Zinc-coordinating DNA-binding domains | MCF-7 | GSM631452 | 6549_sort_peaks.narrowPeak.bed.bed | Cistrome |
| Breast_Cancer | ESR1 | Zinc-coordinating DNA-binding domains | MCF-7 | GSM631453 | 6550_sort_peaks.narrowPeak.bed.bed | Cistrome |
| Breast_Cancer | ESR1 | Zinc-coordinating DNA-binding domains | MCF-7 | GSM631454 | 6551_sort_peaks.narrowPeak.bed.bed | Cistrome |
| Breast_Cancer | ESR1 | Zinc-coordinating DNA-binding domains | MCF-7 | GSM631455 | 6552_sort_peaks.narrowPeak.bed.bed | Cistrome |
| Breast_Cancer | ESR1 | Zinc-coordinating DNA-binding domains | MCF-7 | GSM631456 | 6553_sort_peaks.narrowPeak.bed.bed | Cistrome |
| Breast_Cancer | ESR1 | Zinc-coordinating DNA-binding domains | MCF-7 | GSM631457 | 6554_sort_peaks.narrowPeak.bed.bed | Cistrome |
| Breast_Cancer | ESR1 | Zinc-coordinating DNA-binding domains | MCF-7 | GSM631458 | 6555_sort_peaks.narrowPeak.bed.bed | Cistrome |
| Breast_Cancer | ESR1 | Zinc-coordinating DNA-binding domains | MCF-7 | GSM631459 | 6556_sort_peaks.narrowPeak.bed.bed | Cistrome |
| Breast_Cancer | ESR1 | Zinc-coordinating DNA-binding domains | MCF-7 | GSM631460 | 6557_sort_peaks.narrowPeak.bed.bed | Cistrome |
| Breast_Cancer | ESR1 | Zinc-coordinating DNA-binding domains | MCF-7 | GSM631461 | 6558_sort_peaks.narrowPeak.bed.bed | Cistrome |
| Breast_Cancer | ESR1 | Zinc-coordinating DNA-binding domains | MCF-7 | GSM631462 | 6559_sort_peaks.narrowPeak.bed.bed | Cistrome |
| Breast_Cancer | ESR1 | Zinc-coordinating DNA-binding domains | MCF-7 | GSM631463 | 6560_sort_peaks.narrowPeak.bed.bed | Cistrome |
| Breast_Cancer | ESR1 | Zinc-coordinating DNA-binding domains | MCF-7 | GSM631464 | 6561_sort_peaks.narrowPeak.bed.bed | Cistrome |
| Breast_Cancer | ESR1 | Zinc-coordinating DNA-binding domains | MCF-7 | GSM631465 | 6562_sort_peaks.narrowPeak.bed.bed | Cistrome |
| Breast_Cancer | ESR1 | Zinc-coordinating DNA-binding domains | MCF-7 | GSM631466 | 6563_sort_peaks.narrowPeak.bed.bed | Cistrome |
| Breast_Cancer | ESR1 | Zinc-coordinating DNA-binding domains | MCF-7 | GSM631467 | 6564_sort_peaks.narrowPeak.bed.bed | Cistrome |
| Breast_Cancer | ESR1 | Zinc-coordinating DNA-binding domains | MCF-7 | GSM631468 | 6565_sort_peaks.narrowPeak.bed.bed | Cistrome |
| Breast_Cancer | CTCF | Zinc-coordinating DNA-binding domains | MCF-7 | GSM631475 | 6572_sort_peaks.narrowPeak.bed.bed | Cistrome |
| Breast_Cancer | CTCF | Zinc-coordinating DNA-binding domains | MCF-7 | GSM631476 | 6573_sort_peaks.narrowPeak.bed.bed | Cistrome |
| Breast_Cancer | CTCF | Zinc-coordinating DNA-binding domains | MCF-7 | GSM631477 | 6574_sort_peaks.narrowPeak.bed.bed | Cistrome |
| Breast_Cancer | CTCF | Zinc-coordinating DNA-binding domains | MCF-7 | GSM631478 | 6575_sort_peaks.narrowPeak.bed.bed | Cistrome |
| Breast_Cancer | CTCF | Zinc-coordinating DNA-binding domains | MCF-7 | GSM631479 | 6576_sort_peaks.narrowPeak.bed.bed | Cistrome |
| Breast_Cancer | ESR1 | Zinc-coordinating DNA-binding domains | MCF-7 | GSM631480 | 6577_sort_peaks.narrowPeak.bed.bed | Cistrome |
| Breast_Cancer | ESR1 | Zinc-coordinating DNA-binding domains | MCF-7 | GSM631481 | 6578_sort_peaks.narrowPeak.bed.bed | Cistrome |
| Breast_Cancer | ESR1 | Zinc-coordinating DNA-binding domains | MCF-7 | GSM631482 | 6579_sort_peaks.narrowPeak.bed.bed | Cistrome |
| Breast_Cancer | ESR1 | Zinc-coordinating DNA-binding domains | MCF-7 | GSM631483 | 6580_sort_peaks.narrowPeak.bed.bed | Cistrome |
| Breast_Cancer | ESR1 | Zinc-coordinating DNA-binding domains | MCF-7 | GSM631484 | 6581_sort_peaks.narrowPeak.bed.bed | Cistrome |
| Breast_Cancer | ESR1 | Zinc-coordinating DNA-binding domains | ZR-75-1 | GSM631485 | 40371_sort_peaks.narrowPeak.bed.bed | Cistrome |
| Breast_Cancer | ESR1 | Zinc-coordinating DNA-binding domains | ZR-75-1 | GSM631486 | 40375_sort_peaks.narrowPeak.bed.bed | Cistrome |
| Breast_Cancer | ESR1 | Zinc-coordinating DNA-binding domains | ZR-75-1 | GSM631487 | 40372_sort_peaks.narrowPeak.bed.bed | Cistrome |
| Breast_Cancer | ESR1 | Zinc-coordinating DNA-binding domains | ZR-75-1 | GSM631488 | 40373_sort_peaks.narrowPeak.bed.bed | Cistrome |
| Breast_Cancer | ESR1 | Zinc-coordinating DNA-binding domains | MCF-7 | GSM640422 | 2287_sort_peaks.narrowPeak.bed.bed | Cistrome |
| Breast_Cancer | GATA3 | Zinc-coordinating DNA-binding domains | MCF-7 | GSM720422 | 2325_sort_peaks.narrowPeak.bed.bed | Cistrome |
| Breast_Cancer | GATA3 | Zinc-coordinating DNA-binding domains | MCF-7 | GSM720423 | 2324_sort_peaks.narrowPeak.bed.bed | Cistrome |
| Breast_Cancer | PGR | Zinc-coordinating DNA-binding domains | T-47D | GSM770762 | 33567_sort_peaks.narrowPeak.bed.bed | Cistrome |
| Breast_Cancer | PGR | Zinc-coordinating DNA-binding domains | T-47D | GSM770764 | 33570_sort_peaks.narrowPeak.bed.bed | Cistrome |
| Breast_Cancer | PGR | Zinc-coordinating DNA-binding domains | T-47D | GSM770765 | 33566_sort_peaks.narrowPeak.bed.bed | Cistrome |
| Breast_Cancer | ESR1 | Zinc-coordinating DNA-binding domains | MCF-7 | GSM798423 | 33525_sort_peaks.narrowPeak.bed.bed | Cistrome |
| Breast_Cancer | ESR1 | Zinc-coordinating DNA-binding domains | MCF-7 | GSM798424 | 33504_sort_peaks.narrowPeak.bed.bed | Cistrome |
| Breast_Cancer | ESR1 | Zinc-coordinating DNA-binding domains | MCF-7 | GSM798425 | 33526_sort_peaks.narrowPeak.bed.bed | Cistrome |
| Breast_Cancer | ESR1 | Zinc-coordinating DNA-binding domains | ZR-75-1 | GSM798426 | 33487_sort_peaks.narrowPeak.bed.bed | Cistrome |
| Breast_Cancer | ESR1 | Zinc-coordinating DNA-binding domains | ZR-75-1 | GSM798427 | 33494_sort_peaks.narrowPeak.bed.bed | Cistrome |
| Breast_Cancer | ESR1 | Zinc-coordinating DNA-binding domains | MCF-7 | GSM798434 | 33520_sort_peaks.narrowPeak.bed.bed | Cistrome |
| Breast_Cancer | ESR1 | Zinc-coordinating DNA-binding domains | MCF-7 | GSM798435 | 33491_sort_peaks.narrowPeak.bed.bed | Cistrome |
| Breast_Cancer | ESR1 | Zinc-coordinating DNA-binding domains | MCF-7 | GSM854690 | 38474_sort_peaks.narrowPeak.bed.bed | Cistrome |
| Breast_Cancer | ESR1 | Zinc-coordinating DNA-binding domains | MCF-7 | GSM862178 | 5495_sort_peaks.narrowPeak.bed.bed | Cistrome |
| Breast_Cancer | ESR1 | Zinc-coordinating DNA-binding domains | MCF-7 | GSM862179 | 5496_sort_peaks.narrowPeak.bed.bed | Cistrome |
| Breast_Cancer | ESR1 | Zinc-coordinating DNA-binding domains | MCF-7 | GSM862180 | 5497_sort_peaks.narrowPeak.bed.bed | Cistrome |
| Breast_Cancer | ESR1 | Zinc-coordinating DNA-binding domains | MCF-7 | GSM862181 | 5498_sort_peaks.narrowPeak.bed.bed | Cistrome |
| Breast_Cancer | KMT2A | Zinc-coordinating DNA-binding domains | MCF-7 | GSM2264621 | 73847_sort_peaks.narrowPeak.bed.bed | Cistrome |
| Breast_Cancer | KMT2D | Zinc-coordinating DNA-binding domains | MCF-7 | GSM2264622 | 73846_sort_peaks.narrowPeak.bed.bed | Cistrome |
| Breast_Cancer | ESR1 | Zinc-coordinating DNA-binding domains | MCF-7 | GSM1643946 | 74402_sort_peaks.narrowPeak.bed.bed | Cistrome |
| Breast_Cancer | ESR1 | Zinc-coordinating DNA-binding domains | MCF-7 | GSM1643947 | 74401_sort_peaks.narrowPeak.bed.bed | Cistrome |
| Breast_Cancer | ESR1 | Zinc-coordinating DNA-binding domains | MCF-7 | GSM1643948 | 74400_sort_peaks.narrowPeak.bed.bed | Cistrome |
| Breast_Cancer | ESR1 | Zinc-coordinating DNA-binding domains | MCF-7 | GSM1643949 | 74399_sort_peaks.narrowPeak.bed.bed | Cistrome |
| Breast_Cancer | ESR1 | Zinc-coordinating DNA-binding domains | MCF-7 | GSM1643950 | 74398_sort_peaks.narrowPeak.bed.bed | Cistrome |
| Breast_Cancer | ESR1 | Zinc-coordinating DNA-binding domains | MCF-7 | GSM1643951 | 74397_sort_peaks.narrowPeak.bed.bed | Cistrome |
| Breast_Cancer | ESR1 | Zinc-coordinating DNA-binding domains | MCF-7 | GSM1643952 | 74396_sort_peaks.narrowPeak.bed.bed | Cistrome |
| Breast_Cancer | ESR1 | Zinc-coordinating DNA-binding domains | MCF-7 | GSM1643953 | 74395_sort_peaks.narrowPeak.bed.bed | Cistrome |
| Breast_Cancer | ESR1 | Zinc-coordinating DNA-binding domains | MCF-7 | GSM1643954 | 74394_sort_peaks.narrowPeak.bed.bed | Cistrome |
| Breast_Cancer | ESR1 | Zinc-coordinating DNA-binding domains | MCF-7 | GSM1643955 | 74393_sort_peaks.narrowPeak.bed.bed | Cistrome |
| Breast_Cancer | ESR1 | Zinc-coordinating DNA-binding domains | MCF-7 | GSM1643998 | 74384_sort_peaks.narrowPeak.bed.bed | Cistrome |
| Breast_Cancer | ESR1 | Zinc-coordinating DNA-binding domains | MCF-7 | GSM1643999 | 74383_sort_peaks.narrowPeak.bed.bed | Cistrome |
| Breast_Cancer | ESR1 | Zinc-coordinating DNA-binding domains | MCF-7 | GSM1644001 | 74381_sort_peaks.narrowPeak.bed.bed | Cistrome |
| Breast_Cancer | ESR1 | Zinc-coordinating DNA-binding domains | MCF-7 | GSM2040043 | 71863_sort_peaks.narrowPeak.bed.bed | Cistrome |
| Breast_Cancer | ESR1 | Zinc-coordinating DNA-binding domains | MCF-7 | GSM2040048 | 71859_sort_peaks.narrowPeak.bed.bed | Cistrome |
| Breast_Cancer | ESR1 | Zinc-coordinating DNA-binding domains | MCF-7 | GSM2061656 | 68057_sort_peaks.narrowPeak.bed.bed | Cistrome |
| Breast_Cancer | ESR1 | Zinc-coordinating DNA-binding domains | MCF-7 | GSM2061657 | 68056_sort_peaks.narrowPeak.bed.bed | Cistrome |
| Breast_Cancer | ESR1 | Zinc-coordinating DNA-binding domains | MCF-7 | GSM2071221 | 71858_sort_peaks.narrowPeak.bed.bed | Cistrome |
| Breast_Cancer | ESR1 | Zinc-coordinating DNA-binding domains | MCF-7 | GSM2071222 | 71857_sort_peaks.narrowPeak.bed.bed | Cistrome |
| Breast_Cancer | ESR1 | Zinc-coordinating DNA-binding domains | MCF-7 | GSM2071224 | 71856_sort_peaks.narrowPeak.bed.bed | Cistrome |
| Breast_Cancer | ESR1 | Zinc-coordinating DNA-binding domains | MCF-7 | GSM2071225 | 71855_sort_peaks.narrowPeak.bed.bed | Cistrome |
| Breast_Cancer | ESR1 | Zinc-coordinating DNA-binding domains | MCF-7 | GSM2135489 | 71066_sort_peaks.narrowPeak.bed.bed | Cistrome |
| Breast_Cancer | ESR1 | Zinc-coordinating DNA-binding domains | MCF-7 | GSM2135490 | 71065_sort_peaks.narrowPeak.bed.bed | Cistrome |
| Breast_Cancer | ESR1 | Zinc-coordinating DNA-binding domains | MCF-7 | GSM2135491 | 71064_sort_peaks.narrowPeak.bed.bed | Cistrome |
| Breast_Cancer | ESR1 | Zinc-coordinating DNA-binding domains | MCF-7 | GSM2135492 | 71063_sort_peaks.narrowPeak.bed.bed | Cistrome |
| Breast_Cancer | ESR1 | Zinc-coordinating DNA-binding domains | MCF-7 | GSM2154984 | 74160_sort_peaks.narrowPeak.bed.bed | Cistrome |
| Breast_Cancer | ESR1 | Zinc-coordinating DNA-binding domains | MCF-7 | GSM2154985 | 74159_sort_peaks.narrowPeak.bed.bed | Cistrome |
| Breast_Cancer | ESR1 | Zinc-coordinating DNA-binding domains | MCF-7 | GSM2154986 | 74158_sort_peaks.narrowPeak.bed.bed | Cistrome |
| Breast_Cancer | ESR1 | Zinc-coordinating DNA-binding domains | MCF-7 | GSM2154987 | 74157_sort_peaks.narrowPeak.bed.bed | Cistrome |
| Breast_Cancer | NR3C1 | Zinc-coordinating DNA-binding domains | MCF-7 | GSM2154988 | 74156_sort_peaks.narrowPeak.bed.bed | Cistrome |
| Breast_Cancer | NR3C1 | Zinc-coordinating DNA-binding domains | MCF-7 | GSM2154990 | 74154_sort_peaks.narrowPeak.bed.bed | Cistrome |
| Breast_Cancer | NR3C1 | Zinc-coordinating DNA-binding domains | MCF-7 | GSM2154991 | 74153_sort_peaks.narrowPeak.bed.bed | Cistrome |
| Breast_Cancer | NR3C1 | Zinc-coordinating DNA-binding domains | MCF-7 | GSM2154992 | 74152_sort_peaks.narrowPeak.bed.bed | Cistrome |
| Breast_Cancer | NR3C1 | Zinc-coordinating DNA-binding domains | MCF-7 | GSM2154993 | 74151_sort_peaks.narrowPeak.bed.bed | Cistrome |
| Breast_Cancer | GATA3 | Zinc-coordinating DNA-binding domains | MCF-7 | GSM2154996 | 74148_sort_peaks.narrowPeak.bed.bed | Cistrome |
| Breast_Cancer | GATA3 | Zinc-coordinating DNA-binding domains | MCF-7 | GSM2154997 | 74147_sort_peaks.narrowPeak.bed.bed | Cistrome |
| Breast_Cancer | GATA3 | Zinc-coordinating DNA-binding domains | MCF-7 | GSM2154998 | 74146_sort_peaks.narrowPeak.bed.bed | Cistrome |
| Breast_Cancer | CTCF | Zinc-coordinating DNA-binding domains | MCF-7 | GSM2257816 | 73002_sort_peaks.narrowPeak.bed.bed | Cistrome |
| Breast_Cancer | CTCF | Zinc-coordinating DNA-binding domains | MCF-7 | GSM2257817 | 73001_sort_peaks.narrowPeak.bed.bed | Cistrome |
| Breast_Cancer | CTCF | Zinc-coordinating DNA-binding domains | MCF-7 | GSM2257818 | 73000_sort_peaks.narrowPeak.bed.bed | Cistrome |
| Breast_Cancer | ESR1 | Zinc-coordinating DNA-binding domains | MCF-7 | GSM2257819 | 72999_sort_peaks.narrowPeak.bed.bed | Cistrome |
| Breast_Cancer | ESR1 | Zinc-coordinating DNA-binding domains | MCF-7 | GSM2257820 | 72998_sort_peaks.narrowPeak.bed.bed | Cistrome |
| Breast_Cancer | ESR1 | Zinc-coordinating DNA-binding domains | MCF-7 | GSM2257821 | 72997_sort_peaks.narrowPeak.bed.bed | Cistrome |
| Breast_Cancer | ESR1 | Zinc-coordinating DNA-binding domains | MCF-7 | GSM2257828 | 72993_sort_peaks.narrowPeak.bed.bed | Cistrome |
| Breast_Cancer | ESR1 | Zinc-coordinating DNA-binding domains | MCF-7 | GSM2257829 | 72992_sort_peaks.narrowPeak.bed.bed | Cistrome |
| Breast_Cancer | ESR1 | Zinc-coordinating DNA-binding domains | MCF-7 | GSM2305313 | 74121_sort_peaks.narrowPeak.bed.bed | Cistrome |
| Breast_Cancer | ESR1 | Zinc-coordinating DNA-binding domains | MCF-7 | GSM2305314 | 74120_sort_peaks.narrowPeak.bed.bed | Cistrome |
| Breast_Cancer | ESR1 | Zinc-coordinating DNA-binding domains | MCF-7 | GSM2467220 | 76109_sort_peaks.narrowPeak.bed.bed | Cistrome |
| Breast_Cancer | ESR1 | Zinc-coordinating DNA-binding domains | MCF-7 | GSM2467221 | 76108_sort_peaks.narrowPeak.bed.bed | Cistrome |
| Breast_Cancer | ESR1 | Zinc-coordinating DNA-binding domains | MCF-7 | GSM2467222 | 76107_sort_peaks.narrowPeak.bed.bed | Cistrome |
| Breast_Cancer | ESR1 | Zinc-coordinating DNA-binding domains | MCF-7 | GSM2467223 | 76106_sort_peaks.narrowPeak.bed.bed | Cistrome |
| Breast_Cancer | ESR1 | Zinc-coordinating DNA-binding domains | MCF-7 | GSM2467224 | 76105_sort_peaks.narrowPeak.bed.bed | Cistrome |
| Breast_Cancer | ESR1 | Zinc-coordinating DNA-binding domains | MCF-7 | GSM2467225 | 76104_sort_peaks.narrowPeak.bed.bed | Cistrome |
| Breast_Cancer | ESR1 | Zinc-coordinating DNA-binding domains | MCF-7 | GSM2467226 | 76103_sort_peaks.narrowPeak.bed.bed | Cistrome |
| Breast_Cancer | ESR1 | Zinc-coordinating DNA-binding domains | MCF-7 | GSM2467227 | 76102_sort_peaks.narrowPeak.bed.bed | Cistrome |
| Breast_Cancer | ESR1 | Zinc-coordinating DNA-binding domains | MCF-7 | GSM2467228 | 76101_sort_peaks.narrowPeak.bed.bed | Cistrome |
| Breast_Cancer | ESR1 | Zinc-coordinating DNA-binding domains | MCF-7 | GSM2467229 | 76100_sort_peaks.narrowPeak.bed.bed | Cistrome |
| Breast_Cancer | KLF10 | Zinc-coordinating DNA-binding domains | MCF-7 | GSM2574805 | 74639_sort_peaks.narrowPeak.bed.bed | Cistrome |
| Breast_Cancer | ESR1 | Zinc-coordinating DNA-binding domains | MCF-7 | GSM2648914 | 88336_sort_peaks.narrowPeak.bed.bed | Cistrome |
| Breast_Cancer | ESR1 | Zinc-coordinating DNA-binding domains | MCF-7 | GSM2648915 | 88335_sort_peaks.narrowPeak.bed.bed | Cistrome |
| Breast_Cancer | ESR1 | Zinc-coordinating DNA-binding domains | MCF-7 | GSM2648916 | 86283_sort_peaks.narrowPeak.bed.bed | Cistrome |
| Breast_Cancer | ESR1 | Zinc-coordinating DNA-binding domains | MCF-7 | GSM2648917 | 86282_sort_peaks.narrowPeak.bed.bed | Cistrome |
| Breast_Cancer | ESR1 | Zinc-coordinating DNA-binding domains | MCF-7 | GSM2648918 | 88349_sort_peaks.narrowPeak.bed.bed | Cistrome |
| Breast_Cancer | ESR1 | Zinc-coordinating DNA-binding domains | MCF-7 | GSM2648919 | 88345_sort_peaks.narrowPeak.bed.bed | Cistrome |
| Breast_Cancer | ESR1 | Zinc-coordinating DNA-binding domains | MCF-7 | GSM2648921 | 82424_sort_peaks.narrowPeak.bed.bed | Cistrome |
| Breast_Cancer | ESR1 | Zinc-coordinating DNA-binding domains | MCF-7 | GSM2648922 | 82425_sort_peaks.narrowPeak.bed.bed | Cistrome |
| Breast_Cancer | ESR1 | Zinc-coordinating DNA-binding domains | MCF-7 | GSM2648923 | 82426_sort_peaks.narrowPeak.bed.bed | Cistrome |
| Breast_Cancer | ESR1 | Zinc-coordinating DNA-binding domains | MCF-7 | GSM2648924 | 82427_sort_peaks.narrowPeak.bed.bed | Cistrome |
| Breast_Cancer | ESR1 | Zinc-coordinating DNA-binding domains | MCF-7 | GSM2670862 | 86212_sort_peaks.narrowPeak.bed.bed | Cistrome |
| Breast_Cancer | ESR1 | Zinc-coordinating DNA-binding domains | MCF-7 | GSM2670863 | 86213_sort_peaks.narrowPeak.bed.bed | Cistrome |
| Breast_Cancer | ESR1 | Zinc-coordinating DNA-binding domains | MCF-7 | GSM2670864 | 86211_sort_peaks.narrowPeak.bed.bed | Cistrome |
| Breast_Cancer | ESR1 | Zinc-coordinating DNA-binding domains | MCF-7 | GSM2747691 | 82325_sort_peaks.narrowPeak.bed.bed | Cistrome |
| Breast_Cancer | ESR1 | Zinc-coordinating DNA-binding domains | MCF-7 | GSM2747692 | 82326_sort_peaks.narrowPeak.bed.bed | Cistrome |
| Breast_Cancer | ESR1 | Zinc-coordinating DNA-binding domains | MCF-7 | GSM2747693 | 82327_sort_peaks.narrowPeak.bed.bed | Cistrome |
| Breast_Cancer | ESR1 | Zinc-coordinating DNA-binding domains | MCF-7 | GSM2747694 | 82328_sort_peaks.narrowPeak.bed.bed | Cistrome |
| Breast_Cancer | ESR1 | Zinc-coordinating DNA-binding domains | MCF-7 | GSM2747695 | 81409_sort_peaks.narrowPeak.bed.bed | Cistrome |
| Breast_Cancer | ESR1 | Zinc-coordinating DNA-binding domains | MCF-7 | GSM2747696 | 82330_sort_peaks.narrowPeak.bed.bed | Cistrome |
| Breast_Cancer | ESR1 | Zinc-coordinating DNA-binding domains | MCF-7 | GSM2747697 | 82331_sort_peaks.narrowPeak.bed.bed | Cistrome |
| Breast_Cancer | ESR1 | Zinc-coordinating DNA-binding domains | MCF-7 | GSM2747698 | 88372_sort_peaks.narrowPeak.bed.bed | Cistrome |
| Breast_Cancer | ESR1 | Zinc-coordinating DNA-binding domains | MCF-7 | GSM2747700 | 83433_sort_peaks.narrowPeak.bed.bed | Cistrome |
| Breast_Cancer | ESR1 | Zinc-coordinating DNA-binding domains | MCF-7 | GSM2747701 | 83434_sort_peaks.narrowPeak.bed.bed | Cistrome |
| Breast_Cancer | ESR1 | Zinc-coordinating DNA-binding domains | MCF-7 | GSM2747702 | 83431_sort_peaks.narrowPeak.bed.bed | Cistrome |
| Breast_Cancer | ESR1 | Zinc-coordinating DNA-binding domains | MCF-7 | GSM2747703 | 83432_sort_peaks.narrowPeak.bed.bed | Cistrome |
| Breast_Cancer | ESR1 | Zinc-coordinating DNA-binding domains | MCF-7 | GSM2747704 | 83429_sort_peaks.narrowPeak.bed.bed | Cistrome |
| Breast_Cancer | ESR1 | Zinc-coordinating DNA-binding domains | MCF-7 | GSM2747705 | 83430_sort_peaks.narrowPeak.bed.bed | Cistrome |
| Breast_Cancer | ESR1 | Zinc-coordinating DNA-binding domains | MCF-7 | GSM2747706 | 83427_sort_peaks.narrowPeak.bed.bed | Cistrome |
| Breast_Cancer | ESR1 | Zinc-coordinating DNA-binding domains | MCF-7 | GSM2747707 | 83428_sort_peaks.narrowPeak.bed.bed | Cistrome |
| Breast_Cancer | CTCF | Zinc-coordinating DNA-binding domains | MCF-7 | GSM2747710 | 82007_sort_peaks.narrowPeak.bed.bed | Cistrome |
| Breast_Cancer | CTCF | Zinc-coordinating DNA-binding domains | MCF-7 | GSM2747711 | 88132_sort_peaks.narrowPeak.bed.bed | Cistrome |
| Breast_Cancer | CTCF | Zinc-coordinating DNA-binding domains | MCF-7 | GSM2747712 | 87651_sort_peaks.narrowPeak.bed.bed | Cistrome |
| Breast_Cancer | CTCF | Zinc-coordinating DNA-binding domains | MCF-7 | GSM2747713 | 87650_sort_peaks.narrowPeak.bed.bed | Cistrome |
| Breast_Cancer | CTCF | Zinc-coordinating DNA-binding domains | MCF-7 | GSM2747714 | 87645_sort_peaks.narrowPeak.bed.bed | Cistrome |
| Breast_Cancer | CTCF | Zinc-coordinating DNA-binding domains | MCF-7 | GSM2747715 | 87644_sort_peaks.narrowPeak.bed.bed | Cistrome |
| Breast_Cancer | CTCF | Zinc-coordinating DNA-binding domains | MCF-7 | GSM2747716 | 87647_sort_peaks.narrowPeak.bed.bed | Cistrome |
| Breast_Cancer | CTCF | Zinc-coordinating DNA-binding domains | MCF-7 | GSM2747717 | 87646_sort_peaks.narrowPeak.bed.bed | Cistrome |
| Breast_Cancer | CTCF | Zinc-coordinating DNA-binding domains | MCF-7 | GSM2747720 | 83736_sort_peaks.narrowPeak.bed.bed | Cistrome |
| Breast_Cancer | CTCF | Zinc-coordinating DNA-binding domains | MCF-7 | GSM2747721 | 83737_sort_peaks.narrowPeak.bed.bed | Cistrome |
| Breast_Cancer | CTCF | Zinc-coordinating DNA-binding domains | MCF-7 | GSM2747722 | 83738_sort_peaks.narrowPeak.bed.bed | Cistrome |
| Breast_Cancer | CTCF | Zinc-coordinating DNA-binding domains | MCF-7 | GSM2747723 | 83739_sort_peaks.narrowPeak.bed.bed | Cistrome |
| Breast_Cancer | CTCF | Zinc-coordinating DNA-binding domains | MCF-7 | GSM2747724 | 83740_sort_peaks.narrowPeak.bed.bed | Cistrome |
| Breast_Cancer | CTCF | Zinc-coordinating DNA-binding domains | MCF-7 | GSM2747725 | 88917_sort_peaks.narrowPeak.bed.bed | Cistrome |
| Breast_Cancer | CTCF | Zinc-coordinating DNA-binding domains | MCF-7 | GSM2747726 | 83741_sort_peaks.narrowPeak.bed.bed | Cistrome |
| Breast_Cancer | CTCF | Zinc-coordinating DNA-binding domains | MCF-7 | GSM2747727 | 83742_sort_peaks.narrowPeak.bed.bed | Cistrome |
| Breast_Cancer | CTCF | Zinc-coordinating DNA-binding domains | MCF-7 | GSM2877977 | 88275_sort_peaks.narrowPeak.bed.bed | Cistrome |
| Breast_Cancer | CTCF | Zinc-coordinating DNA-binding domains | MCF-7 | GSM2877979 | 86828_sort_peaks.narrowPeak.bed.bed | Cistrome |
| Breast_Cancer | CTCF | Zinc-coordinating DNA-binding domains | MCF-7 | GSM2877980 | 83252_sort_peaks.narrowPeak.bed.bed | Cistrome |
| Breast_Cancer | CTCF | Zinc-coordinating DNA-binding domains | MCF-7 | GSM2877981 | 83253_sort_peaks.narrowPeak.bed.bed | Cistrome |
| Breast_Cancer | CTCF | Zinc-coordinating DNA-binding domains | MCF-7 | GSM2877982 | 83251_sort_peaks.narrowPeak.bed.bed | Cistrome |
| Breast_Cancer | CTCF | Zinc-coordinating DNA-binding domains | MCF-7 | GSM2877984 | 83249_sort_peaks.narrowPeak.bed.bed | Cistrome |
| Breast_Cancer | CTCF | Zinc-coordinating DNA-binding domains | MCF-7 | GSM2877985 | 84200_sort_peaks.narrowPeak.bed.bed | Cistrome |
| Breast_Cancer | CTCF | Zinc-coordinating DNA-binding domains | MCF-7 | GSM2877990 | 87487_sort_peaks.narrowPeak.bed.bed | Cistrome |
| Breast_Cancer | CTCF | Zinc-coordinating DNA-binding domains | MCF-7 | GSM2877993 | 87488_sort_peaks.narrowPeak.bed.bed | Cistrome |
| Breast_Cancer | ESR1 | Zinc-coordinating DNA-binding domains | MCF-7 | GSM2915375 | 87947_sort_peaks.narrowPeak.bed.bed | Cistrome |
| Breast_Cancer | ESR1 | Zinc-coordinating DNA-binding domains | MCF-7 | GSM2915376 | 84569_sort_peaks.narrowPeak.bed.bed | Cistrome |
| Breast_Cancer | ESR1 | Zinc-coordinating DNA-binding domains | MCF-7 | GSM2915377 | 87946_sort_peaks.narrowPeak.bed.bed | Cistrome |
| Breast_Cancer | ESR1 | Zinc-coordinating DNA-binding domains | MCF-7 | GSM2915378 | 87948_sort_peaks.narrowPeak.bed.bed | Cistrome |
| Breast_Cancer | ESR1 | Zinc-coordinating DNA-binding domains | MCF-7 | GSM2915379 | 84007_sort_peaks.narrowPeak.bed.bed | Cistrome |
| Breast_Cancer | ESR1 | Zinc-coordinating DNA-binding domains | MCF-7 | GSM2915380 | 83819_sort_peaks.narrowPeak.bed.bed | Cistrome |
| Breast_Cancer | ESR1 | Zinc-coordinating DNA-binding domains | MCF-7 | GSM2915381 | 83818_sort_peaks.narrowPeak.bed.bed | Cistrome |
| Breast_Cancer | ESR1 | Zinc-coordinating DNA-binding domains | MCF-7 | GSM2915382 | 83817_sort_peaks.narrowPeak.bed.bed | Cistrome |
| Breast_Cancer | ESR1 | Zinc-coordinating DNA-binding domains | MCF-7 | GSM2915383 | 83816_sort_peaks.narrowPeak.bed.bed | Cistrome |
| Breast_Cancer | ESR1 | Zinc-coordinating DNA-binding domains | MCF-7 | GSM2915384 | 83815_sort_peaks.narrowPeak.bed.bed | Cistrome |
| Breast_Cancer | ESR1 | Zinc-coordinating DNA-binding domains | MCF-7 | GSM2915385 | 83814_sort_peaks.narrowPeak.bed.bed | Cistrome |
| Breast_Cancer | ESR1 | Zinc-coordinating DNA-binding domains | MCF-7 | GSM2915386 | 83813_sort_peaks.narrowPeak.bed.bed | Cistrome |
| Breast_Cancer | ESR1 | Zinc-coordinating DNA-binding domains | MCF-7 | GSM2915387 | 83812_sort_peaks.narrowPeak.bed.bed | Cistrome |
| Breast_Cancer | ESR1 | Zinc-coordinating DNA-binding domains | MCF-7 | GSM2915388 | 83811_sort_peaks.narrowPeak.bed.bed | Cistrome |
| Breast_Cancer | ESR1 | Zinc-coordinating DNA-binding domains | MCF-7 | GSM2915389 | 83810_sort_peaks.narrowPeak.bed.bed | Cistrome |
| Breast_Cancer | ESR1 | Zinc-coordinating DNA-binding domains | MCF-7 | GSM2915390 | 87687_sort_peaks.narrowPeak.bed.bed | Cistrome |
| Breast_Cancer | ESR1 | Zinc-coordinating DNA-binding domains | MCF-7 | GSM2915391 | 87688_sort_peaks.narrowPeak.bed.bed | Cistrome |
| Breast_Cancer | ESR1 | Zinc-coordinating DNA-binding domains | MCF-7 | GSM2915392 | 87689_sort_peaks.narrowPeak.bed.bed | Cistrome |
| Breast_Cancer | ESR1 | Zinc-coordinating DNA-binding domains | MCF-7 | GSM2970402 | 83184_sort_peaks.narrowPeak.bed.bed | Cistrome |
| Breast_Cancer | ESR1 | Zinc-coordinating DNA-binding domains | MCF-7 | GSM2970403 | 83183_sort_peaks.narrowPeak.bed.bed | Cistrome |
| Breast_Cancer | ESR1 | Zinc-coordinating DNA-binding domains | MCF-7 | GSM2970404 | 83180_sort_peaks.narrowPeak.bed.bed | Cistrome |
| Breast_Cancer | ESR1 | Zinc-coordinating DNA-binding domains | MCF-7 | GSM2970405 | 83179_sort_peaks.narrowPeak.bed.bed | Cistrome |
| Breast_Cancer | ESR1 | Zinc-coordinating DNA-binding domains | MCF-7 | GSM2970406 | 83182_sort_peaks.narrowPeak.bed.bed | Cistrome |
| Breast_Cancer | ESR1 | Zinc-coordinating DNA-binding domains | MCF-7 | GSM2970407 | 85954_sort_peaks.narrowPeak.bed.bed | Cistrome |
| Breast_Cancer | ESR1 | Zinc-coordinating DNA-binding domains | MCF-7 | GSM2970408 | 83187_sort_peaks.narrowPeak.bed.bed | Cistrome |
| Breast_Cancer | ESR1 | Zinc-coordinating DNA-binding domains | MCF-7 | GSM2970409 | 83186_sort_peaks.narrowPeak.bed.bed | Cistrome |
| Breast_Cancer | ESR1 | Zinc-coordinating DNA-binding domains | MCF-7 | GSM2970410 | 87116_sort_peaks.narrowPeak.bed.bed | Cistrome |
| Breast_Cancer | ESR1 | Zinc-coordinating DNA-binding domains | MCF-7 | GSM2970411 | 87117_sort_peaks.narrowPeak.bed.bed | Cistrome |
| Breast_Cancer | ESR1 | Zinc-coordinating DNA-binding domains | MCF-7 | GSM2970412 | 87114_sort_peaks.narrowPeak.bed.bed | Cistrome |
| Breast_Cancer | ESR1 | Zinc-coordinating DNA-binding domains | MCF-7 | GSM2970413 | 87115_sort_peaks.narrowPeak.bed.bed | Cistrome |
| Breast_Cancer | GATA3 | Zinc-coordinating DNA-binding domains | MDA-MB-231 | GSM1855968 | 67519_sort_peaks.narrowPeak.bed.bed | Cistrome |
| Breast_Cancer | GATA3 | Zinc-coordinating DNA-binding domains | MDA-MB-231 | GSM1855969 | 67518_sort_peaks.narrowPeak.bed.bed | Cistrome |
| Breast_Cancer | GATA3 | Zinc-coordinating DNA-binding domains | MDA-MB-231 | GSM1855970 | 67517_sort_peaks.narrowPeak.bed.bed | Cistrome |
| Breast_Cancer | GATA3 | Zinc-coordinating DNA-binding domains | MDA-MB-231 | GSM1855971 | 67516_sort_peaks.narrowPeak.bed.bed | Cistrome |
| Breast_Cancer | GATA3 | Zinc-coordinating DNA-binding domains | MDA-MB-231 | GSM1856012 | 67503_sort_peaks.narrowPeak.bed.bed | Cistrome |
| Breast_Cancer | GATA3 | Zinc-coordinating DNA-binding domains | MDA-MB-231 | GSM1856013 | 67502_sort_peaks.narrowPeak.bed.bed | Cistrome |
| Breast_Cancer | GATA3 | Zinc-coordinating DNA-binding domains | MDA-MB-231 | GSM1856029 | 67501_sort_peaks.narrowPeak.bed.bed | Cistrome |
| Breast_Cancer | GATA3 | Zinc-coordinating DNA-binding domains | MDA-MB-231 | GSM1856030 | 67500_sort_peaks.narrowPeak.bed.bed | Cistrome |
| Breast_Cancer | GATA3 | Zinc-coordinating DNA-binding domains | MDA-MB-231 | GSM1856031 | 67499_sort_peaks.narrowPeak.bed.bed | Cistrome |
| Breast_Cancer | ESR1 | Zinc-coordinating DNA-binding domains | T-47D | GSM1967548 | 59376_sort_peaks.narrowPeak.bed.bed | Cistrome |
| Breast_Cancer | ESR1 | Zinc-coordinating DNA-binding domains | T-47D | GSM1967549 | 59377_sort_peaks.narrowPeak.bed.bed | Cistrome |
| Breast_Cancer | ESR1 | Zinc-coordinating DNA-binding domains | T-47D | GSM1967550 | 59378_sort_peaks.narrowPeak.bed.bed | Cistrome |
| Breast_Cancer | ESR1 | Zinc-coordinating DNA-binding domains | T-47D | GSM2112796 | 68976_sort_peaks.narrowPeak.bed.bed | Cistrome |
| Breast_Cancer | ESR1 | Zinc-coordinating DNA-binding domains | T-47D | GSM2112798 | 68974_sort_peaks.narrowPeak.bed.bed | Cistrome |
| Breast_Cancer | ESR1 | Zinc-coordinating DNA-binding domains | T-47D | GSM2112800 | 68972_sort_peaks.narrowPeak.bed.bed | Cistrome |
| Breast_Cancer | ESR1 | Zinc-coordinating DNA-binding domains | T-47D | GSM2112802 | 68970_sort_peaks.narrowPeak.bed.bed | Cistrome |
| Breast_Cancer | PGR | Zinc-coordinating DNA-binding domains | T-47D | GSM2112807 | 68969_sort_peaks.narrowPeak.bed.bed | Cistrome |
| Breast_Cancer | PGR | Zinc-coordinating DNA-binding domains | T-47D | GSM2112808 | 68968_sort_peaks.narrowPeak.bed.bed | Cistrome |
| Breast_Cancer | PGR | Zinc-coordinating DNA-binding domains | T-47D | GSM2112809 | 68967_sort_peaks.narrowPeak.bed.bed | Cistrome |
| Breast_Cancer | PGR | Zinc-coordinating DNA-binding domains | T-47D | GSM2112810 | 68966_sort_peaks.narrowPeak.bed.bed | Cistrome |
| Breast_Cancer | ESR1 | Zinc-coordinating DNA-binding domains | T-47D | GSM2644567 | 87200_sort_peaks.narrowPeak.bed.bed | Cistrome |
| Breast_Cancer | ESR1 | Zinc-coordinating DNA-binding domains | T-47D | GSM2644568 | 85565_sort_peaks.narrowPeak.bed.bed | Cistrome |
| Breast_Cancer | ESR1 | Zinc-coordinating DNA-binding domains | T-47D | GSM2644569 | 81169_sort_peaks.narrowPeak.bed.bed | Cistrome |
| Breast_Cancer | ESR1 | Zinc-coordinating DNA-binding domains | T-47D | GSM2644570 | 86340_sort_peaks.narrowPeak.bed.bed | Cistrome |
| Breast_Cancer | GATA3 | Zinc-coordinating DNA-binding domains | T-47D | GSM2644571 | 83266_sort_peaks.narrowPeak.bed.bed | Cistrome |
| Breast_Cancer | GATA3 | Zinc-coordinating DNA-binding domains | T-47D | GSM2644572 | 83265_sort_peaks.narrowPeak.bed.bed | Cistrome |
| Breast_Cancer | GATA3 | Zinc-coordinating DNA-binding domains | T-47D | GSM2644573 | 83264_sort_peaks.narrowPeak.bed.bed | Cistrome |
| Breast_Cancer | GATA3 | Zinc-coordinating DNA-binding domains | T-47D | GSM2644574 | 83271_sort_peaks.narrowPeak.bed.bed | Cistrome |
| Breast_Cancer | GATA3 | Zinc-coordinating DNA-binding domains | T-47D | GSM2644579 | 83258_sort_peaks.narrowPeak.bed.bed | Cistrome |
| Breast_Cancer | GATA3 | Zinc-coordinating DNA-binding domains | T-47D | GSM2644580 | 86316_sort_peaks.narrowPeak.bed.bed | Cistrome |
| Breast_Cancer | GATA3 | Zinc-coordinating DNA-binding domains | T-47D | GSM2644581 | 86317_sort_peaks.narrowPeak.bed.bed | Cistrome |
| Breast_Cancer | GATA3 | Zinc-coordinating DNA-binding domains | T-47D | GSM2644582 | 86315_sort_peaks.narrowPeak.bed.bed | Cistrome |
| Breast_Cancer | PGR | Zinc-coordinating DNA-binding domains | T-47D | GSM2644587 | 86312_sort_peaks.narrowPeak.bed.bed | Cistrome |
| Breast_Cancer | PGR | Zinc-coordinating DNA-binding domains | T-47D | GSM2644588 | 88447_sort_peaks.narrowPeak.bed.bed | Cistrome |
| Breast_Cancer | PGR | Zinc-coordinating DNA-binding domains | T-47D | GSM2644589 | 88449_sort_peaks.narrowPeak.bed.bed | Cistrome |
| Breast_Cancer | PGR | Zinc-coordinating DNA-binding domains | T-47D | GSM2644590 | 82472_sort_peaks.narrowPeak.bed.bed | Cistrome |
| Breast_Cancer | PGR | Zinc-coordinating DNA-binding domains | T-47D | GSM2644591 | 82471_sort_peaks.narrowPeak.bed.bed | Cistrome |
| Breast_Cancer | PGR | Zinc-coordinating DNA-binding domains | T-47D | GSM2644592 | 82474_sort_peaks.narrowPeak.bed.bed | Cistrome |
| Breast_Cancer | PGR | Zinc-coordinating DNA-binding domains | T-47D | GSM2644593 | 82473_sort_peaks.narrowPeak.bed.bed | Cistrome |
| Breast_Cancer | PGR | Zinc-coordinating DNA-binding domains | T-47D | GSM2644594 | 81339_sort_peaks.narrowPeak.bed.bed | Cistrome |
| Breast_Cancer | ESR1 | Zinc-coordinating DNA-binding domains | T-47D | GSM589238 | 2726_sort_peaks.narrowPeak.bed.bed | Cistrome |
| Breast_Cancer | ESR1 | Zinc-coordinating DNA-binding domains | T-47D | GSM589239 | 2725_sort_peaks.narrowPeak.bed.bed | Cistrome |
| Breast_Cancer | ESR1 | Zinc-coordinating DNA-binding domains | T-47D | GSM631489 | 6586_sort_peaks.narrowPeak.bed.bed | Cistrome |
| Breast_Cancer | ESR1 | Zinc-coordinating DNA-binding domains | T-47D | GSM631490 | 6587_sort_peaks.narrowPeak.bed.bed | Cistrome |
| Breast_Cancer | ESR1 | Zinc-coordinating DNA-binding domains | T-47D | GSM631491 | 6588_sort_peaks.narrowPeak.bed.bed | Cistrome |
| Breast_Cancer | ESR1 | Zinc-coordinating DNA-binding domains | T-47D | GSM631492 | 6589_sort_peaks.narrowPeak.bed.bed | Cistrome |
| Breast_Cancer | ESR1 | Zinc-coordinating DNA-binding domains | T-47D | GSM631493 | 6590_sort_peaks.narrowPeak.bed.bed | Cistrome |
| Breast_Cancer | ESR1 | Zinc-coordinating DNA-binding domains | T-47D | GSM798428 | 33513_sort_peaks.narrowPeak.bed.bed | Cistrome |
| Breast_Cancer | ESR1 | Zinc-coordinating DNA-binding domains | T-47D | GSM798429 | 33508_sort_peaks.narrowPeak.bed.bed | Cistrome |
| Breast_Cancer | PGR | Zinc-coordinating DNA-binding domains | T-47D | GSM999793 | 39767_sort_peaks.narrowPeak.bed.bed | Cistrome |
| Breast_Cancer | PGR | Zinc-coordinating DNA-binding domains | T-47D | GSM1017848 | 35591_sort_peaks.narrowPeak.bed.bed | Cistrome |
| Breast_Cancer | PGR | Zinc-coordinating DNA-binding domains | T-47D | GSM1017849 | 35584_sort_peaks.narrowPeak.bed.bed | Cistrome |
| Breast_Cancer | PGR | Zinc-coordinating DNA-binding domains | T-47D | GSM1017850 | 35586_sort_peaks.narrowPeak.bed.bed | Cistrome |
| Breast_Cancer | PGR | Zinc-coordinating DNA-binding domains | T-47D | GSM1017851 | 35590_sort_peaks.narrowPeak.bed.bed | Cistrome |
| Breast_Cancer | CTCF | Zinc-coordinating DNA-binding domains | T-47D | GSM1294054 | 47896_sort_peaks.narrowPeak.bed.bed | Cistrome |
| Breast_Cancer | CTCF | Zinc-coordinating DNA-binding domains | T-47D | GSM1294055 | 47897_sort_peaks.narrowPeak.bed.bed | Cistrome |
| Breast_Cancer | ESR1 | Zinc-coordinating DNA-binding domains | BT-474 | GSM798431 | 33484_sort_peaks.narrowPeak.bed.bed | Cistrome |
| Breast_Cancer | ESR1 | Zinc-coordinating DNA-binding domains | BT-474 | GSM798430 | 33512_sort_peaks.narrowPeak.bed.bed | Cistrome |
| Breast_Cancer | ESRRA | Zinc-coordinating DNA-binding domains | BT-474 | GSM1969223 | 67367_sort_peaks.narrowPeak.bed.bed | Cistrome |
| Breast_Cancer | ESR1 | Zinc-coordinating DNA-binding domains | H3396 | GSM811208 | 5539_sort_peaks.narrowPeak.bed.bed | Cistrome |
| Breast_Cancer | ESR1 | Zinc-coordinating DNA-binding domains | H3396 | GSM811209 | 5541_sort_peaks.narrowPeak.bed.bed | Cistrome |
| Breast_Cancer | ESR1 | Zinc-coordinating DNA-binding domains | H3396 | GSM811210 | 5542_sort_peaks.narrowPeak.bed.bed | Cistrome |
| Breast_Cancer | ESR1 | Zinc-coordinating DNA-binding domains | H3396 | GSM811211 | 5543_sort_peaks.narrowPeak.bed.bed | Cistrome |
| Breast_Cancer | ESR1 | Zinc-coordinating DNA-binding domains | H3396 | GSM811212 | 5544_sort_peaks.narrowPeak.bed.bed | Cistrome |
| Breast_Cancer | ESR1 | Zinc-coordinating DNA-binding domains | H3396 | GSM811213 | 5545_sort_peaks.narrowPeak.bed.bed | Cistrome |
| Breast_Cancer | ESR1 | Zinc-coordinating DNA-binding domains | H3396 | GSM811214 | 5546_sort_peaks.narrowPeak.bed.bed | Cistrome |
| Breast_Cancer | ESR1 | Zinc-coordinating DNA-binding domains | H3396 | GSM811215 | 5547_sort_peaks.narrowPeak.bed.bed | Cistrome |
| Breast_Cancer | ESR1 | Zinc-coordinating DNA-binding domains | H3396 | GSM801056 | 5555_sort_peaks.narrowPeak.bed.bed | Cistrome |
| Breast_Cancer | ESR1 | Zinc-coordinating DNA-binding domains | H3396 | GSM801057 | 5556_sort_peaks.narrowPeak.bed.bed | Cistrome |
| Breast_Cancer | ESR1 | Zinc-coordinating DNA-binding domains | MCF-7 | GSM1319626 | 68325_sort_peaks.narrowPeak.bed.bed | Cistrome |
| Breast_Cancer | ESR1 | Zinc-coordinating DNA-binding domains | MCF-7 | GSM1319625 | 68326_sort_peaks.narrowPeak.bed.bed | Cistrome |
| Breast_Cancer | ESR1 | Zinc-coordinating DNA-binding domains | MCF-7 | GSM1319624 | 68327_sort_peaks.narrowPeak.bed.bed | Cistrome |
| Breast_Cancer | ESR1 | Zinc-coordinating DNA-binding domains | MCF-7 | GSM1319623 | 68328_sort_peaks.narrowPeak.bed.bed | Cistrome |
| Breast_Cancer | ESR1 | Zinc-coordinating DNA-binding domains | MCF-7 | GSM2305316 | 74119_sort_peaks.narrowPeak.bed.bed | Cistrome |
| Breast_Cancer | ESR1 | Zinc-coordinating DNA-binding domains | MDA-MB-134 | GSM1235008 | 42615_sort_peaks.narrowPeak.bed.bed | Cistrome |
| Breast_Cancer | ESR1 | Zinc-coordinating DNA-binding domains | MDA-MB-134 | GSM1235010 | 42617_sort_peaks.narrowPeak.bed.bed | Cistrome |
| Breast_Cancer | AR | Zinc-coordinating DNA-binding domains | MDA-MB-453 | GSM1099030 | 36845_sort_peaks.narrowPeak.bed.bed | Cistrome |
| Breast_Cancer | AR | Zinc-coordinating DNA-binding domains | MDA-MB-453 | GSM1909086 | 57275_sort_peaks.narrowPeak.bed.bed | Cistrome |
| Breast_Cancer | AR | Zinc-coordinating DNA-binding domains | MDA-MB-453 | GSM1909087 | 57276_sort_peaks.narrowPeak.bed.bed | Cistrome |
| Breast_Cancer | AR | Zinc-coordinating DNA-binding domains | MDA-MB-453 | GSM1909088 | 57277_sort_peaks.narrowPeak.bed.bed | Cistrome |
| Breast_Cancer | AR | Zinc-coordinating DNA-binding domains | MDA-MB-453 | GSM1909089 | 57278_sort_peaks.narrowPeak.bed.bed | Cistrome |
| Breast_Cancer | AR | Zinc-coordinating DNA-binding domains | MDA-MB-453 | GSM1909090 | 57279_sort_peaks.narrowPeak.bed.bed | Cistrome |
| Breast_Cancer | AR | Zinc-coordinating DNA-binding domains | MDA-MB-453 | GSM1909091 | 57280_sort_peaks.narrowPeak.bed.bed | Cistrome |
| Breast_Cancer | AR | Zinc-coordinating DNA-binding domains | MDA-MB-453 | GSM1717863 | 70140_sort_peaks.narrowPeak.bed.bed | Cistrome |
| Breast_Cancer | AR | Zinc-coordinating DNA-binding domains | MDA-MB-453 | GSM1717862 | 70141_sort_peaks.narrowPeak.bed.bed | Cistrome |
| Breast_Cancer | AR | Zinc-coordinating DNA-binding domains | MDA-MB-453 | GSM1717861 | 70142_sort_peaks.narrowPeak.bed.bed | Cistrome |
| Breast_Cancer | AR | Zinc-coordinating DNA-binding domains | MDA-MB-453 | GSM1717860 | 70143_sort_peaks.narrowPeak.bed.bed | Cistrome |
| Breast_Cancer | ZBTB17 | Zinc-coordinating DNA-binding domains | MDA-MD-231 | GSM1181982 | 41831_sort_peaks.narrowPeak.bed.bed | Cistrome |
| Breast_Cancer | ESR1 | Zinc-coordinating DNA-binding domains | SUM44 | GSM2670868 | 86209_sort_peaks.narrowPeak.bed.bed | Cistrome |
| Breast_Cancer | ESR1 | Zinc-coordinating DNA-binding domains | SUM44 | GSM2670869 | 86210_sort_peaks.narrowPeak.bed.bed | Cistrome |
| Breast_Cancer | GATA3 | Zinc-coordinating DNA-binding domains | MCF-7 | GSM935445 | 45982_sort_peaks.narrowPeak.bed.bed | ENCODE |
| Breast_Cancer | ZNF217 | Zinc-coordinating DNA-binding domains | MCF-7 | GSM935563 | 45978_sort_peaks.narrowPeak.bed.bed | ENCODE |
| Breast_Cancer | ESR1 | Zinc-coordinating DNA-binding domains | T-47D | GSM1010823 | 46369_sort_peaks.narrowPeak.bed.bed | ENCODE |
| Breast_Cancer | CTCF | Zinc-coordinating DNA-binding domains | T-47D | GSM803348 | 46373_sort_peaks.narrowPeak.bed.bed | ENCODE |
| Breast_Cancer | ESR1 | Zinc-coordinating DNA-binding domains | T-47D | GSM803374 | 46375_sort_peaks.narrowPeak.bed.bed | ENCODE |
| Breast_Cancer | GATA3 | Zinc-coordinating DNA-binding domains | T-47D | GSM803514 | 46371_sort_peaks.narrowPeak.bed.bed | ENCODE |
| Breast_Cancer | ESR1 | Zinc-coordinating DNA-binding domains | T-47D | GSM803539 | 46374_sort_peaks.narrowPeak.bed.bed | ENCODE |
| Breast_Cancer | CTCF | Zinc-coordinating DNA-binding domains | MCF-7 | GSM1022658 | 45988_sort_peaks.narrowPeak.bed.bed | ENCODE |
| Breast_Cancer | CTCF | Zinc-coordinating DNA-binding domains | MCF-7 | GSM1022663 | 45987_sort_peaks.narrowPeak.bed.bed | ENCODE |
| Breast_Cancer | GATA3 | Zinc-coordinating DNA-binding domains | MCF-7 | GSM825711 | 45981_sort_peaks.narrowPeak.bed.bed | ENCODE |
| Breast_Cancer | CTCF | Zinc-coordinating DNA-binding domains | MCF-7 | GSM1010734 | 46318_sort_peaks.narrowPeak.bed.bed | ENCODE |
| Breast_Cancer | GATA3 | Zinc-coordinating DNA-binding domains | MCF-7 | GSM1010783 | 46310_sort_peaks.narrowPeak.bed.bed | ENCODE |
| Breast_Cancer | NR2F2 | Zinc-coordinating DNA-binding domains | MCF-7 | GSM1010837 | 46309_sort_peaks.narrowPeak.bed.bed | ENCODE |
| Breast_Cancer | EGR1 | Zinc-coordinating DNA-binding domains | MCF-7 | GSM1010844 | 46319_sort_peaks.narrowPeak.bed.bed | ENCODE |
| Breast_Cancer | REST | Zinc-coordinating DNA-binding domains | MCF-7 | GSM1010891 | 46313_sort_peaks.narrowPeak.bed.bed | ENCODE |
| Breast_Cancer | CTCF | Zinc-coordinating DNA-binding domains | MCF-7 | GSM822305 | 45983_sort_peaks.narrowPeak.bed.bed | ENCODE |
| Breast_Cancer | CTCF | Zinc-coordinating DNA-binding domains | MCF-7 | GSM822308 | 45994_sort_peaks.narrowPeak.bed.bed | ENCODE |
| Breast_Cancer | CTCF | Zinc-coordinating DNA-binding domains | MCF-7 | GSM822309 | 45995_sort_peaks.narrowPeak.bed.bed | ENCODE |
| Breast_Cancer | CTCF | Zinc-coordinating DNA-binding domains | MCF-7 | GSM1006875 | 45984_sort_peaks.narrowPeak.bed.bed | ENCODE |
| Breast_Cancer | CTCF | Zinc-coordinating DNA-binding domains | MCF-7 | GSM1006878 | 45992_sort_peaks.narrowPeak.bed.bed | ENCODE |
| Breast_Cancer | CTCF | Zinc-coordinating DNA-binding domains | MCF-7 | GSM808752 | 38194_sort_peaks.narrowPeak.bed.bed | ENCODE |
| Breast_Cancer | CTCF | Zinc-coordinating DNA-binding domains | MCF-7 | GSM808753 | 38203_sort_peaks.narrowPeak.bed.bed | ENCODE |
| Breast_Cancer | ZNF592 | Zinc-coordinating DNA-binding domains | MCF-7 | ENCSR028NUR_1 | 62567_sort_peaks.narrowPeak.bed.bed | ENCODE |
| Breast_Cancer | ZNF592 | Zinc-coordinating DNA-binding domains | MCF-7 | ENCSR028NUR_2 | 62568_sort_peaks.narrowPeak.bed.bed | ENCODE |
| Breast_Cancer | KLF9 | Zinc-coordinating DNA-binding domains | MCF-7 | ENCSR125ZYC_1 | 62799_sort_peaks.narrowPeak.bed.bed | ENCODE |
| Breast_Cancer | KLF9 | Zinc-coordinating DNA-binding domains | MCF-7 | ENCSR125ZYC_2 | 62800_sort_peaks.narrowPeak.bed.bed | ENCODE |
| Breast_Cancer | ZBTB11 | Zinc-coordinating DNA-binding domains | MCF-7 | ENCSR155VDK_1 | 62870_sort_peaks.narrowPeak.bed.bed | ENCODE |
| Breast_Cancer | ZBTB11 | Zinc-coordinating DNA-binding domains | MCF-7 | ENCSR155VDK_2 | 62871_sort_peaks.narrowPeak.bed.bed | ENCODE |
| Breast_Cancer | KLF4 | Zinc-coordinating DNA-binding domains | MCF-7 | ENCSR265WJC_1 | 63139_sort_peaks.narrowPeak.bed.bed | ENCODE |
| Breast_Cancer | KLF4 | Zinc-coordinating DNA-binding domains | MCF-7 | ENCSR265WJC_2 | 63140_sort_peaks.narrowPeak.bed.bed | ENCODE |
| Breast_Cancer | ZBTB40 | Zinc-coordinating DNA-binding domains | MCF-7 | ENCSR318LVG_1 | 63277_sort_peaks.narrowPeak.bed.bed | ENCODE |
| Breast_Cancer | ZBTB40 | Zinc-coordinating DNA-binding domains | MCF-7 | ENCSR318LVG_2 | 63278_sort_peaks.narrowPeak.bed.bed | ENCODE |
| Breast_Cancer | GATAD2B | Zinc-coordinating DNA-binding domains | MCF-7 | ENCSR389BLX_1 | 63454_sort_peaks.narrowPeak.bed.bed | ENCODE |
| Breast_Cancer | GATAD2B | Zinc-coordinating DNA-binding domains | MCF-7 | ENCSR389BLX_2 | 63455_sort_peaks.narrowPeak.bed.bed | ENCODE |
| Breast_Cancer | GATA3 | Zinc-coordinating DNA-binding domains | MCF-7 | ENCSR423RTK_1 | 63545_sort_peaks.narrowPeak.bed.bed | ENCODE |
| Breast_Cancer | GATA3 | Zinc-coordinating DNA-binding domains | MCF-7 | ENCSR423RTK_2 | 63546_sort_peaks.narrowPeak.bed.bed | ENCODE |
| Breast_Cancer | ZKSCAN1 | Zinc-coordinating DNA-binding domains | MCF-7 | ENCSR449UFF_1 | 63614_sort_peaks.narrowPeak.bed.bed | ENCODE |
| Breast_Cancer | ZKSCAN1 | Zinc-coordinating DNA-binding domains | MCF-7 | ENCSR449UFF_2 | 63615_sort_peaks.narrowPeak.bed.bed | ENCODE |
| Breast_Cancer | ZNF592 | Zinc-coordinating DNA-binding domains | MCF-7 | ENCSR701AQS_1 | 64278_sort_peaks.narrowPeak.bed.bed | ENCODE |
| Breast_Cancer | ZNF592 | Zinc-coordinating DNA-binding domains | MCF-7 | ENCSR701AQS_2 | 64279_sort_peaks.narrowPeak.bed.bed | ENCODE |
| Breast_Cancer | SP1 | Zinc-coordinating DNA-binding domains | MCF-7 | ENCSR729LGA_1 | 64350_sort_peaks.narrowPeak.bed.bed | ENCODE |
| Breast_Cancer | SP1 | Zinc-coordinating DNA-binding domains | MCF-7 | ENCSR729LGA_2 | 64351_sort_peaks.narrowPeak.bed.bed | ENCODE |
| Breast_Cancer | ZNF687 | Zinc-coordinating DNA-binding domains | MCF-7 | ENCSR899BKM_1 | 64786_sort_peaks.narrowPeak.bed.bed | ENCODE |
| Breast_Cancer | ZNF687 | Zinc-coordinating DNA-binding domains | MCF-7 | ENCSR899BKM_2 | 64787_sort_peaks.narrowPeak.bed.bed | ENCODE |
| Breast_Cancer | PGR | Zinc-coordinating DNA-binding domains | T-47D | GSM2112795 | EXP036357_liftoverhg19.bed | GTRD |
| Breast_Cancer | PGR | Zinc-coordinating DNA-binding domains | T-47D | GSM2112797 | EXP036359_liftoverhg19.bed | GTRD |
| Breast_Cancer | PGR | Zinc-coordinating DNA-binding domains | T-47D | GSM2112799 | EXP036361_liftoverhg19.bed | GTRD |
| Breast_Cancer | PGR | Zinc-coordinating DNA-binding domains | T-47D | GSM2112801 | EXP036363_liftoverhg19.bed | GTRD |
| Breast_Cancer | NR3C1 | Zinc-coordinating DNA-binding domains | MCF-7 | GSM2154989 | EXP037448_liftoverhg19.bed | GTRD |
| Breast_Cancer | NR3C1 | Zinc-coordinating DNA-binding domains | MCF-7 | GSM2154994 | EXP037453_liftoverhg19.bed | GTRD |
| Breast_Cancer | NR3C1 | Zinc-coordinating DNA-binding domains | MCF-7 | GSM2154995 | EXP037454_liftoverhg19.bed | GTRD |
| Breast_Cancer | ZNF701 | Zinc-coordinating DNA-binding domains | MCF-7 | GSM2574808 | EXP038565_liftoverhg19.bed | GTRD |
| Breast_Cancer | ZNF75A | Zinc-coordinating DNA-binding domains | MCF-7 | GSM2574809 | EXP038566_liftoverhg19.bed | GTRD |
| Breast_Cancer | ZXDC | Zinc-coordinating DNA-binding domains | MCF-7 | GSM2574810 | EXP038567_liftoverhg19.bed | GTRD |
| Breast_Cancer | ZSCAN2 | Zinc-coordinating DNA-binding domains | MCF-7 | GSM2574815 | EXP038570_liftoverhg19.bed | GTRD |
| Breast_Cancer | ESR1 | Zinc-coordinating DNA-binding domains | MCF-7 | GSE27300 | GSE27300.ESR1.MCF7_TLED_remap2018_hg19__v1_2.bed | Remap |
| Breast_Cancer | PGR | Zinc-coordinating DNA-binding domains | T-47D | GSE31129 | GSE31129.PGR.T47D_remap2018_hg19__v1_2.bed | Remap |
| Breast_Cancer | GATA3 | Zinc-coordinating DNA-binding domains | MCF-7 | GSE40129 | GSE40129.GATA3.MCF7_remap2018_hg19__v1_2.bed | Remap |
| Breast_Cancer | GATA3 | Zinc-coordinating DNA-binding domains | MCF-7 | GSE40129 | GSE40129.GATA3.MCF7_E2_remap2018_hg19__v1_2.bed | Remap |
| Breast_Cancer | ESR1 | Zinc-coordinating DNA-binding domains | MCF-7 | GSE40129 | GSE40129.ESR1.MCF7_SICTR_E2_remap2018_hg19__v1_2.bed | Remap |
| Breast_Cancer | ESR1 | Zinc-coordinating DNA-binding domains | MCF-7 | GSE40129 | GSE40129.ESR1.MCF7_SIGATA_E2_remap2018_hg19__v1_2.bed | Remap |
| Breast_Cancer | ESR1 | Zinc-coordinating DNA-binding domains | ZR-75-1 | GSE40129 | GSE40129.ESR1.ZR751_SICTR_E2_remap2018_hg19__v1_2.bed | Remap |
| Breast_Cancer | ESR1 | Zinc-coordinating DNA-binding domains | ZR-75-1 | GSE40129 | GSE40129.ESR1.ZR751_SIGATA_E2_remap2018_hg19__v1_2.bed | Remap |
| Breast_Cancer | PGR | Zinc-coordinating DNA-binding domains | T-47D | GSE40724 | GSE40724.PGR.T47D_remap2018_hg19__v1_2.bed | Remap |
| Breast_Cancer | CTCF | Zinc-coordinating DNA-binding domains | SUM159 | GSM1122667 | GSE46055.CTCF.SUM159_remap2018_hg19__v1_2.bed | Remap |
| Breast_Cancer | ESR2 | Zinc-coordinating DNA-binding domains | MCF-7 | GSE48096 | GSE48096.ESR2.MCF7_C412_E2_remap2018_hg19__v1_2.bed | Remap |
| Breast_Cancer | AR | Zinc-coordinating DNA-binding domains | MCF-7 | GSE48930 | GSE48930.AR.MCF7_remap2018_hg19__v1_2.bed | Remap |
| Breast_Cancer | GATA3 | Zinc-coordinating DNA-binding domains | MCF-7 | GSE51274 | GSE51274.GATA3.MCF7_remap2018_hg19__v1_2.bed | Remap |
| Breast_Cancer | GATA3 | Zinc-coordinating DNA-binding domains | T-47D | GSE51274 | GSE51274.GATA3.T47D_remap2018_hg19__v1_2.bed | Remap |
| Breast_Cancer | ESR1 | Zinc-coordinating DNA-binding domains | MCF-7 | GSE54592 | GSE54592.ESR1.MCF7_LETR_remap2018_hg19__v1_2.bed | Remap |
| Breast_Cancer | ESR1 | Zinc-coordinating DNA-binding domains | MCF-7 | GSE54592 | GSE54592.ESR1.MCF7_LETR_ANDRO_remap2018_hg19__v1_2.bed | Remap |
| Breast_Cancer | ESR1 | Zinc-coordinating DNA-binding domains | MCF-7 | GSE54592 | GSE54592.ESR1.MCF7_LY2_ETOH_remap2018_hg19__v1_2.bed | Remap |
| Breast_Cancer | ESR1 | Zinc-coordinating DNA-binding domains | T-47D | GSE68355 | GSE68355.ESR1.T47D_remap2018_hg19__v1_2.bed | Remap |
| Breast_Cancer | ESR1 | Zinc-coordinating DNA-binding domains | T-47D | GSE68356 | GSE68356.ESR1.T47D_E2_remap2018_hg19__v1_2.bed | Remap |
| Breast_Cancer | ESR1 | Zinc-coordinating DNA-binding domains | MCF-7 | GSE72249 | GSE72249.ESR1.MCF7_remap2018_hg19__v1_2.bed | Remap |
| Breast_Cancer | NR3C1 | Zinc-coordinating DNA-binding domains | MCF-7 | GSE72249 | GSE72249.NR3C1.MCF7_remap2018_hg19__v1_2.bed | Remap |
| Breast_Cancer | ESR1 | Zinc-coordinating DNA-binding domains | T-47D | GSE72249 | GSE72249.ESR1.T47D_remap2018_hg19__v1_2.bed | Remap |
| Breast_Cancer | NR3C1 | Zinc-coordinating DNA-binding domains | T-47D | GSE72249 | GSE72249.NR3C1.T47D_remap2018_hg19__v1_2.bed | Remap |
| Breast_Cancer | ESR1 | Zinc-coordinating DNA-binding domains | ZR-75-1 | GSE72249 | GSE72249.ESR1.ZR751_remap2018_hg19__v1_2.bed | Remap |
| Breast_Cancer | NR3C1 | Zinc-coordinating DNA-binding domains | ZR-75-1 | GSE72249 | GSE72249.NR3C1.ZR751_remap2018_hg19__v1_2.bed | Remap |
| Breast_Cancer | ZNF143 | Zinc-coordinating DNA-binding domains | MCF-7 | GSE76454 | GSE76454.ZNF143.MCF7_remap2018_hg19__v1_2.bed | Remap |
| Breast_Cancer | CTCF | Zinc-coordinating DNA-binding domains | MCF-7 | GSE78113 | GSE78113.CTCF.MCF7_HYPOXIA_remap2018_hg19__v1_2.bed | Remap |
| Breast_Cancer | CTCF | Zinc-coordinating DNA-binding domains | MCF-7 | GSE78113 | GSE78113.CTCF.MCF7_NORMOXIA_remap2018_hg19__v1_2.bed | Remap |
