# Supplementary material for: FunlncModel: integrating multi-omic features from upstream and downstream regulatory networks into a machine learning framework to identify functional lncRNAs
Source: Brief Bioinform. 2024 Nov 27;26(1):bbae623. doi: 10.1093/bib/bbae623 (PMC11601888; doi:10.1093/bib/bbae623)
Supplement: Supplementary_Table6_bbae623 [file supplementary_table6_bbae623.docx]

| **Supplementary Table 6. Histone modification ChIP-seq datasets** | | | | |
| --- | --- | --- | --- | --- |
| **Sample type** | **Sample name** | **Histone name** | **Series** | **Data sources** |
| Breast_Cancer | MCF-7 | H3K36me3 | ENCFF001VCW | ENCODE |
| Breast_Cancer | MCF-7 | H3K4me1 | ENCFF442KKC | ENCODE |
| Breast_Cancer | MCF-7 | H3K4me3 | ENCFF666IXO | ENCODE |
| Breast_Cancer | MCF-7 | H3K9ac | ENCFF383TDS | ENCODE |
| Breast_Cancer | MCF-7 | H4K20me1 | ENCFF611LFE | ENCODE |
| Colon_Cancer | Caco-2 | H3K36me3 | ENCFF001WWQ | ENCODE |
| Colon_Cancer | Caco-2 | H3K4me3 | ENCFF001WWV | ENCODE |
| Colon_Cancer | HCT116 | H3K36me3 | ENCFF467KXG | ENCODE |
| Colon_Cancer | HCT116 | H3K4me1 | ENCFF637OKJ | ENCODE |
| Colon_Cancer | HCT116 | H3K4me3 | ENCFF001XBX | ENCODE |
| Colon_Cancer | HCT116 | H3K79me2 | ENCFF680FOJ | ENCODE |
| Colon_Cancer | HCT116 | H3K9ac | ENCFF522QMX | ENCODE |
| Colon_Cancer | HCT116 | H4K20me1 | ENCFF331RWP | ENCODE |
| Lung_Cancer | A549 | H3K4me3 | ENCFF610TIN | ENCODE |
| Lung_Cancer | NCI-H1299 | H3K36me3 | ENCFF730FKD | ENCODE |
| Lung_Cancer | NCI-H1299 | H3K4me1 | ENCFF957YLY | ENCODE |
| Lung_Cancer | NCI-H1299 | H3K4me3 | ENCFF462DKM | ENCODE |
| Lung_Cancer | NCI-H1299 | H3K79me2 | ENCFF458LUV | ENCODE |
| Lung_Cancer | NCI-H1299 | H3K9ac | ENCFF121OGP | ENCODE |
| HESC | H1 | H3K36me3 | ENCFF932BMG | ENCODE |
| HESC | H1 | H3K4me1 | ENCFF993QNW | ENCODE |
| HESC | H1 | H3K4me3 | ENCFF076ZOZ | ENCODE |
| HESC | H1 | H3K79me2 | ENCFF270HKF | ENCODE |
| HESC | H1 | H3K9ac | ENCFF734XPE | ENCODE |
| HESC | H1 | H4K20me1 | ENCFF627QBJ | ENCODE |
| HESC | H1 | H3K36me3 | ENCFF803KZR | Roadmap |
| HESC | H1 | H3K4me1 | ENCFF448YRA | Roadmap |
| HESC | H1 | H3K4me3 | ENCFF695ZCD | Roadmap |
| HESC | H1 | H3K79me2 | ENCFF289PBD | Roadmap |
| HESC | H1 | H3K9ac | ENCFF426CVX | Roadmap |
| HESC | H7 | H3K36me3 | ENCFF040WYE | ENCODE |
| HESC | H7 | H3K4me3 | ENCFF001XAT | ENCODE |
| HESC | H7 | H3K4me3 | ENCFF471NKK | ENCODE |
| HESC | H7 | H3K36me3 | ENCFF351YFD | Roadmap |
| HESC | H7 | H3K4me1 | ENCFF526NYL | Roadmap |
| HESC | H7 | H3K9ac | ENCFF135LJC | Roadmap |
| HESC | H9 | H3K36me3 | ENCFF514RPR | ENCODE |
| HESC | H9 | H3K4me1 | ENCFF973TGZ | ENCODE |
| HESC | H9 | H3K36me3 | ENCFF239RJT | Roadmap |
| HESC | H9 | H3K4me1 | ENCFF156NDG | Roadmap |
| HESC | H9 | H3K4me3 | ENCFF386KUS | Roadmap |
| HESC | H9 | H3K79me2 | ENCFF053ZBU | Roadmap |
| HESC | H9 | H3K9ac | ENCFF554CUL | Roadmap |
| HESC | H9 | H4K20me1 | ENCFF737JLU | Roadmap |
| HESC | HUES48 | H3K36me3 | ENCFF616IPT | Roadmap |
| HESC | HUES48 | H3K4me1 | ENCFF246GUK | Roadmap |
| HESC | HUES48 | H3K4me3 | ENCFF123KKT | Roadmap |
| HESC | HUES48 | H3K9ac | ENCFF841QBM | Roadmap |
| HESC | HUES6 | H3K36me3 | ENCFF391TMG | Roadmap |
| HESC | HUES6 | H3K4me1 | ENCFF985WIX | Roadmap |
| HESC | HUES6 | H3K4me3 | ENCFF285GLL | Roadmap |
| HESC | HUES6 | H3K9ac | ENCFF606QXT | Roadmap |
| HESC | HUES64 | H3K36me3 | ENCFF513QJE | Roadmap |
| HESC | HUES64 | H3K4me1 | ENCFF687TLY | Roadmap |
| HESC | HUES64 | H3K4me3 | ENCFF168LOF | Roadmap |
| HESC | HUES64 | H3K9ac | ENCFF530IQL | Roadmap |
| HESC | iPS-11a | H3K4me3 | ENCFF325TPC | Roadmap |
| HESC | iPS-15b | H3K36me3 | ENCFF232VSL | Roadmap |
| HESC | iPS-15b | H3K4me1 | ENCFF270KZW | Roadmap |
| HESC | iPS-15b | H3K4me3 | ENCFF849MZZ | Roadmap |
| HESC | iPS-15b | H3K9ac | ENCFF308IAY | Roadmap |
| HESC | iPS-18a | H3K36me3 | ENCFF547YRE | Roadmap |
| HESC | iPS-18a | H3K4me1 | ENCFF047QAR | Roadmap |
| HESC | iPS-18a | H3K4me3 | ENCFF266KKC | Roadmap |
| HESC | iPS-18a | H3K9ac | ENCFF613SSG | Roadmap |
| HESC | iPS-18c | H3K4me3 | ENCFF616WQI | Roadmap |
| HESC | iPS-20b | H3K36me3 | ENCFF253BYH | Roadmap |
| HESC | iPS-20b | H3K4me1 | ENCFF520ETL | Roadmap |
| HESC | iPS-20b | H3K4me3 | ENCFF960CLR | Roadmap |
| HESC | iPS-20b | H3K9ac | ENCFF905ITQ | Roadmap |
| HESC | iPS-DF-19.11 | H3K36me3 | ENCFF126ZGD | Roadmap |
| HESC | iPS-DF-19.11 | H3K4me1 | ENCFF242HUS | Roadmap |
| HESC | iPS-DF-19.11 | H3K4me3 | ENCFF496WUV | Roadmap |
| HESC | iPS-DF-6.9 | H3K36me3 | ENCFF805YEX | Roadmap |
| HESC | iPS-DF-6.9 | H3K4me1 | ENCFF657KXD | Roadmap |
| HESC | iPS-DF-6.9 | H3K4me3 | ENCFF663TJR | Roadmap |
| HESC | UCSF-4 | H3K36me3 | ENCFF263FOB | Roadmap |
| HESC | UCSF-4 | H3K4me1 | ENCFF306ZJA | Roadmap |
| HESC | UCSF-4 | H3K4me3 | ENCFF413QBE | Roadmap |
