# Supplementary material for: FunlncModel: integrating multi-omic features from upstream and downstream regulatory networks into a machine learning framework to identify functional lncRNAs
Source: Brief Bioinform. 2024 Nov 27;26(1):bbae623. doi: 10.1093/bib/bbae623 (PMC11601888; doi:10.1093/bib/bbae623)
Supplement: Supplementary_Table7_bbae623 [file supplementary_table7_bbae623.docx]

| **Supplementary Table 7. Methylation 450k array datasets** | | | |
| --- | --- | --- | --- |
| **Sample type** | **Sample name** | **Series** | **Data sources** |
| HESC | H1 | - | ENCODE |
| Lung_Cancer | HRE | - | ENCODE |
| Lung_Cancer | A549 | - | ENCODE |
| Colon_Cancer | Caco-2 | - | ENCODE |
| Colon_Cancer | HCT116 | - | ENCODE |
| Breast_Cancer | MCF-7 | - | ENCODE |
| Breast_Cancer | T-47D | - | ENCODE |
