# Supplementary material for: FunlncModel: integrating multi-omic features from upstream and downstream regulatory networks into a machine learning framework to identify functional lncRNAs
Source: Brief Bioinform. 2024 Nov 27;26(1):bbae623. doi: 10.1093/bib/bbae623 (PMC11601888; doi:10.1093/bib/bbae623)
Supplement: Supplementary_Table8_bbae623 [file supplementary_table8_bbae623.docx]

| **Supplementary Table 8. 3D Chromatin interaction datasets** | | | |
| --- | --- | --- | --- |
| **Sample type** | **Sample name** | **Tissue** | **Data sources** |
| HESC | H1 | Embryo | 3D_4DGenome_018 |
| HESC | H1 | Embryo | 3D_4DGenome_019 |
| HESC | H1 | Embryo | 3D_4DGenome_020 |
| HESC | H1 | Embryo | 3D_4DGenome_021 |
| HESC | H1 | Embryo | 3D_4DGenome_022 |
| HESC | iPS-DF-19.11 | Embryo | 3D_4DGenome_032 |
| HESC | iPS-DF-6.9 | Embryo | 3D_4DGenome_033 |
| HESC | hESC | Embryo | 3D_Genome_Browser_001 |
| HESC | ES-I3 | Embryo | 3D_OncoBase_001 |
| HESC | ES-WA7 | Embryo | 3D_OncoBase_002 |
| HESC | H1 | Embryo | 3D_OncoBase_003 |
| HESC | H1 | Embryo | 3D_OncoBase_004 |
| HESC | H1 | Embryo | 3D_OncoBase_005 |
| HESC | H1 | Embryo | 3D_OncoBase_006 |
| HESC | H1 | Embryo | 3D_OncoBase_007 |
| HESC | H9 | Embryo | 3D_OncoBase_008 |
| HESC | H9 | Embryo | 3D_OncoBase_009 |
| HESC | H9 | Embryo | 3D_OncoBase_010 |
| HESC | hESC-Derived-CD184+-Endoderm-Cultured-Cells | Embryo | 3D_OncoBase_011 |
| HESC | hESC-Derived-CD56+-Ectoderm-Cultured-Cells | Embryo | 3D_OncoBase_012 |
| HESC | hESC-Derived-CD56+-Mesoderm-Cultured-Cells | Embryo | 3D_OncoBase_013 |
| HESC | HUES6 | Embryo | 3D_OncoBase_015 |
| HESC | HUES64 | Embryo | 3D_OncoBase_016 |
| HESC | iPS-15b | Embryo | 3D_OncoBase_018 |
| HESC | iPS-18 | Embryo | 3D_OncoBase_019 |
| HESC | iPS-20b | Embryo | 3D_OncoBase_020 |
| HESC | iPS-DF-6.9 | Embryo | 3D_OncoBase_021 |
| HESC | iPS-DF-19.11 | Embryo | 3D_OncoBase_022 |
| HESC | Mesenchymal | Embryo | 3D_OncoBase_023 |
| HESC | ES-UCSF4 | Embryo | 3D_OncoBase_024 |
| HESC | Mesenchymal | Embryo | 3D_OncoBase_025 |
| HESC | Mesenchymal | Embryo | 3D_OncoBase_026 |
| HESC | Mesenchymal | Embryo | 3D_OncoBase_049 |
| HESC | placenta | Embryo | 3D_OncoBase_089 |
| HESC | placenta | Embryo | 3D_OncoBase_097 |
| HESC | hESC | Embryo | 3DIV_030 |
| HESC | H1-Derived-Mesenchymal-Stem-Cells | Embryo | 3DIV_031 |
| HESC | H1-Derived-Mesoderm-Cells | Embryo | 3DIV_032 |
| HESC | H1-Derived-Neuronal-Progenitor-Cells | Embryo | 3DIV_033 |
| HESC | H1-Derived-Trophectoderm-Cells | Embryo | 3DIV_034 |
| HESC | H9-Embryonic-Stem-Cell | Embryo | 3DIV_035 |
| HESC | H9-Derived-Neuro-Ectodermal-Cells | Embryo | 3DIV_036 |
| Lung_Cancer | A549 | Lung | 3D_4DGenome_002 |
| Lung_Cancer | IMR-90 | Lung | 3D_4DGenome_031 |
| Lung_Cancer | A549 | Lung | 3D_Genome_Browser_003 |
| Lung_Cancer | NCI-H460 | Lung | 3D_Genome_Browser_009 |
| Lung_Cancer | IMR-90 | Lung | 3D_Genome_Browser_015 |
| Lung_Cancer | Lung | Lung | 3D_Genome_Browser_031 |
| Lung_Cancer | IMR-90 | Lung | 3D_OncoBase_017 |
| Lung_Cancer | Lung | Lung | 3D_OncoBase_086 |
| Lung_Cancer | Lung | Lung | 3D_OncoBase_094 |
| Lung_Cancer | A549 | Lung | 3D_OncoBase_127 |
| Lung_Cancer | A549 | Lung | 3DIV_001 |
| Lung_Cancer | A549 | Lung | 3DIV_002 |
| Lung_Cancer | A549 | Lung | 3DIV_003 |
| Lung_Cancer | A549 | Lung | 3DIV_004 |
| Lung_Cancer | A549 | Lung | 3DIV_005 |
| Lung_Cancer | A549 | Lung | 3DIV_006 |
| Lung_Cancer | hepatic sinusoid endothelial cell | Lung | 3DIV_013 |
| Lung_Cancer | IMR-90 | Lung | 3DIV_044 |
| Lung_Cancer | IMR-90 | Lung | 3DIV_045 |
| Lung_Cancer | IMR-90 | Lung | 3DIV_046 |
| Lung_Cancer | IMR-90 | Lung | 3DIV_047 |
| Lung_Cancer | Lung | Lung | 3DIV_053 |
| Lung_Cancer | NCI-H460 | Lung | 3DIV_060 |
| Colon_Cancer | Caco-2 | Colon | 3D_4DGenome_006 |
| Colon_Cancer | HCT116 | Colon | 3D_4DGenome_024 |
| Colon_Cancer | HCT116 | Colon | 3D_Genome_Browser_013 |
| Colon_Cancer | HCT116 | Colon | 3D_Genome_Browser_014 |
| Colon_Cancer | Colonic-Mucosa | Colon | 3D_OncoBase_073 |
| Colon_Cancer | Colon-Smooth-Muscle | Colon | 3D_OncoBase_074 |
| Colon_Cancer | sigmoid-colon | Colon | 3D_OncoBase_104 |
| Breast_Cancer | breast-epithelium | Breast | 3D_4DGenome_005 |
| Breast_Cancer | HCC1954 | Breast | 3D_4DGenome_023 |
| Breast_Cancer | HMEC | Breast | 3D_4DGenome_028 |
| Breast_Cancer | MCF-7 | Breast | 3D_4DGenome_039 |
| Breast_Cancer | HMEC | Breast | 3D_Genome_Browser_002 |
| Breast_Cancer | HMEC | Breast | 3D_Genome_Browser_021 |
| Breast_Cancer | Breast | Breast | 3D_OncoBase_027 |
| Breast_Cancer | vHMEC | Breast | 3D_OncoBase_028 |
| Breast_Cancer | HMEC | Breast | 3D_OncoBase_116 |
| Breast_Cancer | HMEC | Breast | 3DIV_042 |
| Breast_Cancer | MCF-10A | Breast | 3DIV_054 |
| Breast_Cancer | MCF-10A | Breast | 3DIV_055 |
| Breast_Cancer | MCF-10A | Breast | 3DIV_056 |
| Breast_Cancer | MCF-7 | Breast | 3DIV_057 |
| Breast_Cancer | MCF-7 | Breast | 3DIV_058 |
| Breast_Cancer | MCF-7 | Breast | 3DIV_059 |
