# Supplementary material for: FunlncModel: integrating multi-omic features from upstream and downstream regulatory networks into a machine learning framework to identify functional lncRNAs
Source: Brief Bioinform. 2024 Nov 27;26(1):bbae623. doi: 10.1093/bib/bbae623 (PMC11601888; doi:10.1093/bib/bbae623)
Supplement: Supplementary_Table9_bbae623 [file supplementary_table9_bbae623.docx]

| **Supplementary Table 9. Performance evaluation metrics** | | | |
| --- | --- | --- | --- |
| **Metrices** | **Description** | **Formula** | **Range** |
| AUROC | The ROC curve reflects the sensitivity and specificity at different thresholds for each prediction tools. For each ROC, the AUROC is a single scalar value, which reduces the complexity of the ROC curve. The higher AUROC stands for the better performance. | $\begin{aligned} \mathrm{AU}\mathrm{RO}C=\int_{0}^{1} \mathrm{TPR}\left( \mathrm{FPR}^{-1}\left( x \right) \right) dx \end{aligned}$ | 0 ~ 1 |
| AUPRC | The AUPRC summarizes a precision-recall curve as the weighted mean of precisions achieved at each threshold, with the increase in recall from the previous threshold used as the weight. The higher AUPRC stands for the better performance. | $\begin{aligned} \mathrm{AU}\mathrm{PR}C=\int_{0}^{1} \mathrm{Precision} dr \end{aligned}$ | 0 ~ 1 |

# Where $\mathrm{Precision}$ was the corresponding precision value when recall value is $r$. $\mathrm{TPR}$ and $\mathrm{FPR}$ represented the true positive rate and false positive rate values under the corresponding thresholds (See Method).
