# Supplementary material for: FunlncModel: integrating multi-omic features from upstream and downstream regulatory networks into a machine learning framework to identify functional lncRNAs
Source: Brief Bioinform. 2024 Nov 27;26(1):bbae623. doi: 10.1093/bib/bbae623 (PMC11601888; doi:10.1093/bib/bbae623)
Supplement: Supplementary_Table10_bbae623 [file supplementary_table10_bbae623.docx]

| **Supplementary Table 10. Performance evaluation for model based on sequence similarity splitting train-test** | | | | |
| --- | --- | --- | --- | --- |
| Fold | AUROC_TestSet | AUPRC_TestSet | AUROC_Cancer independent TestSet | AUPRC_Cancer independent TestSet |
| Fold1 | 0.845373134 | 0.900338551 | 0.892087742 | 0.725786584 |
| Fold2 | 0.832238806 | 0.898939046 | 0.898679656 | 0.731513072 |
| Fold3 | 0.859402985 | 0.932230691 | 0.836288132 | 0.637048192 |
| Fold4 | 0.852537313 | 0.906307828 | 0.892962905 | 0.742888573 |
| Fold5 | 0.846268657 | 0.91618815 | 0.866784009 | 0.692530766 |
| Fold6 | 0.851641791 | 0.921012205 | 0.883679313 | 0.723554109 |
| Fold7 | 0.845074627 | 0.912893329 | 0.884525059 | 0.722278651 |
| Fold8 | 0.851044776 | 0.912596416 | 0.897576509 | 0.740602045 |
| Fold9 | 0.875522388 | 0.942011928 | 0.877099656 | 0.72146832 |
| Fold10 | 0.861492537 | 0.928305426 | 0.827585531 | 0.626274712 |
