# Supplementary material for: FunlncModel: integrating multi-omic features from upstream and downstream regulatory networks into a machine learning framework to identify functional lncRNAs
Source: Brief Bioinform. 2024 Nov 27;26(1):bbae623. doi: 10.1093/bib/bbae623 (PMC11601888; doi:10.1093/bib/bbae623)
Supplement: Supplementary_Table11_bbae623 [file supplementary_table11_bbae623.docx]

| Supplementary Table 11. Method comparison result | | | | |
| --- | --- | --- | --- | --- |
| Method | Cancer-related | | Cancer cell growth-related | |
|  | AUROC | AUPRC | AUROC | AUPRC |
| FunlncModel | 0.8887522 | 0.616 | 0.910 | 0.679 |
| CapsNet-LDA | 0.693565 | 0.578 | 0.665 | 0.519 |
| LncDisease | 0.5120974 | 0.227 | - | - |
