# Supplementary material for: FunlncModel: integrating multi-omic features from upstream and downstream regulatory networks into a machine learning framework to identify functional lncRNAs
Source: Brief Bioinform. 2024 Nov 27;26(1):bbae623. doi: 10.1093/bib/bbae623 (PMC11601888; doi:10.1093/bib/bbae623)
Supplement: Supplementary_Table12_bbae623 [file supplementary_table12_bbae623.docx]

| Supplementary Table 12. The comparison with the gold standard set | | | | | | | |
| --- | --- | --- | --- | --- | --- | --- | --- |
| Breast cancer | | Colon cancer | | Lung cancer | | Cancer | |
| EVLncRNAs2  (the gold standard of known lncRNAs) | HCFun_lnc  (FuncLncModel) | EVLncRNAs2  (the gold standard of known lncRNAs) | HCFun_lnc(FuncLncModel) | EVLncRNAs2  (the gold standard of known lncRNAs) | HCFun_lnc(FuncLncModel) | EVLncRNAs2  (the gold standard of known lncRNAs) | common HCFun_lnc(FuncLncModel) |
| A2M-AS1 | A1BG-AS1 | AC078941.1 | AC009299.3 | AC016683.6 | A1BG-AS1 | A1BG-AS1 | A1BG-AS1 |
| AC026904.1 | AC078852.1 | AC105461.1 | AC026202.3 | AC026904.1 | AC009299.3 | A2M-AS1 | AC009299.3 |
| AC073284.4 | ADAMTS9-AS2 | AC123023.1 | AC078852.1 | ACTA2-AS1 | AFAP1-AS1 | AC005154.6 | AC078852.1 |
| ADAMTS9-AS2 | ADIRF-AS1 | ADAMTS9-AS2 | AP001372.2 | ADAMTS9-AS2 | ASB16-AS1 | AC005550.3 | ASB16-AS1 |
| ADPGK-AS1 | ADPGK-AS1 | ADPGK-AS1 | AP003774.1 | AFAP1-AS1 | BCDIN3D-AS1 | AC006129.1 | BCDIN3D-AS1 |
| AFAP1-AS1 | AGAP1-IT1 | AFAP1-AS1 | ASB16-AS1 | ASB16-AS1 | BOLA3-AS1 | AC007271.3 | BOLA3-AS1 |
| ARAP1-AS1 | AP001063.1 | ARAP1-AS1 | BOLA3-AS1 | BCAR4 | CACTIN-AS1 | AC008753.6 | CASC2 |
| ATXN8OS | ASB16-AS1 | BCAR4 | C1RL-AS1 | CASC11 | CASC2 | AC010761.9 | COX10-AS1 |
| BCAR4 | BCDIN3D-AS1 | BDNF-AS | CASC2 | CASC15 | CERS6-AS1 | AC016683.6 | CRNDE |
| BHLHE40-AS1 | BDNF-AS | CAHM | COX10-AS1 | CASC2 | COX10-AS1 | AC016735.2 | DANCR |
| CASC15 | BOLA3-AS1 | CASC15 | CRNDE | CASC8 | CRNDE | AC026904.1 | DHRS4-AS1 |
| CASC2 | CBR3-AS1 | CASC2 | DANCR | CASC9 | DANCR | AC067945.2 | DLEU2 |
| CASC9 | COX10-AS1 | CASC9 | DHRS4-AS1 | CDKN2B-AS1 | DHRS4-AS1 | AC073257.2 | ENTPD1-AS1 |
| CBR3-AS1 | DLEU2 | CBR3-AS1 | DLEU2 | CRNDE | DLEU2 | AC073284.4 | EPB41L4A-AS1 |
| CDKN2B-AS1 | ENTPD1-AS1 | CDKN2B-AS1 | DLX6-AS1 | DANCR | DLG1-AS1 | AC078941.1 | ERVK13-1 |
| CERS6-AS1 | EPB41L4A-AS1 | CRNDE | ENTPD1-AS1 | DDX11-AS1 | EPB41L4A-AS1 | AC079610.1 | FAM201A |
| CRNDE | FGD5-AS1 | CTA-941F9.9 | EPB41L4A-AS1 | DLEU2 | ERI3-IT1 | AC091729.7 | FENDRR |
| DANCR | FOXD2-AS1 | DANCR | ERVK13-1 | DLX6-AS1 | FAM201A | AC092159.2 | FGD5-AS1 |
| DICER1-AS1 | FOXN3-AS1 | DLX6-AS1 | FAM201A | DSCAM-AS1 | FENDRR | AC093818.1 | FOXD2-AS1 |
| DLX6-AS1 | H1FX-AS1 | EGOT | FAM66C | EGFR-AS1 | FGD5-AS1 | AC100830.4 | FOXN3-AS1 |
| DSCAM-AS1 | HCG17 | EHHADH-AS1 | FENDRR | FAM201A | FLG-AS1 | AC104699.1 | H1FX-AS1 |
| EGFLAM-AS1 | HCG18 | EZR-AS1 | GATA6-AS1 | FAM222A-AS1 | FOXD2-AS1 | AC105461.1 | HCG17 |
| EGOT | HCP5 | FBXL19-AS1 | H1FX-AS1 | FAM83A-AS1 | FOXN3-AS1 | AC114812.8 | HCG18 |
| EZR-AS1 | HNF1A-AS1 | FENDRR | HCG17 | FBXL19-AS1 | GABPB1-AS1 | AC123023.1 | HCP5 |
| FBXL19-AS1 | HOTAIRM1 | FEZF1-AS1 | HCG18 | FENDRR | GAS5 | AC132217.4 | HNF1A-AS1 |
| FENDRR | HOXA-AS3 | FGD5-AS1 | HCP5 | FEZF1-AS1 | H19 | ACTA2-AS1 | HOTAIR |
| FEZF1-AS1 | INTS6-AS1 | FOXD2-AS1 | HLA-F-AS1 | FGD5-AS1 | H1FX-AS1 | ADAMTS9-AS1 | HOTAIRM1 |
| FGF14-AS2 | JMJD1C-AS1 | GAS5 | HNF1A-AS1 | GAS5 | HAR1A | ADAMTS9-AS2 | HOXA11-AS |
| FOXD2-AS1 | KDM4A-AS1 | GHRLOS | HOTAIR | GAS5-AS1 | HCG17 | ADPGK-AS1 | HOXA-AS2 |
| GACAT1 | KIF9-AS1 | H19 | HOTAIRM1 | GAS6-AS1 | HCG18 | AFAP1-AS1 | INTS6-AS1 |
| GAS5 | KTN1-AS1 | HAND2-AS1 | HOTTIP | H19 | HOTAIR | AIRN | KCNQ1OT1 |
| H19 | LBX2-AS1 | HCG18 | HOXA11-AS | HAS2-AS1 | HOXA11-AS | AP000688.29 | KIF9-AS1 |
| HAND2-AS1 | LEF1-AS1 | HCP5 | HOXA-AS2 | HCP5 | HOXA-AS2 | AP001626.1 | KTN1-AS1 |
| HCP5 | LINC00092 | HIF1A-AS1 | HPN-AS1 | HIF1A-AS1 | HOXD-AS2 | AP003419.16 | LBX2-AS1 |
| HIF1A-AS2 | LINC00163 | HIF1A-AS2 | INTS6-AS1 | HNF1A-AS1 | INTS6-AS1 | ARAP1-AS1 | LIMD1-AS1 |
| HNF1A-AS1 | LINC00205 | HLA-F-AS1 | KCNQ1OT1 | HOTAIR | IQCH-AS1 | ARAP1-AS2 | LINC00163 |
| HOTAIR | LINC00242 | HNF1A-AS1 | LBX2-AS1 | HOTAIRM1 | KCNQ1OT1 | ARHGAP5-AS1 | LINC00205 |
| HOTAIRM1 | LINC00261 | HOTAIR | LIMD1-AS1 | HOTTIP | KIF9-AS1 | ASB16-AS1 | LINC00261 |
| HOTTIP | LINC00313 | HOTAIRM1 | LINC00163 | HOXA11-AS | KTN1-AS1 | ATXN8OS | LINC00313 |
| HOXA11-AS | LINC00324 | HOTTIP | LINC00173 | HOXA-AS2 | LIMD1-AS1 | BCAR4 | LINC00324 |
| HOXA-AS2 | LINC00482 | HOXA11-AS | LINC00205 | HOXA-AS3 | LINC00174 | BDNF-AS | LINC00467 |
| HOXC-AS3 | LINC00638 | HOXA-AS2 | LINC00265 | HOXB-AS3 | LINC00205 | BHLHE40-AS1 | LINC00472 |
| IGF2-AS | LINC00668 | HOXB-AS3 | LINC00313 | IGF2-AS | LINC00261 | BOK-AS1 | LINC00511 |
| KCNQ1OT1 | LINC00847 | IQCJ-SCHIP1-AS1 | LINC00319 | KCNQ1OT1 | LINC00461 | BPESC1 | LINC00574 |
| LIFR-AS1 | LINC00857 | ITIH4-AS1 | LINC00324 | KTN1-AS1 | LINC00467 | CAHM | LINC00638 |
| LINC00052 | LOXL1-AS1 | KCNQ1OT1 | LINC00339 | LEF1-AS1 | LINC00472 | CALML3-AS1 | LINC00668 |
| LINC00115 | MAGI1-IT1 | LDLRAD4-AS1 | LINC00467 | LIMD1-AS1 | LINC00511 | CASC11 | LINC00847 |
| LINC00160 | MAPKAPK5-AS1 | LEF1-AS1 | LINC00472 | LINC00173 | LINC00543 | CASC15 | LINC00857 |
| LINC00173 | MATN1-AS1 | LINC00052 | LINC00479 | LINC00210 | LINC00574 | CASC2 | LOXL1-AS1 |
| LINC00309 | MCF2L-AS1 | LINC00114 | LINC00511 | LINC00222 | LINC00662 | CASC8 | MAGI1-IT1 |
| LINC00310 | MCM3AP-AS1 | LINC00174 | LINC00574 | LINC00243 | LINC00668 | CASC9 | MAPKAPK5-AS1 |
| LINC00324 | MIR155HG | LINC00261 | LINC00638 | LINC00261 | LINC00689 | CBR3-AS1 | MCF2L-AS1 |
| LINC00339 | MIR497HG | LINC00265 | LINC00654 | LINC00273 | LINC00847 | CCDC26 | MCM3AP-AS1 |
| LINC00365 | NOP14-AS1 | LINC00324 | LINC00668 | LINC00313 | LINC00896 | CDKN2B-AS1 | MEG3 |
| LINC00458 | NR2F1-AS1 | LINC00339 | LINC00847 | LINC00319 | LOXL1-AS1 | CECR3 | MIR31HG |
| LINC00461 | NR2F2-AS1 | LINC00342 | LINC00857 | LINC00324 | MAGI2-AS3 | CECR9 | MIR497HG |
| LINC00472 | PRKAG2-AS1 | LINC00355 | LINC00899 | LINC00336 | MAPKAPK5-AS1 | CERS6-AS1 | MNX1-AS1 |
| LINC00511 | PRKCQ-AS1 | LINC00460 | LINC00963 | LINC00337 | MCM3AP-AS1 | CHL1-AS1 | NOP14-AS1 |
| LINC00518 | PTOV1-AS1 | LINC00467 | LINC01063 | LINC00339 | MEG3 | CHL1-AS2 | NR2F1-AS1 |
| LINC00520 | PVT1 | LINC00472 | LOXL1-AS1 | LINC00342 | MIR31HG | CLRN1-AS1 | NR2F2-AS1 |
| LINC00574 | RAMP2-AS1 | LINC00504 | LUCAT1 | LINC00355 | MIR497HG | CLYBL-AS2 | PINK1-AS |
| LINC00589 | RASAL2-AS1 | LINC00511 | MAGI1-IT1 | LINC00460 | MNX1-AS1 | CRNDE | PRKAG2-AS1 |
| LINC00598 | RMRP | LINC00518 | MALAT1 | LINC00461 | NNT-AS1 | CTA-941F9.9 | PRKCQ-AS1 |
| LINC00641 | SBF2-AS1 | LINC00520 | MCF2L-AS1 | LINC00466 | NOP14-AS1 | CTB-114C7.4 | PTOV1-AS1 |
| LINC00665 | SDCBP2-AS1 | LINC00525 | MEG3 | LINC00467 | NR2F1-AS1 | CTC-297N7.9 | PVT1 |
| LINC00668 | SLC26A4-AS1 | LINC00637 | MIR31HG | LINC00472 | NUTM2A-AS1 | CTD-2020K17.1 | RAMP2-AS1 |
| LINC00707 | SLX1A-SULT1A3 | LINC00659 | MIR497HG | LINC00485 | PART1 | CTD-3080P12.3 | RASAL2-AS1 |
| LINC00857 | SNHG1 | LINC00662 | MNX1-AS1 | LINC00504 | PINK1-AS | DANCR | RMRP |
| LINC00899 | SNHG10 | LINC00668 | NEAT1 | LINC00511 | PRKAG2-AS1 | DAOA-AS1 | RUSC1-AS1 |
| LINC00901 | SNHG11 | LINC00707 | NOP14-AS1 | LINC00519 | PRKCQ-AS1 | DBH-AS1 | SBF2-AS1 |
| LINC00963 | SNHG12 | LINC00858 | NR2F2-AS1 | LINC00525 | PTOV1-AS1 | DDX11-AS1 | SDCBP2-AS1 |
| LINC00968 | SNHG15 | LINC00958 | OIP5-AS1 | LINC00578 | PVT1 | DGCR9 | SLC26A4-AS1 |
| LINC01016 | SNHG3 | LINC00964 | PCAT6 | LINC00589 | RAMP2-AS1 | DGUOK-AS1 | SLX1A-SULT1A3 |
| LINC-ROR | SNHG5 | LINC00973 | PCBP1-AS1 | LINC00607 | RASAL2-AS1 | DHRS4-AS1 | SNHG1 |
| LOXL1-AS1 | SNHG6 | LINC01082 | PINK1-AS | LINC00662 | RBAKDN | DICER1-AS1 | SNHG10 |
| LUCAT1 | SNHG8 | LINC-ROR | PRKCQ-AS1 | LINC00665 | RMRP | DIO3OS | SNHG11 |
| MACC1-AS1 | SOCS2-AS1 | LUCAT1 | PRR7-AS1 | LINC00667 | RUSC1-AS1 | DLEU2 | SNHG12 |
| MAGI2-AS3 | SRRM2-AS1 | MAGI2-AS3 | PSMG3-AS1 | LINC00668 | RUVBL1-AS1 | DLG1-AS1 | SNHG15 |
| MALAT1 | TAPT1-AS1 | MALAT1 | PVT1 | LINC00691 | SDCBP2-AS1 | DLGAP1-AS1 | SNHG16 |
| MAPT-AS1 | THAP7-AS1 | MAPKAPK5-AS1 | RAMP2-AS1 | LINC00702 | SNHG1 | DLGAP1-AS2 | SNHG3 |
| MEG3 | TMEM161B-AS1 | MBNL1-AS1 | RASAL2-AS1 | LINC00707 | SNHG11 | DLX6-AS1 | SNHG5 |
| MIAT | TMPO-AS1 | MEG3 | RMRP | LINC00710 | SNHG12 | DNM3OS | SNHG8 |
| MIR210HG | TP53TG1 | MEG8 | RP11-831H9.3 | LINC00857 | SNHG14 | DSCAM-AS1 | SOCS2-AS1 |
| MIR22HG | TPT1-AS1 | MIAT | RUSC1-AS1 | LINC00858 | SNHG15 | EGFLAM-AS1 | SRRM2-AS1 |
| MIR31HG | TRAF3IP2-AS1 | MIR155HG | SBF2-AS1 | LINC00887 | SNHG3 | EGFR-AS1 | TAPT1-AS1 |
| MNX1-AS1 | UGDH-AS1 | MIR17HG | SDCBP2-AS1 | LINC00958 | SNHG7 | EGOT | THAP7-AS1 |
| NEAT1 | VIM-AS1 | MIR22HG | SH3RF3-AS1 | LINC00963 | SRRM2-AS1 | EHHADH-AS1 | TMEM161B-AS1 |
| NNT-AS1 | WAC-AS1 | MIR31HG | SLC26A4-AS1 | LINC00968 | TM4SF19-AS1 | EMX2OS | TMPO-AS1 |
| NR2F1-AS1 | ZBED3-AS1 | MIR600HG | SLX1A-SULT1A3 | LINC00970 | TMEM161B-AS1 | EPB41L4A-AS1 | TOB1-AS1 |
| OIP5-AS1 | ZEB1-AS1 | MNX1-AS1 | SNHG1 | LINC00987 | TOB1-AS1 | ESRG | TP53TG1 |
| PCAT1 | ZEB2-AS1 | NEAT1 | SNHG10 | LINC01088 | TP53TG1 | EZR-AS1 | TPT1-AS1 |
| PCAT6 | ZFAS1 | NNT-AS1 | SNHG11 | LINC-ROR | TRAF3IP2-AS1 | F11-AS1 | TRAF3IP2-AS1 |
| PDCD4-AS1 | ZNF503-AS2 | NR2F2-AS1 | SNHG12 | LOXL1-AS1 | TTC28-AS1 | FAM201A | UGDH-AS1 |
| PPP1R26-AS1 |  | OIP5-AS1 | SNHG15 | LUCAT1 | TUSC7 | FAM222A-AS1 | VIM-AS1 |
| PSMG3-AS1 |  | PART1 | SNHG16 | MACC1-AS1 | UGDH-AS1 | FAM66B | WAC-AS1 |
| PTCSC3 |  | PCAT1 | SNHG3 | MAGI2-AS3 | VIM-AS1 | FAM83A-AS1 | ZBED3-AS1 |
| PVT1 |  | PCAT6 | SNHG5 | MALAT1 | WAC-AS1 | FAM99A | ZEB1-AS1 |
| RAD51-AS1 |  | PGM5-AS1 | SNHG8 | MBNL1-AS1 | ZBED3-AS1 | FAM99B | ZFAS1 |
| RHPN1-AS1 |  | POU6F2-AS2 | SOCS2-AS1 | MCM3AP-AS1 | ZEB1-AS1 | FBXL19-AS1 | ZNF503-AS2 |
| RMST |  | PRKCQ-AS1 | SRRM2-AS1 | MEG3 | ZFAS1 | FENDRR |  |
| RPPH1 |  | PVT1 | SSSCA1-AS1 | MEG8 | ZNF667-AS1 | FEZF1-AS1 |  |
| RUSC1-AS1 |  | RHPN1-AS1 | TAPT1-AS1 | MIAT |  | FGD5-AS1 |  |
| SBF2-AS1 |  | RP11-317J10.2 | THAP7-AS1 | MIR17HG |  | FGF14-AS2 |  |
| SNHG1 |  | RP11-362K14.5 | TMEM161B-AS1 | MIR205HG |  | FOXD2-AS1 |  |
| SNHG12 |  | RP11-384P7.7 | TMPO-AS1 | MIR210HG |  | FOXP1-IT1 |  |
| SNHG14 |  | RP11-468E2.5 | TOB1-AS1 | MIR22HG |  | FRMD6-AS2 |  |
| SNHG15 |  | RP1-85F18.6 | TPT1-AS1 | MIR31HG |  | GABPB1-AS1 |  |
| SNHG16 |  | RP4-800J21.3 | TRAF3IP2-AS1 | MNX1-AS1 |  | GACAT1 |  |
| SNHG17 |  | RP5-881L22.5 | ZBED3-AS1 | NEAT1 |  | GAS5 |  |
| SNHG3 |  | RPPH1 | ZNF503-AS2 | NNT-AS1 |  | GAS5-AS1 |  |
| SNHG5 |  | SATB2-AS1 |  | NR2F2-AS1 |  | GAS6-AS1 |  |
| SNHG6 |  | SBF2-AS1 |  | NUTM2A-AS1 |  | GATA3-AS1 |  |
| SNHG7 |  | SNHG1 |  | OIP5-AS1 |  | GATA6-AS1 |  |
| SOX21-AS1 |  | SNHG12 |  | PART1 |  | GDNF-AS1 |  |
| SOX2-OT |  | SNHG14 |  | PCAT1 |  | GHRLOS |  |
| ST8SIA6-AS1 |  | SNHG15 |  | PCAT6 |  | GNG12-AS1 |  |
| STXBP5-AS1 |  | SNHG16 |  | PCAT7 |  | GUSBP11 |  |
| TFAP2A-AS1 |  | SNHG17 |  | PVT1 |  | H19 |  |
| TMPO-AS1 |  | SNHG3 |  | PXN-AS1 |  | HAND2-AS1 |  |
| TP53TG1 |  | SNHG5 |  | RGMB-AS1 |  | HAR1A |  |
| TTN-AS1 |  | SNHG6 |  | RHPN1-AS1 |  | HAR1B |  |
| UCA1 |  | SNHG7 |  | RMRP |  | HAS2-AS1 |  |
| UNC5B-AS1 |  | SOX21-AS1 |  | RP11-1008C21.2 |  | HCG11 |  |
| VIM-AS1 |  | SOX2-OT |  | RSF1-IT2 |  | HCG14 |  |
| WEE2-AS1 |  | ST3GAL6-AS1 |  | SBF2-AS1 |  | HCG15 |  |
| WT1-AS |  | ST8SIA6-AS1 |  | SFTA1P |  | HCG18 |  |
| ZEB1-AS1 |  | STARD13-AS |  | SNHG1 |  | HCP5 |  |
| ZEB2-AS1 |  | TMPO-AS1 |  | SNHG10 |  | HHIP-AS1 |  |
| ZFAS1 |  | TPT1-AS1 |  | SNHG11 |  | HIF1A-AS1 |  |
| ZFHX4-AS1 |  | TTN-AS1 |  | SNHG12 |  | HIF1A-AS2 |  |
| ZNF667-AS1 |  | TUSC7 |  | SNHG14 |  | HLA-AS1 |  |
|  |  | UCA1 |  | SNHG15 |  | HLA-F-AS1 |  |
|  |  | UNC5B-AS1 |  | SNHG16 |  | HNF1A-AS1 |  |
|  |  | VIM-AS1 |  | SNHG3 |  | HOTAIR |  |
|  |  | XIRP2-AS1 |  | SNHG5 |  | HOTAIRM1 |  |
|  |  | XXbac-B476C20.9 |  | SNHG6 |  | HOTTIP |  |
|  |  | ZEB1-AS1 |  | SNHG7 |  | HOXA11-AS |  |
|  |  | ZEB2-AS1 |  | SNHG8 |  | HOXA-AS2 |  |
|  |  | ZFAS1 |  | SOX21-AS1 |  | HOXA-AS3 |  |
|  |  | ZNF667-AS1 |  | SOX2-OT |  | HOXB-AS1 |  |
|  |  |  |  | ST8SIA6-AS1 |  | HOXB-AS3 |  |
|  |  |  |  | STXBP5-AS1 |  | HOXC-AS1 |  |
|  |  |  |  | TDRG1 |  | HOXC-AS2 |  |
|  |  |  |  | TERC |  | HOXC-AS3 |  |
|  |  |  |  | TMPO-AS1 |  | HOXD-AS2 |  |
|  |  |  |  | TNK2-AS1 |  | HTT-AS |  |
|  |  |  |  | TOB1-AS1 |  | IDH1-AS1 |  |
|  |  |  |  | TP53TG1 |  | IFNG-AS1 |  |
|  |  |  |  | TRHDE-AS1 |  | IGF2-AS |  |
|  |  |  |  | TTN-AS1 |  | IGFBP7-AS1 |  |
|  |  |  |  | TUSC7 |  | INHBA-AS1 |  |
|  |  |  |  | UCA1 |  | IQCJ-SCHIP1-AS1 |  |
|  |  |  |  | VPS9D1-AS1 |  | ITGB2-AS1 |  |
|  |  |  |  | WDFY3-AS2 |  | ITIH4-AS1 |  |
|  |  |  |  | WT1-AS |  | KCNQ1DN |  |
|  |  |  |  | ZEB1-AS1 |  | KCNQ1OT1 |  |
|  |  |  |  | ZEB2-AS1 |  | KIF9-AS1 |  |
|  |  |  |  | ZFAS1 |  | KTN1-AS1 |  |
|  |  |  |  | ZNF295-AS1 |  | LAMTOR5-AS1 |  |
|  |  |  |  | ZNF667-AS1 |  | LBX2-AS1 |  |
|  |  |  |  |  |  | LDLRAD4-AS1 |  |
|  |  |  |  |  |  | LEF1-AS1 |  |
|  |  |  |  |  |  | LIFR-AS1 |  |
|  |  |  |  |  |  | LIMD1-AS1 |  |
|  |  |  |  |  |  | LINC00028 |  |
|  |  |  |  |  |  | LINC00052 |  |
|  |  |  |  |  |  | LINC00114 |  |
|  |  |  |  |  |  | LINC00115 |  |
|  |  |  |  |  |  | LINC00160 |  |
|  |  |  |  |  |  | LINC00161 |  |
|  |  |  |  |  |  | LINC00167 |  |
|  |  |  |  |  |  | LINC00173 |  |
|  |  |  |  |  |  | LINC00174 |  |
|  |  |  |  |  |  | LINC00184 |  |
|  |  |  |  |  |  | LINC00205 |  |
|  |  |  |  |  |  | LINC00210 |  |
|  |  |  |  |  |  | LINC00222 |  |
|  |  |  |  |  |  | LINC00229 |  |
|  |  |  |  |  |  | LINC00237 |  |
|  |  |  |  |  |  | LINC00243 |  |
|  |  |  |  |  |  | LINC00261 |  |
|  |  |  |  |  |  | LINC00265 |  |
|  |  |  |  |  |  | LINC00271 |  |
|  |  |  |  |  |  | LINC00273 |  |
|  |  |  |  |  |  | LINC00293 |  |
|  |  |  |  |  |  | LINC00299 |  |
|  |  |  |  |  |  | LINC00304 |  |
|  |  |  |  |  |  | LINC00305 |  |
|  |  |  |  |  |  | LINC00309 |  |
|  |  |  |  |  |  | LINC00310 |  |
|  |  |  |  |  |  | LINC00311 |  |
|  |  |  |  |  |  | LINC00313 |  |
|  |  |  |  |  |  | LINC00319 |  |
|  |  |  |  |  |  | LINC00320 |  |
|  |  |  |  |  |  | LINC00323 |  |
|  |  |  |  |  |  | LINC00324 |  |
|  |  |  |  |  |  | LINC00336 |  |
|  |  |  |  |  |  | LINC00337 |  |
|  |  |  |  |  |  | LINC00339 |  |
|  |  |  |  |  |  | LINC00342 |  |
|  |  |  |  |  |  | LINC00355 |  |
|  |  |  |  |  |  | LINC00364 |  |
|  |  |  |  |  |  | LINC00365 |  |
|  |  |  |  |  |  | LINC00423 |  |
|  |  |  |  |  |  | LINC00449 |  |
|  |  |  |  |  |  | LINC00458 |  |
|  |  |  |  |  |  | LINC00459 |  |
|  |  |  |  |  |  | LINC00460 |  |
|  |  |  |  |  |  | LINC00461 |  |
|  |  |  |  |  |  | LINC00462 |  |
|  |  |  |  |  |  | LINC00466 |  |
|  |  |  |  |  |  | LINC00467 |  |
|  |  |  |  |  |  | LINC00470 |  |
|  |  |  |  |  |  | LINC00472 |  |
|  |  |  |  |  |  | LINC00477 |  |
|  |  |  |  |  |  | LINC00485 |  |
|  |  |  |  |  |  | LINC00486 |  |
|  |  |  |  |  |  | LINC00488 |  |
|  |  |  |  |  |  | LINC00491 |  |
|  |  |  |  |  |  | LINC00504 |  |
|  |  |  |  |  |  | LINC00511 |  |
|  |  |  |  |  |  | LINC00515 |  |
|  |  |  |  |  |  | LINC00518 |  |
|  |  |  |  |  |  | LINC00519 |  |
|  |  |  |  |  |  | LINC00520 |  |
|  |  |  |  |  |  | LINC00523 |  |
|  |  |  |  |  |  | LINC00525 |  |
|  |  |  |  |  |  | LINC00526 |  |
|  |  |  |  |  |  | LINC00536 |  |
|  |  |  |  |  |  | LINC00554 |  |
|  |  |  |  |  |  | LINC00565 |  |
|  |  |  |  |  |  | LINC00574 |  |
|  |  |  |  |  |  | LINC00578 |  |
|  |  |  |  |  |  | LINC00588 |  |
|  |  |  |  |  |  | LINC00589 |  |
|  |  |  |  |  |  | LINC00596 |  |
|  |  |  |  |  |  | LINC00598 |  |
|  |  |  |  |  |  | LINC00601 |  |
|  |  |  |  |  |  | LINC00607 |  |
|  |  |  |  |  |  | LINC00612 |  |
|  |  |  |  |  |  | LINC00635 |  |
|  |  |  |  |  |  | LINC00637 |  |
|  |  |  |  |  |  | LINC00639 |  |
|  |  |  |  |  |  | LINC00641 |  |
|  |  |  |  |  |  | LINC00645 |  |
|  |  |  |  |  |  | LINC00649 |  |
|  |  |  |  |  |  | LINC00659 |  |
|  |  |  |  |  |  | LINC00662 |  |
|  |  |  |  |  |  | LINC00663 |  |
|  |  |  |  |  |  | LINC00665 |  |
|  |  |  |  |  |  | LINC00667 |  |
|  |  |  |  |  |  | LINC00668 |  |
|  |  |  |  |  |  | LINC00671 |  |
|  |  |  |  |  |  | LINC00682 |  |
|  |  |  |  |  |  | LINC00689 |  |
|  |  |  |  |  |  | LINC00691 |  |
|  |  |  |  |  |  | LINC00702 |  |
|  |  |  |  |  |  | LINC00703 |  |
|  |  |  |  |  |  | LINC00707 |  |
|  |  |  |  |  |  | LINC00710 |  |
|  |  |  |  |  |  | LINC00839 |  |
|  |  |  |  |  |  | LINC00844 |  |
|  |  |  |  |  |  | LINC00857 |  |
|  |  |  |  |  |  | LINC00858 |  |
|  |  |  |  |  |  | LINC00880 |  |
|  |  |  |  |  |  | LINC00882 |  |
|  |  |  |  |  |  | LINC00886 |  |
|  |  |  |  |  |  | LINC00887 |  |
|  |  |  |  |  |  | LINC00899 |  |
|  |  |  |  |  |  | LINC00901 |  |
|  |  |  |  |  |  | LINC00909 |  |
|  |  |  |  |  |  | LINC00922 |  |
|  |  |  |  |  |  | LINC00929 |  |
|  |  |  |  |  |  | LINC00941 |  |
|  |  |  |  |  |  | LINC00942 |  |
|  |  |  |  |  |  | LINC00951 |  |
|  |  |  |  |  |  | LINC00958 |  |
|  |  |  |  |  |  | LINC00963 |  |
|  |  |  |  |  |  | LINC00964 |  |
|  |  |  |  |  |  | LINC00968 |  |
|  |  |  |  |  |  | LINC00970 |  |
|  |  |  |  |  |  | LINC00973 |  |
|  |  |  |  |  |  | LINC00974 |  |
|  |  |  |  |  |  | LINC00987 |  |
|  |  |  |  |  |  | LINC00994 |  |
|  |  |  |  |  |  | LINC01006 |  |
|  |  |  |  |  |  | LINC01013 |  |
|  |  |  |  |  |  | LINC01016 |  |
|  |  |  |  |  |  | LINC01018 |  |
|  |  |  |  |  |  | LINC01020 |  |
|  |  |  |  |  |  | LINC01048 |  |
|  |  |  |  |  |  | LINC01060 |  |
|  |  |  |  |  |  | LINC01082 |  |
|  |  |  |  |  |  | LINC01088 |  |
|  |  |  |  |  |  | LINC-ROR |  |
|  |  |  |  |  |  | LMCD1-AS1 |  |
|  |  |  |  |  |  | LOXL1-AS1 |  |
|  |  |  |  |  |  | LUCAT1 |  |
|  |  |  |  |  |  | LY86-AS1 |  |
|  |  |  |  |  |  | MACC1-AS1 |  |
|  |  |  |  |  |  | MAGI1-IT1 |  |
|  |  |  |  |  |  | MAGI2-AS3 |  |
|  |  |  |  |  |  | MALAT1 |  |
|  |  |  |  |  |  | MAMDC2-AS1 |  |
|  |  |  |  |  |  | MAPKAPK5-AS1 |  |
|  |  |  |  |  |  | MAPT-AS1 |  |
|  |  |  |  |  |  | MATN1-AS1 |  |
|  |  |  |  |  |  | MBNL1-AS1 |  |
|  |  |  |  |  |  | MCF2L-AS1 |  |
|  |  |  |  |  |  | MCM3AP-AS1 |  |
|  |  |  |  |  |  | MDC1-AS1 |  |
|  |  |  |  |  |  | MEF2C-AS1 |  |
|  |  |  |  |  |  | MEG3 |  |
|  |  |  |  |  |  | MEG8 |  |
|  |  |  |  |  |  | MIAT |  |
|  |  |  |  |  |  | MIR137HG |  |
|  |  |  |  |  |  | MIR155HG |  |
|  |  |  |  |  |  | MIR17HG |  |
|  |  |  |  |  |  | MIR205HG |  |
|  |  |  |  |  |  | MIR210HG |  |
|  |  |  |  |  |  | MIR22HG |  |
|  |  |  |  |  |  | MIR31HG |  |
|  |  |  |  |  |  | MIR497HG |  |
|  |  |  |  |  |  | MIR600HG |  |
|  |  |  |  |  |  | MIR7-3HG |  |
|  |  |  |  |  |  | MNX1-AS1 |  |
|  |  |  |  |  |  | MRPL23-AS1 |  |
|  |  |  |  |  |  | MYCNOS |  |
|  |  |  |  |  |  | MYLK-AS1 |  |
|  |  |  |  |  |  | NEAT1 |  |
|  |  |  |  |  |  | NEXN-AS1 |  |
|  |  |  |  |  |  | NNT-AS1 |  |
|  |  |  |  |  |  | NR2F1-AS1 |  |
|  |  |  |  |  |  | NR2F2-AS1 |  |
|  |  |  |  |  |  | NUTM2A-AS1 |  |
|  |  |  |  |  |  | OIP5-AS1 |  |
|  |  |  |  |  |  | OXCT1-AS1 |  |
|  |  |  |  |  |  | PART1 |  |
|  |  |  |  |  |  | PCA3 |  |
|  |  |  |  |  |  | PCAT1 |  |
|  |  |  |  |  |  | PCAT6 |  |
|  |  |  |  |  |  | PCAT7 |  |
|  |  |  |  |  |  | PCED1B-AS1 |  |
|  |  |  |  |  |  | PCGEM1 |  |
|  |  |  |  |  |  | PDCD4-AS1 |  |
|  |  |  |  |  |  | PDZRN3-AS1 |  |
|  |  |  |  |  |  | PGM5-AS1 |  |
|  |  |  |  |  |  | PINK1-AS |  |
|  |  |  |  |  |  | POU6F2-AS2 |  |
|  |  |  |  |  |  | PPP1R26-AS1 |  |
|  |  |  |  |  |  | PRKCQ-AS1 |  |
|  |  |  |  |  |  | PRKG1-AS1 |  |
|  |  |  |  |  |  | PROX1-AS1 |  |
|  |  |  |  |  |  | PRRT3-AS1 |  |
|  |  |  |  |  |  | PSMG3-AS1 |  |
|  |  |  |  |  |  | PSORS1C3 |  |
|  |  |  |  |  |  | PTCSC3 |  |
|  |  |  |  |  |  | PTOV1-AS1 |  |
|  |  |  |  |  |  | PTPRG-AS1 |  |
|  |  |  |  |  |  | PVT1 |  |
|  |  |  |  |  |  | PWRN2 |  |
|  |  |  |  |  |  | PXN-AS1 |  |
|  |  |  |  |  |  | RAB11B-AS1 |  |
|  |  |  |  |  |  | RAD51-AS1 |  |
|  |  |  |  |  |  | RAMP2-AS1 |  |
|  |  |  |  |  |  | RGMB-AS1 |  |
|  |  |  |  |  |  | RHPN1-AS1 |  |
|  |  |  |  |  |  | RMRP |  |
|  |  |  |  |  |  | RMST |  |
|  |  |  |  |  |  | RP11-1008C21.2 |  |
|  |  |  |  |  |  | RP11-110I1.14 |  |
|  |  |  |  |  |  | RP11-119F7.5 |  |
|  |  |  |  |  |  | RP11-134G8.8 |  |
|  |  |  |  |  |  | RP11-137H2.4 |  |
|  |  |  |  |  |  | RP11-159K7.2 |  |
|  |  |  |  |  |  | RP11-166P13.3 |  |
|  |  |  |  |  |  | RP11-169D4.1 |  |
|  |  |  |  |  |  | RP11-230G5.2 |  |
|  |  |  |  |  |  | RP11-284F21.9 |  |
|  |  |  |  |  |  | RP11-29G8.3 |  |
|  |  |  |  |  |  | RP11-2B6.2 |  |
|  |  |  |  |  |  | RP11-317J10.2 |  |
|  |  |  |  |  |  | RP11-33A14.1 |  |
|  |  |  |  |  |  | RP11-357H14.17 |  |
|  |  |  |  |  |  | RP11-362K14.5 |  |
|  |  |  |  |  |  | RP11-363E7.4 |  |
|  |  |  |  |  |  | RP11-366H4.1 |  |
|  |  |  |  |  |  | RP11-380D23.2 |  |
|  |  |  |  |  |  | RP11-381N20.2 |  |
|  |  |  |  |  |  | RP11-384P7.7 |  |
|  |  |  |  |  |  | RP11-390F4.3 |  |
|  |  |  |  |  |  | RP1-140A9.1 |  |
|  |  |  |  |  |  | RP1-140K8.5 |  |
|  |  |  |  |  |  | RP11-415D17.3 |  |
|  |  |  |  |  |  | RP11-423H2.3 |  |
|  |  |  |  |  |  | RP11-436H11.5 |  |
|  |  |  |  |  |  | RP11-468E2.5 |  |
|  |  |  |  |  |  | RP11-543N12.1 |  |
|  |  |  |  |  |  | RP11-552M11.4 |  |
|  |  |  |  |  |  | RP11-573G6.10 |  |
|  |  |  |  |  |  | RP11-597D13.9 |  |
|  |  |  |  |  |  | RP11-598D14.1 |  |
|  |  |  |  |  |  | RP11-626G11.3 |  |
|  |  |  |  |  |  | RP11-626H12.3 |  |
|  |  |  |  |  |  | RP11-69I8.3 |  |
|  |  |  |  |  |  | RP11-714G18.1 |  |
|  |  |  |  |  |  | RP11-766N7.3 |  |
|  |  |  |  |  |  | RP11-819C21.1 |  |
|  |  |  |  |  |  | RP11-81H3.2 |  |
|  |  |  |  |  |  | RP11-874J12.4 |  |
|  |  |  |  |  |  | RP11-87C12.5 |  |
|  |  |  |  |  |  | RP11-982M15.8 |  |
|  |  |  |  |  |  | RP1-261G23.7 |  |
|  |  |  |  |  |  | RP1-85F18.6 |  |
|  |  |  |  |  |  | RP3-413H6.2 |  |
|  |  |  |  |  |  | RP4-694A7.2 |  |
|  |  |  |  |  |  | RP4-800J21.3 |  |
|  |  |  |  |  |  | RP5-1120P11.3 |  |
|  |  |  |  |  |  | RP5-843L14.1 |  |
|  |  |  |  |  |  | RP5-875H18.4 |  |
|  |  |  |  |  |  | RP5-881L22.5 |  |
|  |  |  |  |  |  | RP6-65G23.1 |  |
|  |  |  |  |  |  | RPPH1 |  |
|  |  |  |  |  |  | RPS6KA2-AS1 |  |
|  |  |  |  |  |  | RSF1-IT2 |  |
|  |  |  |  |  |  | RUSC1-AS1 |  |
|  |  |  |  |  |  | SAP30L-AS1 |  |
|  |  |  |  |  |  | SATB2-AS1 |  |
|  |  |  |  |  |  | SBF2-AS1 |  |
|  |  |  |  |  |  | SEMA3B-AS1 |  |
|  |  |  |  |  |  | SFTA1P |  |
|  |  |  |  |  |  | SH3BP5-AS1 |  |
|  |  |  |  |  |  | SLC26A4-AS1 |  |
|  |  |  |  |  |  | SLC2A1-AS1 |  |
|  |  |  |  |  |  | SLC7A11-AS1 |  |
|  |  |  |  |  |  | SMAD5-AS1 |  |
|  |  |  |  |  |  | SMIM2-IT1 |  |
|  |  |  |  |  |  | SNHG1 |  |
|  |  |  |  |  |  | SNHG10 |  |
|  |  |  |  |  |  | SNHG11 |  |
|  |  |  |  |  |  | SNHG12 |  |
|  |  |  |  |  |  | SNHG14 |  |
|  |  |  |  |  |  | SNHG15 |  |
|  |  |  |  |  |  | SNHG16 |  |
|  |  |  |  |  |  | SNHG17 |  |
|  |  |  |  |  |  | SNHG18 |  |
|  |  |  |  |  |  | SNHG3 |  |
|  |  |  |  |  |  | SNHG5 |  |
|  |  |  |  |  |  | SNHG6 |  |
|  |  |  |  |  |  | SNHG7 |  |
|  |  |  |  |  |  | SNHG8 |  |
|  |  |  |  |  |  | SNHG9 |  |
|  |  |  |  |  |  | SOCS2-AS1 |  |
|  |  |  |  |  |  | SOX21-AS1 |  |
|  |  |  |  |  |  | SOX2-OT |  |
|  |  |  |  |  |  | SPAG5-AS1 |  |
|  |  |  |  |  |  | SSTR5-AS1 |  |
|  |  |  |  |  |  | ST3GAL6-AS1 |  |
|  |  |  |  |  |  | ST7-AS1 |  |
|  |  |  |  |  |  | ST7-AS2 |  |
|  |  |  |  |  |  | ST8SIA6-AS1 |  |
|  |  |  |  |  |  | STARD13-AS |  |
|  |  |  |  |  |  | STXBP5-AS1 |  |
|  |  |  |  |  |  | SYNE1-AS1 |  |
|  |  |  |  |  |  | TCL6 |  |
|  |  |  |  |  |  | TDRG1 |  |
|  |  |  |  |  |  | TERC |  |
|  |  |  |  |  |  | TFAP2A-AS1 |  |
|  |  |  |  |  |  | THAP9-AS1 |  |
|  |  |  |  |  |  | TMPO-AS1 |  |
|  |  |  |  |  |  | TNK2-AS1 |  |
|  |  |  |  |  |  | TNRC6C-AS1 |  |
|  |  |  |  |  |  | TOB1-AS1 |  |
|  |  |  |  |  |  | TP53TG1 |  |
|  |  |  |  |  |  | TPRG1-AS1 |  |
|  |  |  |  |  |  | TPT1-AS1 |  |
|  |  |  |  |  |  | TRAF3IP2-AS1 |  |
|  |  |  |  |  |  | TRHDE-AS1 |  |
|  |  |  |  |  |  | TRIM52-AS1 |  |
|  |  |  |  |  |  | TTC28-AS1 |  |
|  |  |  |  |  |  | TTLL11-IT1 |  |
|  |  |  |  |  |  | TTN-AS1 |  |
|  |  |  |  |  |  | TUSC7 |  |
|  |  |  |  |  |  | UCA1 |  |
|  |  |  |  |  |  | UCHL1-AS1 |  |
|  |  |  |  |  |  | UNC5B-AS1 |  |
|  |  |  |  |  |  | UPK1A-AS1 |  |
|  |  |  |  |  |  | VAV3-AS1 |  |
|  |  |  |  |  |  | VCAN-AS1 |  |
|  |  |  |  |  |  | VIM-AS1 |  |
|  |  |  |  |  |  | VPS9D1-AS1 |  |
|  |  |  |  |  |  | WDFY3-AS2 |  |
|  |  |  |  |  |  | WDR86-AS1 |  |
|  |  |  |  |  |  | WEE2-AS1 |  |
|  |  |  |  |  |  | WT1-AS |  |
|  |  |  |  |  |  | WWC2-AS1 |  |
|  |  |  |  |  |  | WWTR1-AS1 |  |
|  |  |  |  |  |  | XIRP2-AS1 |  |
|  |  |  |  |  |  | XXbac-B476C20.9 |  |
|  |  |  |  |  |  | XXYLT1-AS2 |  |
|  |  |  |  |  |  | ZBED3-AS1 |  |
|  |  |  |  |  |  | ZBTB20-AS4 |  |
|  |  |  |  |  |  | ZBTB40-IT1 |  |
|  |  |  |  |  |  | ZEB1-AS1 |  |
|  |  |  |  |  |  | ZEB2-AS1 |  |
|  |  |  |  |  |  | ZFAS1 |  |
|  |  |  |  |  |  | ZFAT-AS1 |  |
|  |  |  |  |  |  | ZFHX4-AS1 |  |
|  |  |  |  |  |  | ZNF295-AS1 |  |
|  |  |  |  |  |  | ZNF503-AS1 |  |
|  |  |  |  |  |  | ZNF667-AS1 |  |
