# Supplementary material for: FunlncModel: integrating multi-omic features from upstream and downstream regulatory networks into a machine learning framework to identify functional lncRNAs
Source: Brief Bioinform. 2024 Nov 27;26(1):bbae623. doi: 10.1093/bib/bbae623 (PMC11601888; doi:10.1093/bib/bbae623)
Supplement: Supplementary_Table13_bbae623 [file supplementary_table13_bbae623.docx]

| Supplementary Table 13. The positive and negative set across diverse samples | | | | | | | |
| --- | --- | --- | --- | --- | --- | --- | --- |
| Human embryonic stem cell | | Breast cancer | | Colon cancer | | Lung cancer | |
| Positive Set | Negative | Positive Set | Negative | Positive Set | Negative | Positive Set | Negative |
| AC005037.3 | AC002463.3 | AC008268.1 | AC000111.3 | AC105461.1 | AC000032.2 | AC005083.1 | AC010744.1 |
| AC005154.6 | AC004543.2 | AC026904.1 | AC003664.1 | CAHM | AC004702.2 | AC011526.1 | AC010761.6 |
| AC005387.3 | AC005022.1 | AC104135.3 | AC005276.1 | CASC11 | AC004869.2 | ACTA2-AS1 | AC010971.1 |
| AC007383.3 | AC005235.1 | ADARB2-AS1 | AC005538.5 | CBR3-AS1 | AC004869.3 | ADAMTS9-AS2 | AC011901.2 |
| AC009120.6 | AC005808.3 | AFAP1-AS1 | AC005597.1 | EHHADH-AS1 | AC005358.3 | CASC8 | AC012451.1 |
| AC009133.14 | AC009336.24 | AP000439.3 | AC006150.1 | FBXL19-AS1 | AC006037.2 | CTA-363E6.2 | AC013402.4 |
| AC010761.14 | AC010145.4 | BCAR4 | AC006196.1 | FOXD2-AS1 | AC006296.1 | DLX6-AS1 | AC013727.2 |
| AC015971.2 | AC010894.3 | CASC2 | AC006296.2 | GHRLOS | AC007271.3 | HCP5 | AC023128.1 |
| AC022154.7 | AC011288.2 | CCDC26 | AC007131.2 | HIF1A-AS1 | AC007349.7 | HNF1A-AS1 | AC062020.2 |
| AC022201.5 | AC012368.1 | CDKN2B-AS1 | AC007179.1 | HOXB-AS3 | AC009499.2 | HOTAIRM1 | AC062032.1 |
| AC062029.1 | AC016912.3 | CRNDE | AC007317.1 | HOXD-AS1 | AC011196.3 | HOXA-AS3 | AC064865.1 |
| AC074117.10 | AC016995.3 | CTD-2031P19.5 | AC007631.1 | KTN1-AS1 | AC011524.2 | LINC00115 | AC068542.2 |
| AC078883.4 | AC020743.4 | DANCR | AC007795.1 | LEF1-AS1 | AC011525.4 | LINC00222 | AC073834.3 |
| AC079807.2 | AC027119.1 | DSCAM-AS1 | AC008067.2 | LINC00174 | AC011754.1 | LINC00265 | AC079163.1 |
| AC084018.1 | AC063976.3 | EGOT | AC009110.1 | LINC00261 | AC011998.1 | LINC00313 | AC096554.1 |
| AC093391.2 | AC073218.2 | ERVH48-1 | AC009227.3 | LINC00460 | AC013269.3 | LINC00319 | AC096570.1 |
| AC093673.5 | AC080094.1 | FENDRR | AC010729.3 | LINC00659 | AC013401.1 | LINC00635 | AC226119.5 |
| AC106875.1 | AC083867.4 | FEZF1-AS1 | AC010745.1 | LINC00858 | AC013402.5 | LINC00707 | AJ239322.3 |
| ACVR2B-AS1 | AC084193.1 | FGF14-AS2 | AC010745.2 | LINC00964 | AC068483.1 | LINC00857 | AL050303.7 |
| AD000090.2 | AC092684.1 | GAS5 | AC011516.1 | LINC00973 | AC079799.2 | LINC00969 | AP000402.3 |
| AD000684.2 | AC098828.2 | GNG12-AS1 | AC011518.2 | LINC01021 | AC092660.1 | LINC00987 | AP000797.3 |
| AP000320.7 | AC104135.4 | H19 | AC011524.3 | MACROD2-AS1 | AC096559.2 | LINC01001 | AP001464.4 |
| BAIAP2-AS1 | AC106706.1 | HAR1A | AC013248.2 | MAFG-AS1 | AC104623.2 | LUCAT1 | ARAP1-AS1 |
| CASC15 | AC113617.1 | HCG11 | AC016903.1 | MAMDC2-AS1 | AC113607.3 | MIR137HG | CTA-363E6.7 |
| CKMT2-AS1 | AC114763.1 | HIF1A-AS2 | AC021021.2 | MIR155HG | AP000469.2 | MIR22HG | CTB-35F21.5 |
| COX10-AS1 | AC123023.1 | HOTAIR | AC062020.1 | PART1 | AP000477.2 | NEXN-AS1 | CTB-5E10.3 |
| CSTF3-AS1 | AE000658.31 | HOTTIP | AC062031.1 | RP1-13P20.6 | AP000998.2 | OIP5-AS1 | CTC-250I14.3 |
| CTA-445C9.14 | AF127936.3 | HOXA-AS2 | AC068489.1 | RP1-170O19.17 | AP001136.2 | PCAT1 | CTC-264O10.2 |
| CTA-984G1.5 | AP000459.7 | HOXA11-AS | AC068490.1 | RP11-134G8.8 | AP001465.5 | PCAT6 | CTC-313D10.1 |
| CTB-11I22.1 | AP000477.3 | ITGB2-AS1 | AC068542.1 | RP11-138J23.1 | AP001607.1 | RGMB-AS1 | CTC-518P12.6 |
| CTB-129P6.4 | AP001595.1 | KCNQ1OT1 | AC073928.2 | RP11-167H9.4 | CTA-249B10.1 | RP11-181G12.2 | CTD-2023J5.1 |
| CTC-250I14.6 | AP001628.7 | LINC-ROR | AC074019.1 | RP11-317J10.2 | CTA-280A3__B.2 | RP11-1C1.7 | CTD-2140G10.1 |
| CTC-338M12.4 | AP003774.6 | LINC00052 | AC079154.1 | RP11-392P7.6 | CTA-929C8.5 | RP11-284F21.7 | CTD-2161F6.2 |
| CTC-428G20.6 | AP004550.1 | LINC00152 | AC079630.2 | RP11-481J13.1 | CTB-105L4.2 | RP11-290F20.3 | CTD-2199O4.3 |
| CTD-2026K11.3 | CTB-43E15.3 | LINC00160 | AC092755.4 | RP11-650L12.2 | CTB-180C19.1 | RP11-325F22.5 | CTD-2244C20.2 |
| CTD-2037K23.2 | CTC-573N18.1 | LINC00310 | AC093911.1 | RP11-798K3.2 | CTC-297N7.10 | RP11-325I22.2 | CTD-2247C11.4 |
| CTD-2105E13.14 | CTD-2116N20.1 | LINC00323 | AC108056.1 | RP11-909B2.1 | CTC-400I9.1 | RP11-345L23.1 | CTD-2281E23.3 |
| CTD-2203K17.1 | CTD-2227I18.1 | LINC00339 | AC114730.7 | SNHG17 | CTC-525D6.5 | RP11-445K13.2 | CTD-2287N17.1 |
| CTD-2256P15.2 | CTD-2302E22.2 | LINC00472 | AC118345.1 | SNHG6 | CTC-552D5.1 | RP11-473M20.11 | CTD-2514K5.4 |
| CTD-2265O21.3 | CTD-2650P22.1 | LINC00511 | AC118653.2 | TP53TG1 | CTC-565M22.1 | RP11-50B3.2 | CTD-2531D15.5 |
| CTD-2265O21.7 | CTD-3035D6.2 | LINC00520 | AC133633.1 | TUSC7 | CTD-2019O4.1 | RP11-60H5.1 | CTD-2579N5.3 |
| CTD-2270L9.4 | CTD-3049M7.1 | LINC00574 | AC139712.4 | VIM-AS1 | CTD-2029E14.1 | RP11-89K21.1 | CTD-2616J11.9 |
| CTD-2287O16.5 | KB-1410C5.3 | LINC00598 | AF096876.1 | VPS9D1-AS1 | CTD-2043I16.1 | RP11-8L2.1 | CTD-3073N11.9 |
| CTD-2341M24.1 | LINC00211 | LINC00636 | AF241725.4 | XXbac-B476C20.9 | CTD-2046I8.1 | RP11-909N17.3 | CTD-3099C6.7 |
| CTD-2410N18.4 | LINC00364 | LINC00654 | AL133247.2 | ZEB1-AS1 | CTD-2124B20.2 | RP11-982M15.2 | CTD-3113P16.5 |
| CTD-2527I21.14 | LINC00392 | LINC00657 | AL157359.3 | ZNF582-AS1 | CTD-2140G10.4 | RP3-323P13.2 | FAM74A5 |
| CTD-2616J11.14 | LINC00618 | LINC00704 | AP000470.2 |  | CTD-2161F6.3 | RP5-826L7.1 | LINC00374 |
| CTD-2619J13.8 | LINC00924 | LINC00705 | AP000474.1 |  | CTD-2189E23.2 | SBF2-AS1 | LL22NC03-13G6.2 |
| CTD-2651B20.6 | LINC00942 | LINC00901 | AP000946.2 |  | CTD-2194D22.2 | SFTA1P | LL22NC03-32F9.1 |
| CTD-3065J16.9 | LINC00989 | LINC00917 | AP001172.3 |  | CTD-2194L12.3 | SNHG10 | NTM-IT |
| CTD-3184A7.4 | RP1-46F2.3 | LINC01016 | AP002856.4 |  | CTD-2272D18.1 | SNHG5 | RP1-317E23.7 |
| CTD-3193K9.3 | RP1-97J1.2 | MAGI2-AS3 | AY269186.2 |  | CTD-2308B18.2 | SOX21-AS1 | RP11-100F15.2 |
| DICER1-AS1 | RP11-1041F24.1 | MALAT1 | CTA-796E4.3 |  | CTD-2313F11.2 | TERC | RP11-1094M14.14 |
| DLG5-AS1 | RP11-1080G15.1 | MEG3 | CTB-191D16.1 |  | CTD-2313P7.1 | TM4SF1-AS1 | RP11-1144P22.1 |
| DYNLL1-AS1 | RP11-1114I9.1 | MIAT | CTB-32P11.1 |  | CTD-2382H12.2 | TMPO-AS1 | RP11-115F18.1 |
| EAF1-AS1 | RP11-111A21.1 | MIR31HG | CTB-37A13.1 |  | CTD-2526M8.3 | ZEB2-AS1 | RP11-115J16.3 |
| EIF2B5-AS1 | RP11-125M16.1 | MNX1-AS1 | CTB-89H12.4 |  | CTD-2534J5.1 | ZNF295-AS1 | RP11-118G23.2 |
| EIF3J-AS1 | RP11-134O21.1 | NEAT1 | CTC-264O10.1 |  | CTD-2545H1.1 |  | RP11-120A1.1 |
| EPB41L4A-AS1 | RP11-136I14.5 | NNT-AS1 | CTC-281M20.4 |  | CTD-2555I5.1 |  | RP11-123O1.1 |
| FAR1-IT1 | RP11-138B4.1 | PDCD4-AS1 | CTC-304I17.2 |  | GS1-278J22.2 |  | RP11-133L19.2 |
| GABPB1-AS1 | RP11-1398P2.1 | PINK1-AS | CTC-304I17.4 |  | LA16c-4G1.3 |  | RP11-136I14.2 |
| IL10RB-AS1 | RP11-141M1.3 | PPP1R26-AS1 | CTC-305H11.1 |  | LINC00387 |  | RP11-141A19.2 |
| IPO9-AS1 | RP11-148B18.3 | PTPRG-AS1 | CTC-369A16.2 |  | LINC00430 |  | RP11-141J10.2 |
| KB-1471A8.1 | RP11-157N3.1 | RMST | CTC-400I9.2 |  | LINC00577 |  | RP11-1437A8.3 |
| KCTD21-AS1 | RP11-160H22.5 | RP1-34M23.5 | CTC-400I9.3 |  | LINC00587 |  | RP11-1437A8.5 |
| KIF9-AS1 | RP11-166A12.1 | RP11-1060J15.4 | CTD-2006O16.2 |  | LINC01069 |  | RP11-143N13.2 |
| L34079.4 | RP11-189B4.6 | RP11-202K23.1 | CTD-2007H18.1 |  | LL22NC03-121E8.4 |  | RP11-149F8.5 |
| LA16c-313D11.12 | RP11-190J1.3 | RP11-360F5.1 | CTD-2014E2.2 |  | MDC1-AS1 |  | RP11-152O14.4 |
| LA16c-366D1.3 | RP11-197N18.7 | RP11-434D9.1 | CTD-2050I18.1 |  | NEGR1-IT1 |  | RP11-153F1.2 |
| LAMTOR5-AS1 | RP11-202G11.2 | RP11-445H22.4 | CTD-2050I18.2 |  | NRG3-AS1 |  | RP11-161H23.5 |
| LENG8-AS1 | RP11-204C23.1 | RP11-506D12.5 | CTD-2074D8.1 |  | PLSCR5-AS1 |  | RP11-173L6.1 |
| LIMD1-AS1 | RP11-213H15.1 | RP11-560G2.1 | CTD-2128A3.3 |  | RP1-177I10.1 |  | RP11-183E24.2 |
| LINC00263 | RP11-215P8.4 | RP4-591L5.2 | CTD-2232E5.2 |  | RP1-231P7P.1 |  | RP11-190C22.9 |
| LINC00338 | RP11-222A5.1 | RP4-613B23.1 | CTD-2243E23.1 |  | RP1-23E21.2 |  | RP11-192H23.5 |
| LINC00476 | RP11-227D13.4 | RPS6KA2-AS1 | CTD-2247C11.2 |  | RP1-251M9.3 |  | RP11-196P2.1 |
| LINC00649 | RP11-25I15.3 | SNHG14 | CTD-2247C11.5 |  | RP1-273G13.3 |  | RP11-199F6.4 |
| LINC00909 | RP11-265O12.1 | SNHG16 | CTD-2252P21.1 |  | RP1-37J18.2 |  | RP11-19P22.8 |
| LINC00937 | RP11-277P12.20 | SNHG7 | CTD-2296D1.3 |  | RP1-67K17.3 |  | RP11-202H2.1 |
| LINC00941 | RP11-307P5.1 | SOX2-OT | CTD-2308B18.1 |  | RP11-108P20.4 |  | RP11-203P2.2 |
| LRP4-AS1 | RP11-307P5.2 | ST8SIA6-AS1 | CTD-2318H23.1 |  | RP11-1094M14.12 |  | RP11-210G22.1 |
| MCM3AP-AS1 | RP11-312J18.6 | STXBP5-AS1 | CTD-2333M24.1 |  | RP11-1101K5.1 |  | RP11-219C24.10 |
| MIR302B | RP11-319E12.1 | UCA1 | CTD-2544H17.2 |  | RP11-114L10.2 |  | RP11-21A7A.3 |
| MIR940 | RP11-326C3.12 | WT1-AS | CTD-2560E9.5 |  | RP11-121L11.2 |  | RP11-231C18.1 |
| MYCNOS | RP11-329B9.1 | ZNF667-AS1 | CTD-2576F9.2 |  | RP11-124N14.3 |  | RP11-24N18.1 |
| OR2A1-AS1 | RP11-340A13.3 |  | CTD-3096M3.2 |  | RP11-127L21.1 |  | RP11-24P14.1 |
| PAN3-AS1 | RP11-347J14.7 |  | EGFLAM-AS3 |  | RP11-12K6.2 |  | RP11-252P19.2 |
| PAXBP1-AS1 | RP11-351N6.1 |  | GS1-278J22.1 |  | RP11-133I21.2 |  | RP11-254F7.3 |
| PCAT7 | RP11-364L4.3 |  | HLTF-AS1 |  | RP11-141A19.1 |  | RP11-259K5.2 |
| PCBP1-AS1 | RP11-364P22.2 |  | KB-1299A7.2 |  | RP11-142G1.3 |  | RP11-261P24.2 |
| PDXDC2P | RP11-366H4.1 |  | LINC00354 |  | RP11-149A7.2 |  | RP11-264B14.2 |
| POLR2J4 | RP11-383J24.1 |  | LINC00363 |  | RP11-14C22.6 |  | RP11-26L16.1 |
| PVT1 | RP11-384P7.5 |  | LINC00366 |  | RP11-152K4.2 |  | RP11-271F18.1 |
| PXN-AS1 | RP11-385E5.5 |  | LINC00396 |  | RP11-157B13.8 |  | RP11-279L11.1 |
| RAB11B-AS1 | RP11-406O16.1 |  | LINC00411 |  | RP11-159D8.1 |  | RP11-282C5.1 |
| RAD21-AS1 | RP11-414H23.2 |  | LINC00433 |  | RP11-160N1.9 |  | RP11-284H19.1 |
| RAD51-AS1 | RP11-416O18.1 |  | LINC00446 |  | RP11-163M18.1 |  | RP11-29B9.1 |
| RMRP | RP11-418B12.1 |  | LINC00459 |  | RP11-164N3.3 |  | RP11-2F20.1 |
| RNF139-AS1 | RP11-430H10.1 |  | LINC00529 |  | RP11-168C9.1 |  | RP11-300J18.2 |
| RNU11 | RP11-444P10.1 |  | LINC00644 |  | RP11-168E17.1 |  | RP11-313F23.3 |
| RP1-167A14.2 | RP11-44D19.1 |  | LINC00903 |  | RP11-168O22.1 |  | RP11-317L10.1 |
| RP1-191J18.66 | RP11-44N21.1 |  | LINC01052 |  | RP11-171N4.4 |  | RP11-318I4.1 |
| RP1-283E3.8 | RP11-458K10.3 |  | LINC01066 |  | RP11-182J1.10 |  | RP11-321E8.4 |
| RP1-43E13.2 | RP11-459O1.2 |  | LINC01074 |  | RP11-20I23.11 |  | RP11-324F11.1 |
| RP11-107N15.1 | RP11-462L8.1 |  | LINC01075 |  | RP11-21G20.3 |  | RP11-338K17.8 |
| RP11-108L7.15 | RP11-468E2.5 |  | LINC01078 |  | RP11-227F19.2 |  | RP11-342C20.2 |
| RP11-108L7.4 | RP11-474D1.2 |  | LL22NC03-86D4.1 |  | RP11-232C2.3 |  | RP11-348M17.2 |
| RP11-1094M14.11 | RP11-478P10.1 |  | MAGI2-AS1 |  | RP11-247I13.11 |  | RP11-350A1.2 |
| RP11-10L12.4 | RP11-493L12.4 |  | MAGI2-AS2 |  | RP11-255M2.2 |  | RP11-351A20.2 |
| RP11-110I1.12 | RP11-495P10.3 |  | NRG1-IT3 |  | RP11-258J10.1 |  | RP11-375D13.3 |
| RP11-115C21.2 | RP11-495P10.5 |  | PCDH9-AS4 |  | RP11-25I9.2 |  | RP11-378A13.2 |
| RP11-141B14.1 | RP11-4O3.1 |  | PPP2R2B-IT1 |  | RP11-264A11.1 |  | RP11-383M4.2 |
| RP11-146F11.1 | RP11-509J21.1 |  | ROPN1L-AS1 |  | RP11-26E5.1 |  | RP11-388M20.2 |
| RP11-152N13.5 | RP11-509J21.2 |  | RP1-149L1.1 |  | RP11-272J7.4 |  | RP11-398A8.4 |
| RP11-155O18.6 | RP11-513G11.3 |  | RP1-213J1P__B.2 |  | RP11-277K23.1 |  | RP11-3P17.5 |
| RP11-15B17.1 | RP11-547C5.2 |  | RP1-251M9.2 |  | RP11-27G24.3 |  | RP11-401E14.2 |
| RP11-15H20.7 | RP11-548L20.1 |  | RP1-288M22.2 |  | RP11-281J9.2 |  | RP11-407A16.4 |
| RP11-166B2.8 | RP11-57P19.1 |  | RP1-37J18.1 |  | RP11-284A20.2 |  | RP11-44H4.1 |
| RP11-168O16.1 | RP11-584P21.2 |  | RP1-84O15.2 |  | RP11-284A20.3 |  | RP11-452D21.1 |
| RP11-16C1.1 | RP11-605F22.2 |  | RP1-92C4.2 |  | RP11-284P20.3 |  | RP11-456H18.1 |
| RP11-18H7.1 | RP11-624J12.1 |  | RP11-100L22.3 |  | RP11-28O3.2 |  | RP11-457D2.3 |
| RP11-196G11.4 | RP11-634B22.4 |  | RP11-100M12.1 |  | RP11-292B1.2 |  | RP11-463J10.3 |
| RP11-20I23.7 | RP11-638I2.8 |  | RP11-101E14.2 |  | RP11-298E9.6 |  | RP11-468I15.1 |
| RP11-212P7.2 | RP11-669M16.1 |  | RP11-101E14.3 |  | RP11-298O21.3 |  | RP11-501O2.1 |
| RP11-227H4.5 | RP11-69G7.1 |  | RP11-103C3.1 |  | RP11-299D14.2 |  | RP11-503D12.1 |
| RP11-24C3.2 | RP11-714L20.1 |  | RP11-103H7.1 |  | RP11-2L8.2 |  | RP11-525J21.1 |
| RP11-258F1.1 | RP11-745L13.2 |  | RP11-1082A3.1 |  | RP11-301J16.7 |  | RP11-542C16.1 |
| RP11-267M23.4 | RP11-756H6.1 |  | RP11-1084A12.2 |  | RP11-30L8.1 |  | RP11-57A19.5 |
| RP11-267N12.3 | RP11-794G24.1 |  | RP11-109I13.2 |  | RP11-310I24.1 |  | RP11-586K12.8 |
| RP11-295G20.2 | RP11-802F5.1 |  | RP11-111D3.2 |  | RP11-313C4.1 |  | RP11-597A11.6 |
| RP11-298I3.1 | RP11-810P8.1 |  | RP11-113K21.4 |  | RP11-325D8.1 |  | RP11-605F22.1 |
| RP11-29G8.3 | RP11-834C11.4 |  | RP11-114O8.1 |  | RP11-335I12.2 |  | RP11-619J20.1 |
| RP11-29H23.4 | RP11-893F2.5 |  | RP11-115E19.1 |  | RP11-339A11.2 |  | RP11-626G11.3 |
| RP11-301G19.1 | RP11-89F3.2 |  | RP11-116A1.1 |  | RP11-340A13.1 |  | RP11-630C16.1 |
| RP11-301O19.1 | RP11-93K22.13 |  | RP11-118G23.1 |  | RP11-344F13.1 |  | RP11-664I21.5 |
| RP11-304L19.4 | RP11-95I16.2 |  | RP11-11K13.1 |  | RP11-344G13.1 |  | RP11-68I3.2 |
| RP11-314A20.5 | RP11-95P13.2 |  | RP11-1217F2.15 |  | RP11-344L13.2 |  | RP11-707G14.1 |
| RP11-315I20.1 | RP11-989F5.3 |  | RP11-123M21.1 |  | RP11-347L18.1 |  | RP11-70C1.3 |
| RP11-316M1.3 | RP3-340B19.3 |  | RP11-124I4.2 |  | RP11-34C15.2 |  | RP11-712L6.7 |
| RP11-319G6.1 | RP3-449M8.6 |  | RP11-128A17.1 |  | RP11-350F16.1 |  | RP11-717A5.2 |
| RP11-326K13.4 | RP4-583K8.1 |  | RP11-135A24.4 |  | RP11-355I22.5 |  | RP11-735B13.2 |
| RP11-356B19.11 | RP4-601P9.2 |  | RP11-137J7.2 |  | RP11-359D24.1 |  | RP11-736P16.1 |
| RP11-35O15.1 | RP4-614C15.2 |  | RP11-139J15.5 |  | RP11-35G9.5 |  | RP11-77B22.2 |
| RP11-363J17.1 | RP4-665J23.2 |  | RP11-140M13.1 |  | RP11-35O7.1 |  | RP11-801I18.1 |
| RP11-386G11.10 | RP4-710M16.2 |  | RP11-141J10.1 |  | RP11-361I14.2 |  | RP11-802H3.2 |
| RP11-387D10.2 | RP5-1065P14.2 |  | RP11-1437A8.6 |  | RP11-369E15.2 |  | RP11-846F4.12 |
| RP11-390E23.6 | RP5-1069C8.2 |  | RP11-143P4.2 |  | RP11-379F12.4 |  | RP11-863P13.1 |
| RP11-410D17.2 | RP5-836N10.1 |  | RP11-144G6.4 |  | RP11-38H17.1 |  | RP11-87P13.2 |
| RP11-417L19.4 | RP5-843L14.1 |  | RP11-148M9.1 |  | RP11-395F4.1 |  | RP11-97E7.1 |
| RP11-418J17.1 | snoU13 |  | RP11-14D22.1 |  | RP11-401H2.1 |  | RP13-616I3.1 |
| RP11-421L21.3 | TPRG1-AS1 |  | RP11-14O19.1 |  | RP11-404I7.1 |  | RP3-508I15.18 |
| RP11-452K12.7 | XXyac-YX155B6.5 |  | RP11-152C17.1 |  | RP11-407A16.7 |  | RP4-539M6.22 |
| RP11-467D6.1 |  |  | RP11-153N17.1 |  | RP11-408J6.1 |  | RP4-548D19.3 |
| RP11-469A15.2 |  |  | RP11-154H17.1 |  | RP11-40A13.1 |  | RP4-726F1.1 |
| RP11-474O21.5 |  |  | RP11-157P23.2 |  | RP11-40C11.2 |  | RP4-816N1.7 |
| RP11-486G15.2 |  |  | RP11-158J3.2 |  | RP11-421P23.2 |  | RP5-1050D4.3 |
| RP11-498B4.5 |  |  | RP11-15B24.1 |  | RP11-428L21.1 |  | RP5-827L5.1 |
| RP11-500C11.3 |  |  | RP11-164N3.2 |  | RP11-430L3.1 |  | RP5-827L5.2 |
| RP11-501C14.5 |  |  | RP11-167B3.1 |  | RP11-435M3.2 |  | RP5-905H7.9 |
| RP11-505K9.1 |  |  | RP11-167N24.5 |  | RP11-442O18.1 |  | RP5-947P14.1 |
| RP11-516C1.1 |  |  | RP11-16C1.3 |  | RP11-451L19.1 |  | U66059.58 |
| RP11-517P14.2 |  |  | RP11-16N2.1 |  | RP11-453M23.1 |  | WDFY3-AS1 |
| RP11-51J9.5 |  |  | RP11-171N4.3 |  | RP11-465K16.1 |  | ZBTB20-AS2 |
| RP11-524F11.2 |  |  | RP11-174M13.2 |  | RP11-467L24.1 |  | ZMYND10-AS1 |
| RP11-532F12.5 |  |  | RP11-17J14.2 |  | RP11-475D12.1 |  | AC002398.12 |
| RP11-53O19.1 |  |  | RP11-17M24.2 |  | RP11-47G4.2 |  | AC004837.4 |
| RP11-572O17.1 |  |  | RP11-182L7.1 |  | RP11-483K5.3 |  | AC007403.1 |
| RP11-573D15.9 |  |  | RP11-190I17.2 |  | RP11-489O18.1 |  | AC007948.1 |
| RP11-57A19.2 |  |  | RP11-190J23.1 |  | RP11-495O11.1 |  |  |
| RP11-580I16.2 |  |  | RP11-192C21.2 |  | RP11-4O3.2 |  |  |
| RP11-602N24.3 |  |  | RP11-19D2.1 |  | RP11-508O18.1 |  |  |
| RP11-611E13.2 |  |  | RP11-19D2.2 |  | RP11-523O18.7 |  |  |
| RP11-624G17.3 |  |  | RP11-1J7.1 |  | RP11-524K14.1 |  |  |
| RP11-644F5.11 |  |  | RP11-201M22.1 |  | RP11-526H11.1 |  |  |
| RP11-660L16.2 |  |  | RP11-202D1.3 |  | RP11-529E15.1 |  |  |
| RP11-676J15.1 |  |  | RP11-202D18.2 |  | RP11-529H2.1 |  |  |
| RP11-686O6.2 |  |  | RP11-202I11.2 |  | RP11-530I17.1 |  |  |
| RP11-692D12.1 |  |  | RP11-204N11.2 |  | RP11-531H8.2 |  |  |
| RP11-713M15.2 |  |  | RP11-208N20.1 |  | RP11-542A14.2 |  |  |
| RP11-727F15.11 |  |  | RP11-20L19.1 |  | RP11-566H8.1 |  |  |
| RP11-73E17.2 |  |  | RP11-225H22.7 |  | RP11-571O6.1 |  |  |
| RP11-746M1.1 |  |  | RP11-22D3.1 |  | RP11-574H6.1 |  |  |
| RP11-758P17.2 |  |  | RP11-22D3.2 |  | RP11-577G20.2 |  |  |
| RP11-758P17.3 |  |  | RP11-231C18.2 |  | RP11-586K12.4 |  |  |
| RP11-75C9.1 |  |  | RP11-232C2.2 |  | RP11-594C13.2 |  |  |
| RP11-773D16.1 |  |  | RP11-238K6.2 |  | RP11-5N11.5 |  |  |
| RP11-773H22.4 |  |  | RP11-23D5.1 |  | RP11-603J24.6 |  |  |
| RP11-79P5.9 |  |  | RP11-245A18.1 |  | RP11-618M23.2 |  |  |
| RP11-806H10.4 |  |  | RP11-248N22.2 |  | RP11-638L3.3 |  |  |
| RP11-849H4.4 |  |  | RP11-257I14.1 |  | RP11-64D24.2 |  |  |
| RP11-84G21.1 |  |  | RP11-258O13.1 |  | RP11-64I17.1 |  |  |
| RP11-967K21.1 |  |  | RP11-25O3.1 |  | RP11-659F24.1 |  |  |
| RP11-96H19.1 |  |  | RP11-261C10.1 |  | RP11-65D24.1 |  |  |
| RP11-977G19.11 |  |  | RP11-263E1.1 |  | RP11-663P9.1 |  |  |
| RP13-238F13.3 |  |  | RP11-26O3.1 |  | RP11-666I19.2 |  |  |
| RP13-726E6.2 |  |  | RP11-272B17.1 |  | RP11-667F9.2 |  |  |
| RP3-399L15.3 |  |  | RP11-273B19.1 |  | RP11-675M1.2 |  |  |
| RP3-430N8.11 |  |  | RP11-27G13.1 |  | RP11-706F1.2 |  |  |
| RP3-467N11.1 |  |  | RP11-281N10.1 |  | RP11-711C17.2 |  |  |
| RP3-508I15.9 |  |  | RP11-285B24.1 |  | RP11-719J20.1 |  |  |
| RP4-545C24.1 |  |  | RP11-290K4.1 |  | RP11-71J2.1 |  |  |
| RP4-549L20.3 |  |  | RP11-290L1.3 |  | RP11-730N24.2 |  |  |
| RP4-583P15.10 |  |  | RP11-295G12.1 |  | RP11-732A19.8 |  |  |
| RP4-584D14.5 |  |  | RP11-298H24.1 |  | RP11-74K19.1 |  |  |
| RP4-605O3.4 |  |  | RP11-29B9.2 |  | RP11-756P10.5 |  |  |
| RP4-616B8.4 |  |  | RP11-309H21.2 |  | RP11-75C23.1 |  |  |
| RP4-635E18.6 |  |  | RP11-30G8.2 |  | RP11-75C9.2 |  |  |
| RP4-717I23.3 |  |  | RP11-310I9.1 |  | RP11-760L24.1 |  |  |
| RP4-739H11.3 |  |  | RP11-313I2.11 |  | RP11-763K15.1 |  |  |
| RP4-792G4.2 |  |  | RP11-315A16.1 |  | RP11-775H9.2 |  |  |
| RP5-1024G6.2 |  |  | RP11-317O24.2 |  | RP11-777N19.1 |  |  |
| RP5-1165K10.2 |  |  | RP11-326C3.10 |  | RP11-778D12.2 |  |  |
| RP5-933K21.3 |  |  | RP11-328K15.1 |  | RP11-784B15.1 |  |  |
| RPL34-AS1 |  |  | RP11-332M4.1 |  | RP11-789C17.5 |  |  |
| RPPH1 |  |  | RP11-335O13.7 |  | RP11-78O22.1 |  |  |
| SMARCA5-AS1 |  |  | RP11-337L12.1 |  | RP11-799N11.1 |  |  |
| SNHG1 |  |  | RP11-33G16.1 |  | RP11-79C6.3 |  |  |
| SNHG12 |  |  | RP11-340I6.1 |  | RP11-80F22.4 |  |  |
| SNHG15 |  |  | RP11-343D2.11 |  | RP11-80K6.2 |  |  |
| SNHG3 |  |  | RP11-344A5.1 |  | RP11-84A1.3 |  |  |
| SNHG9 |  |  | RP11-344B2.2 |  | RP11-87N24.3 |  |  |
| SNORA67 |  |  | RP11-349E4.1 |  | RP11-90C4.2 |  |  |
| SNORA76 |  |  | RP11-349P19.1 |  | RP11-916L7.1 |  |  |
| SPAG5-AS1 |  |  | RP11-34N19.1 |  | RP11-93N20.1 |  |  |
| SRP14-AS1 |  |  | RP11-351A20.1 |  | RP11-94B19.5 |  |  |
| STARD4-AS1 |  |  | RP11-354O24.1 |  | RP11-95L3.2 |  |  |
| STX18-AS1 |  |  | RP11-357F12.1 |  | RP11-99C10.1 |  |  |
| TMEM161B-AS1 |  |  | RP11-357H3.1 |  | RP13-653N12.1 |  |  |
| TNKS2-AS1 |  |  | RP11-360L9.7 |  | RP3-331H24.4 |  |  |
| TRAF3IP2-AS1 |  |  | RP11-362A1.1 |  | RP3-495K2.1 |  |  |
| TTC28-AS1 |  |  | RP11-370P15.2 |  | RP4-553F4.2 |  |  |
| U47924.27 |  |  | RP11-374A22.1 |  | RP4-568F9.3 |  |  |
| UBA6-AS1 |  |  | RP11-374M1.4 |  | RP4-672N11.1 |  |  |
| UBL7-AS1 |  |  | RP11-375D13.4 |  | RP4-714D9.2 |  |  |
| WAC-AS1 |  |  | RP11-380F14.2 |  | RP4-742N3.1 |  |  |
| ZBTB11-AS1 |  |  | RP11-381K7.1 |  | RP4-764O22.1 |  |  |
| ZFAS1 |  |  | RP11-384E22.1 |  | RP5-1024N4.2 |  |  |
| ZNF833P |  |  | RP11-386P4.1 |  | RP5-1099E6.3 |  |  |
| ZSCAN16-AS1 |  |  | RP11-389J22.3 |  | RP5-1111A8.3 |  |  |
|  |  |  | RP11-38C18.2 |  | RP5-837I24.4 |  |  |
|  |  |  | RP11-397H6.1 |  | RP5-893G23.1 |  |  |
|  |  |  | RP11-3P22.1 |  | TMEM212-AS1 |  |  |
|  |  |  | RP11-401I19.1 |  | VCAN-AS1 |  |  |
|  |  |  | RP11-401N18.1 |  | RP11-128P17.3 |  |  |
|  |  |  | RP11-417J1.1 |  | RP11-340I6.3 |  |  |
|  |  |  | RP11-419C19.2 |  | RP11-347J14.8 |  |  |
|  |  |  | RP11-423J7.1 |  | RP5-1125M8.2 |  |  |
|  |  |  | RP11-434D2.12 |  |  |  |  |
|  |  |  | RP11-436F23.1 |  |  |  |  |
|  |  |  | RP11-437L7.1 |  |  |  |  |
|  |  |  | RP11-444A22.1 |  |  |  |  |
|  |  |  | RP11-445J9.1 |  |  |  |  |
|  |  |  | RP11-450H5.1 |  |  |  |  |
|  |  |  | RP11-452D21.2 |  |  |  |  |
|  |  |  | RP11-453O5.1 |  |  |  |  |
|  |  |  | RP11-457K10.1 |  |  |  |  |
|  |  |  | RP11-460H9.1 |  |  |  |  |
|  |  |  | RP11-463H12.2 |  |  |  |  |
|  |  |  | RP11-467I17.1 |  |  |  |  |
|  |  |  | RP11-478B9.1 |  |  |  |  |
|  |  |  | RP11-480O10.1 |  |  |  |  |
|  |  |  | RP11-503C24.3 |  |  |  |  |
|  |  |  | RP11-505C13.1 |  |  |  |  |
|  |  |  | RP11-520D19.1 |  |  |  |  |
|  |  |  | RP11-521E5.1 |  |  |  |  |
|  |  |  | RP11-521L9.1 |  |  |  |  |
|  |  |  | RP11-526P5.1 |  |  |  |  |
|  |  |  | RP11-526P6.1 |  |  |  |  |
|  |  |  | RP11-527D15.1 |  |  |  |  |
|  |  |  | RP11-535C7.1 |  |  |  |  |
|  |  |  | RP11-536C10.4 |  |  |  |  |
|  |  |  | RP11-538I12.2 |  |  |  |  |
|  |  |  | RP11-540N6.1 |  |  |  |  |
|  |  |  | RP11-542C10.1 |  |  |  |  |
|  |  |  | RP11-542F9.1 |  |  |  |  |
|  |  |  | RP11-543E8.1 |  |  |  |  |
|  |  |  | RP11-545I10.2 |  |  |  |  |
|  |  |  | RP11-545L5.1 |  |  |  |  |
|  |  |  | RP11-54O7.2 |  |  |  |  |
|  |  |  | RP11-552E20.1 |  |  |  |  |
|  |  |  | RP11-562F9.2 |  |  |  |  |
|  |  |  | RP11-563N6.6 |  |  |  |  |
|  |  |  | RP11-565A3.1 |  |  |  |  |
|  |  |  | RP11-567C20.3 |  |  |  |  |
|  |  |  | RP11-56L13.7 |  |  |  |  |
|  |  |  | RP11-573E11.2 |  |  |  |  |
|  |  |  | RP11-576M8.2 |  |  |  |  |
|  |  |  | RP11-577G20.1 |  |  |  |  |
|  |  |  | RP11-583F24.8 |  |  |  |  |
|  |  |  | RP11-586K12.10 |  |  |  |  |
|  |  |  | RP11-586K12.11 |  |  |  |  |
|  |  |  | RP11-587H10.2 |  |  |  |  |
|  |  |  | RP11-588P7.1 |  |  |  |  |
|  |  |  | RP11-588P7.2 |  |  |  |  |
|  |  |  | RP11-58O15.1 |  |  |  |  |
|  |  |  | RP11-593F5.2 |  |  |  |  |
|  |  |  | RP11-598C10.2 |  |  |  |  |
|  |  |  | RP11-59N23.1 |  |  |  |  |
|  |  |  | RP11-619L12.3 |  |  |  |  |
|  |  |  | RP11-619L19.2 |  |  |  |  |
|  |  |  | RP11-61D3.1 |  |  |  |  |
|  |  |  | RP11-622J8.1 |  |  |  |  |
|  |  |  | RP11-628O18.1 |  |  |  |  |
|  |  |  | RP11-632B21.1 |  |  |  |  |
|  |  |  | RP11-63C8.1 |  |  |  |  |
|  |  |  | RP11-650J17.2 |  |  |  |  |
|  |  |  | RP11-655H13.2 |  |  |  |  |
|  |  |  | RP11-669M2.1 |  |  |  |  |
|  |  |  | RP11-67L3.5 |  |  |  |  |
|  |  |  | RP11-680N20.1 |  |  |  |  |
|  |  |  | RP11-687D19.1 |  |  |  |  |
|  |  |  | RP11-69C17.2 |  |  |  |  |
|  |  |  | RP11-703C10.1 |  |  |  |  |
|  |  |  | RP11-707F2.1 |  |  |  |  |
|  |  |  | RP11-711C17.1 |  |  |  |  |
|  |  |  | RP11-719N22.2 |  |  |  |  |
|  |  |  | RP11-725M22.1 |  |  |  |  |
|  |  |  | RP11-732M18.2 |  |  |  |  |
|  |  |  | RP11-738G5.1 |  |  |  |  |
|  |  |  | RP11-73B2.2 |  |  |  |  |
|  |  |  | RP11-747D18.1 |  |  |  |  |
|  |  |  | RP11-751H17.1 |  |  |  |  |
|  |  |  | RP11-753D20.1 |  |  |  |  |
|  |  |  | RP11-756P10.4 |  |  |  |  |
|  |  |  | RP11-759A9.1 |  |  |  |  |
|  |  |  | RP11-774I5.1 |  |  |  |  |
|  |  |  | RP11-774O3.2 |  |  |  |  |
|  |  |  | RP11-775H9.1 |  |  |  |  |
|  |  |  | RP11-780O17.1 |  |  |  |  |
|  |  |  | RP11-785F11.1 |  |  |  |  |
|  |  |  | RP11-789A21.1 |  |  |  |  |
|  |  |  | RP11-793B23.1 |  |  |  |  |
|  |  |  | RP11-798K3.4 |  |  |  |  |
|  |  |  | RP11-806K15.1 |  |  |  |  |
|  |  |  | RP11-844P9.1 |  |  |  |  |
|  |  |  | RP11-883G14.3 |  |  |  |  |
|  |  |  | RP11-91N2.3 |  |  |  |  |
|  |  |  | RP11-93H12.2 |  |  |  |  |
|  |  |  | RP11-97E7.2 |  |  |  |  |
|  |  |  | RP11-98J9.3 |  |  |  |  |
|  |  |  | RP13-221M14.2 |  |  |  |  |
|  |  |  | RP13-436F16.1 |  |  |  |  |
|  |  |  | RP13-577H12.2 |  |  |  |  |
|  |  |  | RP3-463P15.1 |  |  |  |  |
|  |  |  | RP4-655C5.4 |  |  |  |  |
|  |  |  | RP4-678D15.1 |  |  |  |  |
|  |  |  | RP4-712E4.2 |  |  |  |  |
|  |  |  | RP4-718N17.2 |  |  |  |  |
|  |  |  | RP4-745K6.1 |  |  |  |  |
|  |  |  | RP4-788L20.3 |  |  |  |  |
|  |  |  | RP5-1006K12.1 |  |  |  |  |
|  |  |  | RP5-1048B16.1 |  |  |  |  |
|  |  |  | RP5-1051D14.1 |  |  |  |  |
|  |  |  | RP5-1097P24.1 |  |  |  |  |
|  |  |  | RP5-1103B4.3 |  |  |  |  |
|  |  |  | RP5-837I24.2 |  |  |  |  |
|  |  |  | RP5-837I24.6 |  |  |  |  |
|  |  |  | RP5-837M10.2 |  |  |  |  |
|  |  |  | RP5-933E2.1 |  |  |  |  |
|  |  |  | RP5-944M2.1 |  |  |  |  |
|  |  |  | RP5-986I17.2 |  |  |  |  |
|  |  |  | SCEL-AS1 |  |  |  |  |
|  |  |  | SLC9A9-AS2 |  |  |  |  |
|  |  |  | ZBTB20-AS3 |  |  |  |  |
|  |  |  | RP11-1049A21.2 |  |  |  |  |
|  |  |  | RP11-1437A8.4 |  |  |  |  |
