# Supplementary material for: FunlncModel: integrating multi-omic features from upstream and downstream regulatory networks into a machine learning framework to identify functional lncRNAs
Source: Brief Bioinform. 2024 Nov 27;26(1):bbae623. doi: 10.1093/bib/bbae623 (PMC11601888; doi:10.1093/bib/bbae623)
Supplement: Supplementary_Table14_bbae623 [file supplementary_table14_bbae623.docx]

| Supplementary Table 14. The list of HCFun_lnc in HESC | |
| --- | --- |
| HCFun_lnc | LNCipedia High confidence set |
| AC002454.1 | Yes |
| AC004463.6 | Yes |
| AC012442.5 | Yes |
| AC012442.6 | Yes |
| AC079305.8 | Yes |
| AC087294.2 | Yes |
| AE000662.93 | Yes |
| BOLA3-AS1 | Yes |
| CDC42-IT1 | Yes |
| CTB-13F3.1 | Yes |
| CTC-332L22.1 | Yes |
| CTD-2231E14.5 | Yes |
| CTD-2315E11.1 | Yes |
| CTD-2382E5.1 | No |
| CTD-2516F10.4 | Yes |
| CTD-2616J11.16 | No |
| CTD-3074O7.5 | Yes |
| CTD-3154N5.1 | Yes |
| CTD-3185P2.1 | No |
| ILF3-AS1 | Yes |
| KCNIP2-AS1 | No |
| LINC00938 | Yes |
| MIR1539 | Yes |
| MYLK-AS1 | No |
| PCAT6 | Yes |
| RGMB-AS1 | Yes |
| RNF185-AS1 | Yes |
| RP1-125I3.2 | No |
| RP1-168L15.5 | Yes |
| RP1-39G22.7 | Yes |
| RP11-1094M14.14 | No |
| RP11-111K18.2 | Yes |
| RP11-112H10.4 | No |
| RP11-141O15.1 | Yes |
| RP11-146F11.5 | Yes |
| RP11-158H5.7 | No |
| RP11-15E18.1 | Yes |
| RP11-174G6.5 | Yes |
| RP11-195F19.9 | Yes |
| RP11-20E24.1 | Yes |
| RP11-224O19.2 | Yes |
| RP11-22P6.2 | Yes |
| RP11-24N18.1 | Yes |
| RP11-253M7.4 | Yes |
| RP11-256I23.3 | Yes |
| RP11-256P1.1 | Yes |
| RP11-279F6.1 | Yes |
| RP11-27K13.3 | Yes |
| RP11-293A21.1 | Yes |
| RP11-332H14.2 | Yes |
| RP11-347C12.10 | Yes |
| RP11-353K11.1 | Yes |
| RP11-359B12.2 | Yes |
| RP11-379C10.4 | Yes |
| RP11-390K5.6 | Yes |
| RP11-394I13.3 | Yes |
| RP11-395N3.1 | Yes |
| RP11-395P17.3 | No |
| RP11-398C13.6 | Yes |
| RP11-407G23.4 | Yes |
| RP11-40H20.4 | Yes |
| RP11-417O11.5 | Yes |
| RP11-443B20.1 | No |
| RP11-452L6.7 | Yes |
| RP11-45P15.4 | Yes |
| RP11-463I20.3 | Yes |
| RP11-465N4.4 | Yes |
| RP11-474N24.6 | Yes |
| RP11-483A20.3 | No |
| RP11-519G16.2 | No |
| RP11-552F3.13 | Yes |
| RP11-554J4.1 | Yes |
| RP11-571L19.7 | No |
| RP11-635L1.3 | Yes |
| RP11-666A8.7 | Yes |
| RP11-778D9.13 | Yes |
| RP11-77K12.3 | Yes |
| RP11-798M19.6 | Yes |
| RP11-7O11.3 | Yes |
| RP11-816J6.3 | Yes |
| RP11-84A14.4 | Yes |
| RP11-91A18.4 | No |
| RP11-96D1.11 | Yes |
| RP11-96D1.6 | Yes |
| RP11-96D1.7 | Yes |
| RP11-98I9.4 | Yes |
| RP3-325F22.5 | Yes |
| RP3-483K16.4 | Yes |
| RP4-758J18.13 | Yes |
| RP4-773A18.4 | Yes |
| RP4-789D17.5 | Yes |
| RP4-816N1.7 | Yes |
| RP5-1065J22.8 | Yes |
| SEC24B-AS1 | Yes |
| SMG7-AS1 | Yes |
| TERC | Yes |
| TOB1-AS1 | Yes |
| TRAM2-AS1 | Yes |
| UCHL1-AS1 | Yes |
| VIM-AS1 | Yes |
