# Supplementary material for: FunlncModel: integrating multi-omic features from upstream and downstream regulatory networks into a machine learning framework to identify functional lncRNAs
Source: Brief Bioinform. 2024 Nov 27;26(1):bbae623. doi: 10.1093/bib/bbae623 (PMC11601888; doi:10.1093/bib/bbae623)
Supplement: Supplementary_Table15_bbae623 [file supplementary_table15_bbae623.docx]

| Supplementary Table 15. The list of known functional lncRNAs that influence the states of stem cells | | | |
| --- | --- | --- | --- |
| Name | FunlncModel probability | FunlncModel | LNCipedia (High confidence set) |
| A1BG-AS1 | 0.55 | Yes | Yes |
| AC005592.2 | 0.854 | Yes | Yes |
| AC007246.3 | 0.93 | Yes | Yes |
| AC012146.7 | 0.818 | Yes | Yes |
| AC108488.3 | 0.922 | Yes | NO |
| CTC-228N24.3 | 0.608 | Yes | Yes |
| CTD-2006C1.2 | 0.85 | Yes | Yes |
| CTD-2366F13.1 | 0.746 | Yes | NO |
| CTD-2587H24.5 | 0.716 | Yes | NO |
| DANCR | 0.784 | Yes | Yes |
| LINC00657 | 0.786 | Yes | NO |
| LINC00886 | 0.834 | Yes | NO |
| LINC00938 | 0.974 | Yes | Yes |
| RAB30-AS1 | 0.89 | Yes | NO |
| RP11-110G21.1 | 0.72 | Yes | Yes |
| RP11-195F19.9 | 0.974 | Yes | Yes |
| RP11-253M7.1 | 0.946 | Yes | Yes |
| RP11-395B7.4 | 0.61 | Yes | Yes |
| RP11-473M20.14 | 0.91 | Yes | Yes |
| RP11-48O20.4 | 0.536 | Yes | Yes |
| RP11-545E17.3 | 0.84 | Yes | Yes |
| RP11-674N23.1 | 0.858 | Yes | Yes |
| RP13-20L14.6 | 0.786 | Yes | Yes |
| RP3-510D11.2 | 0.61 | Yes | Yes |
| RP5-1050D4.5 | 0.916 | Yes | NO |
| RP5-886K2.3 | 0.96 | Yes | Yes |
| RSBN1L-AS1 | 0.878 | Yes | NO |
| SBF2-AS1 | 0.822 | Yes | NO |
| TERC | 0.996 | Yes | Yes |
| TMEM9B-AS1 | 0.846 | Yes | Yes |
| ZBED5-AS1 | 0.93 | Yes | NO |
| AC007879.7 | 0.428 | NO | NO |
| DNM3OS | 0.44 | NO | Yes |
| EMX2OS | 0.304 | NO | Yes |
| LINC00667 | 0.424 | NO | Yes |
| LINC00702 | 0.19 | NO | Yes |
| LINC00968 | 0.122 | NO | NO |
| PTPRG-AS1 | 0.46 | NO | NO |
| TP53TG1 | 0.478 | NO | Yes |
