# Supplementary material for: FunlncModel: integrating multi-omic features from upstream and downstream regulatory networks into a machine learning framework to identify functional lncRNAs
Source: Brief Bioinform. 2024 Nov 27;26(1):bbae623. doi: 10.1093/bib/bbae623 (PMC11601888; doi:10.1093/bib/bbae623)
Supplement: Supplementary_MATERIALS_AND_METHODS_bbae623 [file supplementary_materials_and_methods_bbae623.docx]

**Supplementary MATERIALS AND METHODS**

**Supplementary Note 1. Constructing (epi)genetic regulatory network of lncRNAs.**

***Identification of SEs and TEs regulating lncRNAs.*** The ChIP-seq datasets of H3K27ac and matching input DNA were downloaded from NCBI GEO/SRA(1), ENCODE(2), Roadmap(3) and GGR (Genomics of Gene Regulation Project)(2). We retained 82 datasets from them that contained information on four cellular contexts (hESC, breast-, colon-, and lung cancer), and used the streamlined pipeline of Bowtie-MACS-ROSE to identify the TEs/SEs that regulate lncRNAs(4-9). Specifically, each H3K27ac ChIP-seq data was aligned to the reference genome using Bowtie with default parameters. MACS was used to call peaks, and the ROSE algorithm (ROSE_geneMapper.py) was used to identify the TE/SE regions as well as the regulatory relationships between these TEs/SEs and lncRNAs(10) (Figure 1A left panel, Table S3).

***Identification of the regulatory relationship between accessible chromatin regions and lncRNAs.*** We manually collected the ATAC-seq datasets with the keyword of “ATAC-seq” from NCBI GEO/SRA(1). To control the normalization and consistency, we used the streamlined pipeline of Trim Galore-Bowtie2-Picard-MACS2 to identify the chromatin accessibility region(6,11,12). MACS2 was used to identify accessible chromatin regions with the following parameters: ‘-broad -SPMR -nomodel -extsize 200 -q 0.01’. In addition, we discarded some accessible chromatin regions that overlapped with the ENCODE blacklisted regions(2). To reduce the influence of dataset sparsity, we further collected the DNase-seq and ATAC-seq datasets from ENCODE(2), Roadmap(3) and Cistrome(13). Finally, a total of 124 datasets were used to predict regulatory relationships between accessible chromatin regions and lncRNAs (ROSE_geneMapper.py) (Figure 1A left panel, Table S4).

***Identification of TFs associated with lncRNAs.*** The ChIP-seq technique has been proven to be an effective high-throughput strategy for identifying TF targets. To identify the regulatory relationships between lncRNAs and TFs, we first obtained the TF binding sites (TFBS) by collecting 2,096 ChIP-seq datasets of 227 TFs from ENCODE(2), Remap(14), Cistrome(13), ChIP-Atlas(15) and GTRD(16). We defined the region that encompassed 10 kb upstream and 1 kb downstream from the transcription start site(17) of lncRNA as the promoter region. We then constructed TFs-lncRNAs pairs by applying the BEDtools software(18), when the TFBS overlapped with promoter regions of lncRNAs in the matching cell/tissue types. Furthermore, we used Find Individual Motif Occurrences (FIMO) and DNA-binding sequence motifs of ∼700 TFs to predict the relationships between the TFs and regulatory regions with a strict p-value threshold of 1e-6(19,20). Overall, the regulatory relationships between TFs and lncRNAs were identified via the above two strategies (Figure 1A left panel, Table S5).

***Identification of lncRNA-associated histone modification with the transcriptional activation function****.* In total, *79* ChIP-seq datasets of six transcriptional activation histone modifications (H3K36me3, H3K4me3, H3K4me1, H3K79me2, H3K9ac, and H4K20me1) were collected from ENCODE(2) and Roadmap(14), which covered 12,205,512 peaks. BEDTools was used to check for overlaps between the peaks of histone modification and the promoter regions of lncRNAs in the matching cell/tissue types to identify lncRNA-related histone modifications (Figure 1A left panel, Table S6).

***Identification of SNPs and methylation sites occurring in lncRNAs.*** We collected common SNPs from dbSNP(21) and used the VCFTools (v0.1.13)(22) to calculate their allelic frequency. A total of 38,063,729 common SNPs with a minimum allelic frequency (MAF) > 0.05 were thus obtained(23). We also collected a total of 264,514 risk SNPs from the GWAS Catalog(24) and GWASdbv2.0(25). Furthermore, we downloaded 7 DNA methylation 450k array data from ENCODE(2) and discarded sits with beta-values < 0.2. The above-mentioned SNPs and methylation sites were matched in the promoter regions by using BEDTools to identify the regulatory relationships (Figure 1A left panel) (Table S7).

**Supplementary Note 2. The description for complex feature variables.**

***Regulatory Element and lncRNA associations:*** we take the union strategies of RE and lncRNA associations, inferred by gene_mapper (Dis), inferred by global chromatin interaction (Gci), and inferred by intersection of the two strategies (Dis $\cap$ Gci). Beyond the number feature (${Dis\_SE}_{num}$, ${Dis\_TE}_{num}$, ${Dis\_DHS}_{num}$, ${Dis\_ATAC}_{num}$...), we further calculated the other features of lncRNA-associated-REs, for instance, the element number, rank, and signal.

1. **The median element number feature of lncRNA-associated-SEs inferred by gene_mapper**: ${Dis\_SE}_{ele}$, where $i=\left\{ 1,2,...I \right\}$ is set of lncRNA-associated-SEs and ${Dis\_SE}_{ele,i}$ represent the element number of i th lncRNA-associated-SE.
2. **The rank feature of lncRNA-associated-SEs inferred by gene_mapper:** ${Dis\_SE}_{rank}$, where $\mathrm{SE}_{\mathrm{rank}}$ is set of all lncRNA-associated-SEs rank in S sample.

$${Dis\_SE}_{rank}=max\left( {SE}_{rank} \right)-median\left( {{Dis\_SE}_{rank,1},{Dis\_SE}_{rank,2},...Dis\_SE}_{rank,I} \right)$$

3. **The mean ChIP-seq signal feature of lncRNA-associated-SEs inferred by gene_mapper:** ${Dis\_SE}_{signal}$., where ${SE}_{signal,i}$ represent the ChIP-seq signal value of i th lncRNA-associated-SE.
4. **The median element number feature of lncRNA-associated-TEs inferred by global chromatin interaction:** ${Gci\_TE}_{ele}$, where $j=\left\{ 1,2,...J \right\}$ is set of lncRNA-associated-TEs and ${Gci\_TE}_{ele,j}$ represent the element number of j th lncRNA-associated-TE.
5. **The rank feature of lncRNA-associated-TEs inferred by global chromatin interaction:** ${Gci\_TE}_{rank}$, where $\mathrm{TE}_{\mathrm{rank}}$ is set of all lncRNA-associated-TEs rank in S sample.

$${Gci\_TE}_{rank}=max\left( {TE}_{rank} \right)-median\left( {{Gci\_TE}_{rank,1},{Gci\_TE}_{rank,2},...Gci\_TE}_{rank,J} \right)$$

6. **The mean ChIP-seq signal feature of lncRNA-associated-TEs inferred by global chromatin interaction:** ${Gci\_TE}_{signal}$., where ${TE}_{signal,j}$ represent the ChIP-seq signal value of j th lncRNA-associated-TE.
7. **The median element number feature of lncRNA-associated-SEs inferred by gene_mapper and global chromatin interaction:** ${Dis\cap Gci\_SE}_{ele}$, where $z=\left\{ 1,2,...Z \right\}$ is set of lncRNA-associated-SEs and ${Dis\cap Gci\_SE}_{ele,z}$ represent the element number of z th lncRNA-associated-SE.
8. **The rank feature of lncRNA-associated-SEs inferred by gene_mapper and global chromatin interaction:** ${Dis\cap Gci\_SE}_{rank}$, where $\mathrm{SE}_{\mathrm{rank}}$ is set of all lncRNA-associated-SEs rank in S sample.
9. **e mean ChIP-seq signal feature of lncRNA-associated-SEs inferred by gene_mapper and global chromatin interaction:** ${Dis\cap Gci\_SE}_{signal}$., where ${SE}_{signal,z}$ represent the ChIP-seq signal value of z th lncRNA-associated-SE.

***Methylation sits of jth lncRNA promoter:*** ${MS}_{num,j}$*,* where $n=\left\{ 1,2,...N \right\}$ is set of methylation sit*s (*beta>0.2) in jth lncRNA promoter.

***ethylation signal strength of jth lncRNA promoter:*** ${MS}_{j}$*,* where $n=\left\{ 1,2,...N \right\}$ is set of methylation sit*s (*beta>0.2) in jth lncRNA promoter, beta value is percentage of the methylation signal strength of one of the methylation sits, *u* is the non-methylation signal strength, *m* is the methylation signal strength.

$${MS}_{j}=max\left( {MS}_{1,2\cdots j} \right)-\frac{10}{N}\sum_{n}^{N} beta\#\left( 9 \right)$$

***Summary.*** FunlncModel focused on integrating a large number of multi-omic features from the upstream/downstream multi-omic regulatory networks and combining with the random forest algorithm to identify high-confidence functional lncRNAs. These features were further processed to reflect their biological significance in terms of dominating the functional processes of lncRNAs, as well as interpret their complex regulatory mechanisms underlying the functions of lncRNAs (see Table 1, Table S1). Briefly, we collected a mass of relationships and more than 2,000 datasets across 11 data types, including TFs, histone modifications, TEs, SEs, chromatin accessibility regions, chromatin interaction, SNPs, methylation sites, mRNAs, miRNAs, and proteins (Figure 1, Table 1, Table S2-8). After sufficiently leveraging these upstream/downstream transcriptional regulatory relationships and the topological properties of the network, we generated up to 57 features as the input to FunlncModel, which were not well considered by the other existing methods (Figure 1, Table 1, Table S2-8). Moreover, these features were categorized into three major types based on their specific transcriptional regulatory mechanisms: (i) those associated with the upstream proximal regulation of lncRNAs; (ii) those associated with the upstream distant regulation of lncRNAs; and (iii) those associated with the downstream regulation of lncRNAs, as follows (see Table 1, Table S1):

***Feature category 1 (C1):*** *Features associated with* *upstream proximal regulation.* For upstream proximal regulation of lncRNAs, we focused mainly on the regulatory elements in the promoter regions (10kb/1kb), including 6 types of histone modifications, ~700 TFs, methylation sites and SNPs (see Table 1, Table S1). Indeed, many studies have described and emphasized these regulatory elements' functions in influencing and mediating the transcriptional regulatory processes of lncRNAs(26-31). Therefore, we collected a large number of (epi)genetic datasets to improve the functional lncRNAs identification. We retained samples corresponding to the four cellular contexts through manual processing and filtering (Table S5-7). Based on the regulatory networks, we then quantified the upstream proximal regulation of lncRNAs according to their regulatory mechanisms and topological properties in networks, such as their indegree, type and the normalized signal strength of the neighbor nodes (see Table 1, Table S1).

***Feature category 2 (C2):*** *Features associated with upstream distal regulation.* It is well known that super-enhancers/typical-enhancers and accessible chromatin regions usually influence the lncRNA expression and function in distal regulatory manner. Nevertheless, the role of the distal regulatory element has often been ignored in existing methods. To comprehensively capture upstream features and predict functional lncRNAs, we thus collected a large amount of ChIP-seq, ATAC-seq, and DNase-seq data (Table 1, Table S1-4). After filtering them, we processed these retained data by using streamlined pipelines to obtain the region information, and signal strength (see Table 1, Table S1-4). The distant regulatory relationships identification mainly included distance-based (ROSE_geneMapper.py) and 3D chromatin interaction-based (e.g., ChIA-PET 3C, 4C, 5C and Hi-C). We adopted these two strategies (distance- and chromatin interaction-based) to generate distant feature classes (super-enhancers, typical-enhancers and accessible chromatin regions) in multiple combinations. As described in Methods and Supplementary Note1, the upstream distal regulatory landscape of lncRNAs was drawed based on their topological properties in networks, such as the degree of nodes, mean signal strength and normalized rank of the distal regulatory elements.

***Feature category 3 (C3):*** *Features associated with* *downstream regulation.* Compared with upstream regulation, the downstream regulation of lncRNAs has been extensively studied, and many databases provide comprehensive lncRNA-related regulatory information. We collected lncRNA-protein, -miRNA, and -mRNA interactions from published databases LncRNA2Target v2.0(32), starBase v2.0(33), LncACTdb 2.0(34), and EuRBPDB(35). Based on the post-transcriptional regulatory network of lncRNA, we calculated their node outdegree as values of the input features, respectively (see Table 1, Table S1).

**Supplementary Note 3. Overview of the random forest method.**

The random forest model is an ensemble classifier that integrated multiple decision trees. Each tree in the forest has access to only a subset of the training data in the sampled-with-replacement manner, and to a random subset of features in the sampled-without-replacement manner. To estimate the feature importance values, the model calculated the Gini impurity of each tree as well as the sums of weighted reduction in it when splitting on each feature across all trees during iterative optimization. The formula for calculating the Gini impurity was as follows:

where $p_{k}$ was the probability of a certain classification $k$.

For all the models explored, a fifth of the datasets was held out for independent test sets and 10-fold cross-validation was performed on the remaining part. In each round, one of the 10 equal subsets was used as the test set and the remaining subsets were used as the training set. For each decision tree $T_{i}$, we can construct its classification function $f_{i}\left( x \right)$ based on the training set $D$. The final classification of the random forest was the voting result of all decision trees:

where $y$ was the classification result of the random forest for the input sample $x$, $n$ was the number of decision trees, $w_{i}$ was the weight of the $i$ th decision tree ( the default was 1/n).

In addition, the requisite pre-processing transformation (centering, scaling) was estimated from the training data and applied to any dataset with the same variables by using *preProcess* of *R* package *caret.* The simple backward selection method of recursive feature elimination (REF) was utilized to estimate the n optimal features from the 57 features mentioned above (see Table 1, Table S1). It tuned the models by choosing complex parameters that were associated with the optimal resampling-related statistic. Finally, the three categories of features mentioned above were efficiently utilized to construct the two classification models (HESC and Combiner) for prediction of functional lncRNAs related to hESC and cancer, respectively (Figure 1 middle panel, Table 1, Table S1).

**Supplementary Note 4. Building the Combiner model.**

We created a ‘combined’ dataset based on four cellular contexts (hESC, breast cancer, colon cancer, and lung cancer) to identify functional lncRNAs across them. The following procedures were carried out to create and train the Combiner model: We integrated the multi-omic network and further screened out the important features based on their specific transcriptional regulatory mechanisms in each cellular context, respectively (Table 1, Table S1). The roles of many lncRNAs in diseases have been discovered and confirmed, and they can be obtained from Lnc2Cancer v2.0(36), LncRNADisease v2.0(37), LncRNAWiki(38), CRlncRNA(39), EVLncRNAs(40), and LncRNADisease(41). Following the elimination of redundant data, we obtained positive combined sets containing 84 breast cancer-associated lncRNAs, 46 colon cancer-associated lncRNAs, and 57 lung cancer-associated lncRNAs. We then collected lncRNA expression data for various cancer types from CCLE(42). For each specific cancer type, lncRNAs exhibiting zero expression levels across the corresponding cancer samples were identified as candidate negative datasets (Table S13), which facilitated the identification of transcriptionally inactive lncRNAs in particular cancer types. After filtering and removing redundancy, we defined the final negative combined cases (Table S16), including 365 lncRNAs with non-expressive state in 60 breast cancer associated samples, 246 lncRNAs with non-expressive state in 69 colon cancer associated samples, and 167 lncRNAs with non-expressive state in 150 lung cancer associated samples. In each case, one-fifth of the data were held out for testing and 10-fold cross-validation was performed on the remaining part. To ensure the uniform distribution of data, the test sets were randomly extracted according to the four cellular contexts, respectively (Figure 1C right panel). We further standardized and transformed the training dataset, screened a subset of predictors that could be used to produce an accurate model, and finally trained the random forest model by using the open-source *R* package *caret.*

**Supplementary Note 5. Performance evaluation metrics.**

For a binary decision problem, the decision made by the classifier can be represented in a structure known as a confusion matrix, including the true positives (TPs), false positives (FPs), true negatives (TNs), and false negatives (FNs). Given a confusion matrix, we used the *R* package (*ROCR, PRROC,* and *precrec*) to construct and generate the Receiver Operating Characteristic curve (ROC) and the precision-recall curve (PR)(43-45). The False Positive Rate (FPR) was along the x-axis and the true positive rate (TPR) was along the y-axis in the ROC space, and the recall was plotted along the x-axis and the precision along the y-axis in the PR space. The area under the curve (AUC) was used as a simple metric to objectively evaluate the performance of the models. We used the composite trapezoidal method to calculate the area under the receiver operating characteristic curve (AUROC) and the area under the precision-recall curve (AUPRC) to evaluate the performance of the model. The higher AUROC and AUPRC stand for better performance (Table S9). These indicators were defined as follows:

where $\mathrm{Precision}$ was the corresponding precision value when recall value is $r$.

where $\mathrm{TPR}$ and $\mathrm{FPR}$ represented the true positive rate and false positive rate values under the corresponding thresholds.

**Supplementary Note 6. Enrichment analysis based on expression data**

The samples from GSE122380 contained data on the state of undifferentiation and the cardiomyocyte of the 15-day state of differentiation(46,47). We first calculated Spearman’s rank correlation coefficients between each lncRNA and important genes of signaling pathways that regulate the pluripotency of stem cells (hsa04550). The sum of the absolute values of these correlation coefficients was assigned as the final input value for each lncRNA, respectively. Finally, the Gene Set Enrichment Analysis (GSEA) tool was applied to calculate the enrichment scores and estimate their statistical significance levels, where this reflected the distribution of rankings of the input set for all background lncRNAs(48). We randomly selected lncRNA sets with similar expression values as the control group.

**Supplementary Note 7. Enrichment analysis based on drug influence and Cancer survival information**

We first collected information on treatment drugs (Lapatinib, Topotecan and Irinotecan) for three types of cancer from lncMAP(49). The -log10 p-values (p-values of the correlation between each lncRNA and the information on these drugs) were further assigned as the value of assessment for each lncRNA. We then obtained cancer survival information of lncRNAs from lnCAR(50), and further assigned -log10 p-values (p-value of the correlation between the expression of each lncRNA and the information on cancer survival) as the value of assessment for each lncRNA. Based on these input values, we further used the GSEA tool to analyze and estimate their distributions in all lncRNAs.

**Supplementary Note 8. Scores of evolutionary conservation.**

The evolutionary conservations of promoter region of three categories lncRNAs (Fun_lncs, HCFun_lncs and Non_Fun_lncs) using the phastCons scores of the UCSC Genome Browser (51,52). This process was performed using R packages (phastCons100way.UCSC.hg19 and GenomicFeatures).

**Supplementary Note 9. Phenotype and disease analysis.**

To investigate these HCFun_lncs whether conducted even more important influences for diseases than the other two categories lncRNAs, we collected involved cancer phenotype from Cancer RNA-Seq Nexus (53,54) and associated diseases from multiple sources, including LncRNADisease 2.0 (55), Lnc2Cancer v2.0 (36), LncRNAWiki (38), EVLncRNAs (40), lncRNASNP2 (56), CRlncRNA (39) and HDncRNA (57).

**Supplementary Note 10. IGV visualization of histone modifications.**

To clear the change of its transcription activity during differentiation processes, we collected histone modifications bigwig format signal files of HESC and HESC-derived-cardiomyocyte from ENCODE (2), including H3K27ac, H3K4me3, H3K4me1 and H3K27me3 (the inhibiting signal). Based on IGV visualization (58), it is apparent that the promoter region of RP4-792G4.2 was enriched with higher active signal (H3K27ac, H3K4me3 and H3K4me1) and lower inhibiting signal (H3K27me3) in HESC comparing to cardiomyocyte.

**Supplementary Note 11. Comparative assessment with other methods**

For benchmarking, we implemented the LncFunNet and the co-expression methods. LncFunNet focused on mouse species and did not provide the corresponding data of human. We thus collected and processed hESC-associated data as described in Ref(59) to generate inputs to LncFunNet. We first collected human TF ChIP-seq files and the AGO2 PAR-CLIP peak file(13,33) even though the latter was not required for our approach. We then collected a series of raw RNA-seq data of iPSC samples from GSE122380(60), and used the Tophat2-Cufflinks pipeline to generate expression matrix with FPKM values(46,47). These files served as the input of LncFunTK tool, a webtool for the LncFunNet approach. Finally, we obtained the functional information score (FIS) of each lncRNA as the evaluation index of the ROC and PR curves.

For co-expression approach, the statistical correlation between each lncRNA and the positive training sets was calculated by using the expression matrix mentioned above(46,47,60). For each evaluated lncRNA, the sum of the absolute values of Spearman’s rank correlation coefficients was used to calculate the AUROC and AUPRC values(61). The formula for Spearman’s rank correlation coefficient ($\rho$) was given below, where $d_{i}$ was the difference between the ranks of the $i$th pair, and $n$ was the number of observed samples.

**REFERENCES**

1. Barrett, T., Troup, D.B., Wilhite, S.E., Ledoux, P., Evangelista, C., Kim, I.F., Tomashevsky, M., Marshall, K.A., Phillippy, K.H., Sherman, P.M. *et al.* (2011) NCBI GEO: archive for functional genomics data sets--10 years on. *Nucleic Acids Res*, **39**, D1005-1010.

2. Consortium, E.P. (2012) An integrated encyclopedia of DNA elements in the human genome. *Nature*, **489**, 57-74.

3. Bernstein, B.E., Stamatoyannopoulos, J.A., Costello, J.F., Ren, B., Milosavljevic, A., Meissner, A., Kellis, M., Marra, M.A., Beaudet, A.L., Ecker, J.R. *et al.* (2010) The NIH Roadmap Epigenomics Mapping Consortium. *Nat Biotechnol*, **28**, 1045-1048.

4. Langmead, B., Trapnell, C., Pop, M. and Salzberg, S.L. (2009) Ultrafast and memory-efficient alignment of short DNA sequences to the human genome. *Genome Biol*, **10**, R25.

5. Loven, J., Hoke, H.A., Lin, C.Y., Lau, A., Orlando, D.A., Vakoc, C.R., Bradner, J.E., Lee, T.I. and Young, R.A. (2013) Selective inhibition of tumor oncogenes by disruption of super-enhancers. *Cell*, **153**, 320-334.

6. Zhang, Y., Liu, T., Meyer, C.A., Eeckhoute, J., Johnson, D.S., Bernstein, B.E., Nusbaum, C., Myers, R.M., Brown, M., Li, W. *et al.* (2008) Model-based analysis of ChIP-Seq (MACS). *Genome Biol*, **9**, R137.

7. Jiang, Y., Qian, F., Bai, X., Liu, Y., Wang, Q., Ai, B., Han, X., Shi, S., Zhang, J., Li, X. *et al.* (2019) SEdb: a comprehensive human super-enhancer database. *Nucleic Acids Res*, **47**, D235-D243.

8. Qian, F.C., Li, X.C., Guo, J.C., Zhao, J.M., Li, Y.Y., Tang, Z.D., Zhou, L.W., Zhang, J., Bai, X.F., Jiang, Y. *et al.* (2019) SEanalysis: a web tool for super-enhancer associated regulatory analysis. *Nucleic Acids Res*, **47**, W248-W255.

9. Tang, Z., Li, X., Zhao, J., Qian, F., Feng, C., Li, Y., Zhang, J., Jiang, Y., Yang, Y., Wang, Q. *et al.* (2019) TRCirc: a resource for transcriptional regulation information of circRNAs. *Brief Bioinform*, **20**, 2327-2333.

10. Li, Y., Li, X., Yang, Y., Li, M., Qian, F., Tang, Z., Zhao, J., Zhang, J., Bai, X., Jiang, Y. *et al.* (2021) TRlnc: a comprehensive database for human transcriptional regulatory information of lncRNAs. *Brief Bioinform*, **22**, 1929-1939.

11. Langmead, B. and Salzberg, S.L. (2012) Fast gapped-read alignment with Bowtie 2. *Nat Methods*, **9**, 357-359.

12. Wang, F., Bai, X., Wang, Y., Jiang, Y., Ai, B., Zhang, Y., Liu, Y., Xu, M., Wang, Q., Han, X. *et al.* (2021) ATACdb: a comprehensive human chromatin accessibility database. *Nucleic Acids Res*, **49**, D55-D64.

13. Mei, S., Qin, Q., Wu, Q., Sun, H., Zheng, R., Zang, C., Zhu, M., Wu, J., Shi, X., Taing, L. *et al.* (2017) Cistrome Data Browser: a data portal for ChIP-Seq and chromatin accessibility data in human and mouse. *Nucleic Acids Res*, **45**, D658-D662.

14. Cheneby, J., Gheorghe, M., Artufel, M., Mathelier, A. and Ballester, B. (2018) ReMap 2018: an updated atlas of regulatory regions from an integrative analysis of DNA-binding ChIP-seq experiments. *Nucleic Acids Res*, **46**, D267-D275.

15. Oki, S., Ohta, T., Shioi, G., Hatanaka, H., Ogasawara, O., Okuda, Y., Kawaji, H., Nakaki, R., Sese, J. and Meno, C. (2018) ChIP-Atlas: a data-mining suite powered by full integration of public ChIP-seq data. *EMBO Rep*, **19**.

16. Yevshin, I., Sharipov, R., Valeev, T., Kel, A. and Kolpakov, F. (2017) GTRD: a database of transcription factor binding sites identified by ChIP-seq experiments. *Nucleic Acids Res*, **45**, D61-D67.

17. Ramilowski, J.A., Yip, C.W., Agrawal, S., Chang, J.C., Ciani, Y., Kulakovskiy, I.V., Mendez, M., Ooi, J.L.C., Ouyang, J.F., Parkinson, N. *et al.* (2020) Functional annotation of human long noncoding RNAs via molecular phenotyping. *Genome Res*, **30**, 1060-1072.

18. Quinlan, A.R. and Hall, I.M. (2010) BEDTools: a flexible suite of utilities for comparing genomic features. *Bioinformatics*, **26**, 841-842.

19. Grant, C.E., Bailey, T.L. and Noble, W.S. (2011) FIMO: scanning for occurrences of a given motif. *Bioinformatics*, **27**, 1017-1018.

20. Bailey, T.L., Boden, M., Buske, F.A., Frith, M., Grant, C.E., Clementi, L., Ren, J., Li, W.W. and Noble, W.S. (2009) MEME SUITE: tools for motif discovery and searching. *Nucleic Acids Res*, **37**, W202-208.

21. Sherry, S.T., Ward, M.H., Kholodov, M., Baker, J., Phan, L., Smigielski, E.M. and Sirotkin, K. (2001) dbSNP: the NCBI database of genetic variation. *Nucleic Acids Res*, **29**, 308-311.

22. Danecek, P., Auton, A., Abecasis, G., Albers, C.A., Banks, E., DePristo, M.A., Handsaker, R.E., Lunter, G., Marth, G.T., Sherry, S.T. *et al.* (2011) The variant call format and VCFtools. *Bioinformatics*, **27**, 2156-2158.

23. Genomes Project, C., Abecasis, G.R., Auton, A., Brooks, L.D., DePristo, M.A., Durbin, R.M., Handsaker, R.E., Kang, H.M., Marth, G.T. and McVean, G.A. (2012) An integrated map of genetic variation from 1,092 human genomes. *Nature*, **491**, 56-65.

24. Welter, D., MacArthur, J., Morales, J., Burdett, T., Hall, P., Junkins, H., Klemm, A., Flicek, P., Manolio, T., Hindorff, L. *et al.* (2014) The NHGRI GWAS Catalog, a curated resource of SNP-trait associations. *Nucleic Acids Res*, **42**, D1001-1006.

25. Li, M.J., Liu, Z., Wang, P., Wong, M.P., Nelson, M.R., Kocher, J.P., Yeager, M., Sham, P.C., Chanock, S.J., Xia, Z. *et al.* (2016) GWASdb v2: an update database for human genetic variants identified by genome-wide association studies. *Nucleic Acids Res*, **44**, D869-876.

26. Aich, M. and Chakraborty, D. (2020) Role of lncRNAs in stem cell maintenance and differentiation. *Curr Top Dev Biol*, **138**, 73-112.

27. Xie, W., Song, C., Young, N.L., Sperling, A.S., Xu, F., Sridharan, R., Conway, A.E., Garcia, B.A., Plath, K., Clark, A.T. *et al.* (2009) Histone h3 lysine 56 acetylation is linked to the core transcriptional network in human embryonic stem cells. *Mol Cell*, **33**, 417-427.

28. Jain, A.K., Xi, Y., McCarthy, R., Allton, K., Akdemir, K.C., Patel, L.R., Aronow, B., Lin, C., Li, W., Yang, L. *et al.* (2016) LncPRESS1 Is a p53-Regulated LncRNA that Safeguards Pluripotency by Disrupting SIRT6-Mediated De-acetylation of Histone H3K56. *Mol Cell*, **64**, 967-981.

29. Jiang, Y., Jiang, Y.Y., Xie, J.J., Mayakonda, A., Hazawa, M., Chen, L., Xiao, J.F., Li, C.Q., Huang, M.L., Ding, L.W. *et al.* (2018) Co-activation of super-enhancer-driven CCAT1 by TP63 and SOX2 promotes squamous cancer progression. *Nat Commun*, **9**, 3619.

30. Zhong, Q., Lu, M., Yuan, W., Cui, Y., Ouyang, H., Fan, Y., Wang, Z., Wu, C., Qiao, J. and Hang, J. (2021) Eight-lncRNA signature of cervical cancer were identified by integrating DNA methylation, copy number variation and transcriptome data. *J Transl Med*, **19**, 58.

31. Hua, J.T., Ahmed, M., Guo, H., Zhang, Y., Chen, S., Soares, F., Lu, J., Zhou, S., Wang, M., Li, H. *et al.* (2018) Risk SNP-Mediated Promoter-Enhancer Switching Drives Prostate Cancer through lncRNA PCAT19. *Cell*, **174**, 564-575 e518.

32. Cheng, L., Wang, P., Tian, R., Wang, S., Guo, Q., Luo, M., Zhou, W., Liu, G., Jiang, H. and Jiang, Q. (2019) LncRNA2Target v2.0: a comprehensive database for target genes of lncRNAs in human and mouse. *Nucleic Acids Res*, **47**, D140-D144.

33. Li, J.H., Liu, S., Zhou, H., Qu, L.H. and Yang, J.H. (2014) starBase v2.0: decoding miRNA-ceRNA, miRNA-ncRNA and protein-RNA interaction networks from large-scale CLIP-Seq data. *Nucleic Acids Res*, **42**, D92-97.

34. Wang, P., Li, X., Gao, Y., Guo, Q., Wang, Y., Fang, Y., Ma, X., Zhi, H., Zhou, D., Shen, W. *et al.* (2019) LncACTdb 2.0: an updated database of experimentally supported ceRNA interactions curated from low- and high-throughput experiments. *Nucleic Acids Res*, **47**, D121-D127.

35. Liao, J.Y., Yang, B., Zhang, Y.C., Wang, X.J., Ye, Y., Peng, J.W., Yang, Z.Z., He, J.H., Zhang, Y., Hu, K. *et al.* (2020) EuRBPDB: a comprehensive resource for annotation, functional and oncological investigation of eukaryotic RNA binding proteins (RBPs). *Nucleic Acids Res*, **48**, D307-D313.

36. Gao, Y., Wang, P., Wang, Y., Ma, X., Zhi, H., Zhou, D., Li, X., Fang, Y., Shen, W., Xu, Y. *et al.* (2019) Lnc2Cancer v2.0: updated database of experimentally supported long non-coding RNAs in human cancers. *Nucleic Acids Res*, **47**, D1028-D1033.

37. Bao, Z., Yang, Z., Huang, Z., Zhou, Y., Cui, Q. and Dong, D. (2019) LncRNADisease 2.0: an updated database of long non-coding RNA-associated diseases. *Nucleic Acids Res*, **47**, D1034-D1037.

38. Ma, L., Li, A., Zou, D., Xu, X., Xia, L., Yu, J., Bajic, V.B. and Zhang, Z. (2015) LncRNAWiki: harnessing community knowledge in collaborative curation of human long non-coding RNAs. *Nucleic Acids Res*, **43**, D187-192.

39. Wang, J., Zhang, X., Chen, W., Li, J. and Liu, C. (2018) CRlncRNA: a manually curated database of cancer-related long non-coding RNAs with experimental proof of functions on clinicopathological and molecular features. *BMC Med Genomics*, **11**, 114.

40. Zhou, B., Zhao, H., Yu, J., Guo, C., Dou, X., Song, F., Hu, G., Cao, Z., Qu, Y., Yang, Y. *et al.* (2018) EVLncRNAs: a manually curated database for long non-coding RNAs validated by low-throughput experiments. *Nucleic Acids Res*, **46**, D100-D105.

41. Chen, G., Wang, Z., Wang, D., Qiu, C., Liu, M., Chen, X., Zhang, Q., Yan, G. and Cui, Q. (2013) LncRNADisease: a database for long-non-coding RNA-associated diseases. *Nucleic Acids Res*, **41**, D983-986.

42. Barretina, J., Caponigro, G., Stransky, N., Venkatesan, K., Margolin, A.A., Kim, S., Wilson, C.J., Lehar, J., Kryukov, G.V., Sonkin, D. *et al.* (2012) The Cancer Cell Line Encyclopedia enables predictive modelling of anticancer drug sensitivity. *Nature*, **483**, 603-607.

43. Sing, T., Sander, O., Beerenwinkel, N. and Lengauer, T. (2005) ROCR: visualizing classifier performance in R. *Bioinformatics*, **21**, 3940-3941.

44. Grau, J., Grosse, I. and Keilwagen, J. (2015) PRROC: computing and visualizing precision-recall and receiver operating characteristic curves in R. *Bioinformatics*, **31**, 2595-2597.

45. Saito, T. and Rehmsmeier, M. (2017) Precrec: fast and accurate precision-recall and ROC curve calculations in R. *Bioinformatics*, **33**, 145-147.

46. Kim, D., Pertea, G., Trapnell, C., Pimentel, H., Kelley, R. and Salzberg, S.L. (2013) TopHat2: accurate alignment of transcriptomes in the presence of insertions, deletions and gene fusions. *Genome Biol*, **14**, R36.

47. Pollier, J., Rombauts, S. and Goossens, A. (2013) Analysis of RNA-Seq data with TopHat and Cufflinks for genome-wide expression analysis of jasmonate-treated plants and plant cultures. *Methods Mol Biol*, **1011**, 305-315.

48. Subramanian, A., Tamayo, P., Mootha, V.K., Mukherjee, S., Ebert, B.L., Gillette, M.A., Paulovich, A., Pomeroy, S.L., Golub, T.R., Lander, E.S. *et al.* (2005) Gene set enrichment analysis: a knowledge-based approach for interpreting genome-wide expression profiles. *Proc Natl Acad Sci U S A*, **102**, 15545-15550.

49. Li, Y., Li, L., Wang, Z., Pan, T., Sahni, N., Jin, X., Wang, G., Li, J., Zheng, X., Zhang, Y. *et al.* (2018) LncMAP: Pan-cancer atlas of long noncoding RNA-mediated transcriptional network perturbations. *Nucleic Acids Res*, **46**, 1113-1123.

50. Zheng, Y., Xu, Q., Liu, M., Hu, H., Xie, Y., Zuo, Z. and Ren, J. (2019) lnCAR: A Comprehensive Resource for lncRNAs from Cancer Arrays. *Cancer Res*, **79**, 2076-2083.

51. Siepel, A., Bejerano, G., Pedersen, J.S., Hinrichs, A.S., Hou, M., Rosenbloom, K., Clawson, H., Spieth, J., Hillier, L.W., Richards, S. *et al.* (2005) Evolutionarily conserved elements in vertebrate, insect, worm, and yeast genomes. *Genome Res*, **15**, 1034-1050.

52. Lee, B.T., Barber, G.P., Benet-Pages, A., Casper, J., Clawson, H., Diekhans, M., Fischer, C., Gonzalez, J.N., Hinrichs, A.S., Lee, C.M. *et al.* (2022) The UCSC Genome Browser database: 2022 update. *Nucleic Acids Res*, **50**, D1115-D1122.

53. Li, J.R., Sun, C.H., Li, W., Chao, R.F., Huang, C.C., Zhou, X.J. and Liu, C.C. (2016) Cancer RNA-Seq Nexus: a database of phenotype-specific transcriptome profiling in cancer cells. *Nucleic Acids Res*, **44**, D944-951.

54. Chen, J., Zhang, J., Gao, Y., Li, Y., Feng, C., Song, C., Ning, Z., Zhou, X., Zhao, J., Feng, M. *et al.* (2021) LncSEA: a platform for long non-coding RNA related sets and enrichment analysis. *Nucleic Acids Res*, **49**, D969-D980.

55. Wang, J., Ma, R., Ma, W., Chen, J., Yang, J., Xi, Y. and Cui, Q. (2016) LncDisease: a sequence based bioinformatics tool for predicting lncRNA-disease associations. *Nucleic Acids Res*, **44**, e90.

56. Miao, Y.R., Liu, W., Zhang, Q. and Guo, A.Y. (2018) lncRNASNP2: an updated database of functional SNPs and mutations in human and mouse lncRNAs. *Nucleic Acids Res*, **46**, D276-D280.

57. Wang, W.J., Wang, Y.M., Hu, Y., Lin, Q., Chen, R., Liu, H., Cao, W.Z., Zhu, H.F., Tong, C., Li, L. *et al.* (2018) HDncRNA: a comprehensive database of non-coding RNAs associated with heart diseases. *Database (Oxford)*, **2018**.

58. Thorvaldsdottir, H., Robinson, J.T. and Mesirov, J.P. (2013) Integrative Genomics Viewer (IGV): high-performance genomics data visualization and exploration. *Brief Bioinform*, **14**, 178-192.

59. Zhou, J., Zhang, S., Wang, H. and Sun, H. (2017) LncFunNet: an integrated computational framework for identification of functional long noncoding RNAs in mouse skeletal muscle cells. *Nucleic Acids Res*, **45**, e108.

60. Strober, B.J., Elorbany, R., Rhodes, K., Krishnan, N., Tayeb, K., Battle, A. and Gilad, Y. (2019) Dynamic genetic regulation of gene expression during cellular differentiation. *Science*, **364**, 1287-1290.

61. Sun, L., Luo, H., Liao, Q., Bu, D., Zhao, G., Liu, C., Liu, Y. and Zhao, Y. (2013) Systematic study of human long intergenic non-coding RNAs and their impact on cancer. *Sci China Life Sci*, **56**, 324-334.
